# Nasal oxytocin for the treatment of psychiatric disorders and pain: achieving meaningful brain concentrations

**DOI:** 10.1038/s41398-021-01511-7

**Published:** 2021-07-10

**Authors:** David C. Yeomans, Leah R. Hanson, Dean S. Carson, Brendan J. Tunstall, Mary R. Lee, Alexander Z. Tzabazis, Daniel Jacobs, William H. Frey

**Affiliations:** 1 Department of Anaesthesiology, Perioperative and Pain Medicine, School of Medicine, Stanford University, Stanford, CA USA; 2 Department of Neuroscience, HealthPartners Institute, Minneapolis, MN USA; 3 Trigemina, Inc., Moraga, CA USA; 4 Psychiatry and Behavioral Sciences, School of Medicine, Stanford University, Stanford, CA USA; 5 Department of Pharmacology, Addiction Science, and Toxicology, The University of Tennessee Health Science Center, Memphis, TN USA; 6 Veterans Affairs Medical Center, Washington, DC USA; 7 Department of Surgery, Permanente Medical Group, Santa Clara, CA USA

**Keywords:** Physiology, Neuroscience, Pharmacodynamics

## Abstract

There is evidence of the therapeutic potential of intranasal oxytocin for the treatment of pain and various psychiatric disorders, however, there is scant evidence that oxytocin reaches the brain. We quantified the concentration and distribution pattern of [^125^I]-radiolabeled oxytocin in the brains and peripheral tissues of rats after intranasal delivery using gamma counting and autoradiography, respectively. Radiolabel was detected in high concentrations in the trigeminal and olfactory nerves as well as in brain regions along their trajectories. Considerable concentrations were observed in the blood, however, relatively low levels of radiolabel were measured in peripheral tissues. The addition of a mucoadhesive did not enhance brain concentrations. These results provide support for intranasal OT reaching the brain via the olfactory and trigeminal neural pathways. These findings will inform the design and interpretation of clinical studies with intranasal oxytocin.

## Introduction

A growing body of research highlights the potential for the neuropeptide oxytocin (OT) in the treatment of a wide range of central nervous system (CNS) disorders, spanning autism to chronic pain [1, 2]. Oral delivery of peptide therapeutics leads to limited absorption due in part to degradation in the gut, injections are not favored for chronic daily use, and both delivery methods typically result in limited CNS penetration for large molecules like OT (molecular weight = 1007.19 Da) [3]. In order to circumvent the restrictions of the blood–brain barrier (BBB), many clinical researchers have utilized the noninvasive intranasal delivery route in the hopes of enhancing OT brain penetration by bypassing the BBB [4].

Intranasal delivery of large molecular weight drugs and proteins resulted in substantial brain penetration via transport along the perivascular space of blood vessels associated with olfactory and trigeminal nerves [5]. Thorne et al. [6] reported that intranasal, but not intravenous, delivery of radioiodinated insulin-like growth factor-I (7.65 kDa) rapidly (<30 min) resulted in significant brain penetration along extracellular olfactory and trigeminal nerve pathways. Similar results were found in nonhuman primates (cynomolgus monkeys) after intranasal administration of interferon-β1b (20 kDa) with the highest concentration of radioiodinated IFN-β1b found in the olfactory bulb as well as the basal ganglia [7]. Intranasal, compared with intravenous, administration of hypocretin (3.5 kDa) resulted in significantly greater tissue-to-blood concentrations in all brain regions measured over 2 h while blood concentrations were tenfold lower [8]. Thus, there is strong evidence supporting brain penetrance and limited systemic exposure after intranasal administration of therapeutic peptides.

Regarding OT, a number of recent studies have measured both blood and CSF OT concentrations following intranasal delivery of OT to nonhuman primates and demonstrated dose-dependent effects in blood OT concentration and activation of brain regions in humans [9, 10]. Lee et al. [11] administered intranasal, labeled (deuterated) OT (80 IU) to rhesus macaques and measured, by mass spectrometry, plasma and CSF concentrations of administered OT in the CSF and plasma. There were significant elevations of labeled OT in CSF and plasma over the 60-min sampling period after intranasal administration. These results built on previous studies in rhesus macaques where unlabeled OT was administered intranasally and significant elevations in CSF OT concentrations were observed after 40 [12] and 120 [13] min. In humans, Striepens et al. also reported significant elevation of CSF OT delivered intranasally after 75 min [14]. Thus, there is good evidence to support the delivery of OT to the CNS of nonhuman primates and humans after intranasal administration, although, it is important to note that the CSF recovery of the delivered OT dose in such studies is on the order of 0.001% [15, 16]. One possibility not tested in these aforementioned studies is that intranasal OT delivery bypasses the BBB and results in significantly greater elevations of administered OT in brain parenchyma compared to CSF.

Importantly, many studies that test the ability of OT to cross the BBB use CSF as a surrogate for determining distribution within the CNS and do not test for delivery to the brain parenchyma itself. It is important to note that CSF drug concentration is not always a good proxy for brain concentration for drugs administered intranasally. For example, Dhuria et al. [8] reported that the concentration of hypocretin in trigeminal nerves and olfactory bulbs was much higher than the hypocretin concentration in cisternal CSF 30 min after intranasal delivery compared to intravenous delivery. As regards OT, administration of intranasal OT resulted in a significant increase in OT concentrations in microdialysates taken from both the amygdala and hippocampus, however, there were no changes in ventricular CSF OT concentrations [17]. More recently, intranasal OT administered to OT-null mice resulted in a similar elevation in central OT concentrations in microdialysates, suggesting that the elevation is due solely to exogenous OT [18].

Measuring brain concentrations after intranasal administration of labeled OT, Lee et al. [19] reported that deuterated OT administered intranasally to rhesus macaques was quantified 2 h after administration in brain tissue collected from regions along the trajectory of the trigeminal and olfactory nerves, demonstrating that intranasal OT achieves brain penetration. Interestingly, IV administration of OT did not generate detectable brain concentrations of OT at this time point, consistent with OT’s rapid degradation in the bloodstream. This suggests that intranasal OT’s penetration of brain tissue might involve a mechanism that bypasses absorption into the blood.

Taken together, intranasal delivery of large molecules holds promise for rapid, targeted drug delivery, particularly to regions along the trajectory of the olfactory and trigeminal nerves as well as to the striatum. Drugs delivered via this route permeate the nasal epithelium and rapidly (~10 min) enter the olfactory and trigeminal systems, from which there is widespread reach to brain areas via convective bulk transport within the perivascular space of the cerebrovasculature [5]. Intranasal delivery of large molecular weight biologics (e.g., proteins, gene vectors, stem cells) results in the substantial and widespread distribution in brain tissue but with significantly less accumulation in CSF or blood.

The purpose of the present rodent study was to answer important open questions surrounding the brain penetrance of intranasal OT delivery that will inform the design and interpretation of clinical studies with intranasal OT. These questions are the extent of brain and body distribution of OT after intranasal delivery over time, the effect of OT dose on the latter, and the effect of mucoadhesive formulation on brain penetrance. Primarily, we aimed to quantify the concentration and distribution pattern of labeled OT throughout the CNS and several peripheral regions after a single dose of [^125^I]-labeled OT was delivered intranasally to anesthetized rats. Thirty minutes after the onset of delivery the concentration of OT in the CNS and peripheral tissues was quantified using gamma counting and the distribution pattern in the CNS was visualized using autoradiography. We also examined how this distribution pattern is altered by dose, mucoadhesive formulation, and time. To this end, we repeated the experiment with two additional doses of labeled OT, used a formulation of OT that contained a mucoadhesive, and studied brain penetrance with tissue collected at 60 min post intranasal OT administration. All major brain regions were analyzed, including those lying in the projection fields of the trigeminal and olfactory nerves.

## Materials and methods

### Animals

Adult male Sprague-Dawley rats (*N* = 61, 225–250 g) were used throughout the study. Sample sizes approximating ten subjects per group were chosen to provide reasonable estimations of standard deviation. It was not feasible to randomly assign rats in this study across the different groups given the order that experiments were completed. Due to the order of treatments and the preparation and/or visual appearance of the different solutions, it was also not feasible to blind the experimenter to the treatment condition. Animals were group-housed in the Regions Hospital Animal Care Facility with free access to food and water. Animals were kept on a 12 h light–dark cycle. All experimental procedures were approved by the Animal Care and Use Committee at Regions Hospital.

### Study 1: brain distribution and concentration measured with gamma counting and autoradiography

There were two experimental groups of animals: group I (*n* = 13) received [^125^I]-OT (0.4 mg) and was analyzed with gamma counting; group II (*n* = 8) received [^125^I]-OT (0.4 mg) and was analyzed with autoradiography.

#### Compounds and dosing formulations

OT was custom ^125^I-radiolabeled by GE Healthcare (Piscataway, NJ, USA). Radiolabeled OT was prepared in a buffer containing 10 mM sodium phosphate (pH 7.4), 2.7 mM potassium phosphate, 127 mM sodium chloride, and 3.7% acetonitrile, 0.015% TFA. Using the molecular weight of OT (1007.19 g/mol), the hot solution was calculated to contain 1985.72 µCi/µg at synthesis. Cold OT was purchased from Sigma Aldrich (St. Louis, MO, USA) and stored at −20 °C until ready for use. Using information provided by GE Healthcare along with our own measurements, the volume and concentration of hot and cold OT in each dose solution were calculated to yield a dose of 45 µCi and 0.4 mg OT in a volume of 48 µL.

#### [^125^I]-OT intranasal administration

The concentration of [^125^I]-OT in the CNS and body, and distribution pattern of [^125^I]-OT in the CNS was assessed 30 min following the initiation of intranasal delivery which was performed using a previously described method [6]. Rats were anesthetized using a cocktail of 3:3:1 ketamine:xylazine:acepromazine administered subcutaneously at a dose of 0.5 ml/kg. Animals were then placed on their backs on a heating pad in a metal surgical tray inside a fume hood. A lubricated rectal probe was inserted to monitor and maintain the core temperature of animals at 37 °C. Two 2 in ×2 in gauze pads were rolled into a pillow that was then placed under the rat’s head to maintain the position of the head so that the underside of the neck and mouth were horizontal. A cotton swab to be used for occlusion of the opposite naris was covered with parafilm. A lead impregnated shield was placed in front of the surgical tray to protect the experimenter from radiation. A series of three ×3 µL standards were aspirated from the microcentrifuge tube containing the dosing solution and expelled into pre-weighed and labeled gamma counting tubes. A 6 µL drop was loaded into the pipette behind the shield. The cotton swab was used to occlude one naris completely. The flat part of the swab was pushed gently against the naris at approximately 45° to prevent airflow. Holding the pipette vertically, the 6 µL drop was slowly expelled, forming a drop at the end of the tip. The drop was lowered onto the open naris to be snorted, and the cotton swab was removed from the opposite naris. After 2 min, the alternate naris was occluded and a 6 µL drop was administered in the same fashion to the open naris. This process was repeated every two min to alternating nares until a total of eight drops were delivered (four to each naris) for a total of 48 µL.

#### Brain dissection for gamma counting

Eight min prior to exsanguination, animals were further anesthetized with an additional half dose (50 mg/kg, IP) of sodium pentobarbital to ensure the surgical level of sedation and transcardially perfused with 60 ml of saline followed by 360 ml of 4% paraformaldehyde at a rate of 15 ml/min. A midline incision was made on the dorsal side of the skull. The skin was peeled back up to the eyes. The top of the brain was then exposed all the way to the olfactory bulbs, and dorsal dura was collected off the surface of the brain. The head was inverted, and the brain was removed. The ventral dura and the ophthalmic, mandibular, and maxillary branches of the trigeminal nerves as well as the trigeminal ganglia were collected from the skull cavity and placed into pre-weighed gamma tubes. The brain was placed dorsal side down in a coronal brain matrix. Razor blades were placed every 2 mm outward from the optic chiasm in order to generate slices from the midbrain to the tip of the forebrain, resulting in six slices. The olfactory bulbs were retrieved from the brain matrix and trimmed of any excess tissue. The upper cervical spinal cord was dissected and placed in the appropriate tube. The remaining brain tissue was bisected along the midline and the midbrain, pons, and medulla and scored straight down from the inferior colliculus, marking the midbrain. Another score was made under the point in the fourth ventricle, marking the medulla. The cerebellum was peeled off and placed in the corresponding tube. Following the previously made scores, the midbrain, pons, and medulla were dissected and placed into pre-weighed tubes.

The ventral side of the neck was then cut anteriorly and neck muscles exposed. The superficial and deep cervical nodes were dissected, cleared of connective tissues, and placed in pre-weighed gamma tubes. The thyroid was collected by gently peeling it from the trachea using forceps. A single-edge razor blade was then used to bisect the skull along the midline. The olfactory and respiratory epitheliums were collected from the cavity by scoring the edges and removing them using forceps. Care was taken to avoid the nasal septum.

#### Body dissection for gamma counting

The rat body was placed on its stomach and a superficial incision was made down the length of the animal from shoulders to hips, following the spine. The skin was peeled away from the underlying tissue on both sides to expose the shoulder blades. The axillary nodes in the connective tissue surrounding the shoulders were dissected, cleared of connective tissue, and placed in pre-weighed tubes. A 3 mm^2^ piece of right deltoid muscle was dissected and placed into a pre-weighed gamma tube. The rat was placed supine, and the sternum was cut vertically to further expose the abdominal and pleural cavities. In all, 3 mm^2^ square samples of the following tissues were dissected and placed into pre-weighed gamma counting tubes: liver, right superficial lobe; left kidney, tip; right lung, top lobe; spleen, tip; heart, apex. Urine was removed from the bladder using a 1cc syringe and transferred to a pre-weighed tube. A section of both the trachea and the esophagus was collected at the point of decapitation and placed in respective tubes. The body was placed prone and the entire remaining spinal cord was exposed, removed, and cut into lower cervical, thoracic, and lumbar portions. The dura was peeled from each section using forceps and a small spatula. All sections and the dura were then placed in respective pre-weighed tubes.

#### Brain dissection for autoradiography

The brain was removed from the skull as described above and the trigeminal ganglion with a portion of each of the three major branches was collected and arranged on microscope slides. The brain was retrieved and placed dorsal side down in a coronal brain matrix. A razor blade was inserted at the optic chiasm and the blade was marked with a permanent marker for later identification. Additional blades were placed every 1 mm outward from the optic chiasm. Slices were generated along the entire brain, from the front of the olfactory bulbs to the back of the cerebellum. The blades were removed one by one starting at the olfactory bulbs, inverted, and the tissue arranged on the microscope slides. The slices were labeled in reference to the optic chiasm, positive in the rostral direction and negative in the caudal direction. The remaining upper cervical spinal cord was retrieved, and 1-mm slices were cut. Superficial nodes, deep cervical nodes, thyroid, and olfactory and respiratory epitheliums were collected as described above.

#### Tissue counting

The pre-weighed gamma tubes containing samples were reweighed to determine tissue weight. Tissue samples were then counted using a COBRA II Auto-Gamma Counter (Packard Instrument Co., Meriden, CT, USA). The samples were counted under a standard ^125^I protocol with 5 min of count time. In the analysis of gamma counting results, any value outside two standard deviations of the mean for each tissue was considered an outlier and removed from the dataset.

#### Autoradiography

Samples were placed in a felt-lined box and an autoradiography screen (Super Sensitive; Packard Instrument Co.) was placed on top of the tissue samples. The box was closed and allowed to rest for 4 weeks. After 4 weeks, the screen was developed using the Cyclone Storage Phosphor System (Packard Instrument Co.).

#### Data analysis and calculations

Sample calculations for rat 1 were completed by hand and imported to Microsoft Excel^®^ (Seattle, WA, USA). All values were then auto-calculated using identical spreadsheets and double-checked for accuracy.

### Study 2: effect of OT dose on brain concentrations

Study 1 was repeated with 2 lower doses of OT (0.04 and 0.004 mg). There were two experimental groups of animals: group I (*n* = 10) received 0.04 mg OT and was analyzed with gamma counting, group II (*n* = 10) received 0.004 mg OT and was analyzed with gamma counting

### Study 3: the effect of a mucoadhesive administered with OT on delivery to the CNS

Study 1 was repeated with the addition of a mucoadhesive administered with [^125^I]-OT. There were two experimental groups of animals: group I (*n* = 10) received [^125^I]-OT (0.4 mg) with a mucoadhesive and was analyzed with gamma counting; group II (*n* = 8) received [^125^I]-OT (0.4 mg) with a mucoadhesive and was analyzed with autoradiography.

Avicel™ RC-591 mucoadhesive was obtained from FMC BioPolymer (Philadelphia, PA). The hygroscopic powder was stored at ambient temperature. At the advice of FMC staff, it was decided to use the mucoadhesive at a concentration of 1% by weight in the dosing solution. Dosing calculations for animals receiving mucoadhesive were adjusted accordingly to account for 1% mucoadhesive.

### Study 4: extended time interval (60 min) between administration of OT and brain sampling

One rat was treated with [^125^I]-OT, while the second rat received [^125^I]-OT with 1% Avicel™ mucoadhesive; both were sacrificed at 1 h after the onset of intranasal delivery.

## Results

### Study 1: brain distribution

#### Gamma counts

Gamma counts in the brain and body of rats are listed in Table 1 for animals that received intranasal administration of 0.4 mg[^125^I]-OT. These data indicate that following administration of the 0.4 mg OT dose, OT concentrations sufficient for OT-receptor activation were measured in all CNS tissues assayed, especially in the trigeminal system, including the dura, which is innervated by trigeminal nerve afferents.

**Table 1:** Gamma counts representing [125I] oxytocin (OT) concentration 30 min after 0.4 mg [125I] OT intranasal administration (A) without and (B) with Avicel™ mucoadhesive.

	A	B
Tissue description	Mean (±SD) [^125^I] OT concentration (nM) after IN OT (0.4 mg)	Mean (±SD) [^125^I] OT concentration (nM) after IN OT (0.4 mg) with 1% Avicel
Blood	61 (±4)	74 (±6)
Extra-olfactory nasal epithelium	680,567 (±85,021)	817642 (±41,406)
Olfactory epithelium	4112 (±694)	3153 (±432)
Trigeminal ganglion	311 (±79)	150 (±38)
Maxillary nerve	264 (±46)	340 (±50)
Mandibular nerve	291 (±38)	477 (±117)
Ophthalmic nerve	320 (±58)	278 (±51)
Olfactory bulbs	33 (±13)	60 (±15)
Anterior olfactory nucleus	24 (±5)	39 (±9)
Frontal cortex	21 (±4)	38 (±10)
Caudate/putamen	41 ± (10.8)	39 (±12.0)
Septal nucleus	28 (±6)	40 (±13)
Parietal cortex	28 (±7)	29 (±4)
Hippocampus	16 (±3)	21 (±5)
Thalamus	15 (±6)	10 (±1)
Hypothalamus	21 (±4)	30 (±4)
Midbrain	14 (±3)	17 (±4)
Pons	26 (±10)	27 (±6)
Medulla	25 (±9)	27 (±7)
Cerebellum	10 (±1)	16 (±2)
Dorsal dura	152 (±12)	395 (±78)
Ventral dura	271 (±43)	992 (±235)
Spinal dura	31 (±8)	15 (±2)
Upper cervical spinal cord	33 (±8)	50 (±15)
Lower cervical spinal cord	4 ± (0.3)	4 (±0.4)
Thoracic spinal cord	5 ± (0.5)	5 (±0.2)
Lumbar spinal cord	5 ± (0.6)	4 (±0.3)
Superficial nodes (2)	232 (±25)	365 (±44)
Cervical nodes (2)	213 (±36)	398 (±80)
Axillary nodes (2)	22 (±2)	19 (±2)
Thyroid	213 (±36)	739 (±110)
Muscle	14 (±1)	13 (±1)
Liver	13 (±1)	13 (±1)
Kidney	44 (±3)	53 (±6)
Lung	21 (±2)	21 (±2)
Trachea	26 (±3)	24 (±2)
Esophagus	25 (±2)	25 (±2)
Spleen	23 (±2)	22 (±2)
Heart	16 (±1)	14 (±1)
Urine	73 (±11)	72 (±12)
Drug standard	8,788,619 (±122,341)	9,160,899 (±78,596)
CPM/fmol	0.17 (±0.0)	0.17 (±0.0)

*CPM* counts per minute.

The concentration of [^125^I]-OT in the extra-olfactory nasal epithelium averaged 680,567 nM leading to effective delivery along the trigeminal nerve pathway (maxillary and ophthalmic branches) which innervate the lower nasal epithelium. The trigeminal ganglion contained an average concentration of 311 nM while the three major branches of the trigeminal nerve, the maxillary, mandibular, and ophthalmic, contained average concentrations of 264, 291, and 320 nM, respectively. The dorsal and ventral dura matter, which are heavily innervated by the trigeminal nerve, had concentrations of 152 and 271 nM, respectively. The average concentration of olfactory bulb samples was 33 nM.

The highest concentrations of [^125^I]-OT in the brain were observed in the caudate/putamen (41 nM), parietal cortex (28 nM), septal nucleus (28 nM), pons (26 nM), and medulla (25 nM). Trigeminal nerve entry into the brainstem may account for the high concentrations in the pons and medulla. Substantial concentrations of [^125^I]-OT were also found in the anterior olfactory nucleus (24 nM), frontal cortex (21 nM), hippocampus (16 nM), thalamus (15 nM), hypothalamus (21 nM), and midbrain (14 nM). The lowest concentration of [^125^I]-OT in the brain was found in the cerebellum at 10 nM. The dorsal and ventral dura/meninges contained high concentrations of [^125^I]-OT at 152 and 271 nM, respectively.

The average concentration of [^125^I]-OT in the upper cervical spinal cord was higher than anticipated at 33 nM. The remaining spinal cord segments, lower cervical, thoracic, and lumbar, contained average concentrations of 4, 5, and 5 nM respectively.

Thirty minutes after initiation of intranasal delivery, the concentration of radiolabel in the blood was 61 nM. As the half-life of OT in the blood is about 5 min, a significant portion of this radiolabel will represent degraded OT. Irrespective of the amount of degradation in the blood, the [^125^I]-OT concentration is at least five times higher in the trigeminal ganglion compared to the blood at this time.

#### Autoradiography

**Fig. 1:**
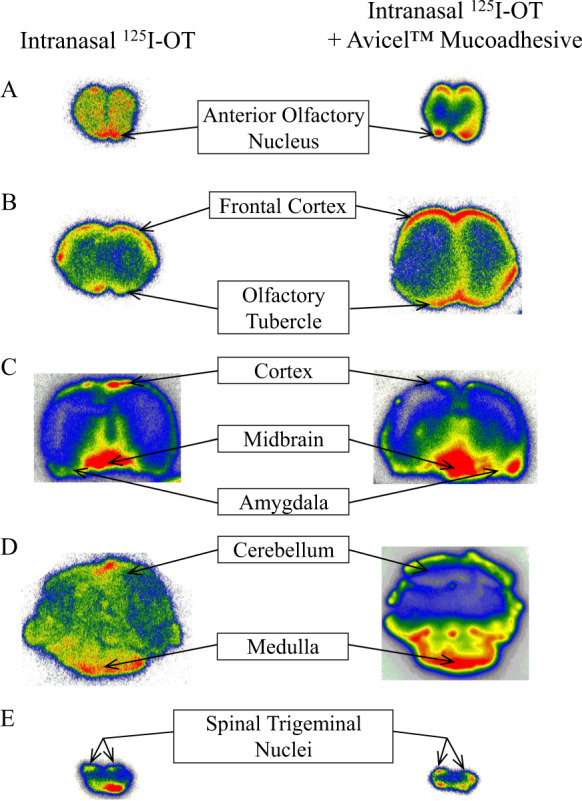
Qualitative distribution throughout brain tissue after intranasal ^125^I-oxytocin (OT) with or without the addition of 1% Avicel™ mucoadhesive. Autoradiograms of selected representative brain tissue slices illustrate the distribution of radiolabeled OT throughout the rat brain following intranasal administration with (left series of images) or without (right series of images) addition of 1% Avicel™ mucoadhesive in the formulation. Labels indicate for brain regions of interest the signal observed using the closest-available reference point among the representative slices. Full series of brain tissue slices for individual animals are presented in Supplemental Materials. The addition of Avicel™ mucoadhesive did not appreciably change the distribution of radiolabeled OT. High signal was detected in the anterior olfactory nucleus, olfactory tubercle, and cortex consistent with transport along the trajectory of the olfactory nerve; high signal in medulla/spinal trigeminal nuclei is consistent with transport along the trigeminal nerve. In addition, high signal was detected in the midbrain and some signals could be detected in amygdala regions. For intranasal OT without Avicel™ mucoadhesive (left series) representative slices = (**A**) rat 16, A/P + 6; (**B**) rat 12, A/P + 3; (**C**) rat 11, A/P −7; (**D**) rat 14, A/P −12; (**E**) Rat 13, A/P −19. For intranasal oxytocin with Avicel™ mucoadhesive (right series), representative slices = (**A**) rat 29, A/P + 7; (**B**) rat 29, A/P + 3; (**C**) rat 23, A/P −7; (**D**) Rat 28, A/P −13; (**E**) rat 29, A/P −18. See Supplemental Materials for details including full series of brain tissue slices for individual animals.

Qualitative autoradiography studies confirmed that intranasal [^125^I]-OT (0.4 mg) reached the CNS. The distribution of OT in autoradiography experiments was similar to that identified by gamma counting, with the greatest intensity of radiolabel in the trigeminal ganglion, olfactory bulbs, frontal cortex, midbrain, and medulla (Fig. 1).

### Study 2: effect of mucoadhesive

The addition of 1% Avicel™ mucoadhesive to the dosing solution did not markedly affect the brain tissue concentrations achieved at the 30 min sacrifice time (Table 1). Systemic tissue concentrations between drug formulations remained relatively unchanged with an identical concentration in the liver (13 nM), esophagus (25 nM), and lung (21 nM),). Despite this, the concentration of OT found in the thyroid was over three times higher with the addition of mucoadhesive to the dosing solution. The superficial and cervical nodes also exhibited a 1.5-fold increase with the addition of mucoadhesive.

Within the brain, the addition of mucoadhesive to the dosing solution caused an increase in the concentration of several rostral tissues including olfactory bulbs, anterior olfactory nucleus, frontal cortex, and septal nucleus. However, of these tissues, only the concentrations in the olfactory bulbs increased markedly. In addition, the concentration of OT in the trigeminal ganglion of animals receiving mucoadhesive was only half that of animals that did not receive mucoadhesive. Both groups contained concentrations high enough to feasibly activate OT receptors in all CNS tissues studied, especially in the trigeminal system. Animals receiving mucoadhesive had higher concentrations in the extra-olfactory epithelium but lower in the olfactory epithelium when compared to animals that did not receive mucoadhesive. This may be a result of the increased viscosity of the dosing solution containing mucoadhesive, which makes it more difficult to move all the way up past the turbinate to the olfactory epithelium at the roof of the nasal cavity. Mucoadhesive also increased OT concentrations in the dorsal and ventral dura at 30 min. These findings encourage studies with extended time points to investigate the advantage of mucoadhesive as a function of time. Qualitative autoradiography studies completed on animals receiving [^125^I]-OT with 1% Avicel ™ mucoadhesive resulted in a similar distribution to animals that received [^125^I]-OT without the mucoadhesive. The highest intensity was achieved in the trigeminal nerves, olfactory bulbs, and frontal cortex.

### Study 3: effect of dose

**Fig. 2:**
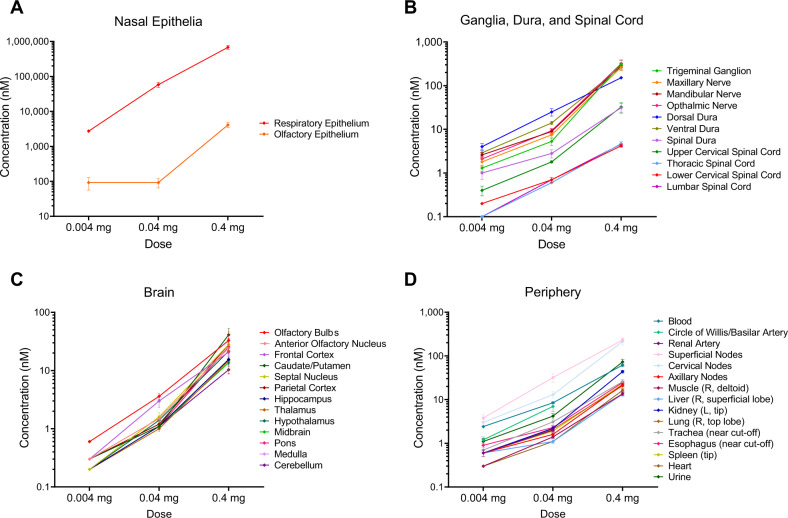
Quantification of ^125^I-oxytocin (OT) concentrations following intranasal administration, throughout central and peripheral tissues. **A** A dose-dependent signal was detected in both extra-olfactory and olfactory epithelia. **B** Dose-dependent concentrations of ^125^I-OT were detected throughout the dura, ganglia, and spinal cord. Concentrations were particularly high in the trigeminal ganglia and maxillary, mandibular, and ophthalmic nerve branches as well as the dorsal and ventral dura, both of which are also innervated by the trigeminal nerve. **C** Dose-dependent concentrations of [^125^I]-radiolabeled OT were detected throughout the brain regions assayed. At the 0.4 and 0.04 mg doses, the olfactory bulb and frontal cortex had markedly higher concentrations relative to most other brain regions. In this time course, i.e., 30 min post administration, this distribution is consistent with OT transport directly to the brain via an extraneuronal transport mechanism. High concentrations in the pons and medulla are consistent with transport along the trajectory of the trigeminal nerve. The high concentrations in the caudate/putamen (0.4 mg dose) as well as in the parietal cortex and septal nucleus may reflect regions of OT-receptor binding or accumulation along major CSF drainage pathways in the brain. **D** OT was detected in dose-dependent concentrations across peripheral tissues. The elevated concentrations in superficial and cervical nodes relative to blood and urine concentrations may reflect drainage of CSF into local tissue lymphatics and accumulation in local lymph tissue. See Table 1 and Supplemental Materials for details.

Table 2 reports mean concentrations in the brain and periphery after administration of 0.04 mg, and 0.004 mg of labeled OT, respectively (see Table 2 for comparison to the 0.4 mg dose). Brain penetrance with the 0.004 mg dose this dose was low, with all brain-region concentrations essentially equal, at 0.2 to 0.3 nM, too low a concentration to feasibly activate OT receptors. However, the trigeminal nerve and ganglia, which express functional OT receptors [20] had an adequate concentration for possible activation of OT receptors (1.3–2.6 nM) even at this dose as has the dorsal dura matter (4.0 nM). The 0.04 mg dose resulted in concentrations that were low that may still feasibly activate OT receptors. For visual comparison, concentrations in epithelia; brain, dura, and spinal cord; brain; and peripheral tissues were plotted across doses to evaluate the dose–response relationship of intranasal OT. As seen in Fig. 2 and Table 2, the concentration of OT across all tissues examined increases as the dose of intranasal OT increases.

**Table 2:** Gamma counts representing [125I] oxytocin (OT) concentration 30 min after: (A) 0.4 mg, (B) 0.04 mg, and (C)0.004 mg [I125] OT intranasal administration.

	A	B	C
Tissue description	Mean (±SD) [^125^I] OT concentration (nM) IN OT (0.4 mg)	Mean (±SD) [^125^I] OT concentration (nM) after IN OT (0.04 mg)	Mean (±SD) [^125^I] OT concentration (nM) after IN OT (0.004 mg)
Blood	61 (±4)	8.46 (±0.4)	2.42 (±0.2)
Extra-olfactory epithelium	680,567 (±85,021)	58,080 (±10,300)	2728 (±233)
Olfactory epithelium	4112 (±694)	92 (±27)	92 (±36)
Trigeminal Ganglion	311 (±79)	5.3 (±1)	1.3 (±0.2)
Maxillary nerve	264 (±46)	7.5 (±0.9)	1.8 (±0.4)
Mandibular nerve	291 (±38)	8.9 (±1.2)	2.6 (±0.3)
Ophthalmic nerve	320 (±58)	9.3 (±1.2)	2.1 (±0.3)
Olfactory bulbs	33 (±13)	3.6 (±0.4)	0.6 (±0.0)
Anterior olfactory nucleus	24 (±5)	1.5 (±0.1)	0.3 (±0.0)
Frontal cortex	21 (±4)	3.0 (±0.7)	0.3 (±0.0)
Caudate/putamen	41 ± (10.8)	1.1 (±0.1)	0.3 (±0.0)
Septal nucleus	28 (±6)	1.6 (±0.3)	0.2 (±0.0)
Parietal cortex	28 (±7)	1.2 (±0.1)	0.3 (±0.0)
Hippocampus	16 (±3)	1.1 (±0.1)	0.2 (±0.0)
Thalamus	15 (±6)	1.0 (±0.1)	0.2 (±0.0)
Hypothalamus	21 (±4)	1.4 (±0.2)	0.2 (±0.0)
Midbrain	14 (±3)	1.1 (±0.1)	0.2 (±0.0)
Pons	26 (±10)	1.1 (±0.1)	0.2 (±0.0)
Medulla	25 (±9)	1.1 (±0.1)	0.3 (±0.0)
Cerebellum	10 (±1)	1.1 (±0.0)	0.3 (±0.0)
Dorsal dura	152 (±12)	25 (±4.8)	4.0 (±0.7)
Ventral dura	271 (±43)	14 (±1.3)	2.9 (±0.6)
Spinal dura	31 (±8)	2.8 (±0.6)	1.0 (±0.3)
Upper cervical spinal cord	33 (±8)	1.8 (±0.1)	0.4 (±0.1)
Lower cervical spinal cord	4 ± (0.3)	0.7 (±0.1)	0.2 (±0.0)
Thoracic spinal cord	5 ± (0.5)	0.6 (±0.0)	0.1 (±0.0)
Lumbar spinal cord	5 ± (0.6)	0.7 (±0.0)	0.1 (±0.0)
Circle of Willis/basilar artery	Not tested	7 (±1.7)	1.2 (±0.2)
Renal artery	Not tested	2.0 (±0.2)	0.6 (±0.1)
Superficial nodes (2)	232 (±25)	32 (±7)	3.8 (±0.7)
Cervical nodes (2)	213 (±36)	13 (±2.2)	3.0 (±0.5)
Axillary nodes (2)	22 (±2)	1.6 (±0.1)	0.6 (±0.1)
Thyroid	213 (±36)	80 (±10)	29 (±5)
Muscle	14 (±1)	1.4 (±0.1)	0.3 (±0.0)
Liver	13 (±1)	1.1 (±0.1)	0.6 (±0.1)
Kidney	44 (±3)	2.2 (±0.3)	0.6 (±0.1)
Lung	21 (±2)	2.1 (±0.2)	0.6 (±0.1)
Trachea	26 (±3)	3.1 (±0.5)	0.7 (±0.1)
Esophagus	25 (±2)	2.3 (±0.2)	0.9 (±0.1)
Spleen	23 (±2)	1.9 (±0.2)	0.6 (±0.1)
Heart	16 (±1)	1.1 (±0.1)	0.3 (±0.0)
Urine	73 (±11)	4.2 (±0.6)	1.1 (±0.2)
CPM/fmol	0.17 (±0.0)	1.4 (±0.0)	12.7 (±0.1)

*CPM* counts per minute.

### Study 4: effect of time

Both animals at 60 min post 0.4 mg/kg IN OT delivery had high enough concentrations to feasibly activate OT receptors in all brain tissues. In the trigeminal system, concentrations were especially high. Table 3 demonstrates that in the majority of brain regions (with the exception of the frontal cortex), the animal receiving mucoadhesive had lower brain tissue concentrations than the animal receiving only [^125^I]-OT. In addition, the blood and majority of the peripheral tissues also had lower concentrations of OT in the animal receiving the mucoadhesive (Table 3). The animal receiving the mucoadhesive had higher concentrations of OT in the trigeminal ganglion, maxillary nerve, mandibular nerve, and ophthalmic nerve. This data is inconsistent with the 30 min data suggesting that mucoadhesive might decrease the concentration of OT reaching the trigeminal system over time. Larger sample size at later time points is necessary before completely ruling out the potential of the present mucoadhesive formulation.

**Table 3:** Gamma Counts representing [125I]oxytocin (OT) concentration 60 min after intranasal administration of [125I]OT: (A) 0.4 mg and (B) 0.4 mg with Avicel®™ mucoadhesive (one rat per condition; CPM = counts per minute).

	A	B
Tissue description	[^125^I] OT concentration (nM) after IN OT (0.4 mg)	[^125^I] OT concentration (nM) after IN OT (0.4 mg) + Avicel™
Blood	155	103
Extra-olfactory epithelium	1,075,273	1,078,689
Olfactory epithelium	100,193	9839
Trigeminal ganglion	301	1106
Maxillary nerve	163	538
Mandibular nerve	201	346
Ophthalmic nerve	209	462
Olfactory bulbs	80	17
Anterior olfactory nucleus	42	12
Frontal cortex	25	66
Caudate/putamen	31	24
Septal nucleus	24	15
Parietal cortex	14	12
Hippocampus	16	13
Thalamus	11	7
Hypothalamus	26	23
Midbrain	20	12
Pons	42	16
Medulla	37	12
Cerebellum	27	14
Dorsal dura	364	232
Ventral dura	565	7168
Spinal dura	26	10
Upper cervical spinal cord	18	26
Lower cervical spinal cord	10	5
Thoracic spinal cord	11	7
Lumbar spinal cord	18	6
Superficial nodes (2)	109	192
Cervical nodes (2)	381	446
Axillary nodes (2)	51	30
Thyroid	2882	3925
Muscle	36	28
Liver	22	39
Kidney	166	56
Lung	56	22
Trachea	105	79
Esophagus	59	126
Spleen	68	48
Heart	35	30
Urine	676	103
Drug standard	3,995,562	4,029,771
CPM/fmol	0.17	0.16

## Discussion

This study examined the brain distribution and dose–response of [^125^I]-radiolabeled OT after its intranasal administration to male Sprague-Dawley rats. This was done using quantitative gamma counts in the brain and body, and qualitative assessment of distribution patterns in the CNS using autoradiography 30 and 60 min following nasal [^125^I]-OT administration. Despite an abundance of preclinical and clinical data pointing toward the potential of intranasal OT as a treatment for craniofacial pain as well as a wide range of CNS disorders, there remains little evidence that intranasal OT enters the brain. The tendency in previous studies to use CSF OT concentrations as a proxy measure for brain tissue penetrance likely contributes to this issue. In addition, little is known about the brain concentrations of administered OT as a function of dose or time and if the addition of a mucoadhesive improves brain penetrance.

Here, we provide direct evidence that intranasal [^125^I]-OT concentrates in peripheral trigeminal and olfactory nerves as well as several brain regions such as the striatum, one brain region where OT exerts its prosocial effect [21]. We report also preliminary data on the utility of mucoadhesive formulation and the effect of time on brain concentration of the administered peptide.

After nasal application of [^125^I]-OT, very high concentrations of OT were found in all three branches of the trigeminal nerve and the trigeminal ganglion, and to a lesser, but significant extent in the olfactory system, providing further support for the importance of these cranial nerve pathways for delivering large molecules to the brain [5]. Significantly, all three branches of the trigeminal nerve had high levels of [^125^I]-OT, despite only two branches innervating the nose. High levels were also measured in the dorsal and ventral dura matter, where heavy innervation by the trigeminal nerve afferent fibers plays a critical role in migraine headache [22]. These findings are important in that they demonstrate that at least some (likely most) of the OT distribution along nerve fibers following nasal administration is *via* extracellular rather than intracellular transport mechanisms (i.e., transport is not restricted to directly innervated pathways).

Relatively high levels of [^125^I]-OT were also seen in the olfactory system, providing further support for the importance of both the trigeminal and olfactory nerve pathways for delivering large molecules to the brain [5]. Significant quantities of [^125^I]-OT were found in the frontal and parietal cortex, and subcortical structures, including the caudate–putamen, thalamus, septal nucleus, hypothalamus, and hippocampus. Of note, a recent study by Lee et al. [19] in nonhuman primates also found labeled OT after intranasal administration in brain regions along the trajectory of the trigeminal and olfactory nerves; like the present study, they found a robust accumulation of administered OT in the striatum. All of these brain regions have been implicated in the modulatory effects of OT on social behavior, memory, and reward processing [23]. In rodents and humans, the OT receptor is expressed in other deep-brain structures critical for the regulation of social and emotional behavior. This includes, for example, several amygdala subdivisions where OT has been implicated in the modulation of social interaction, fear and stress responses, and anxiety-like behavior [24]. The present data demonstrate intranasal OT can reach deep-brain structures such as the amygdala in sufficient concentrations to allow OT-receptor activation. This is important given that OT’s putative therapeutic efficacy is proposed to involve remediation of dysregulated OT signaling in amygdala subregions, as exists in autism spectrum and alcohol use disorders [24, 25].

A significant concentration of [^125^I]-OT was also found in the olfactory bulb, suggesting entry to the brain via olfactory nerve afferents located within close proximity to many of the subcortical brain regions shown here to contain high concentrations of [^125^I]-OT. These concentrations may have functional relevance as the olfactory bulb is densely populated by OT receptors and is considered important for detection of odors and encoding of social memory, recently demonstrated to be stimulated by systemic OT administration as measured by functional imaging in awake rodents [26, 27].

The presence of [^125^I]-OT in the pons, medulla, and cerebellum suggests hindbrain access via the trigeminal nerve. Importantly, OT has been shown to impact a variety of autonomic functions via OT receptors distributed in the hindbrain (e.g., appetite) [28]. Further, we found very high concentrations of [^125^I]-OT in the dorsal and ventral dura and significant concentrations in the spinal dura and cervical, but not the thoracic or lumbar spinal cord. In addition, our finding of specific autoradiographic labeling of [^125^I]-OT in the spinal trigeminal nucleus dorsal horn is consistent with the potential contribution of OT in pain modulation in the CNS [29]. OT may also modulate pain by acting directly on peripheral nociceptive neurons [20, 30, 31]. Consistent with this concept, we recently reported that OT receptors are co-localized with calcitonin gene-related peptide (CGRP), a neuropeptide selectively expressed in nociceptive neurons, in trigeminal ganglion neurons [32]. Further, OT receptors were upregulated on these neurons following inflammatory and/or noxious stimulation. Importantly, the application of OT to dural tissue can block the evoked release of CGRP, suggesting a potential for OT as a treatment for migraine and other chronic pain disorders involving the trigeminal system.

Given the potential for OT to be developed as a treatment for a wide range of neuropsychiatric disorders and given that BBB penetrance of OT is a major hurdle to targeting central OT receptors, there is great interest in developing formulations of OT that can enhance brain penetrance by bypassing the BBB with intranasal delivery [33]. Several factors contribute to the effectiveness of intranasal delivery of large molecules to the brain including the targeting of the sprayed formulation to the olfactory epithelium, the metabolism and clearance of the drug in the nasal mucosa, the activity of transporters in the case of intracellular transport, and nasal epithelium permeability in the case of extracellular transport [34]. The effectiveness of nose-to-brain delivery of large molecules depends heavily on the physicochemical properties of the drug and the mechanism of its transport [34]. The CNS distribution and time course of OT appearance in regions that fall along olfactory and trigeminal pathways are consistent with the notion that OT can be transported to the brain via an extracellular transport mechanism, moving in the CSF that flows within the perivascular spaces of blood vessels associated with the olfactory and trigeminal nerve bundles [7, 35].

It is important to clarify here that travel along nerve bundles is likely not the only mechanism by which intranasally applied OT enters the brain (for discussion of this issue, see ref. [36]. Emerging evidence suggests that vascular endothelium RAGE transporters play a role in allowing OT transport across the blood–brain barrier. Intranasal OT application in RAGE-transporter-null mice contrasted with wild-type mice resulted in blunted brain OT concentrations detected in perfusates collected from the medial prefrontal cortex (60-min timepoint) and periventricular nucleus of the hypothalamus (60-min timepoint; [37], and the amygdala (90-min timepoint; [38]. It is likely that OT’s absorption into the bloodstream through the rich vasculature of the nasal epithelia and subsequent transfer across endothelial RAGE transporters in cerebral microvasculature is critical to OT’s penetration of deep-brain structures. While transport along nerve bundles appears to bypass the blood–brain barrier, it remains to be determined whether the RAGE transporter is also critical for this pathway (i.e., if OT may travel in blood vessels along these pathways). In any case, it is likely that transfer along these different routes is differently important for different brain regions and at different times. For example, in a recent human study comparing intranasal spray, intranasal nebulizer, and intravenous administration of OT on regional cerebral blood flow, it was observed that amygdala perfusion correlated with peripheral OT concentration following administration by each route. However, other changes in regional cerebral blood flow could not be accounted for by blood levels of OT [39]. The results suggest the involvement of transport (or direct neuronal action) in olfactory/trigeminal neuronal bundles, however, even including these mechanisms, the full range of effects observed could not easily be explained (for discussion, see ref. [39]. The high concentrations of oxytocin observed along the trigeminal nerve and pons, medulla, and cerebellum in the present study appear to also suggest a route along the trigeminal nerve that may bypass the blood–brain barrier. Separately, high concentrations in the olfactory bulb, the dura matter, superficial cortical layers, and additional concentrations observed in the caudate/putamen, parietal cortex, and septal nucleus may reflect OT accumulation along CSF bulk flow and/or drainage pathways that suggests intranasal OT’s unique pattern of distribution may also involve absorption from nasal epithelia directly to the CSF. It also appears likely that concentration of OT in deeper brain structures that do not sit directly along trigeminal or olfactory pathways (e.g., the amygdala) necessitate a mechanism for transport across the blood–brain barrier (i.e., via the RAGE transporter, [38]. Supporting this possibility, significant OT concentrations were indeed observed in blood measures in the present dataset.

Enhancing residence time in the nasal mucosa may enhance intranasal drug delivery along these pathways. Avicel™ RC 591 is commonly used in suspending agents for aqueous compositions, a formulation of common pharmaceutical stabilizing and suspending agents microcrystalline cellulose and sodium carboxymethyl cellulose [40]. This study tested the ability of Avicel™ RC 591 as a prototypical mucoadhesive formulation to increase the residency time of OT in the nasal mucosa to allow for greater quantities to pass along the trigeminal and olfactory nerve pathways and thereby reach higher concentrations in brain tissue. While an increase in concentration in the extra-olfactory epithelium was observed at 30 min (no longer at 60 min), there was a reduction in detected OT in the olfactory epithelium at 30 min and 60 min indicating a failure of the mucoadhesive formulation to enhance residence time in the upper reaches of the nasal cavity, perhaps due to the increased viscosity of the mucoadhesive. Regardless of the mechanism, overall, the addition of this mucoadhesive did not appreciably increase brain concentrations of OT.

There are several limitations that pose considerable hurdles to translating the present rat data toward inferences about the putative nose-to-brain transfer and distribution of OT in humans. First, there are clear differences in the structure and accessibility of the rodent vs. human nasal cavities, as well as differences in the size of the rat vs. human brain and relative size of the rat vs. human nasal cavity in terms of surface area and innervation, that likely improve the effectiveness of nose-to-brain transfer in rats compared to humans. Notably, the olfactory epithelium that is the proposed target for a nose-to-brain transfer accounts for ~45–50% of the rodent nasal cavity. In contrast, the olfactory epithelium makes up ~5% of the nonhuman primate nasal cavity and just 3% of the nasal cavity in humans [41]. Second, the present detection method does not guarantee the percentage of detected radiolabel that remains attached to the OT peptide. This is a strength of previous work using mass spectrometry to confirm deuterated OT is recovered in brain tissue of nonhuman primates (see convergent data provided by Lee et al. [19]). Third, this study was completed in male rats, and it is unclear whether sex differences in the distribution of intranasally applied OT (or any compound) may exist and could contribute to putative sex differences in the response to intranasally applied compounds. Nevertheless, these limitations do not alter the conclusion regarding the mucoadhesive formulation that did not appreciably enhance the ability of OT to enter brain tissue. This finding highlights the difficulty of developing enhanced nose-to-brain delivery treatments and is important given the considerable efforts to date with mucoadhesive formulation and the attendant regulatory burden of developing new formulations.

Although OT has a short plasma half-life [42] and limited BBB penetrability [43], there is evidence for a therapeutic benefit of systemic OT for CNS disorders outlined in both clinical research (e.g., autism) [44, 45] and a wide range of preclinical studies [25, 46–48]. Thus, it remains possible that the apparent efficacy of nasal OT in the CNS is mediated through its actions in the periphery [49]. Conversely, our results demonstrate an alternative pathway, along the trigeminal and olfactory neural pathways, to central nervous system sites where these effects might be mediated. The dose–response data reported here (Table 2 and Fig. 2 indicate that the 0.04 mg dose of OT results in significant regional elevation of brain OT concentrations. This is consistent with Lee et al. [19], who found similar concentrations could be achieved with intranasal administration of labeled OT to nonhuman primates.

In addition to the experiments described above, one animal was available to test at 60 min after initiating intranasal administration of [^125^I]-OT (Table 3). This animal had similar [^125^I]-OT concentrations and distribution patterns in the brain and body to the animals tested 30 min after initiating intranasal administration of [^125^I]-OT. Lee et al. [19], also found relevant concentrations of labeled OT in brain regions 2 h after intranasal administration. Taken together, this suggests that the half-life of OT in the brain may be longer than originally thought [16].

In conclusion, the results described above provide the first direct evidence for uptake of intranasally delivered [^125^I]-OT to the CNS along the trigeminal and olfactory neural pathways. These data provide information that is important with respect to designing clinical studies with intranasal OT, especially with respect to timing the delivery of intranasal OT, dosage employed, formulation, and brain distribution achieved. As the experimental use of nasal OT in clinical populations increases, the results provide further impetus to determine the pharmacokinetics of OT in humans after nasal application.

## Supplementary information

SUPPLEMENT
