# Supplementary material for: Nasal oxytocin for the treatment of psychiatric disorders and pain: achieving meaningful brain concentrations
Source: Transl Psychiatry. 2021 Jul 10;11:388. doi: 10.1038/s41398-021-01511-7 (PMC8272715; doi:10.1038/s41398-021-01511-7)

SUPPLEMENT

Pre-Clinical Pharmacokinetic Distribution Study in Rats for Intranasal Administration of Oxytocin

PART 1

Executive Summary

- Intranasal oxytocin rapidly reached the central nervous system with very significant concentrations in the trigeminal nerve branches and trigeminal ganglion.

- High concentrations in olfactory and trigeminal structures confirmed delivery along these pathways. The trigeminal ganglion contained an average concentration of 311 nM while the three major branches, maxillary, mandibular, and ophthalmic, contained average concentrations of 264 nM, 291 nM, and 320 nM respectively. The average concentration of olfactory bulb samples was 33 nM.

- The highest delivery of oxytocin in the brain was observed in the caudate/putamen (41 nM), parietal cortex (28 nM), septal nucleus (28 nM), pons (26 nM), and medulla (25 nM).

- Concentrations achieved in the CNS after a single intranasal treatment with 0.4 mg (0.3 umol) oxytocin were high enough to activate high affinity oxytocin receptors in all CNS tissues and both high and low affinity receptors in the trigeminal system and meninges covering the brain.

- Addition of 1% Avicel mucoadhesive to the oxytocin solution did not markedly affect CNS concentrations achieved with intranasal administration at 30 min. Avicel did increase delivery to the meninges and cervical lymph nodes, but delivery to the trigeminal ganglion was reduced by 50% when Avicel was included in the formulation.

- The distribution of oxytocin in autoradiography experiments was similar to that identified by gamma counting, with the greatest intensity in the trigeminal ganglion, olfactory bulbs, septal nucleus, and frontal cortex.

♦ Table of Contents

Executive Summary 2

Table of Contents 3-5

Purpose of Study 6

Compounds and Dosing Calculations 6-8

General Procedures 8-15

Formulation of Dose Solution 8-9
Animals 9
Anesthesia 9
Intranasal Delivery of ^{125}I-Oxytocin and ^{125}I-Oxytocin with 1% Mucoadhesive 10
Transcardial Perfusion 10-11
Brain Dissection 11-13
Body Dissection 13-14
Animal Details 14
Tissue Counting 15
Autoradiography 15
Data Analysis and Calculations 15

Results 16-25

Table 01: Intranasal Delivery of Oxytocin Data Summary 19
Table 02: Intranasal Delivery of Oxytocin with 1% Mucoadhesive Data Summary 20
Table 03: Intranasal Oxytocin vs. Intranasal Oxytocin with 1% Mucoadhesive 21
Table 04: Extended Time point: Intranasal Oxytocin vs. Intranasal Oxytocin with 1% Mucoadhesive 22
Figure 01: Qualitative Distribution after Intranasal Delivery of Oxytocin 23
Figure 02: Qualitative Distribution after Intranasal Delivery of Oxytocin with 1% Mucoadhesive 24
Figure 03: Qualitative Distribution of Oxytocin in Trigeminal Ganglion 25

Appendices

Appendix A – Intranasal Oxytocin Data 26-47

Table A01: Intranasal Oxytocin Rat 1 Data 27
Table A02: Intranasal Oxytocin Rat 2 Data 28
Table A03: Intranasal Oxytocin Rat 3 Data 29
Table A04: Intranasal Oxytocin Rat 4 Data 30
Table A05: Intranasal Oxytocin Rat 5 Data 31
Table A06: Intranasal Oxytocin Rat 6 Data 32

Table A07: Intranasal Oxytocin Rat 7 Data 33
Table A08: Intranasal Oxytocin Rat 8 Data 34
Table A09: Intranasal Oxytocin Rat 9 Data 35
Table A10: Intranasal Oxytocin Rat 10 Data 36
Table A11: Intranasal Oxytocin Rat 11 Data 37
Table A12: Intranasal Oxytocin Rat 12 Data 38
Table A13: Intranasal Oxytocin Rat 13 Data 39
Table A14: Intranasal Oxytocin Rat 14 Data 40
Table A15: Intranasal Oxytocin Rat 15 Data 41
Table A16: Intranasal Oxytocin Rat 16 Data 42
Table A17: Intranasal Oxytocin Rat 17 Data 43
Table A18: Intranasal Oxytocin Rat 18 Data 44
Table A19: Intranasal Oxytocin Rat 19 Data 45
Table A20: Intranasal Oxytocin Rat 40 Data 46
Table A21: Intranasal Oxytocin Rat 41 Data 47

Appendix B – Intranasal Oxytocin with 1% Avicel ® Mucoadhesive Data 48-66

Table B01: Intranasal Oxytocin with Mucoadhesive, Rat 20 Data 49
Table B02: Intranasal Oxytocin with Mucoadhesive, Rat 21 Data 50
Table B03: Intranasal Oxytocin with Mucoadhesive, Rat 22 Data 51
Table B04: Intranasal Oxytocin with Mucoadhesive, Rat 23 Data 52
Table B05: Intranasal Oxytocin with Mucoadhesive, Rat 24 Data 53
Table B06: Intranasal Oxytocin with Mucoadhesive, Rat 25 Data 54
Table B07: Intranasal Oxytocin with Mucoadhesive, Rat 26 Data 55
Table B08: Intranasal Oxytocin with Mucoadhesive, Rat 27 Data 56
Table B09: Intranasal Oxytocin with Mucoadhesive, Rat 28 Data 57
Table B10: Intranasal Oxytocin with Mucoadhesive, Rat 29 Data 58
Table B11: Intranasal Oxytocin with Mucoadhesive, Rat 32 Data 59
Table B12: Intranasal Oxytocin with Mucoadhesive, Rat 33 Data 60
Table B13: Intranasal Oxytocin with Mucoadhesive, Rat 34 Data 61
Table B14: Intranasal Oxytocin with Mucoadhesive, Rat 35 Data 62
Table B15: Intranasal Oxytocin with Mucoadhesive, Rat 36 Data 63
Table B16: Intranasal Oxytocin with Mucoadhesive, Rat 37 Data 64
Table B17: Intranasal Oxytocin with Mucoadhesive, Rat 38 Data 65
Table B18: Intranasal Oxytocin with Mucoadhesive, Rat 39 Data 66

Appendix C – Intranasal Oxytocin Extended Time point (1 h) 67-69

Table C01: Intranasal Oxytocin Rat 30 (1 h sac) Data 68
Table C02: Intranasal Oxytocin with Mucoadhesive, Rat 31 (1 h sac) Data 69

Appendix D – Autoradiography Images 70-86

Figure D01: Rat 10 Autoradiography Images 71
Figure D02: Rat 11 Autoradiography Images 72

Figure D03: Rat 12 Autoradiography Images 73
Figure D04: Rat 13 Autoradiography Images 74
Figure D05: Rat 14 Autoradiography Images 75
Figure D06: Rat 15 Autoradiography Images 76
Figure D07: Rat 16 Autoradiography Images 77
Figure D08: Rat 17 Autoradiography Images 78
Figure D09: Rat 22 Autoradiography Images 79
Figure D10: Rat 23 Autoradiography Images 80
Figure D11: Rat 28 Autoradiography Images 81
Figure D12: Rat 29 Autoradiography Images 82
Figure D13: Rat 34 Autoradiography Images 83
Figure D14: Rat 35 Autoradiography Images 84
Figure D15: Rat 36 Autoradiography Images 85
Figure D16: Rat 37 Autoradiography Images 86

Appendix E – Coronal Rat Brain Slices 87-94

Figure E01: Brain Slice 01 88
Figure E02: Brain Slice 02 88
Figure E03: Brain Slice 03 89
Figure E04: Brain Slice 04 89
Figure E05: Brain Slice 05 90
Figure E06: Brain Slice 06 90
Figure E07: Lateral Brain Bisection 91

Purpose of Study

The purpose of this study was to quantify the concentration and distribution of oxytocin in the central nervous system (CNS) and peripheral tissues after intranasal delivery to anesthetized rats. We examined the distribution of oxytocin at 30 min after the onset of intranasal delivery. In addition, the effect of a mucoadhesive (Avicel ®) on delivery to the CNS was examined. In all cases, a combination of ^{125}I-labeled oxytocin and unlabeled oxytocin was delivered. At 30 min after the onset of delivery, each animal was perfused with saline while under anesthesia followed by 4% paraformaldehyde and tissues dissected. The concentration of oxytocin in the tissues was quantified using gamma counting while the distribution was visualized using autoradiography. The delivery results of both oxytocin formulations were then compared.

Compounds and Dosing Calculations

Oxytocin was custom ^{125}I-radiolabeled by GE Healthcare in three batches (10/3/07, IMQ.7836v, Batch 0740; 10/23/07, IMQ.7836v, Batch 0743; 11/13/07, IMQ.7836v, Batch 0746) with initial concentrations of 2.30 mCi/ml, 2.32 mCi/ml, and 2.32 mCi/ml respectively. All batches had a specific activity of 2000 Ci/mmol. Radiolabeled oxytocin was prepared in a buffer containing 10 mM sodium phosphate (pH 7.4), 2.7 mM potassium phosphate, 127 mM sodium chloride, and 3.7% acetonitrile, 0.015% TFA. Using the molecular weight of oxytocin (1007.19 g/mol) the hot solution was calculated to contain 1985.72 uCi/ug at synthesis.

Cold oxytocin was purchased from Sigma Aldrich (BioChemika, acetate salt, ≥ 97%), product number 75968, lot 1322440. The product was stored at -20°C until ready for use.

Avicel RC-591® mucoadhesive was obtained from FMC BioPolymer (Philadelphia, PA). The hygroscopic powder was stored at ambient temperature. At the advice of FMC staff, it was decided to use the mucoadhesive at a concentration of 1% by weight in the dosing solution. Dosing calculations for animals receiving mucoadhesive were adjusted accordingly to account for 1% mucoadhesive.

The desired volume for intranasal delivery to anesthetized rats was 48 ul. The total prepared solution contained ~48 ul for dosing, 9 ul for standards, and 5 ul for evaporation totaling 62 ul. Using information provided by GE Healthcare, the volume and concentration of hot and cold oxytocin in each dose solution was calculated with a desired dose of 45 uCi and 0.4 mg oxytocin.

Sample Calculations: ^{125}I-Oxytocin: Batch I, Rat 1

To reach the desired 0.4 mg oxytocin while maintaining a 48 ul dose solution, the cold oxytocin solution (for use with non-mucoadhesive animals) was prepared at 15 mg/ml.

Volume hot to deliver=desired uCi/ (uCi/ul at synthesis)/ (decay rate of ^{125}I*(date of delivery - date of synthesis))

Delivery date 10/08/07:
Volume hot to deliver=45 uCi/2.30 uCi/ul/ (e^ (-0.01155*5 days) =20.73 ul

Volume cold to deliver=desired mg-(ul hot*(uCi/ul at synthesis)/ (uCi/ug at synthesis)/1000) / (cold ug/ul)*1000

Delivery date 4/24/07:
Volume cold to deliver= (0.4 mg-(20.73 uL*2.3 uCi/ul /1,985.7 uCi/ug /1000))/15 ug/ul *1000 =26.67 ul

Total volume to deliver= 20.73 + 26.67 = 47.4 ul

Volume hot to make dose solution=total ul desired/total ul in dose solution*ul hot in dose solution

Delivery date 10/08/07:
Volume hot to make dose solution=61.39 ul/47.4 ul *20.73 ul=26.85 ul

Volume cold to make dose solution=total ul desired/total ul in dose solution*ul cold in dose solution.

Delivery date 10/08/07:
Volume cold to make dose solution: 61.39 ul/47.4 ul*26.67 ul=34.54 ul

Total volume to make dose solution (dose, standards, evaporation) = 26.85+34.54=61.39 ul

Sample Calculations: ^{125}I-Oxytocin with 1% Avicel®: Batch III, Rat 31

In an effort to minimize differences between dosing solutions with and without mucoadhesive, all solutions were aliquoted in the absence of mucoadhesive and frozen until ready for use. To account for the additional volume to be added on the day of delivery, dosing solutions that were to contain mucoadhesive were initially aliquoted at a higher concentration. Upon addition of mucoadhesive on the day of delivery, the dosing solution was diluted to the appropriate 0.4 mg oxytocin in ~48 ul. The cold oxytocin solution for use with mucoadhesive animals was prepared at 69.6 mg/ml.

Volume hot to deliver=desired uCi/ (uCi/ul at synthesis)/ (decay rate of ^{125}I*(date of delivery - date of synthesis))

Delivery date 11/15/07:
Volume hot to deliver=45 uCi/2.32 uCi/ul/ (e^ (-0.01155*2 days) =19.85 ul

Volume cold to deliver=desired mg-(ul hot*(uCi/ul at synthesis)/ (uCi/ug at synthesis)/1000) / (cold ug/ul)*1000

Delivery date 11/15/07:
Volume cold to deliver= (0.4 mg-(19.85 uL*2.32 uCi/ul /1,985.7 uCi/ug /1000))/15 ug/ul *1000 =26.67 ul

> Of the 26.67 ul cold to deliver, 5.75 ul will be 69.6 mg/ml oxytocin while 20.92 ul will be 2.29% Avicel.

Total volume to deliver= 19.85 + 26.67 = 46.52 ul

Volume hot to make dose solution=total ul desired/total ul in dose solution*ul hot in dose solution

Delivery date 11/15/07:
Volume hot to make dose solution=60.51 ul/46.51 ul *19.85 ul=25.82 ul

Volume cold to make dose solution=total ul desired/total ul in dose solution*ul cold in dose solution.

Delivery date 11/15/07:
Volume cold to make dose solution: 60.51 ul/46.51 ul *26.67 ul=34.69 ul

> Volume of cold that is 69.6 mg/ml oxytocin: (vol 69.6 mg/ml oxytocin to deliver)/ (total volume cold to deliver) * (total volume cold to make dose solution) = 5.75 ul/26.67 ul*34.69 ul = 7.47
>
> Volume of 2.29% Avicel to make dose solution: (total volume cold to make dose solution - vol. 69.6 mg/ml oxytocin) = 34.69 ul – 7.47 ul = 27.22 ul 2.29% Avicel

Total volume to make dose solution (dose, standards, evaporation) = 25.82+7.47+27.22=60.51 ul

General Procedures

Throughout experimental procedures, strict precautions were followed to prevent radiation exposure and contamination of animal tissues, surgical tools, and equipment. Geiger counters were placed at each work station to continuously screen tools, workspace, and staff. Personal protective equipment including double layered gloves, lab coats, eye protection, masks, and bouffant caps was worn at all times. Lead impregnated shields were used to minimize exposure to radiation. Radioactive monitoring badges were also worn by staff throughout experimental procedures to quantify exposure.

Formulation of Dose Solution

To make the cold solution, a volume of 1X phosphate buffered saline (PBS) was prepared by diluting 10X PBS (Sigma Aldrich) into sterile water. Oxytocin was then weighed and added to the 1X PBS, making a solution of either 15.0 mg/ml or 69.6 mg/ml oxytocin in 1X PBS, pH 7. The solution was vortexed to ensure proper mixing.

According to dosing calculations, the corresponding volumes of hot and cold oxytocin were then added to pre-labeled 1.5 ml microcentrifuge tubes. The tubes were then sealed with parafilm, placed in lead-lined storage containers, and stored at -20°C until ready for use.

A solution of 2.29% Avicel ® was made in 1X PBS by combining 1.145 g Avicel and 48.855 ml 1X PBS. The Avicel ® was first added to a 50 ml conical tube to which 5-10 ml 1X PBS was added. The mixture was vigorously stirred to combine. 1X PBS was added in 5-10 ml increments and vortexed until 48.855 ml 1X PBS had been added. To thoroughly incorporate the mucoadhesive, high speed mixing was necessary. A probe sonicator was used to homogenize the solution which reached a yogurt-like consistency when adequately mixed. However, when shear

was applied to the Avicel solution, the texture immediately loosened to a milk-like state. Again, as the solution set, the yogurt-like consistency returned. This mixture was stored in a sealed conical tube at ambient temperature.

On the day of delivery, the dosing solution was brought to room temperature on the bench top. If necessary, 2.29% mucoadhesive was added when the solution reached ambient temperature. The resulting solution contained 1% Avicel, 45 uCi, and 0.4 mg oxytocin. All dosing solutions were vortexed prior to delivery.

Animals
Adult male Sprague Dawley rats (N=41, 225-250 g) were used throughout the study. Animals were group-housed in the Regions Hospital Animal Care Facility with free access to food and water. Animals were kept on a 12 h light cycle. All experimental procedures were approved by the Animal Care and Use Committee at HealthPartners Research Foundation.

There were four experimental groups of animals: group I received ^{125}I-Oxytocin and was analyzed with gamma counting, group II received ^{125}I-Oxytocin with 1% Avicel and was analyzed with gamma counting. group III received ^{125}I-Oxytocin and was analyzed with autoradiography, and group IV received ^{125}I-Oxytocin with 1% Avicel and was analyzed with autoradiography.

Anesthesia
Prior to anesthesia, each rat was weighed.

a. An anesthesia cocktail was made containing 3 parts ketamine, 3 parts xylazine, and 1 part acepromazine. A separate syringe containing pentobarbital was also drawn.
b. A 1-cc syringe fitted with a 22G, 1 in needle was assembled.
c. Full, half, and quarter doses of the ketamine cocktail were calculated according to the animal’s weight at 0.5 ml/kg. A half dose of pentobarbital was also calculated at 50 mg/kg for use just prior to exsanguination.
d. A full dose of the ketamine cocktail was drawn into the syringe for injection.
e. The rat was placed ventral side down in the middle of a small hand towel.
f. The towel was wrapped around the animal’s head and shoulders to restrain it.
g. With the animal restrained, a large flap of skin was gently pinched above the animal’s right rear limb.
h. The needle was inserted subcutaneously into the skin fold at a 45° angle.
i. The plunger was pulled back to check for air or a blood flash.
j. If no blood was present, the anesthesia was pushed into the skin fold.
k. The rat was removed from the hand towel and placed in a holding cage.
l. The time and volume of anesthesia was noted on the animal’s data sheet.
m. The animal’s anesthesia level was assessed by pinching of the hind paw or tail. If the animal solicited a reflex, a ½ or ¼ dose booster was administered as necessary.
n. If the animal required an additional booster during drug delivery, the booster was given subcutaneously above the animal’s left hind limb as follows:
    i. A skin flap above the hip joint was gently pinched and the needle inserted into the fold.
    ii. The plunger was pulled back to check for a blood flash.

iii. If none was present, the booster was injected.
iv. The time and dose were noted on the data sheet.

Intranasal Delivery of ^{125}I-Oxytocin

Anesthesia was monitored throughout the procedure. The animal's reflex was assessed by pinching the hind paw or tail while stabilizing the head to prevent movement. Quarter and half dose boosters of the ketamine cocktail were delivered subcutaneously as needed. Intranasal delivery was performed as follows:

a. The fully anesthetized rat was placed on its back on a heating pad in a metal surgical tray inside a fume hood. A properly lubricated rectal probe was inserted to monitor and maintain the rat's core temperature at 37°C.
b. Two 2"x2" gauze pads were rolled into a pillow and taped to secure. The pillow was then placed under the rat's head to maintain the position of the head so that the underside of the neck and mouth were horizontal.
c. A cotton swab to be used for occlusion of nostrils was covered with parafilm.
d. A lead impregnated shield was placed in front of the surgical tray to protect the experimenter from radiation.
e. The dose solution, pipette, 30 ul pipette tips, and waste receptacle were arranged behind the shield for easy access.
f. A series of three 3 ul standards were aspirated from the microcentrifuge tube containing the dosing solution and expelled into pre-weighed and labeled gamma counting tubes.
g. A 6 ul drop was loaded into the pipette behind the shield.
h. The cotton swab was used to occlude one naris completely. The flat part of the swab was pushed gently against the naris at approximately a 45° angle to prevent airflow.
i. Holding the pipette vertically, the 6 ul drop was slowly expelled, forming a drop at the end of the tip.
j. The drop was lowered onto the open naris to be inhaled and the cotton swab was removed from the opposite naris.
k. The drop was inhaled.
l. The time and volume of the drop were recorded on the animal's data sheet.
m. After two minutes, the alternate naris was occluded and a 6 ul drop was administered in the same fashion to the open naris. The cotton swab was removed and the time was noted.
n. A drop was administered as described above every two minutes to alternating nares until a total of 8 drops were delivered (4 to each naris) for a total of ~48 ul.
o. Each drop was noted on the data sheet as well as any details regarding the animal or success of delivery.

Transcardial Perfusion

a. Eight minutes prior to exsanguination, a half dose of pentobarbital was administered as an intraperitoneal injection to the anesthetized rat to ensure surgical level of sedation.
b. Two min before the desired sacrifice time, the rectal probe and heating pad were removed from the metal surgical tray. While still on its back, labeling tape was used

to secure the animal's front limbs to the pan. The back of the pan was elevated slightly to allow blood to flow away from the animal.

c. A toothed forceps was used to grasp the skin/fur covering the sternum. A cut was made through the animal's skin, exposing the sternum.
d. The sternum was clamped with a hemostat. The rib cage was cut open laterally, exposing the diaphragm.
e. The diaphragm was cut to expose the pleural cavity.
f. Surgical scissors were used to cut up the sides of the ribcage toward the armpits of the animal, creating a 'V' shaped incision exposing the heart.
g. The hemostat holding the sternum was taped above the animal's head to hold the cavity open.
h. The heart was stabilized using the blunt forceps while a 1 cc-syringe with 20 G, 1" sharp needle was inserted into the left ventricle. Approximately 0.2 cc of blood was removed and placed into a pre-weighed tube for gamma counting.
i. A small cut was made in the left ventricle using a spring scissors and an 18 G blunt needle attached to an extension set filled with 60 cc of saline was inserted through the left ventricle and into the aorta.
j. A large bulldog clamp was placed just above the heart on the aorta, securing the blunt needle in place.
k. The animal was perfused with 60 ml of saline followed by 360 ml of 4% paraformaldehyde at a rate of 15 ml/min.

Brain Dissection- Gamma Counting

Immediately after collection, each tissue sample was placed into a pre-labeled and pre-weighed gamma tube for quantification.

a. The skin and muscle around the neck were cut with a scalpel just above the shoulder blades. A large curved scissors was used to decapitate the animal, cutting dorsal to ventral to avoid contamination from the trachea and esophagus.
b. A midline incision was made on the dorsal side of the skull. The skin was peeled back up to the eyes.
c. A straight hemostat was used to peel the bone away from the top half of the brain. The top of the brain was exposed all the way to the olfactory bulbs.
d. Using a spring scissors and curved forceps, dorsal dura was collected off the surface of the brain.
e. The head was inverted and the brain was removed using a small spatula to free it from the cavity. The posterior optic nerve and trigeminal nerves were trimmed close to the brain using a spring scissors. The brain was then placed into a clean Petri dish, covered with a kim-wipe moistened with 1X PBS, and placed aside for later dissection.
f. The ventral dura was collected from the skull cavity by scraping a forceps on the walls. The collected dura was placed into a pre-weighed gamma tube.
g. The ophthalmic branch of the trigeminal nerve was visualized on the inside of the skull. The scalpel was then used to carefully slice through the skull on the caudal side of the nerve, creating a vertical slice in the wall of the skull running parallel to the nerve.

h. A small hemostat was used to chip away the wall of the skull just up to the ophthalmic nerve.
i. A small spatula was run under the nerve and the skull, freeing the nerve from the skull wall. The nerve was then trimmed from the cavity and placed in the appropriate tube.
j. Once both sides of the ophthalmic nerve had been collected, the small hemostat was again used to chip away the bottom portion of the skull walls on both sides.
k. When the muscle layers were visible, the small spatula was used to tease out the mandibular nerve on both sides. Care was taken to retrieve a large portion by following the nerve into the lower jaw and chipping bone away with a hemostat if necessary.
l. Finally, the maxillary nerve and trigeminal ganglion were collected. The ganglion were removed with a curved forceps, trimming them from the maxillary segment just in front of the branching using a spring scissors.
m. The maxillary segment was collected by first removing any residual dura from the area using a forceps. Then, the curved forceps was used to gently pull the maxillary nerve while a spring scissors was inserted into the cavity as far as possible and used to snip the nerve from the remaining portion.
n. Once all of the nerve segments had been collected, the head was set aside and covered with a moist kim-wipe for later dissection.
o. The brain was retrieved and uncovered for dissection.
p. The brain was placed dorsal side down in a coronal brain matrix.
q. A razor blade was inserted at the optic chiasm. Additional blades were placed every 2 mm outward from the optic chiasm. Slices were generated from the midbrain to the tip of the forebrain, resulting in 6 slices. The blades were removed and inverted for dissection.
r. The olfactory bulbs were retrieved from the brain matrix and trimmed of any excess tissue. The bulbs were rostral to the first slice.
s. Tissues were dissected from corresponding slices (1-6) as diagrammed in Appendix E.
t. The remaining brain tissue in the matrix was retrieved and the upper cervical spinal cord was dissected and placed in the appropriate tube.
u. The brain segment was then bisected along the midline. Following figure 7 in Appendix E, the midbrain, pons, and medulla were collected by scoring each half straight down from the inferior colliculus. This marked the midbrain. Another score was made under the point in the fourth ventricle, marking the medulla. The cerebellum was peeled off and placed in the corresponding tube. If necessary, remaining cortex was also removed. Following the previously made scores, the midbrain, pons, and medulla were dissected and placed into pre-weighed tubes.
v. Next, the head was retrieved and the ventral side of the neck was cut anteriorly. The skin was peeled back exposing the neck muscles. The superficial nodes were dissected, cleared of connective tissues, and placed in pre-weighed gamma tubes.
w. The deep cervical nodes were located, dissected, cleared of connective tissue, and placed into pre-weighed tubes.
x. The thyroid was collected by gently peeling it off of the trachea with a forceps.

y. A single edge razor blade was then used to bisect the skull along the midline. The olfactory and respiratory epitheliums were collected from the cavity by scoring the edges and removing with a forceps. Care was taken to avoid the nasal septum.

Brain Dissection- Autoradiography

Prior to dissection, the autoradiography slides were prepared. A piece of corrugated cardboard was cut approximately 2 inches wider and ½ the length of the autoradiography screen to be used. Seven microscope slides were secured to the cardboard with white glue. The cardboard and slides were labeled for later identification.

a. The brain was removed from the skull as described above.
b. The ventral dura was collected as described above and placed into a pre-weighed gamma tube.
c. Next, the trigeminal ganglion with a portion of each of the three major branches was collected. First, the ophthalmic branch of the trigeminal nerve was visualized on the inside of the skull. The scalpel was then used to carefully slice through the skull on the caudal side of the nerve, creating a vertical slice in the wall of the skull running parallel to the nerve.
d. A small hemostat was used to chip away the wall of the skull just up to the ophthalmic nerve.
e. A small spatula was run under the nerve and the skull, freeing the nerve from the skull wall. The ophthalmic nerve was trimmed, leaving a large portion attached to the trigeminal ganglion.
f. The small hemostat was again used to chip away the bottom portion of the skull walls on both sides to visualize the mandibular portion of the nerve.
g. When the muscle layers were visible, the small spatula was used to tease out the mandibular nerve. Care was taken to retrieve a large portion by following the nerve into the lower jaw and chipping bone away with a hemostat if necessary, while still leaving the nerve attached to the trigeminal ganglion.
h. A forceps and spring scissors were used to remove any residual dura from the ganglion and maxillary nerve. A small spatula was used to tease the ganglion and maxillary portion from the cavity. Using a curved forceps, the ganglion with three branches attached was carefully removed from the skull. A spring scissors was used to trim the last branch, the maxillary nerve, freeing it from the skull.
i. The ganglion and branches were then arranged on the microscope slide in the anatomical configuration, leaving all three branches easily visible, stemming from the ganglion.
j. When both ganglion had been collected and arranged on the microscope slide in the proper orientation, the head was set aside and covered with a moist kim-wipe for later dissection.
k. The brain was retrieved and placed dorsal side down in a coronal brain matrix.
l. A razor blade was inserted at the optic chiasm and the blade was marked with a permanent marker for later identification.

m. Additional blades were placed every 1 mm outward from the optic chiasm. Slices were generated along the entire brain, from the front of the olfactory bulbs to the back of the cerebellum.
n. The blades were removed one by one starting at the olfactory bulbs, inverted, and arranged on the microscope slides.
o. The slices were labeled in reference to the optic chiasm, positive in the rostral direction and negative in the caudal direction.
p. Once all of the slices in the brain matrix were positioned on the slides, the remaining upper cervical spinal cord was retrieved.
q. Using a forceps and single edged razor blade, the upper cervical spinal cord was stabilized while 1 mm slices were cut. The slices were then arranged on the slides and labeled for later identification.
r. Next, the superficial nodes, deep cervical nodes, thyroid, and olfactory and respiratory epitheliums were collected as described above.

Body Dissection

a. The rat was placed on its stomach and a superficial incision was made down the length of the animal from shoulders to hips, following the spine.
b. The skin was peeled away from the underlying tissue on both sides to expose the shoulder blades.
c. The axillary nodes in the connective tissue surrounding the shoulders were dissected, cleared of connective tissue, and placed in pre-weighed tubes.
d. A piece of right deltoid muscle was dissected, approximately 3 mm square, and placed into a pre-weighed gamma tube.
e. The rat was flipped onto its back and the sternum was cut vertically to further expose the abdominal and pleural cavities.
f. 3 mm square samples of the following tissues were dissected and placed into pre-weighed gamma counting tubes.
   1. liver, right superficial lobe
   2. left kidney, tip
   3. right lung, top lobe
   4. spleen, tip
   5. heart, apex
f. Urine was removed from the bladder using a 1cc syringe and transferred to a pre-weighed tube.
g. A section of both the trachea and the esophagus were collected at the point of decapitation and placed in respective tubes.
h. The body was flipped over onto its stomach. The muscles overlying the spine were scored with a scalpel. A small hemostat was inserted into the spinal column, above the cord, at the point of decapitation. The hemostat was clamped on the overlying vertebrae and tissues. The vertebrae and tissues were chipped away using an upward motion. The hemostat was inserted into the column repetitively, chipping away small pieces of the column each time, until the entire length of the cord was exposed.
i. When the spinal cord was fully exposed, a small spatula was used to loosen the cord from the spinal cavity. The spinal cord was than removed using a forceps and placed into a petri dish.

j. The intact spinal cord was cut into lower cervical, thoracic, and lumbar portions. The dura was peeled off of each section using a forceps and small spatula. All sections and the dura were then placed in respective pre-weighed tubes.

Tissue Counting

The pre-weighed gamma tubes containing samples were reweighed to determine tissue weight. Tissue samples were then counted using a COBRA II Auto-Gamma Counter. The samples were counted under a standard ^{125}I protocol with 5 min count time and an elevator position of 3. In analysis of gamma counting results, any value outside two standard deviations of the mean for each tissue was considered an outlier and removed from the data set.

Autoradiography

Samples were placed in a felt lined box and an autoradiography screen (Super Sensitive, Packard Instrument Co.) was placed on top of the tissue samples. The box was closed and allowed to rest for 4 weeks. After 4 weeks, the screen was developed using the Packard Cyclone Storage Phosphor System.

Data Analysis and Calculations

Sample calculations for rat 1 were completed by hand and imported to Microsoft EXCEL®. All values were then auto-calculated using identical spreadsheets and double checked for accuracy.

Sample Calculations

Specific activity from Amersham Specs = (uCi delivered*decay on day counted/decay on day delivered)/estimated nmol delivered * (2.22*10^6 CPM/fmol) * counting efficiency * (10^{-6}) = specific activity (cpm/fmol)

Example: Rat 1 = (45.58 uCi * 0.9439 / 0.9439) / 402.23 nmol * (2.22 * 10^6 CPM/fmol) * 0.8 * (10^{-6}) = 0.201 CPM/fmol

Specific Activity from Standards = 1/ (average volume of standards (ul) * concentration of dose solution (ug/ul)/oxytocin molecular weight (ug/umol)/ average of standards (cpm-background)*10^9 fmol/umol) = specific activity (CPM/fmol)

Example: Rat 1 =1/ (3 ul *8.44 ug/ul /1007ug/umol /4,129,668 cpm * 10^9 fmol/umol)
= 0.164 CPM/fmol

nM tissue concentration (from Amersham Specs) = (CPM-background)/ (specific activity from specs in cpm/fmol)/ (tissue weight) *10^3 ml/L*10^{-6} fmol/nmol = nM tissue concentration

Example: Rat 1, Olfactory bulbs= (3282-25 CPM)/ (0.201 CPM/fmol)/ (0.06798 g)/10^3
= 238.08 nM

nM tissue concentration (from standards) = (CPM -background)/tissue weight/ (specific activity from standards in CPM /fmol) *10^3 ml/L*10^{-6} fmol/nmol = nM tissue concentration

Example: Rat 1, Olfactory bulbs= (3282-25 CPM)/ (0.164 CPM/fmol)/ (0.06798 g)/10^3=
291.66 nM

Results

Intranasal Delivery of ^{125}I-Oxytocin

Intranasal oxytocin successfully reaches the central nervous system with very significant concentrations in the trigeminal nerve branches and trigeminal ganglion. The trigeminal ganglion contained an average concentration of 311 nM while the three major branches, maxillary, mandibular, and ophthalmic, contained average concentrations of 264 nM, 291 nM, and 320 nM respectively (Table 01). The average concentration of olfactory bulb samples was 33 nM (Table 01). The high concentrations in these tissues confirm delivery to the brain along the olfactory and trigeminal nerve pathways. The highest delivery of oxytocin in the brain was observed in the caudate/putamen (41 nM), parietal cortex (28 nM), septal nucleus (28 nM), pons (26 nM), and medulla (25nM, Table 01). Trigeminal nerve entry into the brainstem may account for the high concentrations in the pons and medulla. The lowest concentration of oxytocin in the brain was found in the cerebellum at 10 nM (Table 01). The dorsal and ventral dura/meninges also contained high concentrations of oxytocin at 152 and 271 nM, respectively (Table 01). As the Kd for high affinity oxytocin receptors is ~1 nM and the Kd for low affinity oxytocin receptors is ~ 50 nM, this data suggests that at the dose administered, high affinity oxytocin receptors would be activated in all CNS tissues and both high and low affinity receptors would be activated in the trigeminal system. The concentration of oxytocin in the respiratory epithelium averaged 680,567 nM (Table 01) leading to effective delivery along the trigeminal nerve pathway. The average concentration of oxytocin in the upper cervical spinal cord was higher than anticipated at 33 nM (Table 01). The remaining spinal cord segments, lower cervical, thoracic, and lumbar, contained average concentrations of 4 nM, 5 nM, and 5 nM respectively (Table 02). Again, these concentrations are high enough to activate high affinity oxytocin receptors. Although our measurements were made 30 minutes after initiating intranasal treatment and 16 minutes after ending intranasal treatment, intranasal oxytocin likely reaches the trigeminal nerves within a minute or two following administration to the nasal cavity and reaches the brain within 10 minutes. Thirty minutes after initiation of intranasal delivery, the concentration of radiolabel in the blood was 61 nM (Table 01). As the half-life of oxytocin in the blood is about 10 minutes, a significant portion of this radiolabel will represent degraded oxytocin. Irrespective of the amount of degradation in the blood, the oxytocin concentration is at least five-times higher in the trigeminal ganglion than in the blood at this time.

*Note: Rats 1, 2, and 3 were omitted from the data due to a variance in the anesthesia used. Initially, pentobarbital was selected for anesthesia but due to severe breathing problems experienced by these three animals during experimentation, the anesthesia was changed to a ketamine cocktail for animals 4 through 41.

Intranasal Delivery of ^{125}I-Oxytocin with 1% Avicel ® Mucoadhesive

The addition of a mucoadhesive to the delivery solution was investigated in attempt to increase the efficiency of intranasal delivery. Oxytocin was again successfully delivered to the brain using intranasal delivery with the addition of a mucoadhesive. The average concentration of olfactory bulb samples was 60 nM (Table 02). The trigeminal ganglion contained an average concentration of 150 nM while the three major branches, maxillary, mandibular, and ophthalmic, contained average concentrations of 340 nM, 477 nM, and 278 nM respectively (Table 02). The highest delivery of oxytocin in the brain was observed in the septal nucleus (40 nM), caudate/putamen

(39 nM), anterior olfactory nucleus (39 nM), and frontal cortex (38 nM, Table 02). High concentrations in the pons (27 nM) and medulla (27 nM) may result from close proximity to the trigeminal nerve entry in the brainstem. The lowest concentration of oxytocin in the brain was found in the thalamus at 10 nM (Table 02). Concentration of oxytocin in the respiratory epithelium averaged 817,642 nM (Table 02) leading to effective delivery along the trigeminal nerve pathway. Again, high affinity oxytocin receptors would be activated in all CNS tissues while low affinity receptors would be activated in the trigeminal system. The average concentration of oxytocin in the upper cervical spinal cord was higher than anticipated at 50 nM (Table 02). The remaining spinal cord segments, lower cervical, thoracic, and lumbar, contained average concentrations of 4 nM, 5 nM, and 4 nM respectively (Table 02). At 30 min after the onset of delivery, blood samples average 74 nM oxytocin (Table 02).

Comparison of Intranasal Oxytocin and Intranasal Oxytocin with Mucoadhesive

The addition of 1% Avicel mucoadhesive to the dosing solution did not markedly affect the tissue concentrations achieved at the 30 min sacrifice time. Systemic tissue concentrations between drug formulations remained relatively unchanged with identical concentrations found in the liver (13 nM), esophagus (25 nM), and lung (21 nM, Table 03). Despite this, the concentration of oxytocin found in the thyroid was over three times higher with the addition of mucoadhesive to the dosing solution (Table03). The superficial and cervical nodes also exhibited a 1.5 fold increase with the addition of mucoadhesive (Table 03). Within the brain, the addition of mucoadhesive to the dosing solution caused an increase in concentration of several rostral tissues including olfactory bulbs, anterior olfactory nucleus, frontal cortex, and septal nucleus (Table 03). However, of the tissues that increased in concentration with the addition of mucoadhesive to the dosing solution, only the olfactory bulbs increased enough to reach concentrations capable of activating low affinity oxytocin receptors. In addition, the concentration of oxytocin in the trigeminal ganglion of animals receiving mucoadhesive was only half that of animals that did not receive mucoadhesive (Table 03). Both groups contained concentrations high enough to activate high affinity receptors in all CNS tissues and high enough to activate low affinity receptors in the trigeminal system. Animals receiving mucoadhesive had higher concentrations in the respiratory epithelium but lower in the olfactory epithelium when compared to animals that did not receive mucoadhesive Table 03). This may be a result of the increased viscosity of the dosing solution containing mucoadhesive, which makes it more difficult to move all the way up past the turbinate to the olfactory epithelium at the roof of the nasal cavity. Mucoadhesive also increased oxytocin concentrations in the dorsal and ventral dura at 30 minutes (Table 03). These findings encourage studies with extended time points to investigate the advantage of mucoadhesive as a function of time.

Qualitative Distribution of Oxytocin after Intranasal Delivery

Qualitative autoradiography studies confirmed that intranasal oxytocin successfully reached the central nervous system. The distribution of oxytocin in autoradiography experiments was similar to that identified by gamma counting, with the greatest intensity in the trigeminal ganglion, olfactory bulbs, septal nucleus, and frontal cortex (Figure 01). In some cases non-symmetrical image intensity results from unequal pressure distribution on the tissues caused by the cardboard/plastic wrap when placed in the autoradiography cassette. In future studies, a thinner cardboard would be used to prevent this adverse effect and the images would be allowed to develop for a longer period of time, producing more detailed and intense images.

Qualitative Distribution of Oxytocin after Intranasal Delivery with Mucoadhesive
Qualitative autoradiography studies completed on animals receiving ^{125}I-oxytocin with 1% Avicel ® mucoadhesive resulted in a similar distribution to ^{125}I-oxytocin animals. The highest intensity was achieved in the trigeminal nerves, olfactory bulbs, and frontal cortex (Figure 02). Again, some non-symmetrical image intensity results from unequal pressure distribution on the tissues caused by the cardboard/plastic wrap when placed in the autoradiography cassette. The quality of autoradiography images obtained from animals receiving mucoadhesive are better than the images from animals receiving only oxytocin as the mucoadhesive samples were allowed to develop for a longer period of time, producing more detailed and intense images.

Extended Time point: Intranasal Delivery at 1 h
Due to lower than expected mortality, two animals were available to test a longer time point after intranasal administration of oxytocin with and without mucoadhesive. One rat was treated with ^{125}I-oxytocin while the second rat received ^{125}I-oxytocin with 1% Avicel ® mucoadhesive; both were sacrificed at 1 hr after the onset of intranasal delivery. As with the 30 minute animals, both animals had high enough concentrations to activate high affinity oxytocin receptors in all CNS tissues and high and low receptors in the trigeminal system. The animal receiving the mucoadhesive contained higher concentrations of oxytocin in the trigeminal ganglion, maxillary nerve, mandibular nerve, and ophthalmic nerve. However, with the exception of frontal cortex, the animal receiving mucoadhesive had lower brain tissue concentrations than the animal receiving only ^{125}I-oxytocin (Table 04). In addition, the blood and majority of the peripheral tissues also had lower concentrations of oxytocin in the animal receiving the mucoadhesive (Table 04). This data is inconsistent with the 30 min data suggesting that mucoadhesive might increase the concentration of oxytocin reaching the trigeminal system over time. A larger sample size with multiple time points is necessary to adequately evaluate the efficacy of a mucoadhesive additive.

Future Studies
It is recommended that further quantitative studies be conducted at additional time points including 1, 2, and 5 hours both with and without Avicel mucoadhesive to learn more about the pharmacokinetics and clearance of intranasal oxytocin from the nasal epithelium, trigeminal nerves, brain, and upper spinal cord. In this new study, it is encouraged to consider measuring the concentration of oxytocin in the blood vessel walls of the internal carotid and Circle of Willis as migraine is thought to also involve the cerebrovasculature. Each new time point would consist of a group of 20 animals (10 with and 10 without mucoadhesive). The cost of carrying out these additional time points using gamma counting is estimated at $93,832.66, or $31,277.55 per time point.

Table 01: Intranasal Delivery of Oxytocin Data Summary

**Anesthesia**			Ketamine	Ketamine	Ketamine	Ketamine	Ketamine	Ketamine	Ketamine	Ketamine	Ketamine	Ketamine	Ketamine	Ketamine	Ketamine	Ketamine	Ketamine	Ketamine
**mg oxytocin delivered**			0.39	0.40	0.40	0.40	0.40	0.40	0.40	0.40	0.40	0.40	0.40	0.40	0.40	0.40	0.40	0.40
**nmol oxytocin delivered**			387.94	397.15	397.15	397.15	397.14	397.14	396.90	397.22	397.22	397.27	397.27	397.10	397.19	397.17	397.17	397.16
**uCi delivered**			44.0	45.0	45.0	45.0	45.0	45.0	45.0	45.0	45.0	45.0	45.0	45.0	45.0	45.0	45.0	45.0
**uL delivered**			48.0	49.1	49.4	49.4	49.9	49.9	51.0	51.9	51.9	53.1	53.1	47.7	47.7	48.2	48.2	50.0
**Delivery time**			14:00	14:02	14:03	14:02	14:01	14:12	14:01	14:09	14:02	14:00	14:00	14:00	14:05	14:00	14:07	14:00
**Sac time**			32:02	32:00	30:57	30:55	31:06	30:57	30:56	30:59	31:04	30:50	31:19	32:43	31:14	31:25	30:50	30:44
**Notes**			No breathing problems	Steady breathing throughout	Steady breathing throughout	Steady breathing throughout	Steady breathing throughout	Steady breathing throughout	Steady breathing throughout	Steady breathing throughout	Steady breathing throughout	Steady breathing throughout	Steady breathing throughout	Steady breathing throughout	Steady breathing throughout	Steady breathing throughout	Steady breathing throughout	Steady breathing throughout
	**Mean**	**SE**	**Rat 4**	**Rat 5**	**Rat 6**	**Rat 7**	**Rat 8**	**Rat 9**	**Rat 10**	**Rat 12**	**Rat 13**	**Rat 14**	**Rat 15**	**Rat 16**	**Rat 17**	**Rat 18**	**Rat 19**	**Rat 40**
**Tissue Description**	**nM**		**nM**	**nM**	**nM**	**nM**	**nM**	**nM**	**nM**	**nM**	**nM**	**nM**	**nM**	**nM**	**nM**	**nM**	**nM**	**nM**
**Blood Sample (30:00)**	61	±4.1	55	93	84	56	63	36	55	38	41	68	74	72	62	73	66	45
**Respiratory Epithelium**	680567	±85020.5	771077	928050	975234	423808	203670	180350	1104447	506223	536609	1203130	400752	848976	977456	912785	761200	155305
**Olfactory Epithelium**	4112	±693.7	7488	X	X	X	4059	2399	3710	X	X	6935	6785	1627	3681	2515	1923	X
**Trigeminal Ganglion**	311	±79.0	690	X	354	284	90	X	-	-	-	-	-	-	-	60	369	328
**Maxillary Nerve**	264	±46.2	499	X	X	350	134	X	116	475	563	229	78	85	317	120	188	277
**Mandibular Nerve**	291	±37.5	243	X	330	X	X	X	123	X	485	172	187	178	349	260	432	444
**Opthalmic Nerve**	320	±57.7	194	362	130	534	X	X	46	682	306	78	146	506	201	145	612	534
**Olfactory Bulbs**	33	±12.7	20	7	38	93	X	X	-	-	-	-	-	-	-	20	19	X
**Anterior Olfactory Nucleus**	24	±4.6	19	32	18	39	X	46	-	-	-	-	-	-	-	8	19	15
**Frontal Cortex**	21	±3.8	22	26	18	40	X	X	-	-	-	-	-	-	-	16	10	14
**Caudate/Putamen**	41	±10.8	44	71	27	108	15	26	-	-	-	-	-	-	-	10	13	54
**Septal Nucleus**	28	±6.5	19	27	29	62	27	16	-	-	-	-	-	-	-	9	6	57
**Parietal Cortex**	28	±6.8	12	37	12	64	54	32	-	-	-	-	-	-	-	10	13	17
**Hippocampus**	16	±3.0	7	11	16	31	28	9	-	-	-	-	-	-	-	6	14	17
**Thalamus**	15	±5.6	7	12	9	8	59	7	-	-	-	-	-	-	-	5	13	12
**Hypothalamus**	21	±3.9	10	37	23	29	X	23	-	-	-	-	-	-	-	8	18	X
**Midbrain**	14	±2.7	8	19	12	13	X	9	-	-	-	-	-	-	-	6	12	30
**Pons**	26	±9.6	12	24	13	25	100	9	-	-	-	-	-	-	-	6	14	26
**Medulla**	25	±8.8	14	22	12	43	90	9	-	-	-	-	-	-	-	7	15	17
**Cerebellum**	10	±1.4	11	X	12	17	X	9	-	-	-	-	-	-	-	5	8	10
**Dorsal Dura**	152	±11.6	134	X	X	X	X	58	168	X	190	128	155	201	142	177	172	154
**Ventral Dura**	271	±43.4	218	388	326	X	235	51	136	591	495	518	208	350	188	103	64	193
**Spinal Dura**	31	±7.9	22	20	14	28	46	127	14	16	17	43	34	29	19	10	X	X
**Upper Cervical Spinal Cord**	33	±7.5	14	65	35	X	65	19	-	-	-	-	-	-	-	11	26	26
**Lower Cervical Spinal Cord**	4	±0.3	4	4	5	5	5	4	4	4	3	4	X	X	3	5	6	2
**Thoracic Spinal Cord**	5	±0.5	5	6	5	X	7	X	3	4	3	5	X	4	3	4	X	8
**Lumbar Spinal Cord**	5	±0.6	5	5	4	X	X	X	3	2	2	4	7	5	X	3	9	6
**Superficial Nodes (2)**	232	±25.3	144	X	X	X	99	157	333	276	401	335	240	236	110	212	267	207
**Cervical Nodes (2)**	213	±35.5	214	126	X	X	159	294	110	452	306	277	123	95	494	134	116	88
**Axillary Nodes (2)**	22	±2.1	18	25	18	21	42	24	12	12	21	29	20	X	20	14	33	18
**Thyroid**	213	±35.5	214	126	X	X	159	294	110	452	306	277	123	95	494	134	116	88
**Muscle (R, deltoid)**	14	±1.2	9	15	17	11	22	19	7	10	10	14	X	X	12	10	21	16
**Liver (R, superficial lobe)**	13	±1.3	15	X	23	17	18	20	7	12	9	11	X	14	10	13	11	6
**Kidney (L, tip)**	44	±3.0	38	47	64	48	59	25	37	35	52	X	X	46	38	35	54	33
**Lung (R, top lobe)**	21	±2.4	33	17	19	33	20	12	26	11	14	12	41	23	8	19	31	12
**Trachea (near cut-off)**	26	±3.2	16	40	31	31	52	22	16	10	13	34	40	22	14	36	26	9
**Esophagus (near cut-off)**	25	±2.0	13	X	30	21	31	21	13	17	34	35	19	24	18	32	25	35
**Spleen (tip)**	23	±2.1	15	39	37	19	28	24	13	17	15	29	30	20	11	24	15	27
**Heart**	16	±1.2	20	22	16	18	X	23	13	11	12	19	X	16	22	13	13	9
**Urine (fill to line of tube)**	73	±11.2	15	34	121	24	97	46	134	67	44	99	146	X	50	124	57	36
**Drug Standard**			8283744	8283744	7849263	7849263	8423547	8423547	8969882	8113623	8113623	7961561	7961561	10061372	10061372	8464974	8486112	7443003
**Drug Standard**	8788619	±122341	8558332	8558332	8253577	8253577	9289377	9289377	9065938	7847807	7847807	8669004	8669004	9990586	9990586	9782016	9768403	7172863
**Drug Standard**			9947728	9947728	9061085	9061085	7579119	7579119	10091400	9793279	9793279	8105591	8105591	9709856	9709856	8909867	8899521	9802843
**CPM/fmol**	0.17	±0.0	0.16	0.16	0.16	0.16	0.17	0.17	0.16	0.16	0.16	0.16	0.16	0.17	0.17	0.18	0.18	0.16

AUTORADIOGRAPHY

Table 02: Intranasal Delivery of Oxytocin with 1% Mucoadhesive Data Summary

Anesthesia			Ketamine	Ketamine	Ketamine	Ketamine	Ketamine	Ketamine	Ketamine	Ketamine	Ketamine	Ketamine	Ketamine	Ketamine	Ketamine	Ketamine	Ketamine	Ketamine
mg oxytocin delivered			0.4	0.4	0.4	0.4	0.4	0.4	0.4	0.4	0.4	0.4	0.4	0.4	0.4	0.4	0.4	0.4
nmol oxytocin delivered			397.1	397.1	397.2	397.2	397.2	397.1	397.2	397.2	397.2	397.1	397.1	397.1	397.1	397.1	397.2	397.2
uCi delivered			45.0	45.0	45.0	45.0	45.0	45.0	45.0	45.0	45.0	45.0	45.0	45.0	45.0	45.0	45.0	45.0
uL delivered			49.2	49.2	49.5	49.5	50.0	51.1	51.4	51.4	51.4	47.5	47.7	47.7	49.2	49.2	49.5	49.5
Delivery time			14:00	14:09	14:01	14:00	14:02	14:00	14:00	14:00	14:00	14:00	14:00	14:00	14:00	14:00	14:00	14:00
Sac time			30:48	30:15	30:54	31:00	30:43	30:44	30:45	30:40	30:39	31:07	30:20	30:49	30:43	30:45	30:02	31:49
Notes	IN Oxytocin with 1% Mucoadhesive		1% Avicel Mucoadhesive; No breathing problems	1% Avicel Mucoadhesive; No breathing problems	1% Avicel Mucoadhesive; No breathing problems	1% Avicel Mucoadhesive; No breathing problems	1% Avicel Mucoadhesive; No breathing problems	1% Avicel Mucoadhesive; No breathing problems	1% Avicel Mucoadhesive; No breathing problems	1% Avicel Mucoadhesive; No breathing problems	1% Avicel Mucoadhesive; No breathing problems	1% Avicel Mucoadhesive; No breathing problems	1% Avicel Mucoadhesive; No breathing problems	1% Avicel Mucoadhesive; No breathing problems	1% Avicel Mucoadhesive; No breathing problems	1% Avicel Mucoadhesive; No breathing problems	1% Avicel Mucoadhesive; No breathing problems	1% Avicel Mucoadhesive; No breathing problems
	Mean	SE	Rat 20	Rat 21	Rat 22	Rat 23	Rat 25	Rat 27	Rat 28	Rat 29	Rat 32	Rat 33	Rat 34	Rat 35	Rat 36	Rat 37	Rat 38	Rat 39
Tissue Description	nM		nM	nM	nM	nM	nM	nM	nM	nM	nM	nM	nM	nM	nM	nM	nM	nM
Blood Sample (30:00)	74	±6	70	45	104	119	123	90	85	78	58	63	57	73	72	48	44	61
Respiratory Epithelium	817642	±41406	936476	841821	728714	967799	652956	1070178	803631	X	680313	857355	X	1026380	598624	594804	923860	764083
Olfactory Epithelium	3153	±432	2047	4886	5341	3915	X	1897	3148	X	4506	1613	X	3938	X	2057	1335	X
Trigeminal Ganglion	150	±38	216	317	-	-	X	134	-	-	50	79	-	-	-	-	52	199
Maxillary Nerve	340	±50	556	314	632	X	440	91	566	296	139	336	X	310	227	109	159	583
Mandibular Nerve	477	±117	472	499	375	117	518	1422	274	X	117	119	1158	121	1112	215	160	X
Opthalmic Nerve	278	±51	X	200	342	X	408	579	164	379	115	X	554	47	258	181	105	X
Olfactory Bulbs	60	±15	55	27	-	-	X	23	-	-	125	61	-	-	-	-	70	X
Anterior Olfactory Nucleus	39	±9	21	12	-	-	69	11	-	-	38	77	-	-	-	-	30	58
Frontal Cortex	38	±10	19	25	-	-	X	10	-	-	26	62	-	-	-	-	43	82
Caudate/Putamen	39	±12	15	16	-	-	85	17	-	-	X	34	-	-	-	-	22	84
Septal Nucleus	40	±13	21	10	-	-	25	9	-	-	32	23	-	-	-	-	94	104
Parietal Cortex	29	±4	11	27	-	-	45	X	-	-	31	28	-	-	-	-	22	41
Hippocampus	21	±5	9	X	AUTORAD.	-	X	8	AUTORAD.	-	34	13	AUTORADIOGRAPHY			-	26	36
Thalamus	10	±1	11	X	-	-	11	10	-	-	12	4	-	-	-	-	10	11
Hypothalamus	30	±4	38	24	-	-	30	48	-	-	X	14	-	-	-	-	20	38
Midbrain	17	±4	20	14	-	-	28	7	-	-	8	7	-	-	-	-	X	36
Pons	27	±6	14	8	-	-	X	45	-	-	49	12	-	-	-	-	25	36
Medulla	27	±7	16	12	-	-	48	18	-	-	9	12	-	-	-	-	52	50
Cerebellum	16	±2	11	20	-	-	23	9	-	-	13	8	-	-	-	-	19	24
Dorsal Dura	395	±78	X	144	X	513	X	X	662	179	548	784	X	53	675	107	255	426
Ventral Dura	992	±235	193	473	230	1414	2135	55	524	2783	507	1718	2809	63	662	55	1266	984
Spinal Dura	15	±2	10	8	24	13	26	13	16	21	8	11	15	19	X	X	X	X
Upper Cervical Spinal Cord	50	±15	36	15	-	-	116	36	-	-	8	95	-	-	-	-	40	X
Lower Cervical Spinal Cord	4	±0	6	5	4	5	5	3	7	1	4	5	4	5	X	X	3	X
Thoracic Spinal Cord	5	±0	5	4	6	5	5	5	X	5	4	5	6	6	5	X	5	X
Lumbar Spinal Cord	4	±0.3	3	3	4	5	X	3	6	5	4	4	4	X	X	5	3	X
Superficial Nodes (2)	365	±44	258	232	486	629	580	183	554	487	219	418	X	117	492	398	297	125
Cervical Nodes (2)	398	±80	415	243	183	X	812	193	586	505	209	130	222	858	X	121	131	967
Axillary Nodes (2)	19	±1.8	19	18	15	X	17	X	21	32	12	33	18	X	19	13	14	17
Thyroid	739	±110	412	378	701	674	X	1828	645	X	641	716	1042	649	1109	376	442	X
Muscle (R, deltoid)	13	±1	14	11	9	18	15	15	16	19	X	12	18	X	11	6	10	10
Liver (R, superficial lobe)	13	±1.1	20	17	9	17	X	13	15	18	7	13	10	11	6	11	7	16
Kidney (L, tip)	53	±6	73	58	76	72	90	14	X	52	59	45	41	72	42	17	45	34
Lung (R, top lobe)	21	±2	34	17	26	X	10	18	36	12	36	23	15	21	13	15	18	14
Trachea (near cut-off)	24	±2	21	15	X	23	26	31	41	41	33	22	26	24	26	13	14	9
Esophagus (near cut-off)	25	±1.6	31	14	21	23	36	23	28	X	22	29	36	20	23	27	22	23
Spleen (tip)	22	±1.6	28	16	X	28	25	X	33	18	13	21	14	15	20	25	20	26
Heart	14	±1	21	11	16	15	11	12	13	15	13	16	11	22	14	9	10	22
Urine (fill to line of tube)	72	±12	143	69	145	101	X	96	120	X	23	82	89	8	33	23	36	42
Drug Standard			9705188	9705188	9213637	9213637	8216111	8790444	9294890	9294890	8272417	8312553	9292479	9292479	9858107	9858107	8848929	8848929
Drug Standard	9160899	±78596	9084920	9084920	9665191	9665191	9339034	9475008	8517540	8517540	8561349	8534454	9263366	9263366	8025998	8025998	9714674	9714674
Drug Standard			8294524	8294524	10158390	10158390	9553041	X	9421429	9421429	9957771	9941373	8171569	8171569	9454884	9454884	9818639	9818639
CPM/fmol	0.17	±0.0	0.18	0.18	0.17	0.17	0.16	0.17	0.17	0.17	0.16	0.16	0.16	0.16	0.15	0.15	0.17	0.17

Table 03: Intranasal Oxytocin vs. Intranasal Oxytocin with 1% Mucoadhesive

	IN Oxytocin		IN Oxytocin with 1% Avicel	
	Mean	SE	Mean	SE
Tissue Description	nM		nM	
Blood Sample (30:00)	61	±4	74	±6
Respiratory Epithelium	680,567	85,021	817,642	41,406
Olfactory Epithelium	4112	±694	3153	±432
Trigeminal Ganglion	311	±79	150	±38
Maxillary Nerve	264	±46	340	±50
Mandibular Nerve	291	±38	477	±117
Opthalmic Nerve	320	±58	278	±51
Olfactory Bulbs	33	±13	60	±15
Anterior Olfactory Nucleus	24	±5	39	±9
Frontal Cortex	21	±4	38	±10
Caudate/Putamen	41	±10.8	39	±12.0
Septal Nucleus	28	±6	40	±13
Parietal Cortex	28	±7	29	±4
Hippocampus	16	±3	21	±5
Thalamus	15	±6	10	±1
Hypothalamus	21	±4	30	±4
Midbrain	14	±3	17	±4
Pons	26	±10	27	±6
Medulla	25	±9	27	±7
Cerebellum	10	±1	16	±2
Dorsal Dura	152	±12	395	±78
Ventral Dura	271	±43	992	±235
Spinal Dura	31	±8	15	±2
Upper Cervical Spinal Cord	33	±8	50	±15
Lower Cervical Spinal Cord	4	±0.3	4	±0.4
Thoracic Spinal Cord	5	±0.5	5	±0.2
Lumbar Spinal Cord	5	±0.6	4	±0.3
Superficial Nodes (2)	232	±25	365	±44
Cervical Nodes (2)	213	±36	398	±80
Axillary Nodes (2)	22	±2	19	±2
Thyroid	213	±36	739	±110
Muscle (R, deltoid)	14	±1	13	±1
Liver (R, superficial lobe)	13	±1	13	±1
Kidney (L, tip)	44	±3	53	±6
Lung (R, top lobe)	21	±2	21	±2
Trachea (near cut-off)	26	±3	24	±2
Esophagus (near cut-off)	25	±2	25	±2
Spleen (tip)	23	±2	22	±2
Heart	16	±1	14	±1
Urine (fill to line of tube)	73	±11	72	±12
Drug Standard	8,788,619	122,341	9,160,899	78,596
CPM/fmol	0.17	±0.0	0.17	±0.0

Table 04: Extended Time Point, Intranasal Oxytoin vs. Intranasal Oxytocin with 1% Mucoadhesive at 60 min.

Tissue Description	Rat 30: IN Oxytocin Mean nM	Rat 31: IN Oxytocin with 1% Mean nM
Blood Sample (60:00)	155	103
Respiratory Epithelium	1,075,273	1,078,689
Olfactory Epithelium	100,193	9,839
Trigeminal Ganglion	301	1106
Maxillary Nerve	163	538
Mandibular Nerve	201	346
Opthalmic Nerve	209	462
Olfactory Bulbs	80	17
Anterior Olfactory Nucleus	42	12
Frontal Cortex	25	66
Caudate/Putamen	31	24
Septal Nucleus	24	15
Parietal Cortex	14	12
Hippocampus	16	13
Thalamus	11	7
Hypothalamus	26	23
Midbrain	20	12
Pons	42	16
Medulla	37	12
Cerebellum	27	14
Dorsal Dura	364	232
Ventral Dura	565	7168
Spinal Dura	26	10
Upper Cervical Spinal Cord	18	26
Lower Cervical Spinal Cord	10	5
Thoracic Spinal Cord	11	7
Lumbar Spinal Cord	18	6
Superficial Nodes (2)	109	192
Cervical Nodes (2)	381	446
Axillary Nodes (2)	51	30
Thyroid	2,882	3,925
Muscle (R, deltoid)	36	28
Liver (R, superficial lobe)	22	39
Kidney (L, tip)	166	56
Lung (R, top lobe)	56	22
Trachea (near cut-off)	105	79
Esophagus (near cut-off)	59	126
Spleen (tip)	68	48
Heart	35	30
Urine (fill to line of tube)	676	103
Drug Standard	3,995,562	4,029,771
CPM/fmol	0.17	0.16

Figure 01: Qualitative Distribution after Intranasal Delivery of ^{125}I-Oxytocin

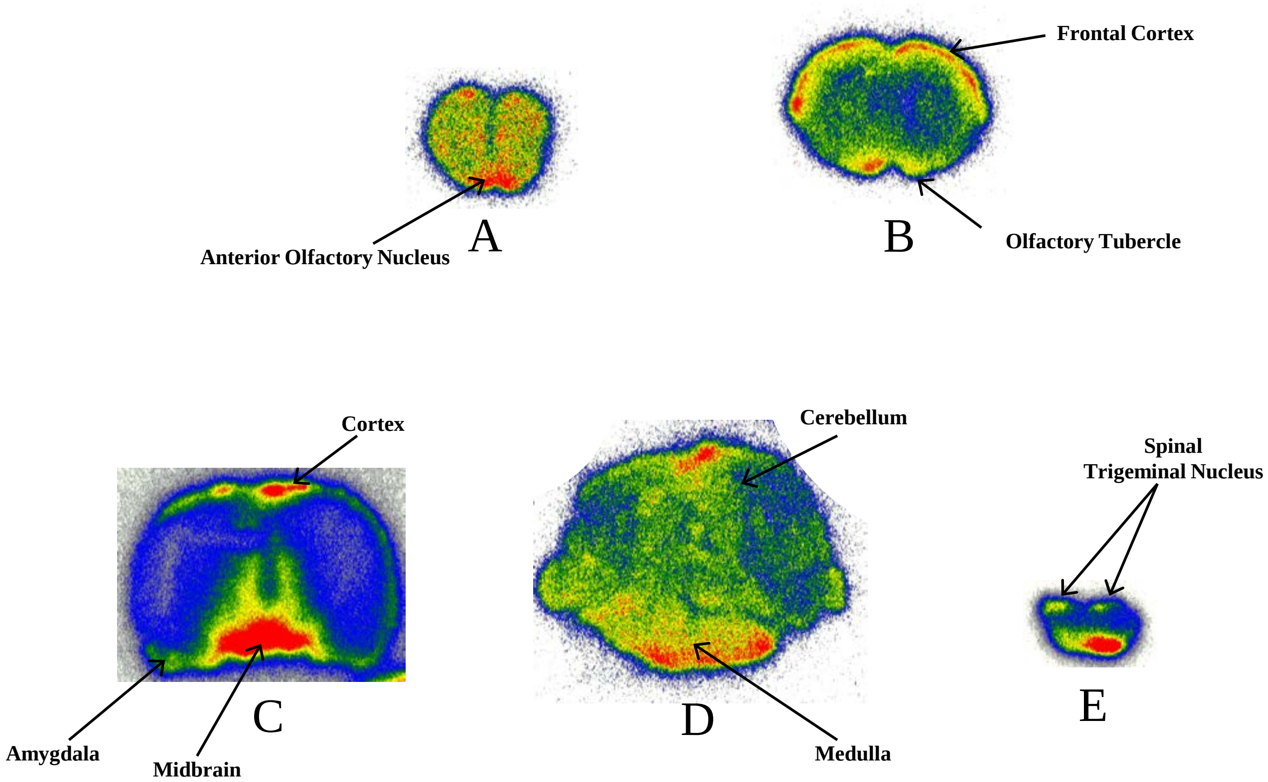

A) Rat 16, slice +6. Image intensity: 400-1167.

B) Rat 12, slice +3. Image intensity: 312-1411.

C) Rat 11, slice -7. Image intensity: 115-1824.

D) Rat 14, slice -12. Image intensity: 277-1040.

E) Rat 13, slice -19. Image intensity: 359-2151.

*Slices were measured from Bregma.
**All images were exported at 200% magnification and displayed at 20%.

Figure 02: Qualitative Distribution after Intranasal 125I-Oxytocin with 1% Avicel Mucoadhesive

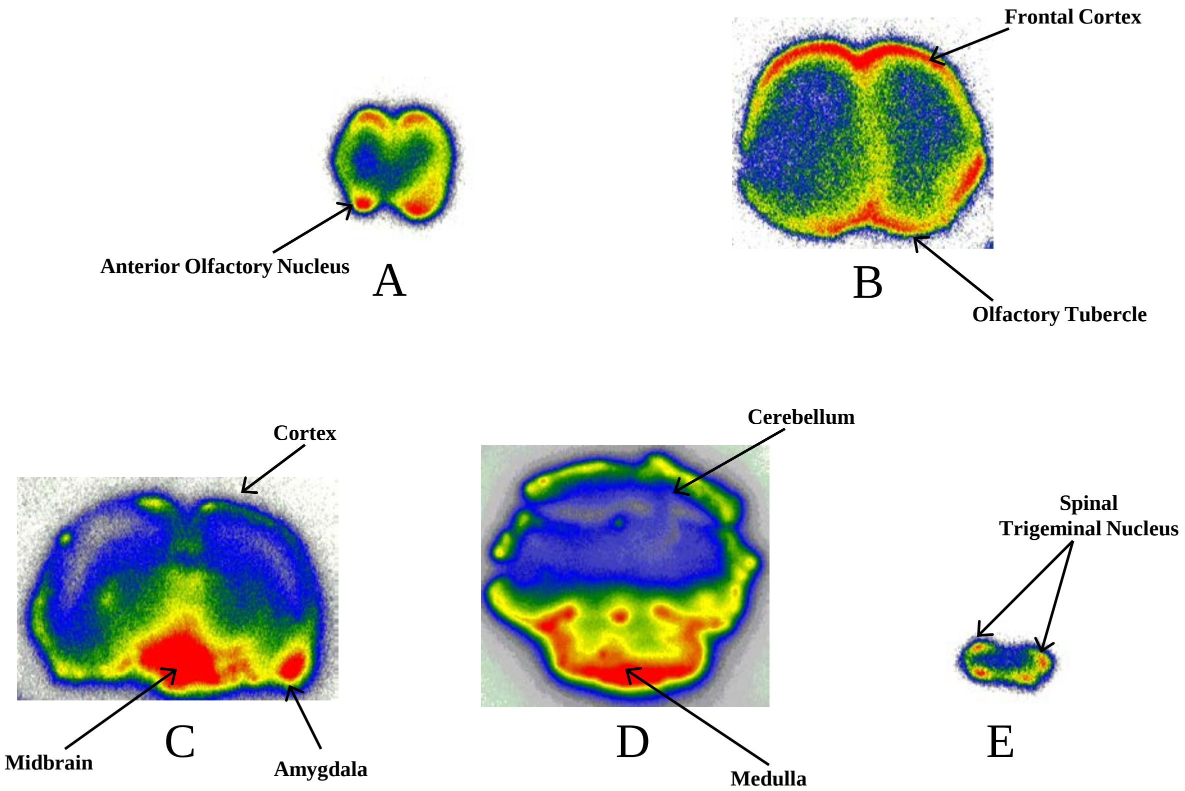

A) Rat 29, slice +7. Image intensity: 492-3,287.

B) Rat 29, slice +3. Image intensity: 331-1,140.

C) Rat 23, slice -7. Image intensity: 413-3,432.

D) Rat 28, slice -13. Image intensity: 0-25,757.

E) Rat 29, slice -18. Image intensity: 648-2,201.

*Slices were measured from Bregma.
**All images were exported at 200% magnification and displayed at 20%.

Figure 03: Trigeminal Ganglion of Animals Receiving ^{125}I-Oxytocin and ^{125}I-Oxytocin with 1% Avicel Mucoadhesive

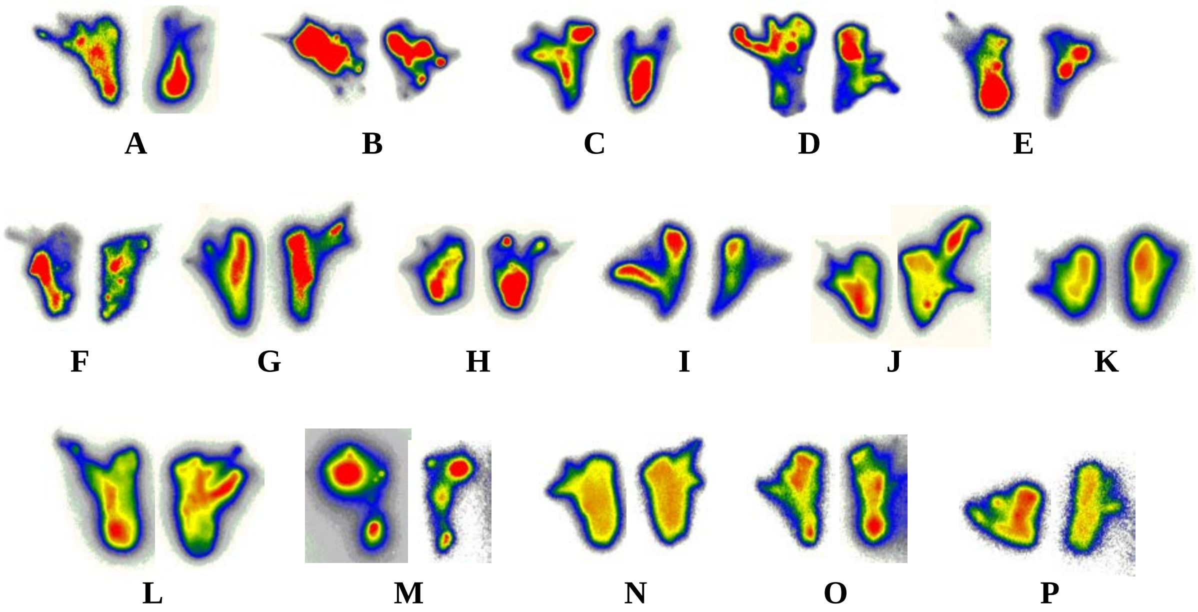

Intranasal ^{125}I-Oxytocin Animals:

A) Rat 10: Left (intensity: 420-2970) and Right (intensity: 329-19,546)Trigeminal Ganglion
B) Rat 11: Left (intensity: 1,278-11,494) and Right (intensity: 6,786-53,983)Trigeminal Ganglion
C) Rat 12: Left (intensity: 4,534-34,091) and Right (intensity: 1,485-18,309)Trigeminal Ganglion
D) Rat 13: Left (intensity: 3,241-18,501) and Right (intensity: 2,654-17,523)Trigeminal Ganglion
E) Rat 14: Left (intensity: 840-4,395) and Right (intensity: 979-7,371)Trigeminal Ganglion
F) Rat 15: Left (intensity: 714-7,750) and Right (intensity: 486-3,152)Trigeminal Ganglion
G) Rat 16: Left (intensity: 1,066-25,000) and Right (intensity: 677-6,222)Trigeminal Ganglion
H) Rat 17: Left (intensity: 1,094-65,283) and Right (intensity: 1,091-27,062)Trigeminal Ganglion

Intranasal ^{125}I-Oxytocin with 1% Mucoadhesive Animals:

I) Rat 22: Left (intensity: 969-10,044) and Right (intensity: 969-10,044)Trigeminal Ganglion
J) Rat 23: Left (intensity: 2,308-50,000) and Right (intensity: 2,112-27,822)Trigeminal Ganglion
K) Rat 28: Left (intensity: 1,164-30,306) and Right (intensity: 1,164-35,753)Trigeminal Ganglion
L) Rat 29: Left (intensity: 592-30,000) and Right (intensity: 986-50,000)Trigeminal Ganglion
M) Rat 34: Left (intensity: 397-39,409) and Right (intensity: 1,338-6,385)Trigeminal Ganglion
N) Rat 35: Left (intensity: 819-15,810) and Right (intensity: 667-6,669)Trigeminal Ganglion
O)Rat 36: Left (intensity: 1,009-4,664) and Right (intensity: 1,186-9,982)Trigeminal Ganglion P)
P) Rat 37: Left (intensity: 607-5,015) and Right (intensity: 489-2,623)Trigeminal Ganglion

**All images were copied at 200% magnification and displayed at 15%.

Appendix A – Intranasal Oxytocin Data

Tables

Table A01: Intranasal Oxytocin Rat 1 Data
Table A02: Intranasal Oxytocin Rat 2 Data
Table A03: Intranasal Oxytocin Rat 3 Data
Table A04: Intranasal Oxytocin Rat 4 Data
Table A05: Intranasal Oxytocin Rat 5 Data
Table A06: Intranasal Oxytocin Rat 6 Data
Table A07: Intranasal Oxytocin Rat 7 Data
Table A08: Intranasal Oxytocin Rat 8 Data
Table A09: Intranasal Oxytocin Rat 9 Data
Table A10: Intranasal Oxytocin Rat 10 Data
Table A11: Intranasal Oxytocin Rat 11 Data
Table A12: Intranasal Oxytocin Rat 12 Data
Table A13: Intranasal Oxytocin Rat 13 Data
Table A14: Intranasal Oxytocin Rat 14 Data
Table A15: Intranasal Oxytocin Rat 15 Data
Table A16: Intranasal Oxytocin Rat 16 Data
Table A17: Intranasal Oxytocin Rat 17 Data
Table A18: Intranasal Oxytocin Rat 18 Data
Table A19: Intranasal Oxytocin Rat 19 Data
Table A20: Intranasal Oxytocin Rat 40 Data
Table A21: Intranasal Oxytocin Rat 41 Data

Table A01: Intranasal Oxytocin Rat 1 Data

Oxytocin Rat 1	Route	Com-pound	Volume (uL)	uCi	mg	Time (min)	Perfused @ (min)
Amount Delivered:	IN	Oxytocin	48	45.58	0.405	14:00:00	30:40:00

Tissue Description	Tube #	Tube (g)	Tissue + Tube (g)	Tissue Weight (g)	CPM	CPM -25 bkgd	nM (Spec. Act.)	nM (Stan.)
Dorsal Dura	1	3.01010	3.02595	0.01585	782	757	237.33	290.74
Ventral Dura	2	2.97360	2.98360	0.01000	734	709	352.32	431.60
Trigeminal Ganglion	3	2.98301	3.00588	0.02287	6081	6056	1,315.87	1,611.97
Maxillary Nerve	4	2.97272	2.98487	0.01215	4525	4500	1,840.47	2,254.62
Opthalmic Nerve	5	2.98445	2.98740	0.00295	373	348	586.21	718.11
Mandibular Nerve	6	2.97976	2.98412	0.00436	1520	1495	1,703.91	2,087.33
Upper Cervical Spinal Cord	7	2.99311	3.05070	0.05759	562	537	46.34	56.76
Olfactory Bulbs	8	2.97921	3.04719	0.06798	3282	3257	238.08	291.66
Anterior Olfactory Nucleus	9	2.95899	2.99727	0.03828	698	673	87.36	107.02
Frontal Cortex	10	2.98091	3.01846	0.03755	237	212	28.06	34.37
Caudate/Putamen	11	2.96759	2.97879	0.01120	71	46	20.41	25.00
Septal Nucleus	12	2.96712	2.97780	0.01068	89	64	29.78	36.48
Parietal Cortex	13	2.96334	2.99864	0.03530	138	113	15.91	19.49
Hippocampus	14	3.00539	3.03489	0.02950	88	63	10.61	13.00
Thalamus	15	2.99637	3.05372	0.05735	183	158	13.69	16.77
Hypothalamus	16	2.99756	3.01876	0.02120	347	322	75.48	92.46
Midbrain	17	2.98102	3.10148	0.12046	737	712	29.37	35.98
Pons	18	2.95627	3.02326	0.06699	421	396	29.38	35.99
Medulla	19	2.95652	3.04044	0.08392	512	487	28.84	35.33
Cerebellum	20	2.99099	3.22203	0.23104	1128	1103	23.72	29.06
Superficial Nodes (2)	21	3.06435	3.13725	0.07290	339	314	21.40	26.22
Cervical Nodes (2)	22	2.96836	2.98147	0.01311	2014	1989	753.92	923.57
Thyroid	23	2.97883	2.99650	0.01767	3355	3330	936.48	1,147.21
Olfactory Epithelium	24	2.95726	3.05675	0.09949	4009971	4009946	200,286.73	245,355.35
Respiratory Epithelium	25	2.99576	3.06882	0.07306	4913993	4913968	334,230.23	409,438.90
Axillary Nodes (2)	26	2.99975	3.02743	0.02768	156	131	23.52	28.81
Muscle (R, deltoid)	27	3.01312	3.03442	0.02130	102	77	17.96	22.01
Liver (R, superficial lobe)	28	3.07831	3.10490	0.02659	209	184	34.39	42.12
Kidney (L, tip)	29	3.01389	3.04249	0.02860	380	355	61.68	75.56
Lung (R, top lobe)	30	2.98465	3.00985	0.02520	227	202	39.83	48.80
Trachea (near cut-off)	31	2.99365	3.00564	0.01199	25634	25609	10,613.69	13,001.99
Esophagus (near cut-off)	32	2.99155	3.00575	0.01420	8154	8129	2,844.74	3,484.86
Spleen (tip)	33	3.00784	3.04004	0.03220	271	246	37.96	46.51
Heart	34	3.04134	3.07506	0.03372	179	154	22.69	27.80
Urine (fill to line of tube)	35	2.97516	3.25863	0.28347	8318	8293	145.38	178.09
Spinal Dura	36	2.98731	3.00219	0.01488	202	177	59.11	72.41
Lower Cervical Spinal Cord	37	2.98871	3.08033	0.09162	789	764	41.44	50.76
Thoracic Spinal Cord	38	3.04743	3.13014	0.08271	389	364	21.87	26.79
Lumbar Spinal Cord	39	3.08444	3.20457	0.12013	270	245	10.13	12.42
Blood Sample	40	2.98091	3.22503	0.24412	4953	4928	100.31	122.89
Drug Standard	41	2.98305	2.98602	0.00297	4202487	4202462	7,031,377.63	8,613,581.91
Drug Standard	42	2.96106	2.96388	0.00282	4135202	4135177	7,286,820.52	8,926,504.69
Drug Standard	43	2.98902	2.99184	0.00282	4051391	4051366	7,139,132.59	8,745,583.95
							0.201 cpm/fmol	0.164 cpm/fmol

Auto Calculations		Enter Values	
Total uL of Dose Sol =	61.4	Oxytocin Molecular Weight	1,007
Hot mg =	0.000031	Date of Synthesis =	10/03/07
Cold mg =	0.5181	Hot uCi/uL at Synthesis =	**2.300**
Total mg in Dose Sol =	0.5182	Hot uCi/ug at Synthesis =	**1985.72**
Conc Of Dose Sol (ug/uL) =	8.4400	Cold ug/uL =	15.00
Days after Synthesis Delivered =	5	Desired uCi =	45.0
Decay on Day Delivered =	0.9439	Desired mg =	0.400
Total uCi in Dose Sol on Day Delivered =	58.29	Estimated Desired nmol =	397.1
uCi/uL in Dose Sol on Day Delivered =	0.9495	**Date of Delivery =**	**10/08/07**
mg delivered =	0.405	**uL delivered (actual) =**	**48.0**
uCi delivered =	45.58	**Date of Counting =**	**10/08/07**
Estimated nmol delivered =	402.23	**Dose Calculator**	
Days after Synthesis Counted =	5	uL Hot to Deliver =	20.73
Decay on Day Counted =	0.9439	uL Cold to Deliver =	26.67
Specific Activity from Amersham Specs (cpm/fmol) =	0.201	Total uL to Deliver =	47.39
Avg. Volume of Standard (uL)* =	3.0	Hot to Cold Ratio =	0.78
Avg. CPM-bkgd of Standard =	4,129,668	Total uL to Make =	61.39
Specific Activity from Standards: (CPM/fmol) =	0.164	Dose Solution uL Hot =	26.85
Date of Disposal from Decay Storage =	05/25/09	Dose Solution uL Cold =	34.54

Table A02: Intranasal Oxytocin Rat 2 Data

**Oxytocin Rat 2**	Route	Com-pound	Volume (uL)	uCi	mg	Time (min)	Perfused @ (min)
Amount Delivered:	IN	Oxytocin	48	44.88	0.399	14:30:00	19:41:00

Tissue Description	Tube #	Tube (g)	Tissue + Tube (g)	Tissue Weight (g)	CPM	CPM -25 bkgd	nM (Spec. Act.)	nM (Stan.)
Dorsal Dura	1	2.98818	3.00330	0.01512	401	376	123.57	148.55
Ventral Dura	2	3.00873	3.01614	0.00741	796	771	517.05	621.54
Trigeminal Ganglion	3	3.01057	3.03710	0.02653	9956	9931	1,860.15	2,236.08
Maxillary Nerve	4	3.00654	3.02260	0.01606	5452	5427	1,679.22	2,018.58
Opthalmic Nerve	5	3.04874	3.05097	0.00223	254	229	510.30	613.43
Mandibular Nerve	6	3.00407	3.00764	0.00357	590	565	786.45	945.39
Upper Cervical Spinal Cord	7	2.98850	3.04562	0.05712	847	822	71.51	85.96
Olfactory Bulbs	8	2.99192	3.05358	0.06166	3508	3483	280.70	337.43
Anterior Olfactory Nucleus	9	3.01640	3.05402	0.03762	514	489	64.59	77.65
Frontal Cortex	10	2.98514	3.02403	0.03889	173	148	18.91	22.73
Caudate/Putamen	11	3.07400	3.08505	0.01105	67	42	18.89	22.70
Septal Nucleus	12	3.00514	3.01517	0.01003	84	59	29.23	35.14
Parietal Cortex	13	2.99673	3.03402	0.03729	120	95	12.66	15.22
Hippocampus	14	2.97512	3.01188	0.03676	79	54	7.30	8.78
Thalamus	15	3.08133	3.15359	0.07226	186	161	11.07	13.31
Hypothalamus	16	2.98907	3.01316	0.02409	304	279	57.55	69.18
Midbrain	17	3.00710	3.10757	0.10047	464	439	21.71	26.10
Pons	18	3.05209	3.11663	0.06454	377	352	27.10	32.58
Medulla	19	2.98394	3.07279	0.08885	936	911	50.95	61.25
Cerebellum	20	2.99045	3.21777	0.22732	1624	1599	34.95	42.02
Superficial Nodes (2)	21	2.98393	3.04658	0.06265	1280	1255	99.54	119.66
Cervical Nodes (2)	22	2.99324	3.00006	0.00682	975	950	692.20	832.09
Thyroid	23	3.02080	3.04792	0.02712	4629	4604	843.60	1,014.09
Olfactory Epithelium	24	2.98865	3.11091	0.12226	5923985	5923960	240,780.22	289,440.22
Respiratory Epithelium	25	2.99542	3.03626	0.04084	2967056	2967031	361,018.48	433,977.79
Axillary Nodes (2)	26	2.99503	3.06740	0.07237	133	108	7.42	8.91
Muscle (R, deltoid)	27	2.96846	2.98938	0.02092	72	47	11.16	13.42
Liver (R, superficial lobe)	28	2.97778	3.01466	0.03688	160	135	18.19	21.87
Kidney (L, tip)	29	2.97853	3.01178	0.03325	279	254	37.96	45.63
Lung (R, top lobe)	30	3.06381	3.07934	0.01553	152	127	40.64	48.85
Trachea (near cut-off)	31	2.98044	2.99153	0.01109	8149	8124	3,640.25	4,375.92
Esophagus (near cut-off)	32	2.96610	2.97945	0.01335	1232	1207	449.28	540.08
Spleen (tip)	33	2.99118	3.03841	0.04723	205	180	18.94	22.77
Heart	34	3.00503	3.03324	0.02821	184	159	28.01	33.67
Urine (fill to line of tube)	35	2.99665	3.21820	0.22155	2044	2019	45.29	54.44
Spinal Dura	36	2.97461	2.98277	0.00816	138	113	68.81	82.72
Lower Cervical Spinal Cord	37	3.00809	3.12053	0.11244	334	309	13.66	16.42
Thoracic Spinal Cord	38	2.99645	3.06370	0.06725	207	182	13.45	16.17
Lumbar Spinal Cord	39	2.97142	3.10496	0.13354	212	187	6.96	8.36
Blood Sample	40	3.00094	3.30003	0.29909	6047	6022	100.05	120.27
Drug Standard	41	2.98465	2.98677	0.00212	4194193	4194168	9,831,120.94	11,817,921.94
Drug Standard	42	2.98250	2.98426	0.00176	3990123	3990098	11,265,850.19	13,542,599.97
Drug Standard	43	3.04367	3.04579	0.00212	4249343	4249318	9,960,392.42	11,973,318.29
							0.201 cpm/fmol	0.167 cpm/fmol

**Auto Calculations**		**Enter Values**	
Total uL of Dose Sol =	62.1	Oxytocin Molecular Weight	1,007
Hot mg =	0.0000	Date of Synthesis =	10/03/07
Cold mg =	0.5163	Hot uCi/uL at Synthesis =	**2.300**
Total mg in Dose Sol =	0.5164	Hot uCi/ug at Synthesis =	**1985.72**
Conc Of Dose Sol (ug/uL) =	8.3118	Cold ug/uL =	15.00
Days after Synthesis Delivered =	8	Desired uCi =	45.0
Decay on Day Delivered =	0.9117	Desired mg =	0.400
Total uCi in Dose Sol on Day Delivered =	58.09	Estimated Desired nmol =	397.1
uCi/uL in Dose Sol on Day Delivered =	0.9351	**Date of Delivery =**	**10/11/07**
mg delivered =	0.399	**uL delivered (actual) =**	**48.0**
uCi delivered =	44.88	**Date of Counting =**	**10/11/07**
Estimated nmol delivered =	396.12	**Dose Calculator**	
Days after Synthesis Counted =	8	uL Hot to Deliver =	21.46
Decay on Day Counted =	0.9117	uL Cold to Deliver =	26.67
Specific Activity from Amersham Specs (cpm/fmol) =	0.201	Total uL to Deliver =	48.12
Avg. Volume of Standard (uL)* =	3.0	Hot to Cold Ratio =	0.80
Avg. CPM-bkgd of Standard =	4,144,528	Total uL to Make =	62.12
Specific Activity from Standards: (CPM/fmol) =	0.167	Dose Solution uL Hot =	27.70
Date of Disposal from Decay Storage =	05/25/09	Dose Solution uL Cold =	34.42

Table A03: Intranasal Oxytocin Rat 3 Data

Oxytocin Rat 3	Route	Com-pound	Volume (uL)	uCi	mg	Time (min)	Perfused @ (min)
Amount Delivered:	IN	Oxytocin	48	44.88	0.399	14:01:00	30:30:00

Tissue Description	Tube #	Tube (g)	Tissue + Tube (g)	Tissue Weight (g)	CPM	CPM -25 bkgd	nM (Spec. Act.)	nM (Stan.)
Dorsal Dura	1	2.98222	2.99067	0.00845	852	827	486.34	584.86
Ventral Dura	2	2.98906	3.00177	0.01271	507	482	188.45	226.62
Trigeminal Ganglion	3	2.99660	3.01803	0.02143	833	808	187.36	225.32
Maxillary Nerve	4	3.01705	3.02623	0.00918	849	824	446.04	536.40
Opthalmic Nerve	5	3.01276	3.01629	0.00353	353	328	461.73	555.27
Mandibular Nerve	6	3.02464	3.02979	0.00515	164	139	134.12	161.29
Upper Cervical Spinal Cord	7	2.99489	3.00858	0.01369	264	239	86.75	104.33
Olfactory Bulbs	8	2.99530	3.07149	0.07619	548	523	34.11	41.02
Anterior Olfactory Nucleus	9	2.99413	3.02707	0.03294	144	119	17.95	21.59
Frontal Cortex	10	3.00851	3.05039	0.04188	147	122	14.48	17.41
Caudate/Putamen	11	3.00739	3.02060	0.01321	71	46	17.30	20.81
Septal Nucleus	12	3.01892	3.03334	0.01442	71	46	15.85	19.06
Parietal Cortex	13	3.01357	3.04459	0.03102	90	65	10.41	12.52
Hippocampus	14	3.01269	3.05545	0.04276	89	64	7.44	8.94
Thalamus	15	2.99047	3.06192	0.07145	179	154	10.71	12.88
Hypothalamus	16	3.07538	3.09611	0.02073	99	74	17.74	21.33
Midbrain	17	3.00260	3.11708	0.11448	340	315	13.67	16.44
Pons	18	2.99275	3.04592	0.05317	218	193	18.04	21.69
Medulla	19	3.01266	3.09667	0.08401	287	262	15.50	18.64
Cerebellum	20	3.00469	3.23542	0.23073	1008	983	21.17	25.46
Superficial Nodes (2)	21	3.01548	3.08673	0.07125	3218	3193	222.69	267.80
Cervical Nodes (2)	22	2.99721	3.00427	0.00706	223	198	139.36	167.60
Thyroid	23	3.01899	3.04046	0.02147	27680	27655	6,400.81	7,697.38
Olfactory Epithelium	24	2.96732	2.99275	0.02543	1704319	1704294	333,036.04	400,497.36
Respiratory Epithelium	25	3.01976	3.08965	0.06989	875145	875120	62,222.24	74,826.27
Axillary Nodes (2)	26	2.98992	3.00989	0.01997	182	157	39.07	46.98
Muscle (R, deltoid)	27	2.97596	2.99729	0.02133	109	84	19.57	23.53
Liver (R, superficial lobe)	28	3.00633	3.04573	0.03940	184	159	20.05	24.12
Kidney (L, tip)	29	3.00506	3.04376	0.03870	310	285	36.60	44.01
Lung (R, top lobe)	30	2.95733	2.96906	0.01173	198	173	73.29	88.14
Trachea (near cut-off)	31	2.97508	2.98648	0.01140	26636	26611	11,599.77	13,949.47
Esophagus (near cut-off)	32	2.95403	2.96635	0.01232	607963	607938	245,211.97	294,883.24
Spleen (tip)	33	2.98440	3.02191	0.03751	220	195	25.83	31.07
Heart	34	2.99520	3.04042	0.04522	251	226	24.84	29.87
Urine (fill to line of tube)	35	3.00929	3.16878	0.15949	10584	10559	328.99	395.63
Spinal Dura	36	2.97412	2.98534	0.01122	235	210	93.01	111.85
Lower Cervical Spinal Cord	37	2.96956	3.11129	0.14173	529	504	17.67	21.25
Thoracic Spinal Cord	38	2.95489	3.04533	0.09044	193	168	9.23	11.10
Lumbar Spinal Cord	39	2.99487	3.13581	0.14094	573	548	19.32	23.24
Blood Sample	40	2.98184	3.26946	0.28762	5414	5389	93.11	111.97
Drug Standard	41	2.98465	2.98677	0.00212	4194788	4194763	9,832,515.62	11,824,235.51
Drug Standard	42	2.98250	2.98426	0.00176	3983203	3983178	11,246,311.90	13,524,416.91
Drug Standard	43	3.04367	3.04579	0.00212	4250792	4250767	9,963,788.88	11,982,100.08
							0.201 cpm/fmol	0.167 cpm/fmol

Auto Calculations		Enter Values	
Total uL of Dose Sol =	62.1	Oxytocin Molecular Weight	1,007
Hot mg =	0.0000	Date of Synthesis =	10/03/07
Cold mg =	0.5163	Hot uCi/uL at Synthesis =	**2.300**
Total mg in Dose Sol =	0.5164	Hot uCi/ug at Synthesis =	**1985.72**
Conc Of Dose Sol (ug/uL) =	8.3118	Cold ug/uL =	15.00
Days after Synthesis Delivered =	8	Desired uCi =	45.0
Decay on Day Delivered =	0.9117	Desired mg =	0.400
Total uCi in Dose Sol on Day Delivered =	58.09	Estimated Desired nmol =	397.1
uCi/uL in Dose Sol on Day Delivered =	0.9351	**Date of Delivery =**	**10/11/07**
mg delivered =	0.399	**uL delivered (actual) =**	**48.0**
uCi delivered =	44.88	**Date of Counting =**	**10/11/07**
Estimated nmol delivered =	396.12	**Dose Calculator**	
Days after Synthesis Counted =	8	uL Hot to Deliver =	21.46
Decay on Day Counted =	0.9117	uL Cold to Deliver =	26.67
Specific Activity from Amersham Specs (cpm/fmol) =	0.201	Total uL to Deliver =	48.12
Avg. Volume of Standard (uL)* =	3.0	Hot to Cold Ratio =	0.80
Avg. CPM-bkgd of Standard =	4,142,903	Total uL to Make =	62.12
Specific Activity from Standards: (CPM/fmol) =	0.167	Dose Solution uL Hot =	27.70
Date of Disposal from Decay Storage =	05/25/09	Dose Solution uL Cold =	34.42

Table A04: Intranasal Oxytocin Rat 4 Data

Oxytocin Rat 4	Route	Com-pound	Volume (uL)	uCi	mg	Time (min)	Perfused @ (min)
Amount Delivered:	IN	Oxytocin	48.0	43.96	0.391	14:00:00	32:00:00

Tissue Description	Tube #	Tube (g)	Tissue + Tube (g)	Tissue Weight (g)	CPM	CPM -25 bkgd	nM (Spec. Act.)	nM (Stan.)
Dorsal Dura	1	3.01197	3.02478	0.01281	305	280	108.62	134.50
Ventral Dura	2	3.01926	3.02830	0.00904	345	320	175.90	217.82
Trigeminal Ganglion	3	3.08378	3.10802	0.02424	2745	2720	557.61	690.47
Maxillary Nerve	4	2.99555	3.01076	0.01521	1258	1233	402.83	498.82
Opthalmic Nerve	5	3.01186	3.01717	0.00531	192	167	156.28	193.52
Mandibular Nerve	6	3.03741	3.04075	0.00334	157	132	196.39	243.19
Upper Cervical Spinal Cord	7	3.01020	3.05645	0.04625	132	107	11.50	14.24
Olfactory Bulbs	8	2.98239	3.05396	0.07157	261	236	16.39	20.29
Anterior Olfactory Nucleus	9	3.03957	3.08534	0.04577	163	138	14.98	18.55
Frontal Cortex	10	3.00554	3.03952	0.03398	145	120	17.55	21.73
Caudate/Putamen	11	3.07779	3.09135	0.01356	123	98	35.91	44.47
Septal Nucleus	12	3.00202	3.01791	0.01589	75	50	15.64	19.36
Parietal Cortex	13	3.00620	3.03299	0.02679	76	51	9.46	11.71
Hippocampus	14	3.00746	3.04000	0.03254	63	38	5.80	7.19
Thalamus	15	3.01601	3.08542	0.06941	99	74	5.30	6.56
Hypothalamus	16	3.01466	3.04107	0.02641	69	44	8.28	10.25
Midbrain	17	3.07206	3.18799	0.11593	181	156	6.69	8.28
Pons	18	2.99457	3.06305	0.06848	159	134	9.72	12.04
Medulla	19	2.98287	3.06381	0.08094	209	184	11.30	13.99
Cerebellum	20	2.97841	3.24200	0.26359	484	459	8.65	10.72
Superficial Nodes (2)	21	3.01550	3.09388	0.07838	1864	1839	116.59	144.37
Cervical Nodes (2)	22	3.00593	3.01725	0.01132	418	393	172.52	213.63
Thyroid	23	3.00100	3.02549	0.02449	8750	8725	1,770.39	2,192.23
Olfactory Epithelium	24	3.00325	3.08126	0.07801	94956	94931	6,047.15	7,488.04
Respiratory Epithelium	25	3.01720	3.04889	0.03169	3971111	3971086	622,701.81	771,076.57
Axillary Nodes (2)	26	2.96689	2.99796	0.03107	118	93	14.87	18.42
Muscle (R, deltoid)	27	2.95544	2.98076	0.02532	63	38	7.46	9.23
Liver (R, superficial lobe)	28	2.97190	3.00280	0.03090	101	76	12.22	15.13
Kidney (L, tip)	29	2.98662	3.01919	0.03257	226	201	30.67	37.97
Lung (R, top lobe)	30	2.97276	2.99119	0.01843	125	100	26.96	33.39
Trachea (near cut-off)	31	3.06223	3.07554	0.01331	59	34	12.69	15.72
Esophagus (near cut-off)	32	2.99967	3.01161	0.01194	50	25	10.40	12.88
Spleen (tip)	33	2.97510	3.01128	0.03618	111	86	11.81	14.63
Heart	34	2.99119	3.02202	0.03083	125	100	16.12	19.96
Urine (fill to line of tube)	35	2.97076	3.23913	0.26837	664	639	11.83	14.65
Spinal Dura	36	2.97020	2.98202	0.01182	67	42	17.66	21.86
Lower Cervical Spinal Cord	37	3.00247	3.12990	0.12743	115	90	3.51	4.35
Thoracic Spinal Cord	38	2.98200	3.07002	0.08802	90	65	3.67	4.54
Lumbar Spinal Cord	39	2.97788	3.12039	0.14251	131	106	3.70	4.58
Blood Sample	40	2.99370	3.28305	0.28935	2609	2584	44.38	54.95
Drug Standard	41	2.99100	2.99392	0.00292	3930989	3930964	6,689,740.82	8,283,744.02
Drug Standard	42	3.02776	3.03062	0.00286	3977841	3977816	6,911,490.80	8,558,331.65
Drug Standard	43	2.98891	2.99133	0.00242	3912295	3912270	8,033,532.05	9,947,728.15
							0.201 cpm/fmol	0.163 cpm/fmol

Auto Calculations		Enter Values	
Total uL of Dose Sol =	63.1	Oxytocin Molecular Weight	1,007
Hot mg =	0.0000	Date of Synthesis =	10/03/07
Cold mg =	0.5139	Hot uCi/uL at Synthesis =	**2.300**
Total mg in Dose Sol =	0.5140	Hot uCi/ug at Synthesis =	**1985.72**
Conc Of Dose Sol (ug/uL) =	8.1402	Cold ug/uL =	15.00
Days after Synthesis Delivered =	12	Desired uCi =	45.0
Decay on Day Delivered =	0.8706	Desired mg =	0.400
Total uCi in Dose Sol on Day Delivered =	57.82	Estimated Desired nmol =	397.1
uCi/uL in Dose Sol on Day Delivered =	0.9158	**Date of Delivery =**	**10/15/07**
mg delivered =	0.391	**uL delivered (actual) =**	**48.0**
uCi delivered =	43.96	**Date of Counting =**	**10/15/07**
Estimated nmol delivered =	387.94	**Dose Calculator**	
Days after Synthesis Counted =	12	uL Hot to Deliver =	22.47
Decay on Day Counted =	0.8706	uL Cold to Deliver =	26.66
Specific Activity from Amersham Specs (cpm/fmol) =	0.201	Total uL to Deliver =	49.14
Avg. Volume of Standard (uL)* =	3.0	Hot to Cold Ratio =	0.84
Avg. CPM-bkgd of Standard =	3,940,350	Total uL to Make =	63.14
Specific Activity from Standards: (CPM/fmol) =	0.163	Dose Solution uL Hot =	28.88
Date of Disposal from Decay Storage =	05/25/09	Dose Solution uL Cold =	34.26

Table A05: Intranasal Oxytocin Rat 5 Data

Oxytocin Rat 5	Route	Com-pound	Volume (uL)	uCi	mg	Time (min)	Perfused @ (min)
Amount Delivered:	IN	Oxytocin	49	45.00	0.400	14:02:00	34:00:00

Tissue Description	Tube #	Tube (g)	Tissue + Tube (g)	Tissue Weight (g)	CPM	CPM -25 bkgd	nM (Spec. Act.)	nM (Stan.)
Dorsal Dura	1	3.07509	3.08809	0.01300	1352	1327	507.25	628.11
Ventral Dura	2	3.03766	3.04844	0.01078	704	679	313.00	387.58
Trigeminal Ganglion	3	2.98708	3.00927	0.02219	4236	4211	943.02	1,167.72
Maxillary Nerve	4	3.00777	3.01839	0.01062	2935	2910	1,361.64	1,686.08
Opthalmic Nerve	5	2.99020	2.99642	0.00622	391	366	292.40	362.08
Mandibular Nerve	6	2.99419	2.99910	0.00491	884	859	869.37	1,076.52
Upper Cervical Spinal Cord	7	3.00853	3.05267	0.04414	494	469	52.80	65.38
Olfactory Bulbs	8	2.98361	3.06209	0.07848	882	857	54.26	6.72
Anterior Olfactory Nucleus	9	2.98357	3.01825	0.03468	207	182	26.08	32.29
Frontal Cortex	10	3.03825	3.07622	0.03797	187	162	21.20	26.25
Caudate/Putamen	11	3.01235	3.02010	0.00775	115	90	57.71	71.46
Septal Nucleus	12	3.01369	3.02448	0.01079	72	47	21.65	26.80
Parietal Cortex	13	2.98705	3.00070	0.01365	106	81	29.49	36.51
Hippocampus	14	2.96960	3.01084	0.04124	97	72	8.68	10.74
Thalamus	15	2.99412	3.07537	0.08125	180	155	9.48	11.74
Hypothalamus	16	3.03802	3.06832	0.03030	209	184	30.18	37.37
Midbrain	17	3.04764	3.17636	0.12872	422	397	15.33	18.98
Pons	18	3.03169	3.10153	0.06984	296	271	19.28	23.88
Medulla	19	3.00957	3.09885	0.08928	343	318	17.70	21.92
Cerebellum	20	3.00946	3.25237	0.24291	997	972	19.88	24.62
Superficial Nodes (2)	21	3.00817	3.08889	0.08072	8810	8785	540.82	669.69
Cervical Nodes (2)	22	2.99429	3.00869	0.01440	320	295	101.80	126.06
Thyroid	23	2.99876	3.01918	0.02042	1586	1561	379.87	470.39
Olfactory Epithelium	24	2.99339	3.06521	0.07182	135533	135508	9,375.89	11,609.94
Respiratory Epithelium	25	3.04354	3.09709	0.05355	8076472	8076447	749,469.48	928,049.90
Axillary Nodes (2)	26	2.98645	3.01369	0.02724	135	110	20.07	24.85
Muscle (R, deltoid)	27	2.96714	2.99128	0.02414	83	58	11.94	14.78
Liver (R, superficial lobe)	28	2.97421	3.00503	0.03082	178	153	24.67	30.55
Kidney (L, tip)	29	2.98985	3.02860	0.03875	318	293	37.57	46.53
Lung (R, top lobe)	30	2.97733	3.01025	0.03292	114	89	13.43	16.64
Trachea (near cut-off)	31	2.98949	3.00806	0.01857	147	122	32.65	40.43
Esophagus (near cut-off)	32	3.05999	3.07597	0.01598	161	136	42.29	52.37
Spleen (tip)	33	3.01004	3.04714	0.03710	259	234	31.34	38.81
Heart	34	3.02561	3.04464	0.01903	93	68	17.76	21.99
Urine (fill to line of tube)	35	2.95460	3.19151	0.23691	1332	1307	27.41	33.95
Spinal Dura	36	2.97607	2.99215	0.01608	76	51	15.76	19.52
Lower Cervical Spinal Cord	37	2.98224	3.10932	0.12708	116	91	3.56	4.41
Thoracic Spinal Cord	38	2.99307	3.08509	0.09202	122	97	5.24	6.49
Lumbar Spinal Cord	39	2.97664	3.10756	0.13092	123	98	3.72	4.61
Blood Sample	40	2.98491	3.23403	0.24912	3796	3771	75.22	93.14
Drug Standard	41	2.99100	2.99392	0.00292	3930989	3930964	6,689,740.82	8,283,744.02
Drug Standard	42	3.02776	3.03062	0.00286	3977841	3977816	6,911,490.80	8,558,331.65
Drug Standard	43	2.98891	2.99133	0.00242	3912295	3912270	8,033,532.05	9,947,728.15
							0.201 cpm/fmol	0.163 cpm/fmol

Auto Calculations		Enter Values	
Total uL of Dose Sol =	63.1	Oxytocin Molecular Weight	1,007
Hot mg =	0.0000	Date of Synthesis =	10/03/07
Cold mg =	0.5139	Hot uCi/uL at Synthesis =	**2.300**
Total mg in Dose Sol =	0.5140	Hot uCi/ug at Synthesis =	**1985.72**
Conc Of Dose Sol (ug/uL) =	8.1402	Cold ug/uL =	15.00
Days after Synthesis Delivered =	12	Desired uCi =	45.0
Decay on Day Delivered =	0.8706	Desired mg =	0.400
Total uCi in Dose Sol on Day Delivered =	57.82	Estimated Desired nmol =	397.1
uCi/uL in Dose Sol on Day Delivered =	0.9158	**Date of Delivery =**	**10/15/07**
mg delivered =	0.400	**uL delivered (actual) =**	**49.1**
uCi delivered =	45.00	**Date of Counting =**	**10/15/07**
Estimated nmol delivered =	397.15	**Dose Calculator**	
Days after Synthesis Counted =	12	uL Hot to Deliver =	22.47
Decay on Day Counted =	0.8706	uL Cold to Deliver =	26.66
Specific Activity from Amersham Specs (cpm/fmol) =	0.201	Total uL to Deliver =	49.14
Avg. Volume of Standard (uL)* =	3.0	Hot to Cold Ratio =	0.84
Avg. CPM-bkgd of Standard =	3,940,350	Total uL to Make =	63.14
Specific Activity from Standards: (CPM/fmol) =	0.163	Dose Solution uL Hot =	28.88
Date of Disposal from Decay Storage =	05/25/09	Dose Solution uL Cold =	34.26

Table A06: Intranasal Oxytocin Rat 6 Data

Oxytocin Rat 6	Route	Com-pound	Volume (uL)	uCi	mg	Time (min)	Perfused @ (min)
Amount Delivered:	IN	Oxytocin	49	45.00	0.400	14:03:00	30:57:00

Tissue Description	Tube #	Tube (g)	Tissue + Tube (g)	Tissue Weight (g)	CPM	CPM -25 bkgd	nM (Spec. Act.)	nM (Stan.)
Dorsal Dura	1	2.98734	2.99610	0.00876	3080	3055	1,733.01	2,158.63
Ventral Dura	2	3.00782	3.01438	0.00656	370	345	261.34	325.53
Trigeminal Ganglion	3	2.99584	3.02547	0.02963	1718	1693	283.93	353.67
Maxillary Nerve	4	2.99307	3.00970	0.01663	2063	2038	608.98	758.55
Opthalmic Nerve	5	2.99163	2.99614	0.00451	120	95	104.67	130.38
Mandibular Nerve	6	3.00305	3.00777	0.00472	277	252	265.31	330.47
Upper Cervical Spinal Cord	7	3.01224	3.06331	0.05107	315	290	28.22	35.15
Olfactory Bulbs	8	2.99614	3.07578	0.07964	512	487	30.39	37.85
Anterior Olfactory Nucleus	9	3.01838	3.05841	0.04003	143	118	14.65	18.25
Frontal Cortex	10	2.99642	3.03442	0.03800	135	110	14.38	17.92
Caudate/Putamen	11	2.98825	3.00107	0.01282	81	56	21.71	27.04
Septal Nucleus	12	2.99189	3.00573	0.01384	90	65	23.34	29.07
Parietal Cortex	13	2.99225	3.02763	0.03538	96	71	9.97	12.42
Hippocampus	14	3.00071	3.04819	0.04748	147	122	12.77	15.90
Thalamus	15	3.02225	3.10617	0.08392	151	126	7.46	9.29
Hypothalamus	16	3.01289	3.03193	0.01904	97	72	18.79	23.41
Midbrain	17	3.07539	3.13991	0.06452	149	124	9.55	11.90
Pons	18	2.99996	3.06981	0.06985	175	150	10.67	13.29
Medulla	19	3.01481	3.12522	0.11041	247	222	9.99	12.45
Cerebellum	20	2.99336	3.25738	0.26402	540	515	9.69	12.07
Superficial Nodes (2)	21	2.99211	3.05619	0.06408	6101	6076	471.18	586.90
Cervical Nodes (2)	22	3.04851	3.05885	0.01034	1486	1461	702.14	874.58
Thyroid	23	2.99260	3.01216	0.01956	1575	1550	393.78	490.49
Olfactory Epithelium	24	2.99359	3.08248	0.08889	650043	650018	36,338.38	45,262.93
Respiratory Epithelium	25	2.99536	3.02581	0.03045	4797645	4797620	782,945.67	975,233.73
Axillary Nodes (2)	26	2.98853	3.02302	0.03449	123	98	14.12	17.59
Muscle (R, deltoid)	27	3.02203	3.04927	0.02724	98	73	13.32	16.59
Liver (R, superficial lobe)	28	2.97675	3.00588	0.02913	131	106	18.08	22.52
Kidney (L, tip)	29	2.97149	3.00577	0.03428	378	353	51.17	63.74
Lung (R, top lobe)	30	2.98457	3.00713	0.02256	94	69	15.20	18.93
Trachea (near cut-off)	31	2.98926	3.00428	0.01502	100	75	24.81	30.91
Esophagus (near cut-off)	32	2.99267	3.00597	0.01330	89	64	23.91	29.79
Spleen (tip)	33	2.98918	3.03013	0.04095	268	243	29.49	36.73
Heart	34	2.98141	3.01849	0.03708	122	97	13.00	16.19
Urine (fill to line of tube)	35	2.99221	3.24676	0.25455	5016	4991	97.43	121.36
Spinal Dura	36	2.97308	2.98449	0.01141	50	25	10.89	13.56
Lower Cervical Spinal Cord	37	2.99126	3.12149	0.13023	129	104	3.97	4.94
Thoracic Spinal Cord	38	2.97687	3.05706	0.08019	93	68	4.21	5.25
Lumbar Spinal Cord	39	2.95765	3.09079	0.13314	112	87	3.25	4.04
Blood Sample	40	3.02175	3.27900	0.25725	3516	3491	67.44	84.00
Drug Standard	41	2.99942	3.00244	0.00302	3829733	3829708	6,301,613.98	7,849,263.04
Drug Standard	42	3.03916	3.04220	0.00304	4053669	4053644	6,626,208.53	8,253,576.59
Drug Standard	43	2.99521	2.99781	0.00260	3806153	3806128	7,274,499.51	9,061,085.02
							0.201 cpm/fmol	0.162 cpm/fmol

Auto Calculations		Enter Values	
Total uL of Dose Sol =	63.4	Oxytocin Molecular Weight	1,007
Hot mg =	0.0000	Date of Synthesis =	10/03/07
Cold mg =	0.5133	Hot uCi/uL at Synthesis =	**2.300**
Total mg in Dose Sol =	0.5134	Hot uCi/ug at Synthesis =	**1985.72**
Conc Of Dose Sol (ug/uL) =	8.0972	Cold ug/uL =	15.00
Days after Synthesis Delivered =	13	Desired uCi =	45.0
Decay on Day Delivered =	0.8606	Desired mg =	0.400
Total uCi in Dose Sol on Day Delivered =	57.75	Estimated Desired nmol =	397.1
uCi/uL in Dose Sol on Day Delivered =	0.9109	**Date of Delivery =**	**10/16/07**
mg delivered =	0.400	**uL delivered (actual) =**	**49.4**
uCi delivered =	45.00	**Date of Counting =**	**10/16/07**
Estimated nmol delivered =	397.15	**Dose Calculator**	
Days after Synthesis Counted =	13	uL Hot to Deliver =	22.73
Decay on Day Counted =	0.8606	uL Cold to Deliver =	26.66
Specific Activity from Amersham Specs (cpm/fmol) =	0.201	Total uL to Deliver =	49.40
Avg. Volume of Standard (uL)* =	3.0	Hot to Cold Ratio =	0.85
Avg. CPM-bkgd of Standard =	3,896,493	Total uL to Make =	63.40
Specific Activity from Standards: (CPM/fmol) =	0.162	Dose Solution uL Hot =	29.18
Date of Disposal from Decay Storage =	05/25/09	Dose Solution uL Cold =	34.22

Table A07: Intranasal Oxytocin Rat 7 Data

Oxytocin Rat 7	Route	Com-pound	Volume (uL)	uCi	mg	Time (min)	Perfused @ (min)
Amount Delivered:	IN	Oxytocin	49	45.00	0.400	14:02:00	30:55:00

Tissue Description	Tube #	Tube (g)	Tissue + Tube (g)	Tissue Weight (g)	CPM	CPM -25 bkgd	nM (Spec. Act.)	nM (Stan.)
Dorsal Dura	1	3.00143	3.01445	0.01302	743	718	274.04	341.34
Ventral Dura	2	3.03889	3.05308	0.01419	2457	2432	851.68	1,060.84
Trigeminal Ganglion	3	3.01833	3.04813	0.02980	1394	1369	228.29	284.35
Maxillary Nerve	4	3.00502	3.01543	0.01041	613	588	280.69	349.62
Opthalmic Nerve	5	3.00414	3.01066	0.00652	588	563	429.10	534.48
Mandibular Nerve	6	3.04754	3.05020	0.00266	546	521	973.31	1,212.35
Upper Cervical Spinal Cord	7	3.07199	3.13451	0.06252	2787	2762	219.53	273.45
Olfactory Bulbs	8	3.01556	3.08649	0.07093	1092	1067	74.75	93.11
Anterior Olfactory Nucleus	9	2.98920	3.03208	0.04288	294	269	31.17	38.83
Frontal Cortex	10	2.99824	3.03626	0.03802	270	245	32.02	39.89
Caudate/Putamen	11	2.96956	2.98203	0.01247	243	218	86.87	108.21
Septal Nucleus	12	3.08244	3.09405	0.01161	141	116	49.65	61.84
Parietal Cortex	13	3.00368	3.03517	0.03149	353	328	51.76	64.47
Hippocampus	14	3.00483	3.03660	0.03177	184	159	24.87	30.98
Thalamus	15	2.98935	3.07033	0.08098	136	111	6.81	8.48
Hypothalamus	16	3.00812	3.03310	0.02498	144	119	23.67	29.49
Midbrain	17	2.99067	3.10912	0.11845	278	253	10.61	13.22
Pons	18	3.00421	3.06275	0.05854	262	237	20.12	25.06
Medulla	19	3.03855	3.13103	0.09248	670	645	34.66	43.17
Cerebellum	20	2.99897	3.24394	0.24497	703	678	13.75	17.13
Superficial Nodes (2)	21	2.99014	3.07046	0.08032	11714	11689	723.18	900.79
Cervical Nodes (2)	22	3.01158	3.02908	0.01750	1800	1775	504.03	627.81
Thyroid	23	2.98746	3.01921	0.03175	1184	1159	181.40	225.95
Olfactory Epithelium	24	3.00199	3.13221	0.13022	1846303	1846278	70,455.11	87,758.58
Respiratory Epithelium	25	2.99435	3.06898	0.07463	5109929	5109904	340,245.49	423,808.31
Axillary Nodes (2)	26	2.98232	3.00642	0.02410	108	83	17.11	21.32
Muscle (R, deltoid)	27	2.98155	3.00821	0.02666	72	47	8.76	10.91
Liver (R, superficial lobe)	28	2.99971	3.03170	0.03199	114	89	13.83	17.22
Kidney (L, tip)	29	2.97996	3.01906	0.03910	326	301	38.25	47.65
Lung (R, top lobe)	30	2.97656	3.00601	0.02945	183	158	26.66	33.21
Trachea (near cut-off)	31	2.99017	3.00413	0.01396	94	69	24.56	30.59
Esophagus (near cut-off)	32	2.98646	3.00280	0.01634	81	56	17.03	21.21
Spleen (tip)	33	3.02206	3.06818	0.04612	170	145	15.62	19.46
Heart	34	2.99751	3.03415	0.03664	130	105	14.24	17.74
Urine (fill to line of tube)	35	2.97282	3.14263	0.16981	672	647	18.93	23.58
Spinal Dura	36	2.98490	2.99785	0.01295	83	58	22.26	27.72
Lower Cervical Spinal Cord	37	3.06180	3.17139	0.10959	109	84	3.81	4.74
Thoracic Spinal Cord	38	3.03043	3.11497	0.08454	213	188	11.05	13.76
Lumbar Spinal Cord	39	2.99156	3.13669	0.14513	904	879	30.10	37.49
Blood Sample	40	2.98187	3.23717	0.25530	2320	2295	44.67	55.64
Drug Standard	41	2.99942	3.00244	0.00302	3829733	3829708	6,301,613.98	7,849,263.04
Drug Standard	42	3.03916	3.04220	0.00304	4053669	4053644	6,626,208.53	8,253,576.59
Drug Standard	43	2.99521	2.99781	0.00260	3806153	3806128	7,274,499.51	9,061,085.02
							0.201 cpm/fmol	0.162 cpm/fmol

Auto Calculations		Enter Values	
Total uL of Dose Sol =	63.4	Oxytocin Molecular Weight	1,007
Hot mg =	0.0000	Date of Synthesis =	10/03/07
Cold mg =	0.5133	Hot uCi/uL at Synthesis =	**2.300**
Total mg in Dose Sol =	0.5134	Hot uCi/ug at Synthesis =	**1985.72**
Conc Of Dose Sol (ug/uL) =	8.0972	Cold ug/uL =	15.00
Days after Synthesis Delivered =	13	Desired uCi =	45.0
Decay on Day Delivered =	0.8606	Desired mg =	0.400
Total uCi in Dose Sol on Day Delivered =	57.75	Estimated Desired nmol =	397.1
uCi/uL in Dose Sol on Day Delivered =	0.9109	**Date of Delivery =**	**10/16/07**
mg delivered =	0.400	**uL delivered (actual) =**	**49.4**
uCi delivered =	45.00	**Date of Counting =**	**10/16/07**
Estimated nmol delivered =	397.15	**Dose Calculator**	
Days after Synthesis Counted =	13	uL Hot to Deliver =	22.73
Decay on Day Counted =	0.8606	uL Cold to Deliver =	26.66
Specific Activity from Amersham Specs (cpm/fmol) =	0.201	Total uL to Deliver =	49.40
Avg. Volume of Standard (uL)* =	3.0	Hot to Cold Ratio =	0.85
Avg. CPM-bkgd of Standard =	3,896,493	Total uL to Make =	63.40
Specific Activity from Standards: (CPM/fmol) =	0.162	Dose Solution uL Hot =	29.18
Date of Disposal from Decay Storage =	05/25/09	Dose Solution uL Cold =	34.22

Table A08: Intranasal Oxytocin Rat 8 Data

Oxytocin Rat 8	Route	Com-pound	Volume (uL)	uCi	mg	Time (min)	Perfused @ (min)
Amount Delivered:	IN	Oxytocin	50	45.00	0.400	14:01	31:06

Tissue Description	Tube #	Tube (g)	Tissue + Tube (g)	Tissue Weight (g)	CPM	CPM -25 bkgd	nM (Spec. Act.)	nM (Stan.)
Dorsal Dura	1	2.99634	3.00552	0.00918	436	411	222.48	268.27
Ventral Dura	2	3.00873	3.01637	0.00764	325	300	195.13	235.29
Trigeminal Ganglion	3	2.99056	3.01996	0.02940	466	441	74.54	89.88
Maxillary Nerve	4	2.99543	3.01055	0.01512	362	337	110.76	133.55
Opthalmic Nerve	5	3.02109	3.02472	0.00363	12199	12174	16,665.55	20,095.53
Mandibular Nerve	6	3.03825	3.04355	0.00530	1204	1179	1,105.43	1,332.94
Upper Cervical Spinal Cord	7	3.00577	3.08156	0.07579	841	816	53.50	64.51
Olfactory Bulbs	8	3.01048	3.08696	0.07648	11856	11831	768.72	926.93
Anterior Olfactory Nucleus	9	2.99541	3.02679	0.03138	522	497	78.70	94.90
Frontal Cortex	10	2.98194	3.02436	0.04242	2308	2283	267.44	322.48
Caudate/Putamen	11	2.98004	2.99271	0.01267	57	32	12.55	15.13
Septal Nucleus	12	2.98957	3.00425	0.01468	91	66	22.34	26.94
Parietal Cortex	13	2.98908	3.02036	0.03128	308	283	44.96	54.21
Hippocampus	14	3.08156	3.12236	0.04080	219	194	23.63	28.49
Thalamus	15	3.00938	3.09301	0.08363	847	822	48.84	58.90
Hypothalamus	16	3.08230	3.10521	0.02291	2779	2754	597.35	720.30
Midbrain	17	2.99078	3.07745	0.08667	1536	1511	86.63	104.46
Pons	18	3.08441	3.13994	0.05553	951	926	82.87	99.92
Medulla	19	2.96886	3.06608	0.09722	1480	1455	74.37	89.68
Cerebellum	20	2.99633	3.25071	0.25438	3318	3293	64.33	77.57
Superficial Nodes (2)	21	3.01327	3.07715	0.06388	1084	1059	82.38	99.34
Cervical Nodes (2)	22	3.01064	3.02379	0.01315	374	349	131.88	159.03
Thyroid	23	3.01364	3.03599	0.02235	1667	1642	365.08	440.22
Olfactory Epithelium	24	2.99497	3.12088	0.12591	85308	85283	3,365.85	4,058.59
Respiratory Epithelium	25	3.04287	3.11032	0.06745	2292664	2292639	168,906.67	203,669.72
Axillary Nodes (2)	26	2.97735	3.02017	0.04282	328	303	35.16	42.40
Muscle (R, deltoid)	27	2.97159	3.02376	0.05217	217	192	18.29	22.05
Liver (R, superficial lobe)	28	2.99487	3.03949	0.04462	157	132	14.70	17.73
Kidney (L, tip)	29	3.00151	3.05144	0.04993	517	492	48.97	59.04
Lung (R, top lobe)	30	2.98258	3.02426	0.04168	167	142	16.93	20.41
Trachea (near cut-off)	31	2.98989	2.99930	0.00941	107	82	43.30	52.22
Esophagus (near cut-off)	32	2.96658	2.98238	0.01580	106	81	25.48	30.72
Spleen (tip)	33	2.99562	3.03197	0.03635	197	172	23.51	28.35
Heart	34	2.97068	3.00420	0.03352	185	160	23.72	28.60
Urine (fill to line of tube)	35	2.98206	3.29450	0.31244	5066	5041	80.18	96.68
Spinal Dura	36	3.06313	3.07664	0.01351	129	104	38.25	46.13
Lower Cervical Spinal Cord	37	2.97303	3.08969	0.11666	121	96	4.09	4.93
Thoracic Spinal Cord	38	2.99690	3.07914	0.08224	118	93	5.62	6.78
Lumbar Spinal Cord	39	2.98986	3.14437	0.15451	464	439	14.12	17.02
Blood Sample	40	3.00277	3.25763	0.25486	2688	2663	51.92	62.61
Drug Standard	41	3.02032	3.02322	0.00290	4076833	4076808	6,985,786.97	8,423,546.94
Drug Standard	42	2.99939	3.00188	0.00249	3860255	3860230	7,703,833.92	9,289,376.69
Drug Standard	43	2.99850	3.00167	0.00317	4009664	4009639	6,285,489.26	7,579,119.43
							0.201 cpm/fmol	0.167 cpm/fmol

Auto Calculations	
Total uL of Dose Sol =	63.9
Hot mg =	0.0000
Cold mg =	0.5121
Total mg in Dose Sol =	0.5122
Conc Of Dose Sol (ug/uL) =	8.0110
Days after Synthesis Delivered =	15
Decay on Day Delivered =	0.8409
Total uCi in Dose Sol on Day Delivered =	57.62
uCi/uL in Dose Sol on Day Delivered =	0.9012
mg delivered =	0.400
uCi delivered =	45.00
Estimated nmol delivered =	397.14
Days after Synthesis Counted =	15
Decay on Day Counted =	0.8409
Specific Activity from Amersham Specs (cpm/fmol) =	0.201
Avg. Volume of Standard (uL)* =	3.0
Avg. CPM-bkgd of Standard =	3,982,226
Specific Activity from Standards: (CPM/fmol) =	0.167
Date of Disposal from Decay Storage =	05/25/09

Enter Values	
Oxytocin Molecular Weight	1,007
Date of Synthesis =	10/03/07
Hot uCi/uL at Synthesis =	**2.300**
Hot uCi/ug at Synthesis =	**1985.72**
Cold ug/uL =	15.00
Desired uCi =	45.0
Desired mg =	0.400
Estimated Desired nmol =	397.1
**Date of Delivery =**	**10/18/07**
**uL delivered (actual) =**	**49.9**
**Date of Counting =**	**10/18/07**
**Dose Calculator**	
uL Hot to Deliver =	23.27
uL Cold to Deliver =	26.66
Total uL to Deliver =	49.93
Hot to Cold Ratio =	0.87
Total uL to Make =	63.93
Dose Solution uL Hot =	29.79
Dose Solution uL Cold =	34.14

Table A09: Intranasal Oxytocin Rat 9 Data

Oxytocin Rat 9	Route	Com-pound	Volume (uL)	uCi	mg	Time (min)	Perfused @ (min)
Amount Delivered:	IN	Oxytocin	50	45.00	0.400	14:12	30:57

Tissue Description	Tube #	Tube (g)	Tissue + Tube (g)	Tissue Weight (g)	CPM	CPM -25 bkgd	nM (Spec. Act.)	nM (Stan.)
Dorsal Dura	1	2.98629	3.00004	0.01375	158	133	48.07	57.96
Ventral Dura	2	3.02130	3.03352	0.01222	130	105	42.70	51.49
Trigeminal Ganglion	3	3.00034	3.03392	0.03358	8863	8838	1,307.88	1,577.05
Maxillary Nerve	4	2.99655	3.00813	0.01158	2743	2718	1,166.36	1,406.42
Opthalmic Nerve	5	2.99334	2.99913	0.00579	2227	2202	1,889.87	2,278.83
Mandibular Nerve	6	2.99843	3.00153	0.00310	6557	6532	10,470.74	12,625.75
Upper Cervical Spinal Cord	7	2.99071	3.04418	0.05347	199	174	16.17	19.50
Olfactory Bulbs	8	2.97611	3.05088	0.07477	6795	6770	449.94	542.54
Anterior Olfactory Nucleus	9	2.98277	3.02543	0.04266	350	325	37.86	45.65
Frontal Cortex	10	2.99432	3.03468	0.04036	504	479	58.98	71.11
Caudate/Putamen	11	2.98398	2.99660	0.01262	80	55	21.66	26.11
Septal Nucleus	12	2.98162	2.99741	0.01579	66	41	12.90	15.56
Parietal Cortex	13	2.98879	3.02155	0.03276	199	174	26.39	31.83
Hippocampus	14	3.00336	3.04384	0.04048	88	63	7.73	9.33
Thalamus	15	2.97111	3.05846	0.08735	122	97	5.52	6.65
Hypothalamus	16	2.99297	3.01736	0.02439	119	94	19.15	23.09
Midbrain	17	2.97027	3.04218	0.07191	131	106	7.33	8.83
Pons	18	3.01895	3.08126	0.06231	122	97	7.74	9.33
Medulla	19	3.00848	3.11369	0.10521	182	157	7.42	8.94
Cerebellum	20	3.01957	3.26432	0.24475	385	360	7.31	8.81
Superficial Nodes (2)	21	3.07405	3.13568	0.06163	1639	1614	130.14	156.92
Cervical Nodes (2)	22	2.99077	3.00268	0.01191	610	585	244.08	294.32
Thyroid	23	2.99649	3.01941	0.02292	1070	1045	226.57	273.20
Olfactory Epithelium	24	2.99889	3.07728	0.07839	31411	31386	1,989.61	2,399.10
Respiratory Epithelium	25	3.06697	3.11579	0.04882	1469425	1469400	149,566.85	180,349.52
Axillary Nodes (2)	26	2.98323	3.01603	0.03280	156	131	19.85	23.93
Muscle (R, deltoid)	27	2.97637	3.01619	0.03982	150	125	15.60	18.81
Liver (R, superficial lobe)	28	2.99565	3.04254	0.04689	178	153	16.21	19.55
Kidney (L, tip)	29	2.95465	3.00396	0.04931	234	209	21.06	25.40
Lung (R, top lobe)	30	2.99799	3.04333	0.04534	114	89	9.75	11.76
Trachea (near cut-off)	31	2.96052	2.97460	0.01408	76	51	18.00	21.70
Esophagus (near cut-off)	32	2.96476	2.97988	0.01512	79	54	17.75	21.40
Spleen (tip)	33	2.97935	3.01854	0.03919	185	160	20.29	24.46
Heart	34	2.98520	3.01892	0.03372	153	128	18.86	22.75
Urine (fill to line of tube)	35	2.99084	3.19256	0.20172	1562	1537	37.86	45.66
Spinal Dura	36	2.97368	2.98449	0.01081	254	229	105.27	126.94
Lower Cervical Spinal Cord	37	2.99842	3.13042	0.13200	121	96	3.61	4.36
Thoracic Spinal Cord	38	2.97885	3.06057	0.08172	168	143	8.70	10.49
Lumbar Spinal Cord	39	3.00880	3.13016	0.12136	220	195	7.98	9.63
Blood Sample	40	2.98111	3.25589	0.27478	1686	1661	30.04	36.22
Drug Standard	41	3.02032	3.02322	0.00290	4076833	4076808	6,985,786.97	8,423,546.94
Drug Standard	42	2.99939	3.00188	0.00249	3860255	3860230	7,703,833.92	9,289,376.69
Drug Standard	43	2.99850	3.00167	0.00317	4009664	4009639	6,285,489.26	7,579,119.43
							0.201 cpm/fmol	0.167 cpm/fmol

Auto Calculations		Enter Values	
Total uL of Dose Sol =	63.9	Oxytocin Molecular Weight	1,007
Hot mg =	0.0000	Date of Synthesis =	10/03/07
Cold mg =	0.5121	Hot uCi/uL at Synthesis =	**2.300**
Total mg in Dose Sol =	0.5122	Hot uCi/ug at Synthesis =	**1985.72**
Conc Of Dose Sol (ug/uL) =	8.0110	Cold ug/uL =	15.00
Days after Synthesis Delivered =	15	Desired uCi =	45.0
Decay on Day Delivered =	0.8409	Desired mg =	0.400
Total uCi in Dose Sol on Day Delivered =	57.62	Estimated Desired nmol =	397.1
uCi/uL in Dose Sol on Day Delivered =	0.9012	**Date of Delivery =**	**10/18/07**
mg delivered =	0.400	**uL delivered (actual) =**	**49.9**
uCi delivered =	45.00	**Date of Counting =**	**10/18/07**
Estimated nmol delivered =	397.14	**Dose Calculator**	
Days after Synthesis Counted =	15	uL Hot to Deliver =	23.27
Decay on Day Counted =	0.8409	uL Cold to Deliver =	26.66
Specific Activity from Amersham Specs (cpm/fmol) =	0.201	Total uL to Deliver =	49.93
Avg. Volume of Standard (uL)* =	3.0	Hot to Cold Ratio =	0.87
Avg. CPM-bkgd of Standard =	3,982,226	Total uL to Make =	63.93
Specific Activity from Standards: (CPM/fmol) =	0.167	Dose Solution uL Hot =	29.79
Date of Disposal from Decay Storage =	05/25/09	Dose Solution uL Cold =	34.14

Table A10: Intranasal Oxytocin Rat 10 Data

Oxytocin Rat 10	Route	Com-pound	Volume (uL)	uCi	mg	Time (min)	Perfused @ (min)
Amount Delivered:	IN	Oxytocin	51	44.97	0.400	14:01	30:56

Tissue Description	Tube #	Tube (g)	Tissue + Tube (g)	Tissue Weight (g)	CPM	CPM -25 bkgd	nM (Spec. Act.)	nM (Stan.)
Dorsal Dura	1	3.01729	3.03344	0.01615	469	444	136.62	167.95
Ventral Dura	2	2.98284	2.99166	0.00882	221	196	110.43	135.75
Trigeminal Ganglion	3	-	-	-	-	-	-	-
Maxillary Nerve	4	2.99591	3.00541	0.00950	206	181	94.68	116.39
Opthalmic Nerve	5	3.04605	3.04763	0.00158	37	12	37.74	46.40
Mandibular Nerve	6	3.00484	3.00802	0.00318	89	64	100.01	122.95
Upper Cervical Spinal Cord	7	-	-	-	-	-	-	-
Olfactory Bulbs	8	-	-	-	-	-	-	-
Anterior Olfactory Nucleus	9	-	-	-	-	-	-	-
Frontal Cortex	10	-	-	-	-	-	-	-
Caudate/Putamen	11	-	-	-	-	-	-	-
Septal Nucleus	12	-	-	-	-	-	-	-
Parietal Cortex	13	-	-	-	-	-	-	-
Hippocampus	14	-	-	-	-	-	-	-
Thalamus	15	-	-	-	-	-	-	-
Hypothalamus	16	-	-	-	-	-	-	-
Midbrain	17	-	-	-	-	-	-	-
Pons	18	-	-	-	-	-	-	-
Medulla	19	-	-	-	-	-	-	-
Cerebellum	20	-	-	-	-	-	-	-
Superficial Nodes (2)	21	2.98388	3.05466	0.07078	3880	3855	270.65	332.72
Cervical Nodes (2)	22	2.98960	2.99183	0.00223	65	40	89.13	109.58
Thyroid	23	2.97968	2.99319	0.01351	820	795	292.42	359.48
Olfactory Epithelium	24	2.98784	3.07676	0.08892	54021	53996	3,017.56	3,709.62
Respiratory Epithelium	25	3.03051	3.08806	0.05755	10404574	10404549	898,402.62	1,104,447.13
Axillary Nodes (2)	26	2.96778	3.00651	0.03873	101	76	9.75	11.99
Muscle (R, deltoid)	27	2.96092	3.00135	0.04043	74	49	6.02	7.40
Liver (R, superficial lobe)	28	2.96079	3.00101	0.04022	70	45	5.56	6.83
Kidney (L, tip)	29	2.96566	2.99818	0.03252	223	198	30.26	37.19
Lung (R, top lobe)	30	3.00739	3.04567	0.03828	191	166	21.55	26.49
Trachea (near cut-off)	31	2.97595	2.99284	0.01689	68	43	12.65	15.55
Esophagus (near cut-off)	32	2.99180	3.00462	0.01282	53	28	10.85	13.34
Spleen (tip)	33	2.96780	3.01686	0.04906	132	107	10.84	13.32
Heart	34	2.97433	3.01919	0.04486	122	97	10.74	13.21
Urine (fill to line of tube)	35	2.99751	3.28215	0.28464	6259	6234	108.83	133.79
Spinal Dura	36	2.99599	3.01124	0.01525	60	35	11.40	14.02
Lower Cervical Spinal Cord	37	3.02594	3.19956	0.17362	127	102	2.92	3.59
Thoracic Spinal Cord	38	2.95332	3.03702	0.08370	65	40	2.37	2.92
Lumbar Spinal Cord	39	3.00661	3.16945	0.16284	108	83	2.53	3.11
Blood Sample	40	2.98983	3.25695	0.26712	2416	2391	44.48	54.68
Drug Standard	41	3.00922	3.01175	0.00253	3714866	3714841	7,296,469.75	8,969,881.51
Drug Standard	42	3.00332	3.00586	0.00254	3769488	3769463	7,374,606.39	9,065,938.43
Drug Standard	43	3.00648	3.00889	0.00241	3981110	3981085	8,208,758.61	10,091,399.63
							0.201 cpm/fmol	0.164 cpm/fmol

Auto Calculations		Enter Values	
Total uL of Dose Sol =	65.0	Oxytocin Molecular Weight	1,007
Hot mg =	0.0000	Date of Synthesis =	10/03/07
Cold mg =	0.5097	Hot uCi/uL at Synthesis =	**2.300**
Total mg in Dose Sol =	0.5097	Hot uCi/ug at Synthesis =	**1985.72**
Conc Of Dose Sol (ug/uL) =	7.8384	Cold ug/uL =	15.00
Days after Synthesis Delivered =	19	Desired uCi =	45.0
Decay on Day Delivered =	0.8030	Desired mg =	0.400
Total uCi in Dose Sol on Day Delivered =	57.35	Estimated Desired nmol =	397.1
uCi/uL in Dose Sol on Day Delivered =	0.8818	**Date of Delivery =**	**10/22/07**
mg delivered =	0.400	**uL delivered (actual) =**	**51.0**
uCi delivered =	44.97	**Date of Counting =**	**10/22/07**
Estimated nmol delivered =	396.90	**Dose Calculator**	
Days after Synthesis Counted =	19	uL Hot to Deliver =	24.37
Decay on Day Counted =	0.8030	uL Cold to Deliver =	26.66
Specific Activity from Amersham Specs (cpm/fmol) =	0.201	Total uL to Deliver =	51.03
Avg. Volume of Standard (uL)* =	3.0	Hot to Cold Ratio =	0.91
Avg. CPM-bkgd of Standard =	3,821,796	Total uL to Make =	65.03
Specific Activity from Standards: (CPM/fmol) =	0.164	Dose Solution uL Hot =	31.05
Date of Disposal from Decay Storage =	05/25/09	Dose Solution uL Cold =	33.98

Table A11: Intranasal Oxytocin Rat 11 Data

Oxytocin Rat 11	Route	Com-pound	Volume (uL)	uCi	mg	Time (min)	Perfused @ (min)
Amount Delivered:	IN	Oxytocin	51	44.97	0.400	14:00	30:45

Tissue Description	Tube #	Tube (g)	Tissue + Tube (g)	Tissue Weight (g)	CPM	CPM -25 bkgd	nM (Spec. Act.)	nM (Stan.)
Dorsal Dura	1	2.99129	3.00410	0.01281	176	151	58.58	72.01
Ventral Dura	2	3.01307	3.02205	0.00898	194	169	93.52	114.97
Trigeminal Ganglion	3	-	-	-	-	-	-	-
Maxillary Nerve	4	3.00436	3.01528	0.01092	970	945	430.03	528.66
Opthalmic Nerve	5	2.97142	2.97477	0.00335	92	67	99.39	122.18
Mandibular Nerve	6	2.98258	2.98539	0.00281	86	61	107.87	132.61
Upper Cervical Spinal Cord	7	-	-	-	-	-	-	-
Olfactory Bulbs	8	-	-	-	-	-	-	-
Anterior Olfactory Nucleus	9	-	-	-	-	-	-	-
Frontal Cortex	10	-	-	-	-	-	-	-
Caudate/Putamen	11	-	-	-	-	-	-	-
Septal Nucleus	12	-	-	-	-	-	-	-
Parietal Cortex	13	-	-	-	-	-	-	-
Hippocampus	14	-	-	-	-	-	-	-
Thalamus	15	-	-	-	-	-	-	-
Hypothalamus	16	-	-	-	-	-	-	-
Midbrain	17	-	-	-	-	-	-	-
Pons	18	-	-	-	-	-	-	-
Medulla	19	-	-	-	-	-	-	-
Cerebellum	20	-	-	-	-	-	-	-
Superficial Nodes (2)	21	2.97657	3.03353	0.05696	2832	2807	244.89	301.05
Cervical Nodes (2)	22	3.01228	3.02392	0.01164	315	290	123.80	152.20
Thyroid	23	2.99657	3.02019	0.02362	2252	2227	468.53	575.98
Olfactory Epithelium	24	3.07249	3.17166	0.09917	123894	123869	6,206.91	7,630.44
Respiratory Epithelium	25	3.02790	3.06689	0.03899	2641024	2640999	336,595.32	413,791.91
Axillary Nodes (2)	26	2.99948	3.03787	0.03839	128	103	13.33	16.39
Muscle (R, deltoid)	27	3.05953	3.09309	0.03356	826	801	118.61	145.81
Liver (R, superficial lobe)	28	2.97227	3.00417	0.03190	175	150	23.37	28.73
Kidney (L, tip)	29	2.96046	3.00701	0.04655	286	261	27.86	34.25
Lung (R, top lobe)	30	2.99319	3.03294	0.03975	499	474	59.26	72.85
Trachea (near cut-off)	31	2.99721	3.01536	0.01815	158	133	36.41	44.77
Esophagus (near cut-off)	32	2.98504	2.99961	0.01457	92	67	22.85	28.09
Spleen (tip)	33	2.98754	3.02674	0.03920	177	152	19.27	23.69
Heart	34	2.96149	2.99169	0.03020	219	194	31.92	39.24
Urine (fill to line of tube)	35	2.98820	3.27221	0.28401	852	827	14.47	17.79
Spinal Dura	36	2.96613	2.97756	0.01143	130	105	45.65	56.12
Lower Cervical Spinal Cord	37	2.97917	3.13065	0.15148	168	143	4.69	5.77
Thoracic Spinal Cord	38	2.98783	3.07176	0.08393	114	89	5.27	6.48
Lumbar Spinal Cord	39	2.99057	3.13195	0.14138	174	149	5.24	6.44
Blood Sample	40	3.00849	3.26801	0.25952	2986	2961	56.70	69.70
Drug Standard	41	3.00922	3.01175	0.00253	3714866	3714841	7,296,469.75	8,969,881.51
Drug Standard	42	3.00332	3.00586	0.00254	3769488	3769463	7,374,606.39	9,065,938.43
Drug Standard	43	3.00648	3.00889	0.00241	3981110	3981085	8,208,758.61	10,091,399.63
							0.201 cpm/fmol	0.164 cpm/fmol

Auto Calculations		Enter Values	
Total uL of Dose Sol =	65.0	Oxytocin Molecular Weight	1,007
Hot mg =	0.0000	Date of Synthesis =	10/03/07
Cold mg =	0.5097	Hot uCi/uL at Synthesis =	**2.300**
Total mg in Dose Sol =	0.5097	Hot uCi/ug at Synthesis =	**1985.72**
Conc Of Dose Sol (ug/uL) =	7.8384	Cold ug/uL =	15.00
Days after Synthesis Delivered =	19	Desired uCi =	45.0
Decay on Day Delivered =	0.8030	Desired mg =	0.400
Total uCi in Dose Sol on Day Delivered =	57.35	Estimated Desired nmol =	397.1
uCi/uL in Dose Sol on Day Delivered =	0.8818	**Date of Delivery =**	**10/22/07**
mg delivered =	0.400	**uL delivered (actual) =**	**51.0**
uCi delivered =	44.97	**Date of Counting =**	**10/22/07**
Estimated nmol delivered =	396.90	**Dose Calculator**	
Days after Synthesis Counted =	19	uL Hot to Deliver =	24.37
Decay on Day Counted =	0.8030	uL Cold to Deliver =	26.66
Specific Activity from Amersham Specs (cpm/fmol) =	0.201	Total uL to Deliver =	51.03
Avg. Volume of Standard (uL)* =	3.0	Hot to Cold Ratio =	0.91
Avg. CPM-bkgd of Standard =	3,821,796	Total uL to Make =	65.03
Specific Activity from Standards: (CPM/fmol) =	0.164	Dose Solution uL Hot =	31.05
Date of Disposal from Decay Storage =	05/25/09	Dose Solution uL Cold =	33.98

Table A12: Intranasal Oxytocin Rat 12 Data

**Oxytocin Rat 12**	Route	Com-pound	Volume (uL)	uCi	mg	Time (min)	Perfused @ (min)
Amount Delivered:	IN	Oxytocin	52	45.01	0.400	14:09	30:59

Tissue Description	Tube #	Tube (g)	Tissue + Tube (g)	Tissue Weight (g)	CPM	CPM -25 bkgd	nM (Spec. Act.)	nM (Stan.)
Dorsal Dura	1	3.00438	3.01466	0.01028	1803	1778	859.47	1,065.61
Ventral Dura	2	3.02096	3.02943	0.00847	838	813	476.98	591.38
Trigeminal Ganglion	3	-	-	-	-	-	-	-
Maxillary Nerve	4	3.00542	3.01646	0.01104	876	851	383.05	474.92
Opthalmic Nerve	5	2.99631	2.99893	0.00262	315	290	550.03	681.96
Mandibular Nerve	6	3.00839	3.01121	0.00282	1682	1657	2,919.89	3,620.21
Upper Cervical Spinal Cord	7	-	-	-	-	-	-	-
Olfactory Bulbs	8	-	-	-	-	-	-	-
Anterior Olfactory Nucleus	9	-	-	-	-	-	-	-
Frontal Cortex	10	-	-	-	-	-	-	-
Caudate/Putamen	11	-	-	-	-	-	-	-
Septal Nucleus	12	-	-	-	-	-	-	-
Parietal Cortex	13	-	-	-	-	-	-	-
Hippocampus	14	-	-	-	-	-	-	-
Thalamus	15	-	-	-	-	-	-	-
Hypothalamus	16	-	-	-	-	-	-	-
Midbrain	17	-	-	-	-	-	-	-
Pons	18	-	-	-	-	-	-	-
Medulla	19	-	-	-	-	-	-	-
Cerebellum	20	-	-	-	-	-	-	-
Superficial Nodes (2)	21	2.99668	3.05700	0.06032	2727	2702	222.60	275.98
Cervical Nodes (2)	22	2.99192	3.00821	0.01629	1220	1195	364.54	451.97
Thyroid	23	3.07397	3.09194	0.01797	1501	1476	408.16	506.06
Olfactory Epithelium	24	3.08222	3.17885	0.09663	1963983	1963958	100,998.12	125,221.95
Respiratory Epithelium	25	2.98673	3.04655	0.05982	4915079	4915054	408,295.85	506,223.29
Axillary Nodes (2)	26	2.99043	3.03448	0.04405	110	85	9.59	11.89
Muscle (R, deltoid)	27	2.99723	3.03316	0.03593	81	56	7.75	9.60
Liver (R, superficial lobe)	28	2.96681	3.01067	0.04386	107	82	9.29	11.52
Kidney (L, tip)	29	2.98937	3.03171	0.04234	263	238	27.93	34.63
Lung (R, top lobe)	30	3.03066	3.07502	0.04436	106	81	9.07	11.25
Trachea (near cut-off)	31	2.97855	2.99068	0.01213	45	20	8.19	10.16
Esophagus (near cut-off)	32	2.98189	2.99739	0.01550	68	43	13.79	17.09
Spleen (tip)	33	2.98121	3.02594	0.04473	148	123	13.66	16.94
Heart	34	2.96955	3.00770	0.03815	96	71	9.25	11.47
Urine (fill to line of tube)	35	3.00372	3.22396	0.22024	2422	2397	54.08	67.06
Spinal Dura	36	2.95911	2.97247	0.01336	60	35	13.02	16.14
Lower Cervical Spinal Cord	37	2.98551	3.11427	0.12876	102	77	2.97	3.68
Thoracic Spinal Cord	38	2.98687	3.07296	0.08609	80	55	3.17	3.94
Lumbar Spinal Cord	39	3.01100	3.14531	0.13431	74	49	1.81	2.25
Blood Sample	40	2.97819	3.23945	0.26126	1647	1622	30.85	38.25
Drug Standard	41	2.98312	2.98591	0.00279	3674191	3674166	6,544,065.66	8,113,622.71
Drug Standard	42	2.99071	2.99377	0.00306	3897735	3897710	6,329,671.54	7,847,807.38
Drug Standard	43	3.05284	3.05511	0.00227	3608251	3608226	7,898,796.98	9,793,278.67
							0.201 cpm/fmol	0.162 cpm/fmol

**Auto Calculations**		**Enter Values**	
Total uL of Dose Sol =	65.9	Oxytocin Molecular Weight	1,007
Hot mg =	0.0000	Date of Synthesis =	10/03/07
Cold mg =	0.5079	Hot uCi/uL at Synthesis =	**2.300**
Total mg in Dose Sol =	0.5079	Hot uCi/ug at Synthesis =	**1985.72**
Conc Of Dose Sol (ug/uL) =	7.7086	Cold ug/uL =	15.00
Days after Synthesis Delivered =	22	Desired uCi =	45.0
Decay on Day Delivered =	0.7756	Desired mg =	0.400
Total uCi in Dose Sol on Day Delivered =	57.14	Estimated Desired nmol =	397.1
uCi/uL in Dose Sol on Day Delivered =	0.8672	**Date of Delivery =**	**10/25/07**
mg delivered =	0.400	**uL delivered (actual) =**	**51.9**
uCi delivered =	45.01	**Date of Counting =**	**10/25/07**
Estimated nmol delivered =	397.22	**Dose Calculator**	
Days after Synthesis Counted =	22	uL Hot to Deliver =	25.23
Decay on Day Counted =	0.7756	uL Cold to Deliver =	26.66
Specific Activity from Amersham Specs (cpm/fmol) =	0.201	Total uL to Deliver =	51.89
Avg. Volume of Standard (uL)* =	3.0	Hot to Cold Ratio =	0.95
Avg. CPM-bkgd of Standard =	3,726,701	Total uL to Make =	65.89
Specific Activity from Standards: (CPM/fmol) =	0.162	Dose Solution uL Hot =	32.03
Date of Disposal from Decay Storage =	05/25/09	Dose Solution uL Cold =	33.86

Table A13: Intranasal Oxytocin Rat 13 Data

Oxytocin Rat 13	Route	Com-pound	Volume (uL)	uCi	mg	Time (min)	Perfused @ (min)
Amount Delivered:	IN	Oxytocin	52	45.01	0.400	14:02	31:04

Tissue Description	Tube #	Tube (g)	Tissue + Tube (g)	Tissue Weight (g)	CPM	CPM -25 bkgd	nM (Spec. Act.)	nM (Stan.)
Dorsal Dura	1	3.02928	3.04847	0.01919	616	591	153.04	189.75
Ventral Dura	2	2.98636	2.99250	0.00614	518	493	399.00	494.70
Trigeminal Ganglion	3	-	-	-	-	-	-	-
Maxillary Nerve	4	3.00240	3.01411	0.01171	1096	1071	454.49	563.50
Opthalmic Nerve	5	3.01410	3.01809	0.00399	223	198	246.60	305.74
Mandibular Nerve	6	2.97053	2.97391	0.00338	291	266	391.07	484.87
Upper Cervical Spinal Cord	7	-	-	-	-	-	-	-
Olfactory Bulbs	8	-	-	-	-	-	-	-
Anterior Olfactory Nucleus	9	-	-	-	-	-	-	-
Frontal Cortex	10	-	-	-	-	-	-	-
Caudate/Putamen	11	-	-	-	-	-	-	-
Septal Nucleus	12	-	-	-	-	-	-	-
Parietal Cortex	13	-	-	-	-	-	-	-
Hippocampus	14	-	-	-	-	-	-	-
Thalamus	15	-	-	-	-	-	-	-
Hypothalamus	16	-	-	-	-	-	-	-
Midbrain	17	-	-	-	-	-	-	-
Pons	18	-	-	-	-	-	-	-
Medulla	19	-	-	-	-	-	-	-
Cerebellum	20	-	-	-	-	-	-	-
Superficial Nodes (2)	21	2.99348	3.05048	0.05700	3739	3714	323.79	401.45
Cervical Nodes (2)	22	2.99224	3.00365	0.01141	591	566	246.50	305.63
Thyroid	23	2.97242	2.98801	0.01559	1415	1390	443.06	549.32
Olfactory Epithelium	24	2.98674	3.10075	0.11401	1791923	1791898	78,102.23	96,834.60
Respiratory Epithelium	25	2.99999	3.09003	0.09004	7842143	7842118	432,803.70	536,609.21
Axillary Nodes (2)	26	2.99767	3.03194	0.03427	144	119	17.26	21.39
Muscle (R, deltoid)	27	2.97402	3.00155	0.02753	68	43	7.76	9.62
Liver (R, superficial lobe)	28	2.97886	3.02031	0.04145	84	59	7.07	8.77
Kidney (L, tip)	29	2.97189	3.00957	0.03768	345	320	42.20	52.32
Lung (R, top lobe)	30	2.98475	3.01041	0.02566	84	59	11.43	14.17
Trachea (near cut-off)	31	2.97853	2.99176	0.01323	52	27	10.14	12.57
Esophagus (near cut-off)	32	2.96806	2.97743	0.00937	77	52	27.58	34.19
Spleen (tip)	33	2.96927	3.01640	0.04713	139	114	12.02	14.90
Heart	34	2.99834	3.02743	0.02909	83	58	9.91	12.28
Urine (fill to line of tube)	35	2.98575	3.19964	0.21389	1544	1519	35.29	43.75
Spinal Dura	36	2.97052	2.98297	0.01245	59	34	13.57	16.83
Lower Cervical Spinal Cord	37	2.98991	3.12224	0.13233	87	62	2.33	2.89
Thoracic Spinal Cord	38	2.96825	3.07240	0.10415	79	54	2.58	3.19
Lumbar Spinal Cord	39	2.98239	3.12546	0.14307	77	52	1.81	2.24
Blood Sample	40	3.00366	3.27272	0.26906	1836	1811	33.45	41.47
Drug Standard	41	2.98312	2.98591	0.00279	3674191	3674166	6,544,065.66	8,113,622.71
Drug Standard	42	2.99071	2.99377	0.00306	3897735	3897710	6,329,671.54	7,847,807.38
Drug Standard	43	3.05284	3.05511	0.00227	3608251	3608226	7,898,796.98	9,793,278.67
							0.201 cpm/fmol	0.162 cpm/fmol

Auto Calculations		Enter Values	
Total uL of Dose Sol =	65.9	Oxytocin Molecular Weight	1,007
Hot mg =	0.0000	Date of Synthesis =	10/03/07
Cold mg =	0.5079	Hot uCi/uL at Synthesis =	**2.300**
Total mg in Dose Sol =	0.5079	Hot uCi/ug at Synthesis =	**1985.72**
Conc Of Dose Sol (ug/uL) =	7.7086	Cold ug/uL =	15.00
Days after Synthesis Delivered =	22	Desired uCi =	45.0
Decay on Day Delivered =	0.7756	Desired mg =	0.400
Total uCi in Dose Sol on Day Delivered =	57.14	Estimated Desired nmol =	397.1
uCi/uL in Dose Sol on Day Delivered =	0.8672	**Date of Delivery =**	**10/25/07**
mg delivered =	0.400	**uL delivered (actual) =**	**51.9**
uCi delivered =	45.01	**Date of Counting =**	**10/25/07**
Estimated nmol delivered =	397.22	**Dose Calculator**	
Days after Synthesis Counted =	22	uL Hot to Deliver =	25.23
Decay on Day Counted =	0.7756	uL Cold to Deliver =	26.66
Specific Activity from Amersham Specs (cpm/fmol) =	0.201	Total uL to Deliver =	51.89
Avg. Volume of Standard (uL)* =	3.0	Hot to Cold Ratio =	0.95
Avg. CPM-bkgd of Standard =	3,726,701	Total uL to Make =	65.89
Specific Activity from Standards: (CPM/fmol) =	0.162	Dose Solution uL Hot =	32.03
Date of Disposal from Decay Storage =	05/25/09	Dose Solution uL Cold =	33.86

Table A14: Intranasal Oxytocin Rat 14 Data

Oxytocin Rat 14	Route	Com-pound	Volume (uL)	uCi	mg	Time (min)	Perfused @ (min)
Amount Delivered:	IN	Oxytocin	53	45.01	0.400	14:00	30:50

Tissue Description	Tube #	Tube (g)	Tissue + Tube (g)	Tissue Weight (g)	CPM	CPM -25 bkgd	nM (Spec. Act.)	nM (Stan.)
Dorsal Dura	1	2.95456	2.96428	0.00972	223	198	101.23	127.57
Ventral Dura	2	2.97210	2.98115	0.00905	774	749	411.27	518.29
Trigeminal Ganglion	3	-	-	-	-	-	-	-
Maxillary Nerve	4	2.96692	2.98381	0.01689	643	618	181.82	229.14
Opthalmic Nerve	5	2.98275	2.98613	0.00338	67	42	61.75	77.82
Mandibular Nerve	6	2.98574	2.98990	0.00416	139	114	136.18	171.61
Upper Cervical Spinal Cord	7	-	-	-	-	-	-	-
Olfactory Bulbs	8	-	-	-	-	-	-	-
Anterior Olfactory Nucleus	9	-	-	-	-	-	-	-
Frontal Cortex	10	-	-	-	-	-	-	-
Caudate/Putamen	11	-	-	-	-	-	-	-
Septal Nucleus	12	-	-	-	-	-	-	-
Parietal Cortex	13	-	-	-	-	-	-	-
Hippocampus	14	-	-	-	-	-	-	-
Thalamus	15	-	-	-	-	-	-	-
Hypothalamus	16	-	-	-	-	-	-	-
Midbrain	17	-	-	-	-	-	-	-
Pons	18	-	-	-	-	-	-	-
Medulla	19	-	-	-	-	-	-	-
Cerebellum	20	-	-	-	-	-	-	-
Superficial Nodes (2)	21	2.97355	3.03348	0.05993	3228	3203	265.59	334.70
Cervical Nodes (2)	22	2.97067	2.98421	0.01354	624	599	219.84	277.04
Thyroid	23	2.95828	2.97610	0.01782	1334	1309	365.03	460.01
Olfactory Epithelium	24	2.97073	3.06035	0.08962	99274	99249	5,503.19	6,935.19
Respiratory Epithelium	25	2.98318	3.02616	0.04298	8257396	8257371	954,703.39	1,203,130.30
Axillary Nodes (2)	26	3.02979	3.07869	0.04890	251	226	22.97	28.94
Muscle (R, deltoid)	27	3.00764	3.03589	0.02825	86	61	10.73	13.52
Liver (R, superficial lobe)	28	3.00043	3.05477	0.05434	124	99	9.05	11.41
Kidney (L, tip)	29	3.04644	3.08580	0.03936	535	510	64.39	81.14
Lung (R, top lobe)	30	3.05026	3.08541	0.03515	94	69	9.75	12.29
Trachea (near cut-off)	31	2.98657	3.00148	0.01491	105	80	26.66	33.60
Esophagus (near cut-off)	32	3.01023	3.02247	0.01224	93	68	27.61	34.79
Spleen (tip)	33	2.98652	3.02728	0.04076	212	187	22.80	28.73
Heart	34	2.99828	3.03437	0.03609	133	108	14.87	18.74
Urine (fill to line of tube)	35	3.00854	3.34471	0.33617	5352	5327	78.74	99.23
Spinal Dura	36	2.98561	2.99978	0.01417	123	98	34.37	43.31
Lower Cervical Spinal Cord	37	2.98221	3.11310	0.13089	108	83	3.15	3.97
Thoracic Spinal Cord	38	3.01037	3.08746	0.07709	85	60	3.87	4.87
Lumbar Spinal Cord	39	2.97499	3.13390	0.15891	131	106	3.31	4.18
Blood Sample	40	2.98868	3.25761	0.26893	2937	2912	53.81	67.81
Drug Standard	41	2.99211	2.99489	0.00278	3534344	3534319	6,317,627.66	7,961,560.99
Drug Standard	42	2.98650	2.98904	0.00254	3516161	3516136	6,878,995.50	8,669,004.44
Drug Standard	43	2.97921	2.98207	0.00286	3701829	3701804	6,431,917.49	8,105,590.60
							0.201 cpm/fmol	0.160 cpm/fmol

Auto Calculations		Enter Values	
Total uL of Dose Sol =	67.1	Oxytocin Molecular Weight	1,007
Hot mg =	0.0000	Date of Synthesis =	10/03/07
Cold mg =	0.5055	Hot uCi/uL at Synthesis =	**2.300**
Total mg in Dose Sol =	0.5055	Hot uCi/ug at Synthesis =	**1985.72**
Conc Of Dose Sol (ug/uL) =	7.5354	Cold ug/uL =	15.00
Days after Synthesis Delivered =	26	Desired uCi =	45.0
Decay on Day Delivered =	0.7406	Desired mg =	0.400
Total uCi in Dose Sol on Day Delivered =	56.87	Estimated Desired nmol =	397.1
uCi/uL in Dose Sol on Day Delivered =	0.8477	**Date of Delivery =**	**10/29/07**
mg delivered =	0.400	**uL delivered (actual) =**	**53.1**
uCi delivered =	45.01	**Date of Counting =**	**10/29/07**
Estimated nmol delivered =	397.27	**Dose Calculator**	
Days after Synthesis Counted =	26	uL Hot to Deliver =	26.42
Decay on Day Counted =	0.7406	uL Cold to Deliver =	26.66
Specific Activity from Amersham Specs (cpm/fmol) =	0.201	Total uL to Deliver =	53.08
Avg. Volume of Standard (uL)* =	3.0	Hot to Cold Ratio =	0.99
Avg. CPM-bkgd of Standard =	3,584,086	Total uL to Make =	67.08
Specific Activity from Standards: (CPM/fmol) =	0.160	Dose Solution uL Hot =	33.39
Date of Disposal from Decay Storage =	05/25/09	Dose Solution uL Cold =	33.70

Table A15: Intranasal Oxytocin Rat 15 Data

Oxytocin Rat 15	Route	Com-pound	Volume (uL)	uCi	mg	Time (min)	Perfused @ (min)
Amount Delivered:	IN	Oxytocin	53	45.01	0.400	14:00	31:19

Tissue Description	Tube #	Tube (g)	Tissue + Tube (g)	Tissue Weight (g)	CPM	CPM -25 bkgd	nM (Spec. Act.)	nM (Stan.)
Dorsal Dura	1	2.98611	2.99863	0.01252	334	309	122.64	154.56
Ventral Dura	2	2.97834	2.98686	0.00852	308	283	165.06	208.01
Trigeminal Ganglion	3	-	-	-	-	-	-	-
Maxillary Nerve	4	2.98734	2.99985	0.01251	181	156	61.97	78.09
Opthalmic Nerve	5	2.99807	3.00009	0.00202	72	47	115.62	145.71
Mandibular Nerve	6	2.97417	2.97685	0.00268	105	80	148.34	186.94
Upper Cervical Spinal Cord	7	-	-	-	-	-	-	-
Olfactory Bulbs	8	-	-	-	-	-	-	-
Anterior Olfactory Nucleus	9	-	-	-	-	-	-	-
Frontal Cortex	10	-	-	-	-	-	-	-
Caudate/Putamen	11	-	-	-	-	-	-	-
Septal Nucleus	12	-	-	-	-	-	-	-
Parietal Cortex	13	-	-	-	-	-	-	-
Hippocampus	14	-	-	-	-	-	-	-
Thalamus	15	-	-	-	-	-	-	-
Hypothalamus	16	-	-	-	-	-	-	-
Midbrain	17	-	-	-	-	-	-	-
Pons	18	-	-	-	-	-	-	-
Medulla	19	-	-	-	-	-	-	-
Cerebellum	20	-	-	-	-	-	-	-
Superficial Nodes (2)	21	2.96129	3.02535	0.06406	2479	2454	190.36	239.90
Cervical Nodes (2)	22	2.99088	3.00370	0.01282	276	251	97.29	122.61
Thyroid	23	3.00123	3.01840	0.01717	1336	1311	379.42	478.16
Olfactory Epithelium	24	2.97353	3.06173	0.08820	95592	95567	5,384.34	6,785.42
Respiratory Epithelium	25	2.99725	3.04879	0.05154	3298267	3298242	318,002.97	400,751.71
Axillary Nodes (2)	26	2.98928	3.03053	0.04125	160	135	16.26	20.49
Muscle (R, deltoid)	27	3.01352	3.05206	0.03854	1656	1631	210.30	265.02
Liver (R, superficial lobe)	28	3.00248	3.03729	0.03481	728	703	100.36	126.47
Kidney (L, tip)	29	2.99237	3.03659	0.04422	732	707	79.45	100.12
Lung (R, top lobe)	30	3.00334	3.04413	0.04079	291	266	32.41	40.84
Trachea (near cut-off)	31	3.01701	3.03208	0.01507	121	96	31.66	39.89
Esophagus (near cut-off)	32	2.98629	2.99911	0.01282	64	39	15.12	19.05
Spleen (tip)	33	2.98300	3.03323	0.05023	265	240	23.74	29.92
Heart	34	3.00444	3.03380	0.02936	397	372	62.96	79.35
Urine (fill to line of tube)	35	3.02017	3.29361	0.27344	6406	6381	115.96	146.14
Spinal Dura	36	3.00088	3.02491	0.02403	157	132	27.30	34.40
Lower Cervical Spinal Cord	37	2.99504	3.07816	0.08312	159	134	8.01	10.10
Thoracic Spinal Cord	38	3.01772	3.09746	0.07974	261	236	14.71	18.53
Lumbar Spinal Cord	39	2.99791	3.12184	0.12393	156	131	5.25	6.62
Blood Sample	40	3.00207	3.29856	0.29649	3547	3522	59.03	74.39
Drug Standard	41	2.99211	2.99489	0.00278	3534344	3534319	6,317,627.66	7,961,560.99
Drug Standard	42	2.98650	2.98904	0.00254	3516161	3516136	6,878,995.50	8,669,004.44
Drug Standard	43	2.97921	2.98207	0.00286	3701829	3701804	6,431,917.49	8,105,590.60
							0.201 cpm/fmol	0.160 cpm/fmol

Auto Calculations		Enter Values	
Total uL of Dose Sol =	67.1	Oxytocin Molecular Weight	1,007
Hot mg =	0.0000	Date of Synthesis =	10/03/07
Cold mg =	0.5055	Hot uCi/uL at Synthesis =	**2.300**
Total mg in Dose Sol =	0.5055	Hot uCi/ug at Synthesis =	**1985.72**
Conc Of Dose Sol (ug/uL) =	7.5354	Cold ug/uL =	15.00
Days after Synthesis Delivered =	26	Desired uCi =	45.0
Decay on Day Delivered =	0.7406	Desired mg =	0.400
Total uCi in Dose Sol on Day Delivered =	56.87	Estimated Desired nmol =	397.1
uCi/uL in Dose Sol on Day Delivered =	0.8477	**Date of Delivery =**	**10/29/07**
mg delivered =	0.400	**uL delivered (actual) =**	**53.1**
uCi delivered =	45.01	**Date of Counting =**	**10/29/07**
Estimated nmol delivered =	397.27	**Dose Calculator**	
Days after Synthesis Counted =	26	uL Hot to Deliver =	26.42
Decay on Day Counted =	0.7406	uL Cold to Deliver =	26.66
Specific Activity from Amersham Specs (cpm/fmol) =	0.201	Total uL to Deliver =	53.08
Avg. Volume of Standard (uL)* =	3.0	Hot to Cold Ratio =	0.99
Avg. CPM-bkgd of Standard =	3,584,086	Total uL to Make =	67.08
Specific Activity from Standards: (CPM/fmol) =	0.160	Dose Solution uL Hot =	33.39
Date of Disposal from Decay Storage =	05/25/09	Dose Solution uL Cold =	33.70

Table A16: Intranasal Oxytocin Rat 16 Data

Oxytocin Rat 16	Route	Com-pound	Volume (uL)	uCi	mg	Time (min)	Perfused @ (min)
Amount Delivered:	IN	Oxytocin	48	45.00	0.400	14:00	32:43

Tissue Description	Tube #	Tube (g)	Tissue + Tube (g)	Tissue Weight (g)	CPM	CPM -25 bkgd	nM (Spec. Act.)	nM (Stan.)
Dorsal Dura	1	2.99507	3.00814	0.01307	479	454	172.61	200.81
Ventral Dura	2	3.00441	3.03776	0.03335	2042	2017	300.54	349.63
Trigeminal Ganglion	3	-	-	-	-	-	-	-
Maxillary Nerve	4	2.99888	3.00882	0.00994	171	146	72.99	84.91
Opthalmic Nerve	5	3.00301	3.00532	0.00231	227	202	434.54	505.52
Mandibular Nerve	6	2.98387	2.98706	0.00319	123	98	152.66	177.60
Upper Cervical Spinal Cord	7	-	-	-	-	-	-	-
Olfactory Bulbs	8	-	-	-	-	-	-	-
Anterior Olfactory Nucleus	9	-	-	-	-	-	-	-
Frontal Cortex	10	-	-	-	-	-	-	-
Caudate/Putamen	11	-	-	-	-	-	-	-
Septal Nucleus	12	-	-	-	-	-	-	-
Parietal Cortex	13	-	-	-	-	-	-	-
Hippocampus	14	-	-	-	-	-	-	-
Thalamus	15	-	-	-	-	-	-	-
Hypothalamus	16	-	-	-	-	-	-	-
Midbrain	17	-	-	-	-	-	-	-
Pons	18	-	-	-	-	-	-	-
Medulla	19	-	-	-	-	-	-	-
Cerebellum	20	-	-	-	-	-	-	-
Superficial Nodes (2)	21	3.00187	3.07912	0.07725	3183	3158	203.15	236.33
Cervical Nodes (2)	22	3.01372	3.02020	0.00648	132	107	82.05	95.46
Thyroid	23	2.97467	2.99674	0.02207	2766	2741	617.16	717.97
Olfactory Epithelium	24	2.99842	3.07036	0.07194	20277	20252	1,398.91	1,627.41
Respiratory Epithelium	25	3.00536	3.06338	0.05802	8520706	8520681	729,776.14	848,976.47
Axillary Nodes (2)	26	2.97058	3.01387	0.04329	3274	3249	372.95	433.87
Muscle (R, deltoid)	27	2.98451	3.03852	0.05401	537	512	47.11	54.80
Liver (R, superficial lobe)	28	2.97389	3.01398	0.04009	125	100	12.40	14.42
Kidney (L, tip)	29	2.99197	3.02899	0.03702	320	295	39.60	46.07
Lung (R, top lobe)	30	2.97576	3.00658	0.03082	145	120	19.35	22.51
Trachea (near cut-off)	31	2.97354	2.98879	0.01525	82	57	18.57	21.61
Esophagus (near cut-off)	32	2.99947	3.01608	0.01661	95	70	20.94	24.36
Spleen (tip)	33	3.00858	3.05307	0.04449	181	156	17.42	20.27
Heart	34	2.95714	2.98993	0.03279	114	89	13.49	15.69
Urine (fill to line of tube)	35	2.95944	3.26274	0.30330	10900	10875	178.18	207.28
Spinal Dura	36	2.95813	2.98058	0.02245	139	114	25.23	29.36
Lower Cervical Spinal Cord	37	3.02500	3.17297	0.14797	390	365	12.26	14.26
Thoracic Spinal Cord	38	2.98137	3.06330	0.08193	81	56	3.40	3.95
Lumbar Spinal Cord	39	2.97909	3.12494	0.14585	151	126	4.29	4.99
Blood Sample	40	2.97981	3.26040	0.28059	3531	3506	62.09	72.23
Drug Standard	41	2.99813	3.00077	0.00264	4594777	4594752	8,648,707.83	10,061,372.30
Drug Standard	42	3.06522	3.06738	0.00216	3732919	3732894	8,587,860.55	9,990,586.35
Drug Standard	43	2.98506	2.98782	0.00276	4635805	4635780	8,346,546.41	9,709,856.38
							0.201 cpm/fmol	0.173 cpm/fmol

Auto Calculations		Enter Values	
Total uL of Dose Sol =	61.7	Oxytocin Molecular Weight	1,007
Hot mg =	0.0000	Date of Synthesis =	10/23/07
Cold mg =	0.5174	Hot uCi/uL at Synthesis =	**2.320**
Total mg in Dose Sol =	0.5174	Hot uCi/ug at Synthesis =	**1985.72**
Conc Of Dose Sol (ug/uL) =	8.3866	Cold ug/uL =	15.00
Days after Synthesis Delivered =	7	Desired uCi =	45.0
Decay on Day Delivered =	0.9223	Desired mg =	0.400
Total uCi in Dose Sol on Day Delivered =	58.21	Estimated Desired nmol =	397.1
uCi/uL in Dose Sol on Day Delivered =	0.9435	**Date of Delivery =**	**10/30/07**
mg delivered =	0.400	**uL delivered (actual) =**	**47.7**
uCi delivered =	45.00	**Date of Counting =**	**10/30/07**
Estimated nmol delivered =	397.10	**Dose Calculator**	
Days after Synthesis Counted =	7	uL Hot to Deliver =	21.03
Decay on Day Counted =	0.9223	uL Cold to Deliver =	26.67
Specific Activity from Amersham Specs (cpm/fmol) =	0.201	Total uL to Deliver =	47.69
Avg. Volume of Standard (uL)* =	3.0	Hot to Cold Ratio =	0.79
Avg. CPM-bkgd of Standard =	4,321,142	Total uL to Make =	61.69
Specific Activity from Standards: (CPM/fmol) =	0.173	Dose Solution uL Hot =	27.20
Date of Disposal from Decay Storage =	06/14/09	Dose Solution uL Cold =	34.49

Table A17: Intranasal Oxytocin Rat 17 Data

Oxytocin Rat 17	Route	Com-pound	Volume (uL)	uCi	mg	Time (min)	Perfused @ (min)
Amount Delivered:	IN	Oxytocin	48	45.00	0.400	14:05	31:14

Tissue Description	Tube #	Tube (g)	Tissue + Tube (g)	Tissue Weight (g)	CPM	CPM -25 bkgd	nM (Spec. Act.)	nM (Stan.)
Dorsal Dura	1	2.97876	2.98855	0.00979	265	240	121.82	141.72
Ventral Dura	2	3.04210	3.04886	0.00676	245	220	161.72	188.14
Trigeminal Ganglion	3	-	-	-	-	-	-	-
Maxillary Nerve	4	2.99183	3.00212	0.01029	589	564	272.37	316.86
Opthalmic Nerve	5	3.05208	3.05455	0.00247	111	86	173.02	201.28
Mandibular Nerve	6	2.98876	2.99123	0.00247	174	149	299.77	348.73
Upper Cervical Spinal Cord	7	-	-	-	-	-	-	-
Olfactory Bulbs	8	-	-	-	-	-	-	-
Anterior Olfactory Nucleus	9	-	-	-	-	-	-	-
Frontal Cortex	10	-	-	-	-	-	-	-
Caudate/Putamen	11	-	-	-	-	-	-	-
Septal Nucleus	12	-	-	-	-	-	-	-
Parietal Cortex	13	-	-	-	-	-	-	-
Hippocampus	14	-	-	-	-	-	-	-
Thalamus	15	-	-	-	-	-	-	-
Hypothalamus	16	-	-	-	-	-	-	-
Midbrain	17	-	-	-	-	-	-	-
Pons	18	-	-	-	-	-	-	-
Medulla	19	-	-	-	-	-	-	-
Cerebellum	20	-	-	-	-	-	-	-
Superficial Nodes (2)	21	2.98719	3.05684	0.06965	1348	1323	94.39	109.81
Cervical Nodes (2)	22	2.99393	3.00489	0.01096	961	936	424.38	493.70
Thyroid	23	2.98787	3.01230	0.02443	1962	1937	394.00	458.36
Olfactory Epithelium	24	3.00087	3.10957	0.10870	69242	69217	3,164.29	3,681.14
Respiratory Epithelium	25	2.99850	3.04087	0.04237	7164045	7164020	840,216.88	977,456.40
Axillary Nodes (2)	26	2.95543	2.99138	0.03595	149	124	17.14	19.94
Muscle (R, deltoid)	27	2.99774	3.03445	0.03671	99	74	10.02	11.65
Liver (R, superficial lobe)	28	2.97897	3.02017	0.04120	93	68	8.20	9.54
Kidney (L, tip)	29	2.96839	3.01352	0.04513	320	295	32.48	37.79
Lung (R, top lobe)	30	3.02547	3.06470	0.03923	82	57	7.22	8.40
Trachea (near cut-off)	31	3.02943	3.05200	0.02257	81	56	12.33	14.34
Esophagus (near cut-off)	32	2.99041	3.00414	0.01373	67	42	15.20	17.68
Spleen (tip)	33	2.99787	3.04901	0.05114	121	96	9.33	10.85
Heart	34	2.97998	3.02009	0.04011	181	156	19.33	22.48
Urine (fill to line of tube)	35	2.97299	3.33491	0.36192	3131	3106	42.65	49.61
Spinal Dura	36	3.00385	3.02154	0.01769	82	57	16.01	18.63
Lower Cervical Spinal Cord	37	2.99317	3.12905	0.13588	106	81	2.96	3.45
Thoracic Spinal Cord	38	2.96548	3.05292	0.08744	70	45	2.56	2.98
Lumbar Spinal Cord	39	2.96517	3.11356	0.14839	1153	1128	37.77	43.94
Blood Sample	40	3.01792	3.22225	0.20433	2215	2190	53.26	61.96
Drug Standard	41	2.99813	3.00077	0.00264	4594777	4594752	8,648,707.83	10,061,372.30
Drug Standard	42	3.06522	3.06738	0.00216	3732919	3732894	8,587,860.55	9,990,586.35
Drug Standard	43	2.98506	2.98782	0.00276	4635805	4635780	8,346,546.41	9,709,856.38
							0.201 cpm/fmol	0.173 cpm/fmol

Auto Calculations		Enter Values	
Total uL of Dose Sol =	61.7	Oxytocin Molecular Weight	1,007
Hot mg =	0.0000	Date of Synthesis =	10/23/07
Cold mg =	0.5174	Hot uCi/uL at Synthesis =	**2.320**
Total mg in Dose Sol =	0.5174	Hot uCi/ug at Synthesis =	**1985.72**
Conc Of Dose Sol (ug/uL) =	8.3866	Cold ug/uL =	15.00
Days after Synthesis Delivered =	7	Desired uCi =	45.0
Decay on Day Delivered =	0.9223	Desired mg =	0.400
Total uCi in Dose Sol on Day Delivered =	58.21	Estimated Desired nmol =	397.1
uCi/uL in Dose Sol on Day Delivered =	0.9435	**Date of Delivery =**	**10/30/07**
mg delivered =	0.400	**uL delivered (actual) =**	**47.7**
uCi delivered =	45.00	**Date of Counting =**	**10/30/07**
Estimated nmol delivered =	397.19	**Dose Calculator**	
Days after Synthesis Counted =	7	uL Hot to Deliver =	21.03
Decay on Day Counted =	0.9223	uL Cold to Deliver =	26.67
Specific Activity from Amersham Specs (cpm/fmol) =	0.201	Total uL to Deliver =	47.69
Avg. Volume of Standard (uL)* =	3.0	Hot to Cold Ratio =	0.79
Avg. CPM-bkgd of Standard =	4,321,142	Total uL to Make =	61.69
Specific Activity from Standards: (CPM/fmol) =	0.173	Dose Solution uL Hot =	27.20
Date of Disposal from Decay Storage =	06/14/09	Dose Solution uL Cold =	34.49

Table A18: Intranasal Oxytocin Rat 18 Data

Oxytocin Rat 18	Route	Com-pound	Volume (uL)	uCi	mg	Time (min)	Perfused @ (min)
Amount Delivered:	IN	Oxytocin	48	45.00	0.400	14:00	31:25

Tissue Description	Tube #	Tube (g)	Tissue + Tube (g)	Tissue Weight (g)	CPM	CPM -25 bkgd	nM (Spec. Act.)	nM (Stan.)
Dorsal Dura	1	2.99980	3.01309	0.01329	439	414	154.80	176.51
Ventral Dura	2	2.99775	3.00405	0.00630	140	115	90.71	103.43
Trigeminal Ganglion	3	2.96762	2.99835	0.03073	349	324	52.39	59.74
Maxillary Nerve	4	2.98018	2.99048	0.01030	243	218	105.17	119.93
Opthalmic Nerve	5	2.97212	2.97545	0.00333	110	85	126.84	144.64
Mandibular Nerve	6	2.98863	2.99168	0.00305	165	140	228.10	260.10
Upper Cervical Spinal Cord	7	2.98482	3.03964	0.05482	129	104	9.43	10.75
Olfactory Bulbs	8	3.07646	3.16515	0.08869	334	309	17.31	19.74
Anterior Olfactory Nucleus	9	2.97896	3.02255	0.04359	86	61	6.95	7.93
Frontal Cortex	10	2.98105	3.01403	0.03298	121	96	14.46	16.49
Caudate/Putamen	11	2.97835	2.98967	0.01132	45	20	8.78	10.01
Septal Nucleus	12	2.99692	3.00959	0.01267	46	21	8.24	9.39
Parietal Cortex	13	2.99975	3.03391	0.03416	86	61	8.87	10.12
Hippocampus	14	3.01623	3.05827	0.04204	72	47	5.56	6.33
Thalamus	15	2.99915	3.07795	0.07880	99	74	4.67	5.32
Hypothalamus	16	3.07121	3.09728	0.02607	63	38	7.24	8.26
Midbrain	17	2.99421	3.10618	0.11197	149	124	5.50	6.28
Pons	18	3.00996	3.07623	0.06627	100	75	5.62	6.41
Medulla	19	2.97997	3.07386	0.09389	134	109	5.77	6.58
Cerebellum	20	3.00134	3.25988	0.25854	248	223	4.29	4.89
Superficial Nodes (2)	21	3.01231	3.06706	0.05475	2075	2050	186.06	212.17
Cervical Nodes (2)	22	2.99163	3.00298	0.01135	293	268	117.34	133.80
Thyroid	23	2.99353	3.01369	0.02016	1676	1651	406.96	464.05
Olfactory Epithelium	24	2.97788	3.07688	0.09900	43968	43943	2,205.71	2,515.13
Respiratory Epithelium	25	3.00157	3.05946	0.05789	9325395	9325370	800,489.43	912,784.62
Axillary Nodes (2)	26	2.98852	3.02046	0.03194	102	77	11.98	13.66
Muscle (R, deltoid)	27	2.99654	3.05106	0.05452	121	96	8.75	9.98
Liver (R, superficial lobe)	28	2.99119	3.02283	0.03164	100	75	11.78	13.43
Kidney (L, tip)	29	3.03154	3.07394	0.04240	284	259	30.35	34.61
Lung (R, top lobe)	30	2.95729	2.98817	0.03088	129	104	16.74	19.08
Trachea (near cut-off)	31	2.97605	2.99156	0.01551	123	98	31.40	35.80
Esophagus (near cut-off)	32	3.00235	3.01762	0.01527	111	86	27.99	31.91
Spleen (tip)	33	2.97355	3.01624	0.04269	209	184	21.42	24.42
Heart	34	2.96965	3.03421	0.06456	178	153	11.78	13.43
Urine (fill to line of tube)	35	2.96195	3.14306	0.18111	3985	3960	108.65	123.90
Spinal Dura	36	2.97878	2.99926	0.02048	61	36	8.74	9.96
Lower Cervical Spinal Cord	37	2.96198	3.09963	0.13765	142	117	4.22	4.82
Thoracic Spinal Cord	38	2.97727	3.05259	0.07532	77	52	3.43	3.91
Lumbar Spinal Cord	39	2.95405	3.06849	0.11444	93	68	2.95	3.37
Blood Sample	40	2.96856	3.24460	0.27604	3575	3550	63.91	72.87
Drug Standard	41	2.97326	2.97623	0.00297	4436891	4436866	7,423,572.26	8,464,974.49
Drug Standard	42	2.98708	2.98967	0.00259	4471207	4471182	8,578,585.05	9,782,016.13
Drug Standard	43	3.00737	3.01003	0.00266	4182632	4182607	7,813,731.80	8,909,866.85
							0.201 cpm/fmol	0.176 cpm/fmol

Auto Calculations		Enter Values	
Total uL of Dose Sol =	62.2	Oxytocin Molecular Weight	1,007
Hot mg =	0.0000	Date of Synthesis =	10/23/07
Cold mg =	0.5162	Hot uCi/uL at Synthesis =	**2.320**
Total mg in Dose Sol =	0.5162	Hot uCi/ug at Synthesis =	**1985.72**
Conc Of Dose Sol (ug/uL) =	8.3011	Cold ug/uL =	15.00
Days after Synthesis Delivered =	9	Desired uCi =	45.0
Decay on Day Delivered =	0.9013	Desired mg =	0.400
Total uCi in Dose Sol on Day Delivered =	58.07	Estimated Desired nmol =	397.1
uCi/uL in Dose Sol on Day Delivered =	0.9339	**Date of Delivery =**	**11/01/07**
mg delivered =	0.400	**uL delivered (actual) =**	**48.2**
uCi delivered =	45.00	**Date of Counting =**	**11/01/07**
Estimated nmol delivered =	397.17	**Dose Calculator**	
Days after Synthesis Counted =	9	uL Hot to Deliver =	21.52
Decay on Day Counted =	0.9013	uL Cold to Deliver =	26.66
Specific Activity from Amersham Specs (cpm/fmol) =	0.201	Total uL to Deliver =	48.19
Avg. Volume of Standard (uL)* =	3.0	Hot to Cold Ratio =	0.81
Avg. CPM-bkgd of Standard =	4,363,552	Total uL to Make =	62.19
Specific Activity from Standards: (CPM/fmol) =	0.176	Dose Solution uL Hot =	27.77
Date of Disposal from Decay Storage =	06/14/09	Dose Solution uL Cold =	34.41

Table A19: Intranasal Oxytocin Rat 19 Data

Oxytocin Rat 19	Route	Com-pound	Volume (uL)	uCi	mg	Time (min)	Perfused @ (min)
Amount Delivered:	IN	Oxytocin	48	45.00	0.400	14:07	30:50

Tissue Description	Tube #	Tube (g)	Tissue + Tube (g)	Tissue Weight (g)	CPM	CPM -25 bkgd	nM (Spec. Act.)	nM (Stan.)
Dorsal Dura	1	2.99361	3.01062	0.01701	540	515	150.45	172.03
Ventral Dura	2	2.99865	3.00646	0.00781	113	88	55.99	64.02
Trigeminal Ganglion	3	2.98639	3.01947	0.03308	2174	2149	322.82	369.12
Maxillary Nerve	4	2.99531	3.00791	0.01260	443	418	164.85	188.50
Opthalmic Nerve	5	2.99885	3.00221	0.00336	387	362	535.38	612.16
Mandibular Nerve	6	2.99532	2.99899	0.00367	304	279	377.77	431.95
Upper Cervical Spinal Co	7	2.99588	3.05298	0.05710	286	261	22.71	25.97
Olfactory Bulbs	8	3.00439	3.08256	0.07817	284	259	16.46	18.83
Anterior Olfactory Nucleu	9	3.02215	3.06696	0.04481	175	150	16.63	19.02
Frontal Cortex	10	3.02792	3.07082	0.04290	99	74	8.57	9.80
Caudate/Putamen	11	3.05204	3.06257	0.01053	50	25	11.80	13.49
Septal Nucleus	12	3.07949	3.09121	0.01172	38	13	5.51	6.30
Parietal Cortex	13	3.01679	3.05465	0.03786	111	86	11.29	12.91
Hippocampus	14	3.01884	3.05890	0.04006	122	97	12.03	13.76
Thalamus	15	2.97848	3.06799	0.08951	235	210	11.66	13.33
Hypothalamus	16	3.00731	3.03391	0.02660	111	86	16.07	18.37
Midbrain	17	3.00089	3.11360	0.11271	260	235	10.36	11.85
Pons	18	3.03093	3.10186	0.07093	206	181	12.68	14.50
Medulla	19	2.97522	3.08010	0.10488	297	272	12.89	14.74
Cerebellum	20	2.99537	3.24967	0.25430	386	361	7.05	8.07
Superficial Nodes (2)	21	3.00760	3.07827	0.07067	3345	3320	233.45	266.93
Cervical Nodes (2)	22	3.00706	3.02246	0.01540	339	314	101.32	115.85
Thyroid	23	3.01469	3.04404	0.02935	1850	1825	308.99	353.31
Olfactory Epithelium	24	2.97543	3.07835	0.10292	34856	34831	1,681.74	1,922.93
Respiratory Epithelium	25	3.00291	3.07923	0.07632	10224488	10224463	665,725.55	761,200.43
Axillary Nodes (2)	26	2.99475	3.01970	0.02495	169	144	28.68	32.79
Muscle (R, deltoid)	27	2.98186	3.02188	0.04002	172	147	18.25	20.87
Liver (R, superficial lobe)	28	3.06172	3.10324	0.04152	107	82	9.81	11.22
Kidney (L, tip)	29	3.00057	3.04849	0.04792	483	458	47.49	54.31
Lung (R, top lobe)	30	2.98637	3.02226	0.03589	218	193	26.72	30.55
Trachea (near cut-off)	31	2.97688	2.98920	0.01232	82	57	22.99	26.29
Esophagus (near cut-off)	32	2.98304	2.99251	0.00947	66	41	21.51	24.60
Spleen (tip)	33	2.97059	3.01607	0.04548	144	119	13.00	14.87
Heart	34	2.95880	3.02087	0.06207	167	142	11.37	13.00
Urine (fill to line of tube)	35	2.98236	3.30984	0.32748	3312	3287	49.88	57.03
Spinal Dura	36	2.95454	2.96986	0.01532	21972	21947	7,118.84	8,139.79
Lower Cervical Spinal Co	37	2.98492	3.07584	0.09092	129	104	5.68	6.50
Thoracic Spinal Cord	38	2.97064	3.11342	0.14278	4505	4480	155.92	178.28
Lumbar Spinal Cord	39	3.03170	3.18001	0.14831	262	237	7.94	9.08
Blood Sample	40	2.99711	3.21934	0.22223	2614	2589	57.89	66.20
Drug Standard	41	2.97326	2.97623	0.00297	4435788	4435763	7,421,726.77	8,486,112.07
Drug Standard	42	2.98708	2.98967	0.00259	4452756	4452731	8,543,184.24	9,768,403.12
Drug Standard	43	3.00737	3.01003	0.00266	4166333	4166308	7,783,282.85	8,899,520.64
							0.201 cpm/fmol	0.176 cpm/fmol

Auto Calculations		Enter Values	
Total uL of Dose Sol =	62.2	Oxytocin Molecular Weight	1,007
Hot mg =	0.0000	Date of Synthesis =	10/23/07
Cold mg =	0.5162	Hot uCi/uL at Synthesis =	**2.320**
Total mg in Dose Sol =	0.5162	Hot uCi/ug at Synthesis =	**1985.72**
Conc Of Dose Sol (ug/uL) =	8.3011	Cold ug/uL =	15.00
Days after Synthesis Delivered =	9	Desired uCi =	45.0
Decay on Day Delivered =	0.9013	Desired mg =	0.400
Total uCi in Dose Sol on Day Delivered =	58.07	Estimated Desired nmol =	397.1
uCi/uL in Dose Sol on Day Delivered =	0.9339	**Date of Delivery =**	**11/01/07**
mg delivered =	0.400	**uL delivered (actual) =**	**48.2**
uCi delivered =	45.00	**Date of Counting =**	**11/01/07**
Estimated nmol delivered =	397.17	**Dose Calculator**	
Days after Synthesis Counted =	9	uL Hot to Deliver =	21.52
Decay on Day Counted =	0.9013	uL Cold to Deliver =	26.66
Specific Activity from Amersham Specs (cpm/fmol) =	0.201	Total uL to Deliver =	48.19
Avg. Volume of Standard (uL)* =	3.0	Hot to Cold Ratio =	0.81
Avg. CPM-bkgd of Standard =	4,351,601	Total uL to Make =	62.19
Specific Activity from Standards: (CPM/fmol) =	0.176	Dose Solution uL Hot =	27.77
Date of Disposal from Decay Storage =	06/14/09	Dose Solution uL Cold =	34.41

Table A20: Intranasal Oxytocin Rat 40 Data

Oxytocin Rat 40	Route	Com-pound	Volume (uL)	uCi	mg	Time (min)	Perfused @ (min)
Amount Delivered:	IN	Oxytocin	50	45.00	0.400	14:00	30:44

Tissue Description	Tube #	Tube (g)	Tissue + Tube (g)	Tissue Weight (g)	CPM	CPM -25 bkgd	nM (Spec. Act.)	nM (Stan.)
Dorsal Dura	1	3.01569	3.02459	0.00890	239	214	119.49	154.10
Ventral Dura	2	2.99976	3.01121	0.01145	370	345	149.73	193.10
Trigeminal Ganglion	3	3.02003	3.04599	0.02596	1355	1330	254.59	328.34
Maxillary Nerve	4	3.00325	3.01354	0.01029	469	444	214.42	276.53
Opthalmic Nerve	5	2.98240	2.98697	0.00457	406	381	414.29	534.30
Mandibular Nerve	6	2.99682	3.00218	0.00536	396	371	343.96	443.59
Upper Cervical Spinal Cord	7	2.99992	3.05661	0.05669	256	231	20.25	26.11
Olfactory Bulbs	8	2.98390	3.03931	0.05541	2445	2420	217.03	279.90
Anterior Olfactory Nucleus	9	3.00632	3.03645	0.03013	95	70	11.54	14.89
Frontal Cortex	10	3.01982	3.05150	0.03168	92	67	10.51	13.55
Caudate/Putamen	11	3.00384	3.01660	0.01276	132	107	41.67	53.74
Septal Nucleus	12	3.00270	3.01414	0.01144	126	101	43.87	56.58
Parietal Cortex	13	3.01860	3.04934	0.03074	106	81	13.09	16.89
Hippocampus	14	2.99712	3.03032	0.03320	113	88	13.17	16.99
Thalamus	15	2.98416	3.06157	0.07741	167	142	9.12	11.76
Hypothalamus	16	2.99061	3.01591	0.02530	335	310	60.89	78.53
Midbrain	17	2.98396	3.09304	0.10908	529	504	22.96	29.61
Pons	18	3.01817	3.08965	0.07148	319	294	20.44	26.36
Medulla	19	2.99390	3.08689	0.09299	270	245	13.09	16.89
Cerebellum	20	3.01456	3.25957	0.24501	419	394	7.99	10.31
Superficial Nodes (2)	21	3.02781	3.10161	0.07380	2409	2384	160.53	207.03
Cervical Nodes (2)	22	2.97610	2.98942	0.01332	208	183	68.27	88.05
Thyroid	23	2.98509	3.01299	0.02790	1235	1210	215.51	277.94
Olfactory Epithelium	24	3.00488	3.11420	0.10932	1131165	1131140	51,417.36	66,311.65
Respiratory Epithelium	25	2.98318	3.03416	0.05098	1235438	1235413	120,421.88	155,304.99
Axillary Nodes (2)	26	2.97497	3.02501	0.05004	164	139	13.80	17.80
Muscle (R, deltoid)	27	2.98503	3.03066	0.04563	136	111	12.09	15.59
Liver (R, superficial lobe)	28	2.97755	3.03410	0.05655	82	57	5.01	6.46
Kidney (L, tip)	29	2.98206	3.04910	0.06704	366	341	25.28	32.60
Lung (R, top lobe)	30	2.96318	3.00143	0.03825	94	69	8.96	11.56
Trachea (near cut-off)	31	3.05858	3.07972	0.02114	55	30	7.05	9.09
Esophagus (near cut-off)	32	2.96189	2.97910	0.01721	120	95	27.43	35.38
Spleen (tip)	33	2.97236	3.02197	0.04961	233	208	20.83	26.87
Heart	34	2.98193	3.02053	0.03860	80	55	7.08	9.13
Urine (fill to line of tube)	35	2.98238	3.15426	0.17188	980	955	27.61	35.61
Spinal Dura	36	2.97274	2.99373	0.02099	586	561	132.81	171.29
Lower Cervical Spinal Cord	37	2.97158	3.09628	0.12470	73	48	1.91	2.47
Thoracic Spinal Cord	38	2.98810	3.06622	0.07812	121	96	6.11	7.88
Lumbar Spinal Cord	39	2.97419	3.09363	0.11944	134	109	4.53	5.85
Blood Sample	40	3.03828	3.28933	0.25105	1801	1776	35.15	45.34
Drug Standard	41	2.96002	2.96314	0.00312	3623537	3623512	5,771,227.40	7,443,002.87
Drug Standard	42	3.01139	3.01488	0.00349	3906139	3906114	5,561,764.10	7,172,863.46
Drug Standard	43	2.96107	2.96344	0.00237	3625186	3625161	7,601,022.71	9,802,842.61
							0.201 cpm/fmol	0.156 cpm/fmol

Auto Calculations		Enter Values	
Total uL of Dose Sol =	64.0	Oxytocin Molecular Weight	1,007
Hot mg =	0.0000	Date of Synthesis =	11/13/07
Cold mg =	0.5120	Hot uCi/uL at Synthesis =	**2.320**
Total mg in Dose Sol =	0.5120	Hot uCi/ug at Synthesis =	**1985.72**
Conc Of Dose Sol (ug/uL) =	8.0002	Cold ug/uL =	15.00
Days after Synthesis Delivered =	16	Desired uCi =	45.0
Decay on Day Delivered =	0.8313	Desired mg =	0.400
Total uCi in Dose Sol on Day Delivered =	57.60	Estimated Desired nmol =	397.1
uCi/uL in Dose Sol on Day Delivered =	0.9000	**Date of Delivery =**	**11/29/07**
mg delivered =	0.400	**uL delivered (actual) =**	**50.0**
uCi delivered =	45.00	**Date of Counting =**	**11/29/07**
Estimated nmol delivered =	397.16	**Dose Calculator**	
Days after Synthesis Counted =	16	uL Hot to Deliver =	23.33
Decay on Day Counted =	0.8313	uL Cold to Deliver =	26.66
Specific Activity from Amersham Specs (cpm/fmol) =	0.201	Total uL to Deliver =	50.00
Avg. Volume of Standard (uL)* =	3.0	Hot to Cold Ratio =	0.88
Avg. CPM-bkgd of Standard =	3,718,262	Total uL to Make =	64.00
Specific Activity from Standards: (CPM/fmol) =	0.156	Dose Solution uL Hot =	29.87
Date of Disposal from Decay Storage =	07/05/09	Dose Solution uL Cold =	34.13

Table A21: Intranasal Oxytocin Rat 41 Data

Oxytocin Rat 41	Route	Com-pound	Volume (uL)	uCi	mg	Time (min)	Perfused @ (min)
Amount Delivered:	IN	Oxytocin	50	45.00	0.400	14:00	30:50

Tissue Description	Tube #	Tube (g)	Tissue + Tube (g)	Tissue Weight (g)	CPM	CPM -25 bkgd	nM (Spec. Act.)	nM (Stan.)
Dorsal Dura	1	2.99793	3.00791	0.00998	2823	2798	1,393.19	2,221.55
Ventral Dura	2	3.00028	3.01886	0.01858	26934	26909	7,196.89	11,475.98
Trigeminal Ganglion	3	2.97027	2.99758	0.02731	1322	1297	236.00	376.32
Maxillary Nerve	4	3.01236	3.02424	0.01188	796	771	322.50	514.25
Opthalmic Nerve	5	2.99639	3.00033	0.00394	277	252	317.83	506.81
Mandibular Nerve	6	2.97725	2.98120	0.00395	384	359	451.64	720.17
Upper Cervical Spinal Cord	7	3.08289	3.13502	0.05213	729	704	67.11	107.01
Olfactory Bulbs	8	2.98481	3.04740	0.06259	1384	1359	107.90	172.05
Anterior Olfactory Nucleus	9	2.96897	2.99499	0.02602	280	255	48.70	77.66
Frontal Cortex	10	2.99417	3.03297	0.03880	728	703	90.04	143.57
Caudate/Putamen	11	2.97864	2.99151	0.01287	162	137	52.90	84.35
Septal Nucleus	12	2.98499	2.99587	0.01088	242	217	99.11	158.04
Parietal Cortex	13	2.99564	3.03453	0.03889	408	383	48.94	78.04
Hippocampus	14	3.02072	3.05762	0.03690	319	294	39.59	63.13
Thalamus	15	3.00605	3.07811	0.07206	540	515	35.51	56.63
Hypothalamus	16	2.98890	3.01430	0.02540	425	400	78.26	124.79
Midbrain	17	3.00777	3.11253	0.10476	1256	1231	58.39	93.11
Pons	18	2.97528	3.06478	0.08950	1098	1073	59.58	95.00
Medulla	19	3.00766	3.12520	0.11754	1469	1444	61.05	97.35
Cerebellum	20	3.00652	3.25067	0.24415	2924	2899	59.00	94.09
Superficial Nodes (2)	21	2.98374	3.04937	0.06563	7673	7648	579.08	923.39
Cervical Nodes (2)	22	2.99884	3.01278	0.01394	805	780	278.05	443.37
Thyroid	23	3.00782	3.02979	0.02197	67540	67515	15,270.85	24,350.52
Olfactory Epithelium	24	3.01216	3.10629	0.09413	44327	44302	2,338.77	3,729.35
Respiratory Epithelium	25	3.00669	3.05216	0.04547	3256358	3256333	355,874.55	567,468.74
Axillary Nodes (2)	26	2.97564	3.02346	0.04782	1556	1531	159.10	253.69
Muscle (R, deltoid)	27	2.98940	3.04580	0.05640	785	760	66.96	106.78
Liver (R, superficial lobe)	28	2.98693	3.04058	0.05365	1704	1679	155.52	247.98
Kidney (L, tip)	29	2.99113	3.04624	0.05511	3857	3832	345.53	550.98
Lung (R, top lobe)	30	2.97340	3.02115	0.04775	4523	4498	468.10	746.42
Trachea (near cut-off)	31	2.96315	2.98596	0.02281	1418	1393	303.47	483.91
Esophagus (near cut-off)	32	2.97331	2.99214	0.01883	754	729	192.38	306.77
Spleen (tip)	33	2.98950	3.07521	0.08571	7705	7680	445.27	710.02
Heart	34	2.99757	3.04373	0.04616	776	751	80.85	128.92
Urine (fill to line of tube)	35	2.96069	3.18749	0.22680	27013	26988	591.32	942.90
Spinal Dura	36	2.98208	2.99997	0.01789	278	253	70.28	112.06
Lower Cervical Spinal Cord	37	2.97371	3.07232	0.09861	781	756	38.10	60.75
Thoracic Spinal Cord	38	2.98704	3.07119	0.08415	748	723	42.70	68.08
Lumbar Spinal Cord	39	2.98552	3.10660	0.12108	1560	1535	63.00	100.46
Blood Sample	40	2.99286	3.23351	0.24065	44598	44573	920.41	1,467.66
Drug Standard	41	3.06179	3.06449	0.00270	2940894	2940869	5,412,588.28	8,630,779.03
Drug Standard	42	2.97336	2.97594	0.00258	3081681	3081656	5,935,503.00	9,464,605.88
Drug Standard	43	2.96895	2.97199	0.00304	2999364	2999339	4,902,809.83	7,817,898.97
							0.201 cpm/fmol	0.126 cpm/fmol

Auto Calculations		Enter Values	
Total uL of Dose Sol =	64.0	Oxytocin Molecular Weight	1,007
Hot mg =	0.0000	Date of Synthesis =	11/13/07
Cold mg =	0.5120	Hot uCi/uL at Synthesis =	**2.320**
Total mg in Dose Sol =	0.5120	Hot uCi/ug at Synthesis =	**1985.72**
Conc Of Dose Sol (ug/uL) =	8.0002	Cold ug/uL =	15.00
Days after Synthesis Delivered =	16	Desired uCi =	45.0
Decay on Day Delivered =	0.8313	Desired mg =	0.400
Total uCi in Dose Sol on Day Delivered =	57.60	Estimated Desired nmol =	397.1
uCi/uL in Dose Sol on Day Delivered =	0.9000	**Date of Delivery =**	**11/29/07**
mg delivered =	0.400	**uL delivered (actual) =**	**50.0**
uCi delivered =	45.00	**Date of Counting =**	**11/29/07**
Estimated nmol delivered =	397.16	**Dose Calculator**	
Days after Synthesis Counted =	16	uL Hot to Deliver =	23.33
Decay on Day Counted =	0.8313	uL Cold to Deliver =	26.66
Specific Activity from Amersham Specs (cpm/fmol) =	0.201	Total uL to Deliver =	50.00
Avg. Volume of Standard (uL)* =	3.0	Hot to Cold Ratio =	0.88
Avg. CPM-bkgd of Standard =	3,007,288	Total uL to Make =	64.00
Specific Activity from Standards: (CPM/fmol) =	0.126	Dose Solution uL Hot =	29.87
Date of Disposal from Decay Storage =	07/05/09	Dose Solution uL Cold =	34.13

Appendix B – Intranasal Oxytocin with 1% Avicel ® Mucoadhesive Data

Tables

Table B01: Intranasal Oxytocin with Mucoadhesive, Rat 20 Data
Table B02: Intranasal Oxytocin with Mucoadhesive, Rat 21 Data
Table B03: Intranasal Oxytocin with Mucoadhesive, Rat 22 Data
Table B04: Intranasal Oxytocin with Mucoadhesive, Rat 23 Data
Table B05: Intranasal Oxytocin with Mucoadhesive, Rat 24 Data
Table B06: Intranasal Oxytocin with Mucoadhesive, Rat 25 Data
Table B07: Intranasal Oxytocin with Mucoadhesive, Rat 26 Data
Table B08: Intranasal Oxytocin with Mucoadhesive, Rat 27 Data
Table B09: Intranasal Oxytocin with Mucoadhesive, Rat 28 Data
Table B10: Intranasal Oxytocin with Mucoadhesive, Rat 29 Data
Table B11: Intranasal Oxytocin with Mucoadhesive, Rat 32 Data
Table B12: Intranasal Oxytocin with Mucoadhesive, Rat 33 Data
Table B13: Intranasal Oxytocin with Mucoadhesive, Rat 34 Data
Table B14: Intranasal Oxytocin with Mucoadhesive, Rat 35 Data
Table B15: Intranasal Oxytocin with Mucoadhesive, Rat 36 Data
Table B16: Intranasal Oxytocin with Mucoadhesive, Rat 37 Data
Table B17: Intranasal Oxytocin with Mucoadhesive, Rat 38 Data
Table B18: Intranasal Oxytocin with Mucoadhesive, Rat 39 Data

Table B01: Intranasal Oxytocin with 1% Mucoadhesive Rat 20 Data

Oxytocin Rat 20 1% Mucoadhesive	Route	Com-pound	Volume (uL)	uCi	mg	Time (min)	Perfused @ (min)
Amount Delivered:	IN	Oxytocin	49	45.00	0.400	14:00	30:48

Tissue Description	Tube #	Tube (g)	Tissue + Tube (g)	Tissue Weight (g)	CPM	CPM -25 bkgd	nM (Spec. Act.)	nM (Stan.)
Dorsal Dura	1	3.05599	3.06355	0.00756	5616	5591	3,675.03	4,137.14
Ventral Dura	2	2.97742	2.98622	0.00880	328	303	171.10	192.62
Trigeminal Ganglion	3	3.00585	3.03293	0.02708	1070	1045	191.76	215.87
Maxillary Nerve	4	3.00276	3.01157	0.00881	900	875	493.54	555.60
Opthalmic Nerve	5	3.00798	3.01276	0.00478	3963	3938	4,093.93	4,608.72
Mandibular Nerve	6	2.99959	3.00252	0.00293	272	247	418.91	471.59
Upper Cervical Spinal Cord	7	2.99860	3.06902	0.07042	479	454	32.04	36.07
Olfactory Bulbs	8	3.00029	3.07876	0.07847	799	774	49.02	55.18
Anterior Olfactory Nucleus	9	3.00893	3.05280	0.04387	189	164	18.58	20.91
Frontal Cortex	10	2.99241	3.02838	0.03597	145	120	16.58	18.66
Caudate/Putamen	11	2.98903	3.00128	0.01225	58	33	13.39	15.07
Septal Nucleus	12	2.98866	3.00743	0.01877	96	71	18.80	21.16
Parietal Cortex	13	3.00482	3.03872	0.03390	90	65	9.53	10.73
Hippocampus	14	3.01160	3.04908	0.03748	85	60	7.96	8.96
Thalamus	15	2.99986	3.07715	0.07729	174	149	9.58	10.78
Hypothalamus	16	2.99892	3.03155	0.03263	245	220	33.50	37.72
Midbrain	17	3.00713	3.11560	0.10847	412	387	17.73	19.96
Pons	18	3.01357	3.10230	0.08873	249	224	12.54	14.12
Medulla	19	3.02283	3.13237	0.10954	333	308	13.97	15.73
Cerebellum	20	2.99515	3.24980	0.25465	509	484	9.44	10.63
Superficial Nodes (2)	21	3.00818	3.06520	0.05702	2653	2628	229.03	257.83
Cervical Nodes (2)	22	3.01536	3.02760	0.01224	934	909	369.04	415.45
Thyroid	23	3.01850	3.03765	0.01915	1436	1411	366.14	412.18
Olfactory Epithelium	24	3.00826	3.09204	0.08378	30677	30652	1,818.07	2,046.68
Respiratory Epithelium	25	3.00807	3.05351	0.04544	7606834	7606809	831,873.45	936,475.67
Axillary Nodes (2)	26	2.98510	3.03222	0.04712	185	160	16.87	19.00
Muscle (R, deltoid)	27	2.97873	3.03162	0.05289	155	130	12.21	13.75
Liver (R, superficial lobe)	28	2.95973	2.99403	0.03430	147	122	17.67	19.90
Kidney (L, tip)	29	2.98407	3.04523	0.06116	824	799	64.92	73.08
Lung (R, top lobe)	30	2.98606	3.01080	0.02474	177	152	30.53	34.37
Trachea (near cut-off)	31	2.97477	2.99130	0.01653	87	62	18.64	20.98
Esophagus (near cut-off)	32	2.97534	2.98817	0.01283	95	70	27.11	30.52
Spleen (tip)	33	2.96855	3.02257	0.05402	299	274	25.21	28.37
Heart	34	2.98157	3.03719	0.05562	230	205	18.32	20.62
Urine (fill to line of tube)	35	2.98117	3.20921	0.22804	5871	5846	127.39	143.41
Spinal Dura	36	3.01163	3.02460	0.01297	48	23	8.81	9.92
Lower Cervical Spinal Cord	37	2.96450	3.09272	0.12822	173	148	5.74	6.46
Thoracic Spinal Cord	38	2.99392	3.08834	0.09442	105	80	4.21	4.74
Lumbar Spinal Cord	39	2.96464	3.12538	0.16074	97	72	2.23	2.51
Blood Sample	40	3.01196	3.17154	0.15958	2036	2011	62.62	70.50
Drug Standard	41	2.98671	2.98913	0.00242	4198456	4198431	8,621,140.67	9,705,188.21
Drug Standard	42	2.98968	2.99237	0.00269	4368613	4368588	8,070,155.18	9,084,920.19
Drug Standard	43	3.03508	3.03806	0.00298	4418531	4418506	7,368,044.87	8,294,524.47
							0.201 cpm/fmol	0.179 cpm/fmol

Auto Calculations		Enter Values	
Total uL of Dose Sol =	63.2	Oxytocin Molecular Weight	1,007
Hot mg =	0.0000	Date of Synthesis =	10/23/07
Cold mg =	0.5138	Hot uCi/uL at Synthesis =	**2.320**
Total mg in Dose Sol =	0.5138	Hot uCi/ug at Synthesis =	**1985.72**
Conc Of Dose Sol (ug/uL) =	8.1294	Cold ug/uL =	15.00
Days after Synthesis Delivered =	13	Desired uCi =	45.0
Decay on Day Delivered =	0.8606	Desired mg =	0.400
Total uCi in Dose Sol on Day Delivered =	57.80	Estimated Desired nmol =	397.1
uCi/uL in Dose Sol on Day Delivered =	0.9146	**Date of Delivery =**	**11/05/07**
mg delivered =	0.400	**uL delivered (actual) =**	**49.2**
uCi delivered =	45.00	**Date of Counting =**	**11/05/07**
Estimated nmol delivered =	397.11	**Dose Calculator**	
Days after Synthesis Counted =	13	uL Hot to Deliver =	22.54
Decay on Day Counted =	0.8606	uL Cold to Deliver =	26.66
Specific Activity from Amersham Specs (cpm/fmol) =	0.201	Total uL to Deliver =	49.20
Avg. Volume of Standard (uL)* =	3.0	Hot to Cold Ratio =	0.85
Avg. CPM-bkgd of Standard =	4,328,508	Total uL to Make =	63.20
Specific Activity from Standards: (CPM/fmol) =	0.179	Dose Solution uL Hot =	28.95
Date of Disposal from Decay Storage =	06/14/09	Dose Solution uL Cold =	34.25

Table B02: Intranasal Oxytocin with 1% Mucoadhesive Rat 21 Data

**Oxytocin Rat 21 1% Mucoadhesive**	Route	Com-pound	Volume (uL)	uCi	mg	Time (min)	Perfused @ (min)
Amount Delivered:	IN	Oxytocin	49	45.00	0.400	14:09	30:15

Tissue Description	Tube #	Tube (g)	Tissue + Tube (g)	Tissue Weight (g)	CPM	CPM -25 bkgd	nM (Spec. Act.)	nM (Stan.)
Dorsal Dura	1	3.03168	3.03976	0.00808	233	208	127.92	144.01
Ventral Dura	2	2.98387	2.98632	0.00245	232	207	419.85	472.65
Trigeminal Ganglion	3	3.04312	3.06940	0.02628	1514	1489	281.55	316.96
Maxillary Nerve	4	3.01412	3.02912	0.01500	867	842	278.94	314.02
Opthalmic Nerve	5	2.98637	2.98808	0.00171	86	61	177.27	199.56
Mandibular Nerve	6	3.00495	3.00950	0.00455	431	406	443.41	499.17
Upper Cervical Spinal Cord	7	2.99007	3.08064	0.09057	270	245	13.44	15.13
Olfactory Bulbs	8	3.01541	3.09803	0.08262	423	398	23.94	26.95
Anterior Olfactory Nucleus	9	3.01102	3.05191	0.04089	112	87	10.57	11.90
Frontal Cortex	10	2.99878	3.04156	0.04278	218	193	22.42	25.24
Caudate/Putamen	11	3.02605	3.03898	0.01293	61	36	13.84	15.58
Septal Nucleus	12	3.00033	3.01282	0.01249	47	22	8.75	9.85
Parietal Cortex	13	2.98356	3.04015	0.05659	297	272	23.88	26.89
Hippocampus	14	2.99936	3.02596	0.02660	569	544	101.63	114.41
Thalamus	15	2.98311	3.06430	0.08119	366	341	20.87	23.50
Hypothalamus	16	2.99281	3.03425	0.04144	202	177	21.22	23.89
Midbrain	17	2.98923	3.10434	0.11511	315	290	12.52	14.09
Pons	18	3.01015	3.08552	0.07537	135	110	7.25	8.16
Medulla	19	2.99494	3.09868	0.10374	250	225	10.78	12.13
Cerebellum	20	2.99353	3.24292	0.24939	936	911	18.15	20.43
Superficial Nodes (2)	21	3.00190	3.08798	0.08608	3588	3563	205.69	231.55
Cervical Nodes (2)	22	3.01696	3.02806	0.01110	508	483	216.23	243.42
Thyroid	23	3.00139	3.02739	0.02600	1780	1755	335.43	377.60
Olfactory Epithelium	24	2.99459	3.07700	0.08241	72007	71982	4,340.47	4,886.26
Respiratory Epithelium	25	3.00721	3.05975	0.05254	7906400	7906375	747,791.32	841,820.79
Axillary Nodes (2)	26	2.98505	3.03747	0.05242	190	165	15.64	17.61
Muscle (R, deltoid)	27	3.00960	3.06575	0.05615	137	112	9.91	11.16
Liver (R, superficial lobe)	28	2.98985	3.02105	0.03120	121	96	15.29	17.21
Kidney (L, tip)	29	3.00411	3.06033	0.05622	611	586	51.80	58.31
Lung (R, top lobe)	30	2.98670	3.02036	0.03366	127	102	15.06	16.95
Trachea (near cut-off)	31	2.98586	3.00009	0.01423	62	37	12.92	14.55
Esophagus (near cut-off)	32	2.96667	2.98691	0.02024	75	50	12.28	13.82
Spleen (tip)	33	2.99023	3.04292	0.05269	172	147	13.86	15.61
Heart	34	2.96089	3.00579	0.04490	116	91	10.07	11.34
Urine (fill to line of tube)	35	3.00335	3.34015	0.33680	4201	4176	61.61	69.36
Spinal Dura	36	2.97996	2.99782	0.01786	50	25	6.96	7.83
Lower Cervical Spinal Cord	37	2.99794	3.13591	0.13797	156	131	4.72	5.31
Thoracic Spinal Cord	38	2.96608	3.03225	0.06617	77	52	3.91	4.40
Lumbar Spinal Cord	39	2.96142	3.07964	0.11822	94	69	2.90	3.27
Blood Sample	40	2.98097	3.24396	0.26299	2141	2116	39.98	45.01
Drug Standard	41	2.98671	2.98913	0.00242	4198456	4198431	8,621,140.67	9,705,188.21
Drug Standard	42	2.98968	2.99237	0.00269	4368613	4368588	8,070,155.18	9,084,920.19
Drug Standard	43	3.03508	3.03806	0.00298	4418531	4418506	7,368,044.87	8,294,524.47
							0.201 cpm/fmol	0.179 cpm/fmol

**Auto Calculations**		**Enter Values**	
Total uL of Dose Sol =	63.2	Oxytocin Molecular Weight	1,007
Hot mg =	0.0000	Date of Synthesis =	10/23/07
Cold mg =	0.5138	Hot uCi/uL at Synthesis =	**2.320**
Total mg in Dose Sol =	0.5138	Hot uCi/ug at Synthesis =	**1985.72**
Conc Of Dose Sol (ug/uL) =	8.1294	Cold ug/uL =	15.00
Days after Synthesis Delivered =	13	Desired uCi =	45.0
Decay on Day Delivered =	0.8606	Desired mg =	0.400
Total uCi in Dose Sol on Day Delivered =	57.80	Estimated Desired nmol =	397.1
uCi/uL in Dose Sol on Day Delivered =	0.9146	**Date of Delivery =**	**11/05/07**
mg delivered =	0.400	**uL delivered (actual) =**	**49.2**
uCi delivered =	45.00	**Date of Counting =**	**11/05/07**
Estimated nmol delivered =	397.11	**Dose Calculator**	
Days after Synthesis Counted =	13	uL Hot to Deliver =	22.54
Decay on Day Counted =	0.8606	uL Cold to Deliver =	26.66
Specific Activity from Amersham Specs (cpm/fmol) =	0.201	Total uL to Deliver =	49.20
Avg. Volume of Standard (uL)* =	3.0	Hot to Cold Ratio =	0.85
Avg. CPM-bkgd of Standard =	4,328,508	Total uL to Make =	63.20
Specific Activity from Standards: (CPM/fmol) =	0.179	Dose Solution uL Hot =	28.95
Date of Disposal from Decay Storage =	06/14/09	Dose Solution uL Cold =	34.25

Table B03: Intranasal Oxytocin with 1% Mucoadhesive Rat 22 Data

**Oxytocin Rat 22 1% Mucoadhesive**	Route	Com-pound	Volume (uL)	uCi	mg	Time (min)	Perfused @ (min)
Amount Delivered:	IN	Oxytocin	49	45.00	0.400	14:01	30:54

Tissue Description	Tube #	Tube (g)	Tissue + Tube (g)	Tissue Weight (g)	CPM	CPM -25 bkgd	nM (Spec. Act.)	nM (Stan.)
Dorsal Dura	1	2.99965	3.01136	0.01171	3183	3158	1,340.13	1,624.60
Ventral Dura	2	3.00631	3.01105	0.00474	206	181	189.76	230.03
Trigeminal Ganglion	3	-	-	-	-	-	-	-
Maxillary Nerve	4	3.06881	3.08023	0.01142	1223	1198	521.30	631.95
Opthalmic Nerve	5	3.00388	3.00754	0.00366	233	208	282.41	342.35
Mandibular Nerve	6	3.00305	3.00649	0.00344	239	214	309.14	374.76
Upper Cervical Spinal Cord	7	-	-	-	-	-	-	-
Olfactory Bulbs	8	-	-	-	-	-	-	-
Anterior Olfactory Nucleus	9	-	-	-	-	-	-	-
Frontal Cortex	10	-	-	-	-	-	-	-
Caudate/Putamen	11	-	-	-	-	-	-	-
Septal Nucleus	12	-	-	-	-	-	-	-
Parietal Cortex	13	-	-	-	-	-	-	-
Hippocampus	14	-	-	-	-	-	-	-
Thalamus	15	-	-	-	-	-	-	-
Hypothalamus	16	-	-	-	-	-	-	-
Midbrain	17	-	-	-	-	-	-	-
Pons	18	-	-	-	-	-	-	-
Medulla	19	-	-	-	-	-	-	-
Cerebellum	20	-	-	-	-	-	-	-
Superficial Nodes (2)	21	3.00359	3.06589	0.06230	5050	5025	400.81	485.89
Cervical Nodes (2)	22	3.00276	3.01465	0.01189	387	362	151.29	183.41
Thyroid	23	2.99326	3.00732	0.01406	1662	1637	578.57	701.38
Olfactory Epithelium	24	3.06889	3.15659	0.08770	77780	77755	4,405.77	5,340.98
Respiratory Epithelium	25	2.99815	3.09872	0.10057	12165611	12165586	601,115.13	728,713.76
Axillary Nodes (2)	26	2.96352	2.99690	0.03338	110	85	12.65	15.34
Muscle (R, deltoid)	27	3.04280	3.08534	0.04254	90	65	7.59	9.20
Liver (R, superficial lobe)	28	2.97986	3.02637	0.04651	95	70	7.48	9.07
Kidney (L, tip)	29	3.03778	3.08085	0.04307	566	541	62.42	75.67
Lung (R, top lobe)	30	2.98589	3.02190	0.03601	181	156	21.53	26.10
Trachea (near cut-off)	31	3.00535	3.01869	0.01334	196	171	63.70	77.22
Esophagus (near cut-off)	32	3.01151	3.02436	0.01285	69	44	17.02	20.63
Spleen (tip)	33	2.98255	3.02679	0.04424	355	330	37.07	44.94
Heart	34	2.96741	3.00037	0.03296	114	89	13.42	16.27
Urine (fill to line of tube)	35	2.98388	3.40482	0.42094	10148	10123	119.50	144.87
Spinal Dura	36	2.97636	2.99114	0.01478	84	59	19.84	24.05
Lower Cervical Spinal Cord	37	2.97951	3.05705	0.07754	77	52	3.33	4.04
Thoracic Spinal Cord	38	2.97464	3.08862	0.11398	130	105	4.58	5.55
Lumbar Spinal Cord	39	2.97697	3.10175	0.12478	101	76	3.03	3.67
Blood Sample	40	3.01261	3.21175	0.19914	3459	3434	85.69	103.88
Drug Standard	41	2.98686	2.98968	0.00282	4313108	4313083	7,600,318.37	9,213,636.98
Drug Standard	42	2.98104	2.98340	0.00236	3786456	3786431	7,972,804.62	9,665,190.84
Drug Standard	43	3.00139	3.00370	0.00231	3895357	3895332	8,379,644.14	10,158,390.14
							0.201 cpm/fmol	0.166 cpm/fmol

Auto Calculations		Enter Values	
Total uL of Dose Sol =	63.5	Oxytocin Molecular Weight	1,007
Hot mg =	0.0000	Date of Synthesis =	10/23/07
Cold mg =	0.5132	Hot uCi/uL at Synthesis =	**2.320**
Total mg in Dose Sol =	0.5132	Hot uCi/ug at Synthesis =	**1985.72**
Conc Of Dose Sol (ug/uL) =	8.0864	Cold ug/uL =	15.00
Days after Synthesis Delivered =	14	Desired uCi =	45.0
Decay on Day Delivered =	0.8507	Desired mg =	0.400
Total uCi in Dose Sol on Day Delivered =	57.74	Estimated Desired nmol =	397.1
uCi/uL in Dose Sol on Day Delivered =	0.9097	**Date of Delivery =**	**11/06/07**
mg delivered =	0.400	**uL delivered (actual) =**	**49.5**
uCi delivered =	45.00	**Date of Counting =**	**11/06/07**
Estimated nmol delivered =	397.18	**Dose Calculator**	
Days after Synthesis Counted =	14	uL Hot to Deliver =	22.80
Decay on Day Counted =	0.8507	uL Cold to Deliver =	26.66
Specific Activity from Amersham Specs (cpm/fmol) =	0.201	Total uL to Deliver =	49.47
Avg. Volume of Standard (uL)* =	3.0	Hot to Cold Ratio =	0.86
Avg. CPM-bkgd of Standard =	3,998,282	Total uL to Make =	63.47
Specific Activity from Standards: (CPM/fmol) =	0.166	Dose Solution uL Hot =	29.25
Date of Disposal from Decay Storage =	06/14/09	Dose Solution uL Cold =	34.21

Table B04: Intranasal Oxytocin with 1% Mucoadhesive Rat 23 Data

**Trigemina Rat 23 Mucoadhesive**	Route	Com-pound	Volume (uL)	uCi	mg	Time (min)	Perfused @ (min)
Amount Delivered:	IN	Oxytocin	49	45.00	0.400	14:00	31:00

Tissue Description	Tube #	Tube (g)	Tissue + Tube (g)	Tissue Weight (g)	CPM	CPM -25 bkgd	nM (Spec. Act.)	nM (Stan.)
Dorsal Dura	1	2.98582	2.99724	0.01142	997	972	422.95	512.73
Ventral Dura	2	3.06049	3.06807	0.00758	1804	1779	1,166.27	1,413.84
Trigeminal Ganglion	3	-	-	-	-	-	-	-
Maxillary Nerve	4	3.02559	3.03795	0.01236	2136	2111	848.72	1,028.87
Opthalmic Nerve	5	2.99823	3.00119	0.00296	8545	8520	14,303.46	17,339.65
Mandibular Nerve	6	3.00763	3.01046	0.00283	80	55	96.58	117.08
Upper Cervical Spinal Cord	7	-	-	-	-	-	-	-
Olfactory Bulbs	8	-	-	-	-	-	-	-
Anterior Olfactory Nucleus	9	-	-	-	-	-	-	-
Frontal Cortex	10	-	-	-	-	-	-	-
Caudate/Putamen	11	-	-	-	-	-	-	-
Septal Nucleus	12	-	-	-	-	-	-	-
Parietal Cortex	13	-	-	-	-	-	-	-
Hippocampus	14	-	-	-	-	-	-	-
Thalamus	15	-	-	-	-	-	-	-
Hypothalamus	16	-	-	-	-	-	-	-
Midbrain	17	-	-	-	-	-	-	-
Pons	18	-	-	-	-	-	-	-
Medulla	19	-	-	-	-	-	-	-
Cerebellum	20	-	-	-	-	-	-	-
Superficial Nodes (2)	21	3.02616	3.09234	0.06618	6938	6913	519.08	629.26
Cervical Nodes (2)	22	2.98011	2.99293	0.01282	4026	4001	1,550.86	1,880.07
Thyroid	23	3.00222	3.03193	0.02971	3348	3323	555.80	673.78
Olfactory Epithelium	24	3.05125	3.16680	0.11555	75128	75103	3,229.84	3,915.43
Respiratory Epithelium	25	2.98944	3.04809	0.05865	9422413	9422388	798,336.61	967,799.42
Axillary Nodes (2)	26	2.98499	3.02167	0.03668	828	803	108.79	131.88
Muscle (R, deltoid)	27	2.96440	3.02878	0.06438	219	194	14.97	18.15
Liver (R, superficial lobe)	28	2.98408	3.02615	0.04207	143	118	13.94	16.90
Kidney (L, tip)	29	3.00856	3.05706	0.04850	605	580	59.43	72.04
Lung (R, top lobe)	30	3.01013	3.05282	0.04269	679	654	76.13	92.29
Trachea (near cut-off)	31	2.95869	2.97296	0.01427	79	54	18.80	22.80
Esophagus (near cut-off)	32	3.00569	3.02089	0.01520	82	57	18.63	22.59
Spleen (tip)	33	2.97859	3.03186	0.05327	275	250	23.32	28.27
Heart	34	2.98641	3.05754	0.07113	204	179	12.51	15.16
Urine (fill to line of tube)	35	2.99160	3.33452	0.34292	5785	5760	83.47	101.19
Spinal Dura	36	2.99243	3.01023	0.01780	64	39	10.89	13.20
Lower Cervical Spinal Cord	37	2.96120	3.06573	0.10453	112	87	4.14	5.01
Thoracic Spinal Cord	38	2.96865	3.04466	0.07601	83	58	3.79	4.60
Lumbar Spinal Cord	39	2.99066	3.13569	0.14503	139	114	3.91	4.74
Blood Sample	40	3.00522	3.29062	0.28540	5666	5641	98.22	119.07
Drug Standard	41	2.98686	2.98968	0.00282	4313108	4313083	7,600,318.37	9,213,636.98
Drug Standard	42	2.98104	2.98340	0.00236	3786456	3786431	7,972,804.62	9,665,190.84
Drug Standard	43	3.00139	3.00370	0.00231	3895357	3895332	8,379,644.14	10,158,390.14
							0.201 cpm/fmol	0.166 cpm/fmol

**Auto Calculations**		**Enter Values**	
Total uL of Dose Sol =	63.5	Oxytocin Molecular Weight	1,007
Hot mg =	0.0000	Date of Synthesis =	10/23/07
Cold mg =	0.5132	Hot uCi/uL at Synthesis =	**2.320**
Total mg in Dose Sol =	0.5132	Hot uCi/ug at Synthesis =	**1985.72**
Conc Of Dose Sol (ug/uL) =	8.0864	Cold ug/uL =	15.00
Days after Synthesis Delivered =	14	Desired uCi =	45.0
Decay on Day Delivered =	0.8507	Desired mg =	0.400
Total uCi in Dose Sol on Day Delivered =	57.74	Estimated Desired nmol =	397.1
uCi/uL in Dose Sol on Day Delivered =	0.9097	**Date of Delivery =**	**11/06/07**
mg delivered =	0.400	**uL delivered (actual) =**	**49.47**
uCi delivered =	45.00	**Date of Counting =**	**11/06/07**
Estimated nmol delivered =	397.18	**Dose Calculator**	
Days after Synthesis Counted =	14	uL Hot to Deliver =	22.80
Decay on Day Counted =	0.8507	uL Cold to Deliver =	26.66
Specific Activity from Amersham Specs (cpm/fmol) =	0.201	Total uL to Deliver =	49.47
Avg. Volume of Standard (uL)* =	3.0	Hot to Cold Ratio =	0.86
Avg. CPM-bkgd of Standard =	3,998,282	Total uL to Make =	63.47
Specific Activity from Standards: (CPM/fmol) =	0.166	Dose Solution uL Hot =	29.25
Date of Disposal from Decay Storage =	06/14/09	Dose Solution uL Cold =	34.21

Table B05: Intranasal Oxytocin with 1% Mucoadhesive Rat 24 Data

Oxytocin Rat 24 1% Mucoadhesive	Route	Com-pound	Volume (uL)	uCi	mg	Time (min)	Perfused @ (min)
Amount Delivered:	IN	Oxytocin	50	45.00	0.400	14:01	30:49

Tissue Description	Tube #	Tube (g)	Tissue + Tube (g)	Tissue Weight (g)	CPM	CPM -25 bkgd	nM (Spec. Act.)	nM (Stan.)
Dorsal Dura	1	2.99937	3.01807	0.01870	92529	92504	24,581.70	30,678.79
Ventral Dura	2	3.00429	3.01351	0.00922	2851	2826	1,523.12	1,900.91
Trigeminal Ganglion	3	3.00626	3.03313	0.02687	3801	3776	698.32	871.53
Maxillary Nerve	4	2.99649	3.00988	0.01339	5089	5064	1,879.34	2,345.48
Opthalmic Nerve	5	2.97717	2.97992	0.00275	149	124	224.07	279.65
Mandibular Nerve	6	3.00318	3.00751	0.00433	1069	1044	1,198.13	1,495.31
Upper Cervical Spinal Cord	7	2.99515	3.01624	0.02109	4207	4182	985.37	1,229.78
Olfactory Bulbs	8	2.99619	3.05790	0.06171	40944	40919	3,295.05	4,112.34
Anterior Olfactory Nucleus	9	3.00165	3.03138	0.02973	1437	1412	236.01	294.55
Frontal Cortex	10	2.99054	3.02533	0.03479	12062	12037	1,719.32	2,145.77
Caudate/Putamen	11	2.99063	3.00210	0.01147	366	341	147.74	184.38
Septal Nucleus	12	3.00755	3.02014	0.01259	219	194	76.57	95.56
Parietal Cortex	13	3.00010	3.04152	0.04142	2482	2457	294.77	367.89
Hippocampus	14	2.98032	3.01289	0.03257	315	290	44.25	55.22
Thalamus	15	2.99894	3.06547	0.06653	688	663	49.52	61.80
Hypothalamus	16	3.00174	3.03521	0.03347	472	447	66.37	82.83
Midbrain	17	2.98951	3.08513	0.09562	937	912	47.40	59.15
Pons	18	2.99955	3.08111	0.08156	1119	1094	66.66	83.19
Medulla	19	3.06294	3.15041	0.08747	928	903	51.30	64.02
Cerebellum	20	2.98832	3.21646	0.22814	2958	2933	63.89	79.73
Superficial Nodes (2)	21	2.99955	3.05503	0.05548	3159	3134	280.71	350.33
Cervical Nodes (2)	22	3.00281	3.01324	0.01043	670	645	307.30	383.53
Thyroid	23	3.02371	3.04179	0.01808	4015	3990	1,096.65	1,368.65
Olfactory Epithelium	24	2.98884	3.09191	0.10307	524964	524939	25,308.69	31,586.09
Respiratory Epithelium	25	3.00320	3.06993	0.06673	7607904	7607879	566,546.48	707,068.91
Axillary Nodes (2)	26	3.00937	3.04714	0.03777	239	214	28.16	35.14
Muscle (R, deltoid)	27	2.96065	2.99832	0.03767	410	385	50.79	63.38
Liver (R, superficial lobe)	28	3.00536	3.04689	0.04153	280	255	30.51	38.08
Kidney (L, tip)	29	2.98661	3.05080	0.06419	850	825	63.87	79.71
Lung (R, top lobe)	30	2.99072	3.03469	0.04397	368	343	38.76	48.38
Trachea (near cut-off)	31	2.98443	3.00388	0.01945	159	134	34.24	42.73
Esophagus (near cut-off)	32	2.98392	2.99629	0.01237	174	149	59.86	74.70
Spleen (tip)	33	2.98241	3.03706	0.05465	317	292	26.55	33.14
Heart	34	2.98244	3.03869	0.05625	622	597	52.74	65.82
Urine (fill to line of tube)	35	2.98080	3.19902	0.21822	5262	5237	119.26	148.84
Spinal Dura	36	3.01043	3.02924	0.01881	206	181	47.82	59.68
Lower Cervical Spinal Cord	37	2.98939	3.13236	0.14297	553	528	18.35	22.90
Thoracic Spinal Cord	38	2.99668	3.07345	0.07677	106	81	5.24	6.54
Lumbar Spinal Cord	39	2.98741	3.13594	0.14853	178	153	5.12	6.39
Blood Sample	40	3.01451	3.26334	0.24883	4275	4250	84.87	105.93
Drug Standard	41	2.97243	2.97553	0.00310	4106874	4106849	6,583,246.52	8,216,111.27
Drug Standard	42	2.97523	2.97759	0.00236	3553839	3553814	7,483,000.40	9,339,034.12
Drug Standard	43	2.98994	2.99245	0.00251	3866330	3866305	7,654,476.02	9,553,041.42
							0.201 cpm/fmol	0.161 cpm/fmol

Auto Calculations		Enter Values	
Total uL of Dose Sol =	64.0	Oxytocin Molecular Weight	1,007
Hot mg =	0.0000	Date of Synthesis =	10/23/07
Cold mg =	0.5120	Hot uCi/uL at Synthesis =	**2.320**
Total mg in Dose Sol =	0.5120	Hot uCi/ug at Synthesis =	**1985.72**
Conc Of Dose Sol (ug/uL) =	8.0002	Cold ug/uL =	15.00
Days after Synthesis Delivered =	16	Desired uCi =	45.0
Decay on Day Delivered =	0.8313	Desired mg =	0.400
Total uCi in Dose Sol on Day Delivered =	57.60	Estimated Desired nmol =	397.1
uCi/uL in Dose Sol on Day Delivered =	0.9000	**Date of Delivery =**	**11/08/07**
mg delivered =	0.400	**uL delivered (actual) =**	**50.0**
uCi delivered =	45.00	**Date of Counting =**	**11/08/07**
Estimated nmol delivered =	397.16	**Dose Calculator**	
Days after Synthesis Counted =	16	uL Hot to Deliver =	23.33
Decay on Day Counted =	0.8313	uL Cold to Deliver =	26.66
Specific Activity from Amersham Specs (cpm/fmol) =	0.201	Total uL to Deliver =	50.00
Avg. Volume of Standard (uL)* =	3.0	Hot to Cold Ratio =	0.88
Avg. CPM-bkgd of Standard =	3,842,323	Total uL to Make =	64.00
Specific Activity from Standards: (CPM/fmol) =	0.161	Dose Solution uL Hot =	29.87
Date of Disposal from Decay Storage =	06/14/09	Dose Solution uL Cold =	34.13

Table B06: Intranasal Oxytocin with 1% Mucoadhesive Rat 25 Data

**Oxytocin Rat 25 1% Mucoadhesive**	Route	Com-pound	Volume (uL)	uCi	mg	Time (min)	Perfused @ (min)
Amount Delivered:	IN	Oxytocin	50	45.00	0.400	14:02	30:43

Tissue Description	Tube #	Tube (g)	Tissue + Tube (g)	Tissue Weight (g)	CPM	CPM -25 bkgd	nM (Spec. Act.)	nM (Stan.)
Dorsal Dura	1	3.06509	3.07431	0.00922	1622	1597	860.73	1,074.22
Ventral Dura	2	2.97885	2.98575	0.00690	2400	2375	1,710.44	2,134.68
Trigeminal Ganglion	3	2.99956	3.02011	0.02055	1742	1717	415.19	518.18
Maxillary Nerve	4	3.06364	3.07840	0.01476	1072	1047	352.50	439.93
Opthalmic Nerve	5	2.99799	3.00141	0.00342	250	225	326.93	408.01
Mandibular Nerve	6	3.00477	3.00775	0.00298	274	249	415.22	518.21
Upper Cervical Spinal Cord	7	2.99026	3.01463	0.02437	481	456	92.98	116.05
Olfactory Bulbs	8	2.98140	3.04304	0.06164	3360	3335	268.86	335.55
Anterior Olfactory Nucleus	9	3.01568	3.05813	0.04245	494	469	54.90	68.52
Frontal Cortex	10	3.00780	3.04782	0.04002	1068	1043	129.51	161.63
Caudate/Putamen	11	3.00351	3.01514	0.01163	185	160	68.36	85.32
Septal Nucleus	12	2.99364	3.00563	0.01199	74	49	20.31	25.35
Parietal Cortex	13	3.00208	3.05372	0.05164	400	375	36.09	45.04
Hippocampus	14	2.99440	3.02473	0.03033	1005	980	160.56	200.39
Thalamus	15	3.03251	3.10840	0.07589	164	139	9.10	11.36
Hypothalamus	16	2.99208	3.01043	0.01835	113	88	23.83	29.74
Midbrain	17	3.01488	3.12801	0.11313	531	506	22.23	27.74
Pons	18	3.01302	3.08639	0.07337	1911	1886	127.74	159.42
Medulla	19	3.01183	3.08777	0.07594	607	582	38.08	47.53
Cerebellum	20	3.01252	3.23150	0.21898	828	803	18.22	22.74
Superficial Nodes (2)	21	3.01659	3.07100	0.05441	5113	5088	464.69	579.95
Cervical Nodes (2)	22	2.99075	3.00205	0.01130	1504	1479	650.40	811.73
Thyroid	23	3.01133	3.02734	0.01601	6190	6165	1,913.53	2,388.15
Olfactory Epithelium	24	3.00401	3.13038	0.12637	684765	684740	26,926.18	33,604.77
Respiratory Epithelium	25	3.00664	3.09055	0.08391	8834457	8834432	523,188.26	652,956.40
Axillary Nodes (2)	26	2.98789	3.04656	0.05867	185	160	13.55	16.91
Muscle (R, deltoid)	27	2.98618	3.04030	0.05412	153	128	11.75	14.67
Liver (R, superficial lobe)	28	2.98136	3.04484	0.06348	480	455	35.62	44.45
Kidney (L, tip)	29	2.98182	3.04078	0.05896	884	859	72.40	90.36
Lung (R, top lobe)	30	2.97562	3.02903	0.05341	107	82	7.63	9.52
Trachea (near cut-off)	31	2.98022	2.99267	0.01245	78	53	21.15	26.40
Esophagus (near cut-off)	32	2.96436	2.97566	0.01130	91	66	29.02	36.22
Spleen (tip)	33	2.99041	3.06405	0.07364	321	296	19.97	24.93
Heart	34	2.98204	3.06180	0.07976	167	142	8.85	11.04
Urine (fill to line of tube)	35	2.96215	3.19034	0.22819	7146	7121	155.07	193.54
Spinal Dura	36	2.99410	3.00548	0.01138	72	47	20.52	25.61
Lower Cervical Spinal Cord	37	2.97737	3.10890	0.13153	136	111	4.19	5.23
Thoracic Spinal Cord	38	2.99052	3.07162	0.08110	86	61	3.74	4.66
Lumbar Spinal Cord	39	2.98144	3.10157	0.12013	168	143	5.92	7.38
Blood Sample	40	3.06922	3.37518	0.30596	6088	6063	98.47	122.90
Drug Standard	41	2.97243	2.97553	0.00310	4106874	4106849	6,583,246.52	8,216,111.27
Drug Standard	42	2.97523	2.97759	0.00236	3553839	3553814	7,483,000.40	9,339,034.12
Drug Standard	43	2.98994	2.99245	0.00251	3866330	3866305	7,654,476.02	9,553,041.42
							0.201 cpm/fmol	0.161 cpm/fmol

**Auto Calculations**		**Enter Values**	
Total uL of Dose Sol =	64.0	Oxytocin Molecular Weight	1,007
Hot mg =	0.0000	Date of Synthesis =	10/23/07
Cold mg =	0.5120	Hot uCi/uL at Synthesis =	**2.320**
Total mg in Dose Sol =	0.5120	Hot uCi/ug at Synthesis =	**1985.72**
Conc Of Dose Sol (ug/uL) =	8.0002	Cold ug/uL =	15.00
Days after Synthesis Delivered =	16	Desired uCi =	45.0
Decay on Day Delivered =	0.8313	Desired mg =	0.400
Total uCi in Dose Sol on Day Delivered =	57.60	Estimated Desired nmol =	397.1
uCi/uL in Dose Sol on Day Delivered =	0.9000	**Date of Delivery =**	**11/08/07**
mg delivered =	0.400	**uL delivered (actual) =**	**50.0**
uCi delivered =	45.00	**Date of Counting =**	**11/08/07**
Estimated nmol delivered =	397.16	**Dose Calculator**	
Days after Synthesis Counted =	16	uL Hot to Deliver =	23.33
Decay on Day Counted =	0.8313	uL Cold to Deliver =	26.66
Specific Activity from Amersham Specs (cpm/fmol) =	0.201	Total uL to Deliver =	50.00
Avg. Volume of Standard (uL)* =	3.0	Hot to Cold Ratio =	0.88
Avg. CPM-bkgd of Standard =	3,842,323	Total uL to Make =	64.00
Specific Activity from Standards: (CPM/fmol) =	0.161	Dose Solution uL Hot =	29.87
Date of Disposal from Decay Storage =	06/14/09	Dose Solution uL Cold =	34.13

Table B07: Intranasal Oxytocin with 1% Mucoadhesive Rat 26 Data

**Oxytocin Rat 26 1% Mucoadhesive**	Route	Com-pound	Volume (uL)	uCi	mg	Time (min)	Perfused @ (min)
Amount Delivered:	IN	Oxytocin	51	45.00	0.400	14:00	31:09

Tissue Description	Tube #	Tube (g)	Tissue + Tube (g)	Tissue Weight (g)	CPM	CPM -25 bkgd	nM (Spec. Act.)	nM (Stan.)
Dorsal Dura	1	3.05301	3.06175	0.00874	850	825	469.07	563.77
Ventral Dura	2	3.00241	3.01101	0.00860	221	196	113.25	136.12
Trigeminal Ganglion	3	3.02941	3.04959	0.02018	909	884	217.68	261.63
Maxillary Nerve	4	2.98391	2.99632	0.01241	518	493	197.41	237.26
Opthalmic Nerve	5	2.99237	2.99481	0.00244	105	80	162.93	195.82
Mandibular Nerve	6	2.98942	2.99220	0.00278	121	96	171.60	206.24
Upper Cervical Spinal Cord	7	2.97847	3.03742	0.05895	1119	1094	92.22	110.84
Olfactory Bulbs	8	3.00059	3.06612	0.06553	518	493	37.39	44.93
Anterior Olfactory Nucleus	9	2.99945	3.03377	0.03432	265	240	34.75	41.77
Frontal Cortex	10	2.97630	3.01668	0.04038	2159	2134	262.62	315.63
Caudate/Putamen	11	3.00367	3.01615	0.01248	55	30	11.95	14.36
Septal Nucleus	12	3.02260	3.03416	0.01156	50	25	10.75	12.92
Parietal Cortex	13	2.99867	3.04162	0.04295	90	65	7.52	9.04
Hippocampus	14	2.99596	3.02775	0.03179	92	67	10.47	12.59
Thalamus	15	2.99565	3.06520	0.06955	104	79	5.64	6.78
Hypothalamus	16	3.00210	3.02663	0.02453	122	97	19.65	23.62
Midbrain	17	3.00654	3.09816	0.09162	7311	7286	395.18	474.96
Pons	18	3.00289	3.07210	0.06921	8648	8623	619.13	744.12
Medulla	19	2.98931	3.07256	0.08325	39674	39649	2,366.69	2,844.49
Cerebellum	20	2.99906	3.22635	0.22729	11767	11742	256.72	308.54
Superficial Nodes (2)	21	2.99069	3.03977	0.04908	2479	2454	248.46	298.62
Cervical Nodes (2)	22	3.01167	3.02323	0.01156	175	150	64.48	77.50
Thyroid	23	2.99738	3.01631	0.01893	2955	2930	769.15	924.43
Olfactory Epithelium	24	3.03211	3.11273	0.08062	66379	66354	4,089.94	4,915.64
Respiratory Epithelium	25	3.02664	3.08248	0.05584	9520238	9520213	847,216.36	1,018,256.72
Axillary Nodes (2)	26	2.98223	3.03660	0.05437	251	226	20.66	24.83
Muscle (R, deltoid)	27	2.98712	3.01919	0.03207	127	102	15.80	19.00
Liver (R, superficial lobe)	28	2.98993	3.05224	0.06231	147	122	9.73	11.69
Kidney (L, tip)	29	2.97460	3.02989	0.05529	921	896	80.53	96.79
Lung (R, top lobe)	30	2.97617	3.00797	0.03180	124	99	15.47	18.59
Trachea (near cut-off)	31	2.96170	2.97370	0.01200	118	93	38.51	46.29
Esophagus (near cut-off)	32	2.99086	3.00206	0.01120	101	76	33.72	40.53
Spleen (tip)	33	3.03713	3.09907	0.06194	316	291	23.35	28.06
Heart	34	2.97533	3.02561	0.05028	127	102	10.08	12.12
Urine (fill to line of tube)	35	3.00560	3.13318	0.12758	1140	1115	43.43	52.20
Spinal Dura	36	2.99496	3.00567	0.01071	46	21	9.74	11.71
Lower Cervical Spinal Cord	37	2.97313	3.09295	0.11982	96	71	2.94	3.54
Thoracic Spinal Cord	38	2.97827	3.06142	0.08315	109	84	5.02	6.03
Lumbar Spinal Cord	39	2.98438	3.10733	0.12295	118	93	3.76	4.52
Blood Sample	40	3.00403	3.26335	0.25932	4471	4446	85.20	102.40
Drug Standard	41	2.98173	2.98445	0.00272	4006261	4006236	7,319,150.92	8,796,778.44
Drug Standard	42	2.98482	2.98733	0.00251	3976173	3976148	7,871,942.21	9,461,170.05
Drug Standard	43	2.97320	2.97620	0.00300	3728798	3728773	6,176,433.47	7,423,363.36
							0.201 cpm/fmol	0.167 cpm/fmol

**Auto Calculations**		**Enter Values**	
Total uL of Dose Sol =	65.1	Oxytocin Molecular Weight	1,007
Hot mg =	0.0000	Date of Synthesis =	10/23/07
Cold mg =	0.5095	Hot uCi/uL at Synthesis =	**2.320**
Total mg in Dose Sol =	0.5096	Hot uCi/ug at Synthesis =	**1985.72**
Conc Of Dose Sol (ug/uL) =	7.8275	Cold ug/uL =	15.00
Days after Synthesis Delivered =	20	Desired uCi =	45.0
Decay on Day Delivered =	0.7937	Desired mg =	0.400
Total uCi in Dose Sol on Day Delivered =	57.33	Estimated Desired nmol =	397.1
uCi/uL in Dose Sol on Day Delivered =	0.8806	**Date of Delivery =**	**11/12/07**
mg delivered =	0.400	**uL delivered (actual) =**	**51.1**
uCi delivered =	45.00	**Date of Counting =**	**11/12/07**
Estimated nmol delivered =	397.13	**Dose Calculator**	
Days after Synthesis Counted =	20	uL Hot to Deliver =	24.44
Decay on Day Counted =	0.7937	uL Cold to Deliver =	26.66
Specific Activity from Amersham Specs (cpm/fmol) =	0.201	Total uL to Deliver =	51.10
Avg. Volume of Standard (uL)* =	3.0	Hot to Cold Ratio =	0.92
Avg. CPM-bkgd of Standard =	3,903,719	Total uL to Make =	65.10
Specific Activity from Standards: (CPM/fmol) =	0.167	Dose Solution uL Hot =	31.13
Date of Disposal from Decay Storage =	06/14/09	Dose Solution uL Cold =	33.97

Table B08: Intranasal Oxytocin with 1% Mucoadhesive Rat 27 Data

**Oxytocin Rat 27 1% Mucoadhesive**	Route	Com-pound	Volume (uL)	uCi	mg	Time (min)	Perfused @ (min)
Amount Delivered:	IN	Oxytocin	51	45.00	0.400	14:00	30:44

Tissue Description	Tube #	Tube (g)	Tissue + Tube (g)	Tissue Weight (g)	CPM	CPM -25 bkgd	nM (Spec. Act.)	nM (Stan.)
Dorsal Dura	1	3.00560	3.01523	0.00963	2014	1989	1,026.36	1,233.88
Ventral Dura	2	3.00574	3.01220	0.00646	85	60	46.15	55.49
Trigeminal Ganglion	3	3.00739	3.02555	0.01816	433	408	111.64	134.22
Maxillary Nerve	4	3.02403	3.03877	0.01474	250	225	75.85	91.19
Opthalmic Nerve	5	3.01997	3.02282	0.00285	301	276	481.24	578.53
Mandibular Nerve	6	2.99055	2.99475	0.00420	1025	1000	1,183.16	1,422.38
Upper Cervical Spinal Cord	7	3.00200	3.04222	0.04022	266	241	29.78	35.80
Olfactory Bulbs	8	3.00942	3.08607	0.07665	317	292	18.93	22.76
Anterior Olfactory Nucleus	9	3.01308	3.05551	0.04243	100	75	8.78	10.56
Frontal Cortex	10	2.98853	3.02790	0.03937	91	66	8.33	10.01
Caudate/Putamen	11	2.99918	3.01130	0.01212	59	34	13.94	16.76
Septal Nucleus	12	3.00726	3.01766	0.01040	40	15	7.17	8.62
Parietal Cortex	13	3.00402	3.03103	0.02701	622	597	109.84	132.04
Hippocampus	14	2.99569	3.03246	0.03677	76	51	6.89	8.29
Thalamus	15	2.97721	3.05270	0.07549	149	124	8.16	9.81
Hypothalamus	16	2.99146	3.01107	0.01961	184	159	40.29	48.44
Midbrain	17	3.00193	3.11524	0.11331	153	128	5.61	6.75
Pons	18	3.01505	3.08219	0.06714	527	502	37.15	44.67
Medulla	19	2.98697	3.07230	0.08533	284	259	15.08	18.13
Cerebellum	20	3.00270	3.24745	0.24475	414	389	7.90	9.49
Superficial Nodes (2)	21	2.99193	3.05344	0.06151	1907	1882	152.04	182.78
Cervical Nodes (2)	22	2.99716	3.00390	0.00674	243	218	160.73	193.22
Thyroid	23	3.00783	3.02959	0.02176	6683	6658	1,520.47	1,827.88
Olfactory Epithelium	24	3.00253	3.07620	0.07367	23422	23397	1,578.20	1,897.29
Respiratory Epithelium	25	2.99401	3.03552	0.04151	7436123	7436098	890,195.61	1,070,177.75
Axillary Nodes (2)	26	3.04293	3.08290	0.03997	837	812	100.95	121.36
Muscle (R, deltoid)	27	2.97848	3.01767	0.03919	121	96	12.17	14.63
Liver (R, superficial lobe)	28	2.97309	3.02767	0.05458	142	117	10.65	12.81
Kidney (L, tip)	29	3.00222	3.04575	0.04353	130	105	11.99	14.41
Lung (R, top lobe)	30	2.98715	3.03178	0.04463	159	134	14.92	17.94
Trachea (near cut-off)	31	2.99587	3.01248	0.01661	111	86	25.73	30.93
Esophagus (near cut-off)	32	2.97622	2.99691	0.02069	103	78	18.73	22.52
Spleen (tip)	33	2.97912	3.02506	0.04594	309	284	30.72	36.93
Heart	34	2.96953	3.01544	0.04591	116	91	9.85	11.84
Urine (fill to line of tube)	35	2.97152	3.26962	0.29810	4819	4794	79.92	96.07
Spinal Dura	36	2.96045	2.97384	0.01339	55	30	11.13	13.38
Lower Cervical Spinal Cord	37	3.04703	3.18277	0.13574	98	73	2.67	3.21
Thoracic Spinal Cord	38	2.99367	3.06419	0.07052	80	55	3.88	4.66
Lumbar Spinal Cord	39	3.00774	3.13202	0.12428	92	67	2.68	3.22
Blood Sample	40	3.05948	3.31928	0.25980	3925	3900	74.60	89.68
Drug Standard	41	2.98173	2.98445	0.00272	4002385	4002360	7,312,069.70	8,790,443.63
Drug Standard	42	2.98482	2.98733	0.00251	3981003	3980978	7,881,504.60	9,475,008.41
Drug Standard	43	2.97320	2.97620	0.00300	3724945	3724920	6,170,051.26	7,417,528.83
							0.201 cpm/fmol	0.167 cpm/fmol

**Auto Calculations**		**Enter Values**	
Total uL of Dose Sol =	65.1	Oxytocin Molecular Weight	1,007
Hot mg =	0.0000	Date of Synthesis =	10/23/07
Cold mg =	0.5095	Hot uCi/uL at Synthesis =	**2.320**
Total mg in Dose Sol =	0.5096	Hot uCi/ug at Synthesis =	**1985.72**
Conc Of Dose Sol (ug/uL) =	7.8275	Cold ug/uL =	15.00
Days after Synthesis Delivered =	20	Desired uCi =	45.0
Decay on Day Delivered =	0.7937	Desired mg =	0.400
Total uCi in Dose Sol on Day Delivered =	57.33	Estimated Desired nmol =	397.1
uCi/uL in Dose Sol on Day Delivered =	0.8806	**Date of Delivery =**	**11/12/07**
mg delivered =	0.400	**uL delivered (actual) =**	**51.1**
uCi delivered =	45.00	**Date of Counting =**	**11/12/07**
Estimated nmol delivered =	397.13	**Dose Calculator**	
Days after Synthesis Counted =	20	uL Hot to Deliver =	24.44
Decay on Day Counted =	0.7937	uL Cold to Deliver =	26.66
Specific Activity from Amersham Specs (cpm/fmol) =	0.201	Total uL to Deliver =	51.10
Avg. Volume of Standard (uL)* =	3.0	Hot to Cold Ratio =	0.92
Avg. CPM-bkgd of Standard =	3,902,753	Total uL to Make =	65.10
Specific Activity from Standards: (CPM/fmol) =	0.167	Dose Solution uL Hot =	31.13
Date of Disposal from Decay Storage =	06/14/09	Dose Solution uL Cold =	33.97

Table B09: Intranasal Oxytocin with 1% Mucoadhesive Rat 28 Data

Oxytocin Rat 28 1% Mucoadhesive	Route	Com-pound	Volume (uL)	uCi	mg	Time (min)	Perfused @ (min)
Amount Delivered:	IN	Oxytocin	51	45.00	0.400	14:00	30:45

Tissue Description	Tube #	Tube (g)	Tissue + Tube (g)	Tissue Weight (g)	CPM	CPM -25 bkgd	nM (Spec. Act.)	nM (Stan.)
Dorsal Dura	1	3.02854	3.04022	0.01168	1330	1305	555.21	661.99
Ventral Dura	2	2.99814	3.00656	0.00842	769	744	439.09	523.53
Trigeminal Ganglion	3	-	-	-	-	-	-	-
Maxillary Nerve	4	3.01082	3.01964	0.00882	868	843	474.95	566.29
Opthalmic Nerve	5	3.00718	3.01107	0.00389	133	108	137.96	164.50
Mandibular Nerve	6	3.01185	3.01576	0.00391	206	181	230.04	274.27
Upper Cervical Spinal Cord	7	-	-	-	-	-	-	-
Olfactory Bulbs	8	-	-	-	-	-	-	-
Anterior Olfactory Nucleus	9	-	-	-	-	-	-	-
Frontal Cortex	10	-	-	-	-	-	-	-
Caudate/Putamen	11	-	-	-	-	-	-	-
Septal Nucleus	12	-	-	-	-	-	-	-
Parietal Cortex	13	-	-	-	-	-	-	-
Hippocampus	14	-	-	-	-	-	-	-
Thalamus	15	-	-	-	-	-	-	-
Hypothalamus	16	-	-	-	-	-	-	-
Midbrain	17	-	-	-	-	-	-	-
Pons	18	-	-	-	-	-	-	-
Medulla	19	-	-	-	-	-	-	-
Cerebellum	20	-	-	-	-	-	-	-
Superficial Nodes (2)	21	3.04997	3.14435	0.09438	8853	8828	464.81	554.20
Cervical Nodes (2)	22	3.00824	3.01559	0.00735	752	727	491.52	586.04
Thyroid	23	3.01634	3.03239	0.01605	1771	1746	540.58	644.54
Olfactory Epithelium	24	2.99949	3.09256	0.09307	49468	49443	2,639.90	3,147.58
Respiratory Epithelium	25	3.01904	3.09068	0.07164	9716987	9716962	674,012.58	803,630.94
Axillary Nodes (2)	26	2.97481	3.01752	0.04271	174	149	17.34	20.67
Muscle (R, deltoid)	27	2.99074	3.02378	0.03304	113	88	13.24	15.78
Liver (R, superficial lobe)	28	2.99162	3.04690	0.05528	162	137	12.32	14.68
Kidney (L, tip)	29	2.98106	3.02397	0.04291	1021	996	115.34	137.53
Lung (R, top lobe)	30	2.98104	3.00742	0.02638	186	161	30.33	36.16
Trachea (near cut-off)	31	2.99739	3.01026	0.01287	115	90	34.75	41.43
Esophagus (near cut-off)	32	2.96868	2.98310	0.01442	93	68	23.43	27.94
Spleen (tip)	33	2.96639	3.02766	0.06127	367	342	27.74	33.07
Heart	34	2.97750	3.02805	0.05055	133	108	10.62	12.66
Urine (fill to line of tube)	35	2.97818	3.30223	0.32405	6590	6565	100.67	120.03
Spinal Dura	36	2.96444	2.97801	0.01357	61	36	13.18	15.72
Lower Cervical Spinal Cord	37	2.98888	3.09116	0.10228	140	115	5.59	6.66
Thoracic Spinal Cord	38	3.01242	3.09384	0.08142	331	306	18.68	22.27
Lumbar Spinal Cord	39	3.04109	3.16201	0.12092	140	115	4.73	5.63
Blood Sample	40	3.01237	3.26762	0.25525	3706	3681	71.66	85.44
Drug Standard	41	2.96774	2.97025	0.00251	3937667	3937642	7,795,708.37	9,294,889.50
Drug Standard	42	3.03959	3.04223	0.00264	3795240	3795215	7,143,738.26	8,517,539.99
Drug Standard	43	2.97394	2.97646	0.00252	4007175	4007150	7,901,838.26	9,421,429.07
							0.201 cpm/fmol	0.169 cpm/fmol

Auto Calculations		Enter Values	
Total uL of Dose Sol =	65.4	Oxytocin Molecular Weight	1,007
Hot mg =	0.0000	Date of Synthesis =	10/23/07
Cold mg =	0.5089	Hot uCi/uL at Synthesis =	**2.320**
Total mg in Dose Sol =	0.5090	Hot uCi/ug at Synthesis =	**1985.72**
Conc Of Dose Sol (ug/uL) =	7.7843	Cold ug/uL =	15.00
Days after Synthesis Delivered =	21	Desired uCi =	45.0
Decay on Day Delivered =	0.7846	Desired mg =	0.400
Total uCi in Dose Sol on Day Delivered =	57.26	Estimated Desired nmol =	397.1
uCi/uL in Dose Sol on Day Delivered =	0.8757	**Date of Delivery =**	**11/13/07**
mg delivered =	0.400	**uL delivered (actual) =**	**51.4**
uCi delivered =	45.00	**Date of Counting =**	**11/13/07**
Estimated nmol delivered =	397.18	**Dose Calculator**	
Days after Synthesis Counted =	21	uL Hot to Deliver =	24.72
Decay on Day Counted =	0.7846	uL Cold to Deliver =	26.66
Specific Activity from Amersham Specs (cpm/fmol) =	0.201	Total uL to Deliver =	51.39
Avg. Volume of Standard (uL)* =	3.0	Hot to Cold Ratio =	0.93
Avg. CPM-bkgd of Standard =	3,913,336	Total uL to Make =	65.39
Specific Activity from Standards: (CPM/fmol) =	0.169	Dose Solution uL Hot =	31.46
Date of Disposal from Decay Storage =	06/14/09	Dose Solution uL Cold =	33.93

Table B10: Intranasal Oxytocin with 1% Mucoadhesive Rat 29 Data

**Oxytocin Rat 29 1% Mucoadhesive**	Route	Com-pound	Volume (uL)	uCi	mg	Time (min)	Perfused @ (min)
Amount Delivered:	IN	Oxytocin	51	45.00	0.400	14:00	30:40

Tissue Description	Tube #	Tube (g)	Tissue + Tube (g)	Tissue Weight (g)	CPM	CPM -25 bkgd	nM (Spec. Act.)	nM (Stan.)
Dorsal Dura	1	2.99684	3.00983	0.01299	418	393	150.34	179.25
Ventral Dura	2	3.00642	3.02774	0.02132	10040	10015	2,334.30	2,783.21
Trigeminal Ganglion	3	-	-	-	-	-	-	-
Maxillary Nerve	4	3.05699	3.06579	0.00880	464	439	247.90	295.57
Opthalmic Nerve	5	3.01789	3.02003	0.00214	162	137	318.13	379.30
Mandibular Nerve	6	2.99528	2.99876	0.00348	951	926	1,322.28	1,576.57
Upper Cervical Spinal Cord	7	-	-	-	-	-	-	-
Olfactory Bulbs	8	-	-	-	-	-	-	-
Anterior Olfactory Nucleus	9	-	-	-	-	-	-	-
Frontal Cortex	10	-	-	-	-	-	-	-
Caudate/Putamen	11	-	-	-	-	-	-	-
Septal Nucleus	12	-	-	-	-	-	-	-
Parietal Cortex	13	-	-	-	-	-	-	-
Hippocampus	14	-	-	-	-	-	-	-
Thalamus	15	-	-	-	-	-	-	-
Hypothalamus	16	-	-	-	-	-	-	-
Midbrain	17	-	-	-	-	-	-	-
Pons	18	-	-	-	-	-	-	-
Medulla	19	-	-	-	-	-	-	-
Cerebellum	20	-	-	-	-	-	-	-
Superficial Nodes (2)	21	2.98953	3.06313	0.07360	6070	6045	408.14	486.63
Cervical Nodes (2)	22	2.99467	3.00916	0.01449	1260	1235	423.54	504.99
Thyroid	23	3.00453	3.02027	0.01574	5451	5426	1,713.04	2,042.48
Olfactory Epithelium	24	2.98049	3.10398	0.12349	1208793	1208768	48,641.20	57,995.31
Respiratory Epithelium	25	3.01665	3.02641	0.00976	4560134	4560109	2,321,766.19	2,768,261.61
Axillary Nodes (2)	26	2.97613	3.02635	0.05022	295	270	26.72	31.85
Muscle (R, deltoid)	27	2.98526	3.02542	0.04016	156	131	16.21	19.33
Liver (R, superficial lobe)	28	2.97671	3.03213	0.05542	193	168	15.06	17.96
Kidney (L, tip)	29	2.98235	3.03944	0.05709	527	502	43.70	52.10
Lung (R, top lobe)	30	3.03467	3.08843	0.05376	136	111	10.26	12.23
Trachea (near cut-off)	31	2.96691	2.97983	0.01292	114	89	34.23	40.81
Esophagus (near cut-off)	32	2.97752	2.99263	0.01511	389	364	119.71	142.73
Spleen (tip)	33	2.99562	3.04569	0.05007	178	153	15.18	18.10
Heart	34	2.99046	3.03029	0.03983	129	104	12.98	15.47
Urine (fill to line of tube)	35	2.96763	3.28149	0.31386	13144	13119	207.71	247.65
Spinal Dura	36	2.97386	2.98957	0.01571	82	57	18.03	21.50
Lower Cervical Spinal Cord	37	2.98001	3.08615	0.10614	37	12	0.56	0.67
Thoracic Spinal Cord	38	2.97987	3.06081	0.08094	94	69	4.24	5.05
Lumbar Spinal Cord	39	3.04667	3.16295	0.11628	119	94	4.02	4.79
Blood Sample	40	2.98521	3.24633	0.26112	3478	3453	65.71	78.35
Drug Standard	41	2.96774	2.97025	0.00251	3937667	3937642	7,795,708.37	9,294,889.50
Drug Standard	42	3.03959	3.04223	0.00264	3795240	3795215	7,143,738.26	8,517,539.99
Drug Standard	43	2.97394	2.97646	0.00252	4007175	4007150	7,901,838.26	9,421,429.07
							0.201 cpm/fmol	0.169 cpm/fmol

Auto Calculations		Enter Values	
Total uL of Dose Sol =	65.4	Oxytocin Molecular Weight	1,007
Hot mg =	0.0000	Date of Synthesis =	10/23/07
Cold mg =	0.5089	Hot uCi/uL at Synthesis =	**2.320**
Total mg in Dose Sol =	0.5090	Hot uCi/ug at Synthesis =	**1985.72**
Conc Of Dose Sol (ug/uL) =	7.7843	Cold ug/uL =	15.00
Days after Synthesis Delivered =	21	Desired uCi =	45.0
Decay on Day Delivered =	0.7846	Desired mg =	0.400
Total uCi in Dose Sol on Day Delivered =	57.26	Estimated Desired nmol =	397.1
uCi/uL in Dose Sol on Day Delivered =	0.8757	**Date of Delivery =**	**11/13/07**
mg delivered =	0.400	**uL delivered (actual) =**	**51.4**
uCi delivered =	45.00	**Date of Counting =**	**11/13/07**
Estimated nmol delivered =	397.18	**Dose Calculator**	
Days after Synthesis Counted =	21	uL Hot to Deliver =	24.72
Decay on Day Counted =	0.7846	uL Cold to Deliver =	26.66
Specific Activity from Amersham Specs (cpm/fmol) =	0.201	Total uL to Deliver =	51.39
Avg. Volume of Standard (uL)* =	3.0	Hot to Cold Ratio =	0.93
Avg. CPM-bkgd of Standard =	3,913,336	Total uL to Make =	65.39
Specific Activity from Standards: (CPM/fmol) =	0.169	Dose Solution uL Hot =	31.46
Date of Disposal from Decay Storage =	06/14/09	Dose Solution uL Cold =	33.93

Table B11: Intranasal Oxytocin with 1% Mucoadhesive Rat 32 Data

**Oxytocin Rat 32 1% Mucoadhesive**	Route	Com-pound	Volume (uL)	uCi	mg	Time (min)	Perfused @ (min)
Amount Delivered:	IN	Oxytocin	47	45.00	0.400	14:00	30:39

Tissue Description	Tube #	Tube (g)	Tissue + Tube (g)	Tissue Weight (g)	CPM	CPM -25 bkgd	nM (Spec. Act.)	nM (Stan.)
Dorsal Dura	1	2.98140	2.99434	0.01294	1162	1137	436.64	547.86
Ventral Dura	2	2.99921	3.01666	0.01745	1444	1419	404.09	507.02
Trigeminal Ganglion	3	2.99223	3.01334	0.02111	193	168	39.55	49.62
Maxillary Nerve	4	2.98786	3.00124	0.01338	323	298	110.68	138.87
Opthalmic Nerve	5	3.01659	3.02055	0.00396	98	73	91.61	114.94
Mandibular Nerve	6	3.00759	3.01181	0.00422	104	79	93.03	116.72
Upper Cervical Spinal Cord	7	2.98493	3.02991	0.04498	86	61	6.74	8.46
Olfactory Bulbs	8	2.99195	3.05247	0.06052	1241	1216	99.85	125.28
Anterior Olfactory Nucleus	9	2.99694	3.03363	0.03669	246	221	29.93	37.56
Frontal Cortex	10	2.98358	3.01961	0.03603	175	150	20.69	25.96
Caudate/Putamen	11	2.99463	3.00686	0.01223	302	277	112.55	141.22
Septal Nucleus	12	3.05574	3.06556	0.00982	75	50	25.30	31.75
Parietal Cortex	13	3.01511	3.04389	0.02878	166	141	24.35	30.55
Hippocampus	14	2.99458	3.02413	0.02955	188	163	27.41	34.39
Thalamus	15	3.01629	3.07321	0.05692	133	108	9.43	11.83
Hypothalamus	16	3.01405	3.03970	0.02565	304	279	54.05	67.82
Midbrain	17	3.00673	3.10521	0.09848	149	124	6.26	7.85
Pons	18	2.98752	3.06113	0.07361	604	579	39.09	49.04
Medulla	19	2.98559	3.08690	0.10131	164	139	6.82	8.55
Cerebellum	20	2.99139	3.24645	0.25506	572	547	10.66	13.37
Superficial Nodes (2)	21	2.99174	3.09267	0.10093	3576	3551	174.83	219.37
Cervical Nodes (2)	22	3.06062	3.07226	0.01164	415	390	166.50	208.91
Thyroid	23	3.06897	3.08569	0.01672	1744	1719	510.90	641.03
Olfactory Epithelium	24	3.00586	3.08656	0.08070	58343	58318	3,591.06	4,505.79
Respiratory Epithelium	25	2.98080	3.04637	0.06557	7154403	7154378	542,200.33	680,312.55
Axillary Nodes (2)	26	3.00653	3.05036	0.04383	107	82	9.30	11.66
Muscle (R, deltoid)	27	3.03538	3.07878	0.04340	523	498	57.02	71.55
Liver (R, superficial lobe)	28	2.98626	3.04440	0.05814	88	63	5.38	6.76
Kidney (L, tip)	29	2.96606	3.01908	0.05302	527	502	47.05	59.03
Lung (R, top lobe)	30	2.98851	3.02575	0.03724	243	218	29.09	36.50
Trachea (near cut-off)	31	2.98391	3.00066	0.01675	114	89	26.40	33.13
Esophagus (near cut-off)	32	2.96585	2.98490	0.01905	93	68	17.74	22.26
Spleen (tip)	33	2.98258	3.03913	0.05655	147	122	10.72	13.45
Heart	34	2.98192	3.03410	0.05218	132	107	10.19	12.79
Urine (fill to line of tube)	35	2.98799	3.37290	0.38491	1463	1438	18.56	23.29
Spinal Dura	36	2.98815	3.01138	0.02323	56	31	6.63	8.32
Lower Cervical Spinal Cord	37	2.97841	3.09490	0.11649	93	68	2.90	3.64
Thoracic Spinal Cord	38	2.98476	3.06146	0.07670	74	49	3.17	3.98
Lumbar Spinal Cord	39	2.97759	3.09640	0.11881	95	70	2.93	3.67
Blood Sample	40	2.98162	3.26976	0.28814	2707	2682	46.25	58.04
Drug Standard	41	2.97953	2.98253	0.00300	3980289	3980264	6,593,009.49	8,272,416.83
Drug Standard	42	2.99150	2.99441	0.00291	3995730	3995705	6,823,284.90	8,561,349.25
Drug Standard	43	3.04040	3.04297	0.00257	4104461	4104436	7,936,215.32	9,957,771.37
							0.201 cpm/fmol	0.160 cpm/fmol

**Auto Calculations**		**Enter Values**	
Total uL of Dose Sol =	61.5	Oxytocin Molecular Weight	1,007
Hot mg =	0.0000	Date of Synthesis =	11/13/07
Cold mg =	0.5180	Hot uCi/uL at Synthesis =	**2.320**
Total mg in Dose Sol =	0.5180	Hot uCi/ug at Synthesis =	**1985.72**
Conc Of Dose Sol (ug/uL) =	8.4293	Cold ug/uL =	15.00
Days after Synthesis Delivered =	6	Desired uCi =	45.0
Decay on Day Delivered =	0.9330	Desired mg =	0.400
Total uCi in Dose Sol on Day Delivered =	58.28	Estimated Desired nmol =	397.1
uCi/uL in Dose Sol on Day Delivered =	0.9483	**Date of Delivery =**	**11/19/07**
mg delivered =	0.400	**uL delivered (actual) =**	**47.5**
uCi delivered =	45.00	**Date of Counting =**	**11/19/07**
Estimated nmol delivered =	397.12	**Dose Calculator**	
Days after Synthesis Counted =	6	uL Hot to Deliver =	20.79
Decay on Day Counted =	0.9330	uL Cold to Deliver =	26.67
Specific Activity from Amersham Specs (cpm/fmol) =	0.201	Total uL to Deliver =	47.45
Avg. Volume of Standard (uL)* =	3.0	Hot to Cold Ratio =	0.78
Avg. CPM-bkgd of Standard =	4,026,802	Total uL to Make =	61.45
Specific Activity from Standards: (CPM/fmol) =	0.160	Dose Solution uL Hot =	26.92
Date of Disposal from Decay Storage =	07/05/09	Dose Solution uL Cold =	34.53

Table B12: Intranasal Oxytocin with 1% Mucoadhesive Rat 33 Data

Oxytocin Rat 33 1% Mucoadhesive	Route	Com-pound	Volume (uL)	uCi	mg	Time (min)	Perfused @ (min)
Amount Delivered:	IN	Oxytocin	47	45.00	0.400	14:00	31:07

Tissue Description	Tube #	Tube (g)	Tissue + Tube (g)	Tissue Weight (g)	CPM	CPM -25 bkgd	nM (Spec. Act.)	nM (Stan.)
Dorsal Dura	1	3.00538	3.01932	0.01394	1778	1753	624.90	784.08
Ventral Dura	2	3.00813	3.02480	0.01667	4618	4593	1,369.16	1,717.92
Trigeminal Ganglion	3	2.99480	3.01928	0.02448	334	309	62.72	78.70
Maxillary Nerve	4	2.97678	2.99211	0.01533	851	826	267.75	335.95
Opthalmic Nerve	5	2.98739	2.99155	0.00416	539	514	613.99	770.39
Mandibular Nerve	6	3.00820	3.01339	0.00519	124	99	94.79	118.94
Upper Cervical Spinal Cord	7	3.00643	3.06130	0.05487	865	840	76.07	95.45
Olfactory Bulbs	8	3.00319	3.07517	0.07198	724	699	48.26	60.55
Anterior Olfactory Nucleus	9	2.98669	3.02610	0.03941	511	486	61.28	76.89
Frontal Cortex	10	2.99531	3.03571	0.04040	427	402	49.45	62.04
Caudate/Putamen	11	3.00881	3.02202	0.01321	96	71	26.71	33.51
Septal Nucleus	12	2.99894	3.00814	0.00920	59	34	18.36	23.04
Parietal Cortex	13	3.06349	3.09926	0.03577	184	159	22.09	27.72
Hippocampus	14	3.00703	3.03995	0.03292	91	66	9.96	12.50
Thalamus	15	3.01978	3.09224	0.07246	77	52	3.57	4.47
Hypothalamus	16	3.06433	3.09308	0.02875	91	66	11.41	14.31
Midbrain	17	3.01972	3.12973	0.11001	152	127	5.74	7.20
Pons	18	2.99868	3.06969	0.07101	163	138	9.66	12.12
Medulla	19	2.97709	3.07573	0.09864	217	192	9.67	12.14
Cerebellum	20	3.00362	3.24110	0.23748	338	313	6.55	8.22
Superficial Nodes (2)	21	3.01763	3.09494	0.07731	5204	5179	332.89	417.69
Cervical Nodes (2)	22	2.98837	3.00407	0.01570	353	328	103.82	130.26
Thyroid	23	2.99321	3.01388	0.02067	2397	2372	570.25	715.51
Olfactory Epithelium	24	3.02065	3.14685	0.12620	32668	32643	1,285.36	1,612.77
Respiratory Epithelium	25	3.00032	3.09121	0.09089	12497854	12497829	683,300.21	857,354.52
Axillary Nodes (2)	26	3.03498	3.07488	0.03990	237	212	26.40	33.13
Muscle (R, deltoid)	27	2.96142	3.00230	0.04088	105	80	9.72	12.20
Liver (R, superficial lobe)	28	2.98093	3.04439	0.06346	154	129	10.10	12.67
Kidney (L, tip)	29	3.03978	3.08466	0.04488	349	324	35.87	45.01
Lung (R, top lobe)	30	3.04565	3.09363	0.04798	202	177	18.33	23.00
Trachea (near cut-off)	31	3.04455	3.06165	0.01710	86	61	17.73	22.24
Esophagus (near cut-off)	32	2.97725	2.99681	0.01956	116	91	23.12	29.01
Spleen (tip)	33	2.97612	3.02913	0.05301	206	181	16.97	21.29
Heart	34	2.96575	3.01605	0.05030	154	129	12.74	15.99
Urine (fill to line of tube)	35	3.03774	3.25998	0.22224	2945	2920	65.29	81.92
Spinal Dura	36	2.98318	2.99772	0.01454	51	26	8.89	11.15
Lower Cervical Spinal Cord	37	2.98680	3.13204	0.14524	133	108	3.70	4.64
Thoracic Spinal Cord	38	2.96289	3.04897	0.08608	89	64	3.69	4.64
Lumbar Spinal Cord	39	2.99087	3.11939	0.12852	105	80	3.09	3.88
Blood Sample	40	3.00163	3.26652	0.26489	2696	2671	50.11	62.87
Drug Standard	41	2.97953	2.98253	0.00300	3999599	3999574	6,624,995.06	8,312,553.38
Drug Standard	42	2.99150	2.99441	0.00291	3983176	3983151	6,801,847.00	8,534,454.10
Drug Standard	43	3.04040	3.04297	0.00257	4097700	4097675	7,923,142.45	9,941,372.62
							0.201 cpm/fmol	0.160 cpm/fmol

Auto Calculations		Enter Values	
Total uL of Dose Sol =	61.5	Oxytocin Molecular Weight	1,007
Hot mg =	0.0000	Date of Synthesis =	11/13/07
Cold mg =	0.5180	Hot uCi/uL at Synthesis =	**2.320**
Total mg in Dose Sol =	0.5180	Hot uCi/ug at Synthesis =	**1985.72**
Conc Of Dose Sol (ug/uL) =	8.4293	Cold ug/uL =	15.00
Days after Synthesis Delivered =	6	Desired uCi =	45.0
Decay on Day Delivered =	0.9330	Desired mg =	0.400
Total uCi in Dose Sol on Day Delivered =	58.28	Estimated Desired nmol =	397.1
uCi/uL in Dose Sol on Day Delivered =	0.9483	**Date of Delivery =**	**11/19/07**
mg delivered =	0.400	**uL delivered (actual) =**	**47.5**
uCi delivered =	45.00	**Date of Counting =**	**11/19/07**
Estimated nmol delivered =	397.12	**Dose Calculator**	
Days after Synthesis Counted =	6	uL Hot to Deliver =	20.79
Decay on Day Counted =	0.9330	uL Cold to Deliver =	26.67
Specific Activity from Amersham Specs (cpm/fmol) =	0.201	Total uL to Deliver =	47.45
Avg. Volume of Standard (uL)* =	3.0	Hot to Cold Ratio =	0.78
Avg. CPM-bkgd of Standard =	4,026,800	Total uL to Make =	61.45
Specific Activity from Standards: (CPM/fmol) =	0.160	Dose Solution uL Hot =	26.92
Date of Disposal from Decay Storage =	07/05/09	Dose Solution uL Cold =	34.53

Table B13: Intranasal Oxytocin with 1% Mucoadhesive Rat 34 Data

**Oxytocin Rat 34 1% Mucoadhesive**	Route	Com-pound	Volume (uL)	uCi	mg	Time (min)	Perfused @ (min)
Amount Delivered:	IN	Oxytocin	48	45.00	0.400	14:00	30:20

Tissue Description	Tube #	Tube (g)	Tissue + Tube (g)	Tissue Weight (g)	CPM	CPM -25 bkgd	nM (Spec. Act.)	nM (Stan.)
Dorsal Dura	1	2.99846	3.01147	0.01301	8968	8943	3,415.85	4,251.65
Ventral Dura	2	3.00325	3.01560	0.01235	5633	5608	2,256.49	2,808.62
Trigeminal Ganglion	3	-	-	-	-	-	-	-
Maxillary Nerve	4	3.01262	3.02524	0.01262	1727	1702	670.18	834.17
Opthalmic Nerve	5	2.99920	3.00142	0.00222	224	199	445.44	554.44
Mandibular Nerve	6	3.00827	3.01000	0.00173	349	324	930.66	1,158.38
Upper Cervical Spinal Cord	7	-	-	-	-	-	-	-
Olfactory Bulbs	8	-	-	-	-	-	-	-
Anterior Olfactory Nucleus	9	-	-	-	-	-	-	-
Frontal Cortex	10	-	-	-	-	-	-	-
Caudate/Putamen	11	-	-	-	-	-	-	-
Septal Nucleus	12	-	-	-	-	-	-	-
Parietal Cortex	13	-	-	-	-	-	-	-
Hippocampus	14	-	-	-	-	-	-	-
Thalamus	15	-	-	-	-	-	-	-
Hypothalamus	16	-	-	-	-	-	-	-
Midbrain	17	-	-	-	-	-	-	-
Pons	18	-	-	-	-	-	-	-
Medulla	19	-	-	-	-	-	-	-
Cerebellum	20	-	-	-	-	-	-	-
Superficial Nodes (2)	21	3.06700	3.15850	0.09150	12879	12854	698.09	868.90
Cervical Nodes (2)	22	2.99211	3.00128	0.00917	354	329	178.29	221.91
Thyroid	23	3.00391	3.02240	0.01849	3141	3116	837.44	1,042.35
Olfactory Epithelium	24	3.00238	3.14457	0.14219	178789	178764	6,247.47	7,776.12
Respiratory Epithelium	25	2.98110	3.06518	0.08408	4178345	4178320	246,946.04	307,369.51
Axillary Nodes (2)	26	3.03911	3.08249	0.04338	149	124	14.20	17.68
Muscle (R, deltoid)	27	2.98889	3.03923	0.05034	168	143	14.12	17.57
Liver (R, superficial lobe)	28	3.04488	3.12686	0.08198	163	138	8.36	10.41
Kidney (L, tip)	29	2.97869	3.02524	0.04655	335	310	33.09	41.19
Lung (R, top lobe)	30	2.96596	3.01999	0.05403	160	135	12.42	15.45
Trachea (near cut-off)	31	2.98687	3.00190	0.01503	89	64	21.16	26.34
Esophagus (near cut-off)	32	3.04184	3.05697	0.01513	113	88	28.90	35.97
Spleen (tip)	33	2.98758	3.05178	0.06420	175	150	11.61	14.45
Heart	34	2.99410	3.05546	0.06136	139	114	9.23	11.49
Urine (fill to line of tube)	35	2.98924	3.18432	0.19508	2831	2806	71.48	88.97
Spinal Dura	36	2.98525	3.00954	0.02429	85	60	12.27	15.28
Lower Cervical Spinal Cord	37	2.99144	3.09404	0.10260	97	72	3.49	4.34
Thoracic Spinal Cord	38	2.97610	3.05921	0.08311	100	75	4.48	5.58
Lumbar Spinal Cord	39	2.97351	3.09962	0.12611	114	89	3.51	4.37
Blood Sample	40	3.00250	3.24830	0.24580	2306	2281	46.11	57.40
Drug Standard	41	3.04708	3.04973	0.00265	3981332	3981307	7,465,740.16	9,292,478.79
Drug Standard	42	2.98753	2.99028	0.00275	4118626	4118601	7,442,350.24	9,263,365.75
Drug Standard	43	2.97967	2.98271	0.00304	4016335	4016310	6,565,181.25	8,171,568.55
							0.201 cpm/fmol	0.162 cpm/fmol

**Auto Calculations**		**Enter Values**	
Total uL of Dose Sol =	61.7	Oxytocin Molecular Weight	1,007
Hot mg =	0.0000	Date of Synthesis =	11/13/07
Cold mg =	0.5174	Hot uCi/uL at Synthesis =	**2.320**
Total mg in Dose Sol =	0.5174	Hot uCi/ug at Synthesis =	**1985.72**
Conc Of Dose Sol (ug/uL) =	8.3866	Cold ug/uL =	15.00
Days after Synthesis Delivered =	7	Desired uCi =	45.0
Decay on Day Delivered =	0.9223	Desired mg =	0.400
Total uCi in Dose Sol on Day Delivered =	58.21	Estimated Desired nmol =	397.1
uCi/uL in Dose Sol on Day Delivered =	0.9435	**Date of Delivery =**	**11/20/07**
mg delivered =	0.400	**uL delivered (actual) =**	**47.7**
uCi delivered =	45.00	**Date of Counting =**	**11/20/07**
Estimated nmol delivered =	397.10	**Dose Calculator**	
Days after Synthesis Counted =	7	uL Hot to Deliver =	21.03
Decay on Day Counted =	0.9223	uL Cold to Deliver =	26.67
Specific Activity from Amersham Specs (cpm/fmol) =	0.201	Total uL to Deliver =	47.69
Avg. Volume of Standard (uL)* =	3.0	Hot to Cold Ratio =	0.79
Avg. CPM-bkgd of Standard =	4,038,739	Total uL to Make =	61.69
Specific Activity from Standards: (CPM/fmol) =	0.162	Dose Solution uL Hot =	27.20
Date of Disposal from Decay Storage =	07/05/09	Dose Solution uL Cold =	34.49

Table B14: Intranasal Oxytocin with 1% Mucoadhesive Rat 35 Data

Oxytocin Rat 35 1% Mucoadhesive	Route	Com-pound	Volume (uL)	uCi	mg	Time (min)	Perfused @ (min)
Amount Delivered:	IN	Oxytocin	48	45.00	0.400	14:00	30:49

Tissue Description	Tube #	Tube (g)	Tissue + Tube (g)	Tissue Weight (g)	CPM	CPM -25 bkgd	nM (Spec. Act.)	nM (Stan.)
Dorsal Dura	1	3.01558	3.02946	0.01388	144	119	42.60	53.03
Ventral Dura	2	3.05953	3.06744	0.00791	106	81	50.89	63.34
Trigeminal Ganglion	3	-	-	-	-	-	-	-
Maxillary Nerve	4	2.98315	2.99421	0.01106	579	554	248.91	309.82
Opthalmic Nerve	5	3.00322	3.00597	0.00275	46	21	37.95	47.23
Mandibular Nerve	6	3.01085	3.01444	0.00359	95	70	96.89	120.60
Upper Cervical Spinal Cord	7	-	-	-	-	-	-	-
Olfactory Bulbs	8	-	-	-	-	-	-	-
Anterior Olfactory Nucleus	9	-	-	-	-	-	-	-
Frontal Cortex	10	-	-	-	-	-	-	-
Caudate/Putamen	11	-	-	-	-	-	-	-
Septal Nucleus	12	-	-	-	-	-	-	-
Parietal Cortex	13	-	-	-	-	-	-	-
Hippocampus	14	-	-	-	-	-	-	-
Thalamus	15	-	-	-	-	-	-	-
Hypothalamus	16	-	-	-	-	-	-	-
Midbrain	17	-	-	-	-	-	-	-
Pons	18	-	-	-	-	-	-	-
Medulla	19	-	-	-	-	-	-	-
Cerebellum	20	-	-	-	-	-	-	-
Superficial Nodes (2)	21	3.01269	3.08798	0.07529	1451	1426	94.12	117.15
Cervical Nodes (2)	22	3.00681	3.01806	0.01125	1585	1560	689.07	857.68
Thyroid	23	3.01385	3.04430	0.03045	3218	3193	521.08	648.58
Olfactory Epithelium	24	2.99543	3.09444	0.09901	63068	63043	3,164.11	3,938.31
Respiratory Epithelium	25	3.01308	3.07297	0.05989	9938295	9938270	824,611.82	1,026,380.20
Axillary Nodes (2)	26	3.00578	3.05031	0.04453	890	865	96.53	120.15
Muscle (R, deltoid)	27	2.96694	3.01842	0.05148	479	454	43.82	54.55
Liver (R, superficial lobe)	28	2.96769	3.00431	0.03662	90	65	8.82	10.98
Kidney (L, tip)	29	2.97653	3.03738	0.06085	733	708	57.82	71.97
Lung (R, top lobe)	30	2.99323	3.02963	0.03640	150	125	17.06	21.24
Trachea (near cut-off)	31	2.97837	2.99587	0.01750	92	67	19.03	23.68
Esophagus (near cut-off)	32	2.98391	3.00135	0.01744	81	56	15.96	19.86
Spleen (tip)	33	2.97994	3.05495	0.07501	210	185	12.26	15.25
Heart	34	3.00075	3.06878	0.06803	265	240	17.53	21.82
Urine (fill to line of tube)	35	2.98130	3.17415	0.19285	288	263	6.78	8.44
Spinal Dura	36	2.98065	2.99500	0.01435	68	43	14.89	18.53
Lower Cervical Spinal Cord	37	2.97853	3.11622	0.13769	146	121	4.37	5.44
Thoracic Spinal Cord	38	2.97308	3.04444	0.07136	97	72	5.01	6.24
Lumbar Spinal Cord	39	3.00619	3.12719	0.12100	206	181	7.43	9.25
Blood Sample	40	2.99064	3.22288	0.23224	2771	2746	58.76	73.13
Drug Standard	41	3.04708	3.04973	0.00265	3981332	3981307	7,465,740.16	9,292,478.79
Drug Standard	42	2.98753	2.99028	0.00275	4118626	4118601	7,442,350.24	9,263,365.75
Drug Standard	43	2.97967	2.98271	0.00304	4016335	4016310	6,565,181.25	8,171,568.55
							0.201 cpm/fmol	0.162 cpm/fmol

Auto Calculations		Enter Values	
Total uL of Dose Sol =	61.7	Oxytocin Molecular Weight	1,007
Hot mg =	0.0000	Date of Synthesis =	11/13/07
Cold mg =	0.5174	Hot uCi/uL at Synthesis =	**2.320**
Total mg in Dose Sol =	0.5174	Hot uCi/ug at Synthesis =	**1985.72**
Conc Of Dose Sol (ug/uL) =	8.3866	Cold ug/uL =	15.00
Days after Synthesis Delivered =	7	Desired uCi =	45.0
Decay on Day Delivered =	0.9223	Desired mg =	0.400
Total uCi in Dose Sol on Day Delivered =	58.21	Estimated Desired nmol =	397.1
uCi/uL in Dose Sol on Day Delivered =	0.9435	**Date of Delivery =**	**11/20/07**
mg delivered =	0.400	**uL delivered (actual) =**	**47.7**
uCi delivered =	45.00	**Date of Counting =**	**11/20/07**
Estimated nmol delivered =	397.10	**Dose Calculator**	
Days after Synthesis Counted =	7	uL Hot to Deliver =	21.03
Decay on Day Counted =	0.9223	uL Cold to Deliver =	26.67
Specific Activity from Amersham Specs (cpm/fmol) =	0.201	Total uL to Deliver =	47.69
Avg. Volume of Standard (uL)* =	3.0	Hot to Cold Ratio =	0.79
Avg. CPM-bkgd of Standard =	4,038,739	Total uL to Make =	61.69
Specific Activity from Standards: (CPM/fmol) =	0.162	Dose Solution uL Hot =	27.20
Date of Disposal from Decay Storage =	07/05/09	Dose Solution uL Cold =	34.49

Table B15: Intranasal Oxytocin with 1% Mucoadhesive Rat 36 Data

**Oxytocin Rat 36 1% Mucoadhesive**	Route	Com-pound	Volume (uL)	uCi	mg	Time (min)	Perfused @ (min)
Amount Delivered:	IN	Oxytocin	49	45.00	0.400	14:00	30:43

Tissue Description	Tube #	Tube (g)	Tissue + Tube (g)	Tissue Weight (g)	CPM	CPM -25 bkgd	nM (Spec. Act.)	nM (Stan.)
Dorsal Dura	1	3.02742	3.03508	0.00766	825	800	518.98	674.86
Ventral Dura	2	3.00204	3.01004	0.00800	845	820	509.35	662.34
Trigeminal Ganglion	3	-	-	-	-	-	-	-
Maxillary Nerve	4	3.06447	3.07230	0.00783	300	275	174.53	226.95
Opthalmic Nerve	5	3.02031	3.02319	0.00288	140	115	198.43	258.02
Mandibular Nerve	6	3.06660	3.06854	0.00194	359	334	855.54	1,112.50
Upper Cervical Spinal Cord	7	-	-	-	-	-	-	-
Olfactory Bulbs	8	-	-	-	-	-	-	-
Anterior Olfactory Nucleus	9	-	-	-	-	-	-	-
Frontal Cortex	10	-	-	-	-	-	-	-
Caudate/Putamen	11	-	-	-	-	-	-	-
Septal Nucleus	12	-	-	-	-	-	-	-
Parietal Cortex	13	-	-	-	-	-	-	-
Hippocampus	14	-	-	-	-	-	-	-
Thalamus	15	-	-	-	-	-	-	-
Hypothalamus	16	-	-	-	-	-	-	-
Midbrain	17	-	-	-	-	-	-	-
Pons	18	-	-	-	-	-	-	-
Medulla	19	-	-	-	-	-	-	-
Cerebellum	20	-	-	-	-	-	-	-
Superficial Nodes (2)	21	3.00078	3.07344	0.07266	5558	5533	378.41	492.06
Cervical Nodes (2)	22	3.01141	3.02215	0.01074	2781	2756	1,275.17	1,658.17
Thyroid	23	3.00513	3.03585	0.03072	5295	5270	852.48	1,108.52
Olfactory Epithelium	24	3.00560	3.12757	0.12197	619498	619473	25,238.44	32,818.88
Respiratory Epithelium	25	2.99928	3.06740	0.06812	6310676	6310651	460,354.72	598,623.75
Axillary Nodes (2)	26	3.00020	3.03178	0.03158	117	92	14.48	18.82
Muscle (R, deltoid)	27	3.03946	3.10385	0.06439	139	114	8.80	11.44
Liver (R, superficial lobe)	28	2.99742	3.05755	0.06013	77	52	4.30	5.59
Kidney (L, tip)	29	2.99369	3.06099	0.06730	458	433	31.97	41.57
Lung (R, top lobe)	30	2.98969	3.03693	0.04724	123	98	10.31	13.41
Trachea (near cut-off)	31	2.99247	3.01022	0.01775	97	72	20.16	26.21
Esophagus (near cut-off)	32	3.03445	3.05069	0.01624	84	59	18.05	23.48
Spleen (tip)	33	2.97350	3.05255	0.07905	274	249	15.65	20.35
Heart	34	2.98088	3.03126	0.05038	131	106	10.46	13.60
Urine (fill to line of tube)	35	2.96644	3.20455	0.23811	1232	1207	25.19	32.76
Spinal Dura	36	2.98264	2.99503	0.01239	917	892	357.76	465.21
Lower Cervical Spinal Cord	37	2.97995	3.12040	0.14045	608	583	20.63	26.82
Thoracic Spinal Cord	38	2.98019	3.07721	0.09702	101	76	3.89	5.06
Lumbar Spinal Cord	39	2.96365	3.10621	0.14256	198	173	6.03	7.84
Blood Sample	40	3.05308	3.34235	0.28927	3254	3229	55.47	72.13
Drug Standard	41	2.97304	2.97555	0.00251	3829267	3829242	7,581,099.02	9,858,106.66
Drug Standard	42	2.97524	2.97831	0.00307	3813167	3813142	6,172,167.17	8,025,997.56
Drug Standard	43	2.97974	2.98220	0.00246	3599481	3599456	7,271,011.63	9,454,883.51
							0.201 cpm/fmol	0.155 cpm/fmol

Auto Calculations		Enter Values	
Total uL of Dose Sol =	63.2	Oxytocin Molecular Weight	1,007
Hot mg =	0.0000	Date of Synthesis =	11/13/07
Cold mg =	0.5138	Hot uCi/uL at Synthesis =	**2.320**
Total mg in Dose Sol =	0.5138	Hot uCi/ug at Synthesis =	**1985.72**
Conc Of Dose Sol (ug/uL) =	8.1294	Cold ug/uL =	15.00
Days after Synthesis Delivered =	13	Desired uCi =	45.0
Decay on Day Delivered =	0.8606	Desired mg =	0.400
Total uCi in Dose Sol on Day Delivered =	57.80	Estimated Desired nmol =	397.1
uCi/uL in Dose Sol on Day Delivered =	0.9146	**Date of Delivery =**	**11/26/07**
mg delivered =	0.400	**uL delivered (actual) =**	**49.2**
uCi delivered =	45.00	**Date of Counting =**	**11/26/07**
Estimated nmol delivered =	397.11	**Dose Calculator**	
Days after Synthesis Counted =	13	uL Hot to Deliver =	22.54
Decay on Day Counted =	0.8606	uL Cold to Deliver =	26.66
Specific Activity from Amersham Specs (cpm/fmol) =	0.201	Total uL to Deliver =	49.20
Avg. Volume of Standard (uL)* =	3.0	Hot to Cold Ratio =	0.85
Avg. CPM-bkgd of Standard =	3,747,280	Total uL to Make =	63.20
Specific Activity from Standards: (CPM/fmol) =	0.155	Dose Solution uL Hot =	28.95
Date of Disposal from Decay Storage =	07/05/09	Dose Solution uL Cold =	34.25

Table B16: Intranasal Oxytocin with 1% Mucoadhesive Rat 37 Data

Oxytocin Rat 37 1% Mucoadhesive	Route	Com-pound	Volume (uL)	uCi	mg	Time (min)	Perfused @ (min)
Amount Delivered:	IN	Oxytocin	49	45.00	0.400	14:00	30:45

Tissue Description	Tube #	Tube (g)	Tissue + Tube (g)	Tissue Weight (g)	CPM	CPM -25 bkgd	nM (Spec. Act.)	nM (Stan.)
Dorsal Dura	1	3.05923	3.06996	0.01073	202	177	81.97	106.59
Ventral Dura	2	3.01586	3.03112	0.01526	154	129	42.01	54.62
Trigeminal Ganglion	3	-	-	-	-	-	-	-
Maxillary Nerve	4	2.99890	3.00903	0.01013	196	171	83.88	109.08
Opthalmic Nerve	5	2.99481	2.99649	0.00168	72	47	139.02	180.78
Mandibular Nerve	6	3.00379	3.00737	0.00358	144	119	165.18	214.79
Upper Cervical Spinal Cord	7	-	-	-	-	-	-	-
Olfactory Bulbs	8	-	-	-	-	-	-	-
Anterior Olfactory Nucleus	9	-	-	-	-	-	-	-
Frontal Cortex	10	-	-	-	-	-	-	-
Caudate/Putamen	11	-	-	-	-	-	-	-
Septal Nucleus	12	-	-	-	-	-	-	-
Parietal Cortex	13	-	-	-	-	-	-	-
Hippocampus	14	-	-	-	-	-	-	-
Thalamus	15	-	-	-	-	-	-	-
Hypothalamus	16	-	-	-	-	-	-	-
Midbrain	17	-	-	-	-	-	-	-
Pons	18	-	-	-	-	-	-	-
Medulla	19	-	-	-	-	-	-	-
Cerebellum	20	-	-	-	-	-	-	-
Superficial Nodes (2)	21	3.01510	3.05805	0.04295	2670	2645	306.02	397.94
Cervical Nodes (2)	22	3.00900	3.01738	0.00838	182	157	93.10	121.06
Thyroid	23	3.01560	3.03918	0.02358	1398	1373	289.35	376.25
Olfactory Epithelium	24	2.99270	3.08032	0.08762	27913	27888	1,581.64	2,056.69
Respiratory Epithelium	25	2.99219	3.07092	0.07873	7247048	7247023	457,417.17	594,803.90
Axillary Nodes (2)	26	2.99516	3.03300	0.03784	99	74	9.72	12.64
Muscle (R, deltoid)	27	2.98001	3.02235	0.04234	65	40	4.69	6.10
Liver (R, superficial lobe)	28	2.96703	3.02058	0.05355	118	93	8.63	11.22
Kidney (L, tip)	29	2.97310	3.01929	0.04619	146	121	13.02	16.93
Lung (R, top lobe)	30	2.98463	3.01900	0.03437	105	80	11.57	15.04
Trachea (near cut-off)	31	2.97750	2.99399	0.01649	59	34	10.25	13.32
Esophagus (near cut-off)	32	2.98137	2.99524	0.01387	83	58	20.78	27.02
Spleen (tip)	33	2.97100	3.02746	0.05646	241	216	19.01	24.72
Heart	34	2.97583	3.02643	0.05060	96	71	6.97	9.07
Urine (fill to line of tube)	35	2.98083	3.30202	0.32119	1155	1130	17.48	22.73
Spinal Dura	36	2.97184	2.98093	0.00909	486	461	252.02	327.71
Lower Cervical Spinal Cord	37	2.99540	3.12892	0.13352	1312	1287	47.90	62.29
Thoracic Spinal Cord	38	2.99122	3.06834	0.07712	504	479	30.86	40.13
Lumbar Spinal Cord	39	2.98351	3.13752	0.15401	148	123	3.97	5.16
Blood Sample	40	3.00915	3.30557	0.29642	2216	2191	36.73	47.76
Drug Standard	41	2.97304	2.97555	0.00251	3829267	3829242	7,581,099.02	9,858,106.66
Drug Standard	42	2.97524	2.97831	0.00307	3813167	3813142	6,172,167.17	8,025,997.56
Drug Standard	43	2.97974	2.98220	0.00246	3599481	3599456	7,271,011.63	9,454,883.51
							0.201 cpm/fmol	0.155 cpm/fmol

Auto Calculations		Enter Values	
Total uL of Dose Sol =	63.2	Oxytocin Molecular Weight	1,007
Hot mg =	0.0000	Date of Synthesis =	11/13/07
Cold mg =	0.5138	Hot uCi/uL at Synthesis =	**2.320**
Total mg in Dose Sol =	0.5138	Hot uCi/ug at Synthesis =	**1985.72**
Conc Of Dose Sol (ug/uL) =	8.1294	Cold ug/uL =	15.00
Days after Synthesis Delivered =	13	Desired uCi =	45.0
Decay on Day Delivered =	0.8606	Desired mg =	0.400
Total uCi in Dose Sol on Day Delivered =	57.80	Estimated Desired nmol =	397.1
uCi/uL in Dose Sol on Day Delivered =	0.9146	**Date of Delivery =**	**11/26/07**
mg delivered =	0.400	**uL delivered (actual) =**	**49.2**
uCi delivered =	45.00	**Date of Counting =**	**11/26/07**
Estimated nmol delivered =	397.11	**Dose Calculator**	
Days after Synthesis Counted =	13	uL Hot to Deliver =	22.54
Decay on Day Counted =	0.8606	uL Cold to Deliver =	26.66
Specific Activity from Amersham Specs (cpm/fmol) =	0.201	Total uL to Deliver =	49.20
Avg. Volume of Standard (uL)* =	3.0	Hot to Cold Ratio =	0.85
Avg. CPM-bkgd of Standard =	3,747,280	Total uL to Make =	63.20
Specific Activity from Standards: (CPM/fmol) =	0.155	Dose Solution uL Hot =	28.95
Date of Disposal from Decay Storage =	07/05/09	Dose Solution uL Cold =	34.25

Table B17: Intranasal Oxytocin with 1% Mucoadhesive Rat 38 Data

Oxytocin Rat 38 1% Mucoadhesive	Route	Com-pound	Volume (uL)	uCi	mg	Time (min)	Perfused @ (min)
Amount Delivered:	IN	Oxytocin	49	45.00	0.400	14:00	30:02

Tissue Description	Tube #	Tube (g)	Tissue + Tube (g)	Tissue Weight (g)	CPM	CPM -25 bkgd	nM (Spec. Act.)	nM (Stan.)
Dorsal Dura	1	2.98474	2.99929	0.01455	662	637	217.56	254.73
Ventral Dura	2	2.99941	3.01341	0.01400	3070	3045	1,080.82	1,265.53
Trigeminal Ganglion	3	3.03298	3.05865	0.02567	256	231	44.72	52.36
Maxillary Nerve	4	2.97008	2.98181	0.01173	346	321	135.99	159.23
Opthalmic Nerve	5	2.99184	2.99589	0.00405	98	73	89.57	104.88
Mandibular Nerve	6	2.99436	2.99781	0.00345	120	95	136.84	160.22
Upper Cervical Spinal Cord	7	2.98937	3.04383	0.05446	396	371	33.85	39.64
Olfactory Bulbs	8	3.00233	3.07819	0.07586	943	918	60.13	70.41
Anterior Olfactory Nucleus	9	2.98405	3.02345	0.03940	230	205	25.86	30.27
Frontal Cortex	10	2.98955	3.03140	0.04185	337	312	37.05	43.38
Caudate/Putamen	11	3.01634	3.02734	0.01100	67	42	18.97	22.22
Septal Nucleus	12	3.04853	3.06177	0.01324	238	213	79.94	93.61
Parietal Cortex	13	2.99157	3.03063	0.03906	170	145	18.45	21.60
Hippocampus	14	3.01874	3.04413	0.02539	140	115	22.51	26.35
Thalamus	15	2.99313	3.06766	0.07453	159	134	8.93	10.46
Hypothalamus	16	3.02058	3.04180	0.02122	97	72	16.86	19.74
Midbrain	17	2.99985	3.11840	0.11855	3009	2984	125.08	146.46
Pons	18	3.02153	3.10521	0.08368	389	364	21.62	25.31
Medulla	19	3.00677	3.11416	0.10739	984	959	44.38	51.96
Cerebellum	20	3.01201	3.27714	0.26513	889	864	16.19	18.96
Superficial Nodes (2)	21	2.98474	3.06241	0.07767	3995	3970	254.00	297.41
Cervical Nodes (2)	22	2.98332	2.99117	0.00785	202	177	112.05	131.19
Thyroid	23	2.99828	3.01336	0.01508	1170	1145	377.31	441.79
Olfactory Epithelium	24	3.00884	3.08746	0.07862	18060	18035	1,139.92	1,334.73
Respiratory Epithelium	25	3.01584	3.05986	0.04402	6989496	6989471	789,018.79	923,859.81
Axillary Nodes (2)	26	2.96967	3.00569	0.03602	114	89	12.28	14.38
Muscle (R, deltoid)	27	3.06270	3.11785	0.05515	121	96	8.65	10.13
Liver (R, superficial lobe)	28	2.97121	3.05382	0.08261	131	106	6.38	7.47
Kidney (L, tip)	29	2.97531	3.02455	0.04924	407	382	38.55	45.14
Lung (R, top lobe)	30	3.02748	3.06302	0.03554	133	108	15.10	17.68
Trachea (near cut-off)	31	2.97249	2.99077	0.01828	70	45	12.23	14.32
Esophagus (near cut-off)	32	2.98930	3.00891	0.01961	100	75	19.01	22.25
Spleen (tip)	33	3.06399	3.13008	0.06609	250	225	16.92	19.81
Heart	34	3.02486	3.07096	0.04610	104	79	8.52	9.97
Urine (fill to line of tube)	35	2.98206	3.30298	0.32092	2015	1990	30.81	36.08
Spinal Dura	36	2.98441	3.00182	0.01741	143	118	33.68	39.44
Lower Cervical Spinal Cord	37	2.96513	3.10550	0.14037	89	64	2.27	2.65
Thoracic Spinal Cord	38	3.01140	3.10919	0.09779	110	85	4.32	5.06
Lumbar Spinal Cord	39	2.97403	3.14064	0.16661	122	97	2.89	3.39
Blood Sample	40	3.01345	3.29291	0.27946	2115	2090	37.16	43.51
Drug Standard	41	2.98978	2.99256	0.00278	4227915	4227890	7,557,392.19	8,848,928.54
Drug Standard	42	2.98539	2.98786	0.00247	4123975	4123950	8,296,778.87	9,714,674.24
Drug Standard	43	2.99867	3.00108	0.00241	4066860	4066835	8,385,569.97	9,818,639.49
							0.201 cpm/fmol	0.172 cpm/fmol

Auto Calculations		Enter Values	
Total uL of Dose Sol =	63.5	Oxytocin Molecular Weight	1,007
Hot mg =	0.0000	Date of Synthesis =	11/13/07
Cold mg =	0.5132	Hot uCi/uL at Synthesis =	**2.320**
Total mg in Dose Sol =	0.5132	Hot uCi/ug at Synthesis =	**1985.72**
Conc Of Dose Sol (ug/uL) =	8.0864	Cold ug/uL =	15.00
Days after Synthesis Delivered =	14	Desired uCi =	45.0
Decay on Day Delivered =	0.8507	Desired mg =	0.400
Total uCi in Dose Sol on Day Delivered =	57.74	Estimated Desired nmol =	397.1
uCi/uL in Dose Sol on Day Delivered =	0.9097	**Date of Delivery =**	**11/27/07**
mg delivered =	0.400	**uL delivered (actual) =**	**49.5**
uCi delivered =	45.00	**Date of Counting =**	**11/27/07**
Estimated nmol delivered =	397.18	**Dose Calculator**	
Days after Synthesis Counted =	14	uL Hot to Deliver =	22.80
Decay on Day Counted =	0.8507	uL Cold to Deliver =	26.66
Specific Activity from Amersham Specs (cpm/fmol) =	0.201	Total uL to Deliver =	49.47
Avg. Volume of Standard (uL)* =	3.0	Hot to Cold Ratio =	0.86
Avg. CPM-bkgd of Standard =	4,139,558	Total uL to Make =	63.47
Specific Activity from Standards: (CPM/fmol) =	0.172	Dose Solution uL Hot =	29.25
Date of Disposal from Decay Storage =	07/05/09	Dose Solution uL Cold =	34.21

Table B18: Intranasal Oxytocin with 1% Mucoadhesive Rat 39 Data

**Oxytocin Rat 39 1% Mucoadhesive**	Route	Com-pound	Volume (uL)	uCi	mg	Time (min)	Perfused @ (min)
Amount Delivered:	IN	Oxytocin	49	45.00	0.400	14:00	31:49

Tissue Description	Tube #	Tube (g)	Tissue + Tube (g)	Tissue Weight (g)	CPM	CPM -25 bkgd	nM (Spec. Act.)	nM (Stan.)
Dorsal Dura	1	3.00621	3.01778	0.01157	873	848	364.21	426.46
Ventral Dura	2	2.99566	3.01714	0.02148	3657	3632	840.24	983.84
Trigeminal Ganglion	3	3.01364	3.04400	0.03036	1064	1039	170.06	199.12
Maxillary Nerve	4	2.99029	3.00225	0.01196	1224	1199	498.17	583.31
Opthalmic Nerve	5	3.02048	3.02328	0.00280	2759	2734	4,852.14	5,681.36
Mandibular Nerve	6	3.00348	3.00663	0.00315	2812	2787	4,396.63	5,148.00
Upper Cervical Spinal Cord	7	2.97845	2.99964	0.02119	955	930	218.09	255.37
Olfactory Bulbs	8	3.01077	3.07536	0.06459	2062	2037	156.72	183.50
Anterior Olfactory Nucleus	9	2.97806	3.02146	0.04340	456	431	49.35	57.78
Frontal Cortex	10	3.01557	3.05648	0.04091	599	574	69.72	81.64
Caudate/Putamen	11	2.99604	3.00851	0.01247	205	180	71.73	83.99
Septal Nucleus	12	2.99882	3.00797	0.00915	188	163	88.52	103.65
Parietal Cortex	13	2.97833	3.01871	0.04038	310	285	35.07	41.07
Hippocampus	14	3.01925	3.05865	0.03940	272	247	31.15	36.48
Thalamus	15	2.99178	3.07949	0.08771	184	159	9.01	10.55
Hypothalamus	16	3.01112	3.03307	0.02195	167	142	32.15	37.64
Midbrain	17	3.07464	3.17764	0.10300	668	643	31.02	36.32
Pons	18	3.01117	3.08085	0.06968	452	427	30.45	35.66
Medulla	19	3.00793	3.11945	0.11152	978	953	42.47	49.72
Cerebellum	20	3.01155	3.27643	0.26488	1128	1103	20.69	24.23
Superficial Nodes (2)	21	3.01901	3.10734	0.08833	1927	1902	107.00	125.29
Cervical Nodes (2)	22	3.02369	3.03948	0.01579	2650	2625	826.11	967.30
Thyroid	23	3.01593	3.04068	0.02475	12749	12724	2,554.71	2,991.30
Olfactory Epithelium	24	3.03153	3.13960	0.10807	316034	316009	14,530.73	17,013.99
Respiratory Epithelium	25	3.00003	3.06888	0.06885	9041362	9041337	652,562.01	764,082.96
Axillary Nodes (2)	26	2.97565	3.01336	0.03771	138	113	14.89	17.44
Muscle (R, deltoid)	27	2.97858	3.02721	0.04863	106	81	8.28	9.69
Liver (R, superficial lobe)	28	2.99998	3.06296	0.06298	195	170	13.41	15.71
Kidney (L, tip)	29	2.97578	3.05057	0.07479	468	443	29.43	34.46
Lung (R, top lobe)	30	2.96379	3.00597	0.04218	125	100	11.78	13.79
Trachea (near cut-off)	31	2.96887	2.99071	0.02184	59	34	7.74	9.06
Esophagus (near cut-off)	32	2.99771	3.01379	0.01608	88	63	19.47	22.80
Spleen (tip)	33	2.97245	3.02525	0.05280	257	232	21.83	25.57
Heart	34	3.06389	3.11546	0.05157	217	192	18.50	21.66
Urine (fill to line of tube)	35	3.02442	3.29841	0.27399	1990	1965	35.64	41.73
Spinal Dura	36	2.98032	2.99294	0.01262	1001	976	384.31	449.99
Lower Cervical Spinal Cord	37	2.98111	3.15494	0.17383	691	666	19.04	22.29
Thoracic Spinal Cord	38	2.97135	3.04548	0.07413	251	226	15.15	17.74
Lumbar Spinal Cord	39	3.02607	3.15673	0.13066	344	319	12.13	14.21
Blood Sample	40	2.99658	3.29780	0.30122	3172	3147	51.92	60.79
Drug Standard	41	2.98978	2.99256	0.00278	4227915	4227890	7,557,392.19	8,848,928.54
Drug Standard	42	2.98539	2.98786	0.00247	4123975	4123950	8,296,778.87	9,714,674.24
Drug Standard	43	2.99867	3.00108	0.00241	4066860	4066835	8,385,569.97	9,818,639.49
							0.201 cpm/fmol	0.172 cpm/fmol

**Auto Calculations**		**Enter Values**	
Total uL of Dose Sol =	63.5	Oxytocin Molecular Weight	1,007
Hot mg =	0.0000	Date of Synthesis =	11/13/07
Cold mg =	0.5132	Hot uCi/uL at Synthesis =	**2.320**
Total mg in Dose Sol =	0.5132	Hot uCi/ug at Synthesis =	**1985.72**
Conc Of Dose Sol (ug/uL) =	8.0864	Cold ug/uL =	15.00
Days after Synthesis Delivered =	14	Desired uCi =	45.0
Decay on Day Delivered =	0.8507	Desired mg =	0.400
Total uCi in Dose Sol on Day Delivered =	57.74	Estimated Desired nmol =	397.1
uCi/uL in Dose Sol on Day Delivered =	0.9097	**Date of Delivery =**	**11/27/07**
mg delivered =	0.400	**uL delivered (actual) =**	**49.5**
uCi delivered =	45.00	**Date of Counting =**	**11/27/07**
Estimated nmol delivered =	397.18	**Dose Calculator**	
Days after Synthesis Counted =	14	uL Hot to Deliver =	22.80
Decay on Day Counted =	0.8507	uL Cold to Deliver =	26.66
Specific Activity from Amersham Specs (cpm/fmol) =	0.201	Total uL to Deliver =	49.47
Avg. Volume of Standard (uL)* =	3.0	Hot to Cold Ratio =	0.86
Avg. CPM-bkgd of Standard =	4,139,558	Total uL to Make =	63.47
Specific Activity from Standards: (CPM/fmol) =	0.172	Dose Solution uL Hot =	29.25
Date of Disposal from Decay Storage =	07/05/09	Dose Solution uL Cold =	34.21

Appendix C – Intranasal Oxytocin Extended Time Point (1 h)

Tables

Table C01: Intranasal Oxytocin Rat 30 (1 h sac) Data
Table C02: Intranasal Oxytocin with Mucoadhesive, Rat 31 (1 h sac) Data

Table C01: Intranasal Oxytocin Extended Timepoint Rat 30

Oxytocin Rat 30 *1 Hour SAC*	Route	Com-pound	Volume (uL)	uCi	mg	Time (min)	Perfused @ (min)
Amount Delivered:	IN	Oxytocin	52	45.00	0.400	13:56	60:40

Tissue Description	Tube #	Tube (g)	Tissue + Tube (g)	Tissue Weight (g)	CPM	CPM -25 bkgd	nM (Spec. Act.)	nM (Stan.)
Dorsal Dura	1	3.01212	3.02467	0.01255	822	797	315.58	364.43
Ventral Dura	2	2.98277	2.99391	0.01114	1121	1096	488.90	564.57
Trigeminal Ganglion	3	2.97945	3.00207	0.02262	1213	1188	260.99	301.38
Maxillary Nerve	4	3.01021	3.02404	0.01383	418	393	141.21	163.07
Opthalmic Nerve	5	3.01648	3.01857	0.00209	101	76	180.70	208.67
Mandibular Nerve	6	2.98867	2.99272	0.00405	167	142	174.23	201.20
Upper Cervical Spinal Cord	7	2.98985	3.04365	0.05380	191	166	15.33	17.71
Olfactory Bulbs	8	3.01846	3.08977	0.07131	1024	999	69.62	80.39
Anterior Olfactory Nucleus	9	3.00768	3.04386	0.03618	287	262	35.99	41.56
Frontal Cortex	10	3.00296	3.04059	0.03763	191	166	21.92	25.31
Caudate/Putamen	11	2.98035	2.98968	0.00933	76	51	27.16	31.37
Septal Nucleus	12	2.99064	3.00163	0.01099	71	46	20.80	24.02
Parietal Cortex	13	3.02139	3.06659	0.04520	135	110	12.09	13.97
Hippocampus	14	3.06233	3.10633	0.04400	146	121	13.67	15.78
Thalamus	15	3.00124	3.08777	0.08653	193	168	9.65	11.14
Hypothalamus	16	3.01216	3.03286	0.02070	118	93	22.33	25.78
Midbrain	17	3.00182	3.10442	0.10260	386	361	17.48	20.19
Pons	18	2.99813	3.06686	0.06873	528	503	36.37	42.00
Medulla	19	3.05246	3.15468	0.10222	677	652	31.70	36.60
Cerebellum	20	2.97758	3.24430	0.26672	1300	1275	23.75	27.43
Superficial Nodes (2)	21	3.01092	3.12684	0.11592	2221	2196	94.14	108.71
Cervical Nodes (2)	22	3.00024	3.01274	0.01250	854	829	329.56	380.58
Thyroid	23	2.97579	2.99307	0.01728	8702	8677	2,495.28	2,881.52
Olfactory Epithelium	24	3.00004	3.09845	0.09841	1718249	1718224	86,762.81	100,192.82
Respiratory Epithelium	25	3.00639	3.06132	0.05493	10292795	10292770	931,141.64	1,075,272.95
Axillary Nodes (2)	26	2.96417	3.00723	0.04306	411	386	44.55	51.44
Muscle (R, deltoid)	27	2.97834	3.02953	0.05119	346	321	31.16	35.98
Liver (R, superficial lobe)	28	2.97732	3.02915	0.05183	220	195	18.70	21.59
Kidney (L, tip)	29	2.98160	3.04423	0.06263	1838	1813	143.85	166.12
Lung (R, top lobe)	30	2.96820	3.01629	0.04809	493	468	48.36	55.85
Trachea (near cut-off)	31	2.99400	3.01045	0.01645	325	300	90.63	104.65
Esophagus (near cut-off)	32	2.98586	3.00408	0.01822	211	186	50.73	58.58
Spleen (tip)	33	2.98415	3.04748	0.06333	776	751	58.93	68.05
Heart	34	2.97711	3.04488	0.06777	436	411	30.14	34.80
Urine (fill to line of tube)	35	2.98665	3.21091	0.22426	26437	26412	585.25	675.84
Spinal Dura	36	2.97660	2.99388	0.01728	104	79	22.72	26.23
Lower Cervical Spinal Cord	37	2.97884	3.10630	0.12746	255	230	8.97	10.35
Thoracic Spinal Cord	38	3.04253	3.11859	0.07606	171	146	9.54	11.02
Lumbar Spinal Cord	39	3.00499	3.13956	0.13457	457	432	15.95	18.42
Blood Sample	40	3.00842	3.29540	0.28698	7765	7740	134.02	154.77
Drug Standard	41	2.98690	2.98930	0.00240	4020511	4020486	8,324,542.78	9,613,097.89
Drug Standard	42	2.99074	2.99319	0.00245	3980011	3979986	8,072,508.99	9,322,051.81
Drug Standard	43	3.01152	3.01397	0.00245	3986239	3986214	8,085,141.09	9,336,639.23
							0.201 cpm/fmol	0.174 cpm/fmol

Auto Calculations		Enter Values	
Total uL of Dose Sol =	66.0	Oxytocin Molecular Weight	1,007
Hot mg =	0.0000	Date of Synthesis =	10/23/07
Cold mg =	0.5077	Hot uCi/uL at Synthesis =	**2.320**
Total mg in Dose Sol =	0.5078	Hot uCi/ug at Synthesis =	**1985.72**
Conc Of Dose Sol (ug/uL) =	7.6978	Cold ug/uL =	15.00
Days after Synthesis Delivered =	23	Desired uCi =	45.0
Decay on Day Delivered =	0.7667	Desired mg =	0.400
Total uCi in Dose Sol on Day Delivered =	57.12	Estimated Desired nmol =	397.1
uCi/uL in Dose Sol on Day Delivered =	0.8660	**Date of Delivery =**	**11/15/07**
mg delivered =	0.400	**uL delivered (actual) =**	**52.0**
uCi delivered =	45.00	**Date of Counting =**	**11/15/07**
Estimated nmol delivered =	397.12	**Dose Calculator**	
Days after Synthesis Counted =	23	uL Hot to Deliver =	25.30
Decay on Day Counted =	0.7667	uL Cold to Deliver =	26.66
Specific Activity from Amersham Specs (cpm/fmol) =	0.201	Total uL to Deliver =	51.96
Avg. Volume of Standard (uL)* =	3.0	Hot to Cold Ratio =	0.95
Avg. CPM-bkgd of Standard =	3,995,562	Total uL to Make =	65.96
Specific Activity from Standards: (CPM/fmol) =	0.174	Dose Solution uL Hot =	32.11
Date of Disposal from Decay Storage =	06/14/09	Dose Solution uL Cold =	33.85

Table C02: Intranasal Oxytocin Extended Timepoint Rat 31

**Oxytocin Rat 31 1 h SAC, 1% Mucoadhesive**	Route	Com-pound	Volume (uL)	uCi	mg	Time (min)	Perfused @ (min)
Amount Delivered:	IN	Oxytocin	47	45.00	0.400	14:00	60:36

Tissue Description	Tube #	Tube (g)	Tissue + Tube (g)	Tissue Weight (g)	CPM	CPM -25 bkgd	nM (Spec. Act.)	nM (Stan.)
Dorsal Dura	1	3.01585	3.03105	0.01520	580	555	181.44	232.08
Ventral Dura	2	3.06751	3.07734	0.00983	11110	11085	5,603.70	7,167.69
Trigeminal Ganglion	3	3.00020	3.02339	0.02319	4060	4035	864.64	1,105.96
Maxillary Nerve	4	3.00610	3.01811	0.01201	1042	1017	420.80	538.24
Opthalmic Nerve	5	3.06059	3.06378	0.00319	257	232	361.40	462.27
Mandibular Nerve	6	2.99856	3.00089	0.00233	152	127	270.86	346.45
Upper Cervical Spinal Cord	7	3.05914	3.11639	0.05725	259	234	20.31	25.98
Olfactory Bulbs	8	3.01052	3.07508	0.06456	199	174	13.39	17.13
Anterior Olfactory Nucleus	9	3.01589	3.06457	0.04868	116	91	9.29	11.88
Frontal Cortex	10	3.03250	3.07537	0.04287	467	442	51.23	65.53
Caudate/Putamen	11	3.01412	3.02621	0.01209	70	45	18.50	23.66
Septal Nucleus	12	3.00787	3.01981	0.01194	53	28	11.65	14.91
Parietal Cortex	13	3.00292	3.04754	0.04462	110	85	9.47	12.11
Hippocampus	14	3.01466	3.05196	0.03730	101	76	10.13	12.95
Thalamus	15	3.00008	3.07760	0.07752	110	85	5.45	6.97
Hypothalamus	16	3.02887	3.05473	0.02586	117	92	17.68	22.61
Midbrain	17	2.99932	3.09204	0.09272	207	182	9.75	12.48
Pons	18	2.98010	3.05411	0.07401	212	187	12.56	16.06
Medulla	19	3.01458	3.11926	0.10468	217	192	9.11	11.66
Cerebellum	20	3.00558	3.24884	0.24326	565	540	11.03	14.11
Superficial Nodes (2)	21	3.06299	3.12206	0.05907	1806	1781	149.83	191.64
Cervical Nodes (2)	22	3.01539	3.02843	0.01304	941	916	349.07	446.49
Thyroid	23	2.98144	3.00077	0.01933	11962	11937	3,068.71	3,925.19
Olfactory Epithelium	24	3.00338	3.07505	0.07167	110967	110942	7,692.22	9,839.10
Respiratory Epithelium	25	3.02786	3.08058	0.05272	8946967	8946942	843,319.80	1,078,688.73
Axillary Nodes (2)	26	3.04431	3.09098	0.04667	245	220	23.42	29.96
Muscle (R, deltoid)	27	2.99822	3.04586	0.04764	236	211	22.01	28.15
Liver (R, superficial lobe)	28	2.99160	3.06382	0.07222	465	440	30.28	38.73
Kidney (L, tip)	29	3.04163	3.10006	0.05843	541	516	43.88	56.13
Lung (R, top lobe)	30	2.99049	3.03344	0.04295	174	149	17.24	22.05
Trachea (near cut-off)	31	2.97930	2.99553	0.01623	226	201	61.54	78.72
Esophagus (near cut-off)	32	2.99160	3.00696	0.01536	329	304	98.35	125.80
Spleen (tip)	33	2.98368	3.04439	0.06071	482	457	37.41	47.85
Heart	34	2.98004	3.03317	0.05313	275	250	23.38	29.91
Urine (fill to line of tube)	35	3.03614	3.31410	0.27796	4512	4487	80.22	102.61
Spinal Dura	36	2.98856	3.00994	0.02138	60	35	8.13	10.41
Lower Cervical Spinal Cord	37	2.98816	3.10490	0.11674	117	92	3.92	5.01
Thoracic Spinal Cord	38	2.99823	3.06300	0.06477	98	73	5.60	7.16
Lumbar Spinal Cord	39	3.04144	3.17254	0.13110	147	122	4.62	5.91
Blood Sample	40	2.99872	3.26495	0.26623	4357	4332	80.86	103.43
Drug Standard	41	3.04101	3.04414	0.00313	3899841	3899816	6,191,456.93	7,919,480.62
Drug Standard	42	2.97715	2.98036	0.00321	4039416	4039391	6,253,223.31	7,998,485.88
Drug Standard	43	3.04653	3.04931	0.00278	4150131	4150106	7,418,352.58	9,488,800.48
							0.201 cpm/fmol	0.157 cpm/fmol

Auto Calculations		Enter Values	
Total uL of Dose Sol =	60.5	Oxytocin Molecular Weight	1,007
Hot mg =	0.0000	Date of Synthesis =	11/13/07
Cold mg =	0.5204	Hot uCi/uL at Synthesis =	**2.320**
Total mg in Dose Sol =	0.5204	Hot uCi/ug at Synthesis =	**1985.72**
Conc Of Dose Sol (ug/uL) =	8.5994	Cold ug/uL =	15.00
Days after Synthesis Delivered =	2	Desired uCi =	45.0
Decay on Day Delivered =	0.9772	Desired mg =	0.400
Total uCi in Dose Sol on Day Delivered =	58.54	Estimated Desired nmol =	397.1
uCi/uL in Dose Sol on Day Delivered =	0.9674	**Date of Delivery =**	**11/15/07**
mg delivered =	0.400	**uL delivered (actual) =**	**46.5**
uCi delivered =	45.00	**Date of Counting =**	**11/15/07**
Estimated nmol delivered =	397.10	**Dose Calculator**	
Days after Synthesis Counted =	2	uL Hot to Deliver =	19.85
Decay on Day Counted =	0.9772	uL Cold to Deliver =	26.67
Specific Activity from Amersham Specs (cpm/fmol) =	0.201	Total uL to Deliver =	46.51
Avg. Volume of Standard (uL)* =	3.0	Hot to Cold Ratio =	0.74
Avg. CPM-bkgd of Standard =	4,029,771	Total uL to Make =	60.51
Specific Activity from Standards: (CPM/fmol) =	0.157	Dose Solution uL Hot =	25.82
Date of Disposal from Decay Storage =	07/05/09	Dose Solution uL Cold =	34.69

Appendix D – Autoradiography Images

Figures

Figure D01: Rat 10 Autoradiography Images
Figure D02: Rat 11 Autoradiography Images
Figure D03: Rat 12 Autoradiography Images
Figure D04: Rat 13 Autoradiography Images
Figure D05: Rat 14 Autoradiography Images
Figure D06: Rat 15 Autoradiography Images
Figure D07: Rat 16 Autoradiography Images
Figure D08: Rat 17 Autoradiography Images
Figure D09: Rat 22 Autoradiography Images
Figure D10: Rat 23 Autoradiography Images
Figure D11: Rat 28 Autoradiography Images
Figure D12: Rat 29 Autoradiography Images
Figure D13: Rat 34 Autoradiography Images
Figure D14: Rat 35 Autoradiography Images
Figure D15: Rat 36 Autoradiography Images
Figure D16: Rat 37 Autoradiography Images

Figure D01: Rat 10

Intranasal Delivery of Oxytocin (labeled and unlabeled)

Delivered 51 ul over 14 min; 45 uCi, 0.4 mg, 397 nmol

Down on 10/22/07, developed on 11/13/07.

Slices measured from Bregma.

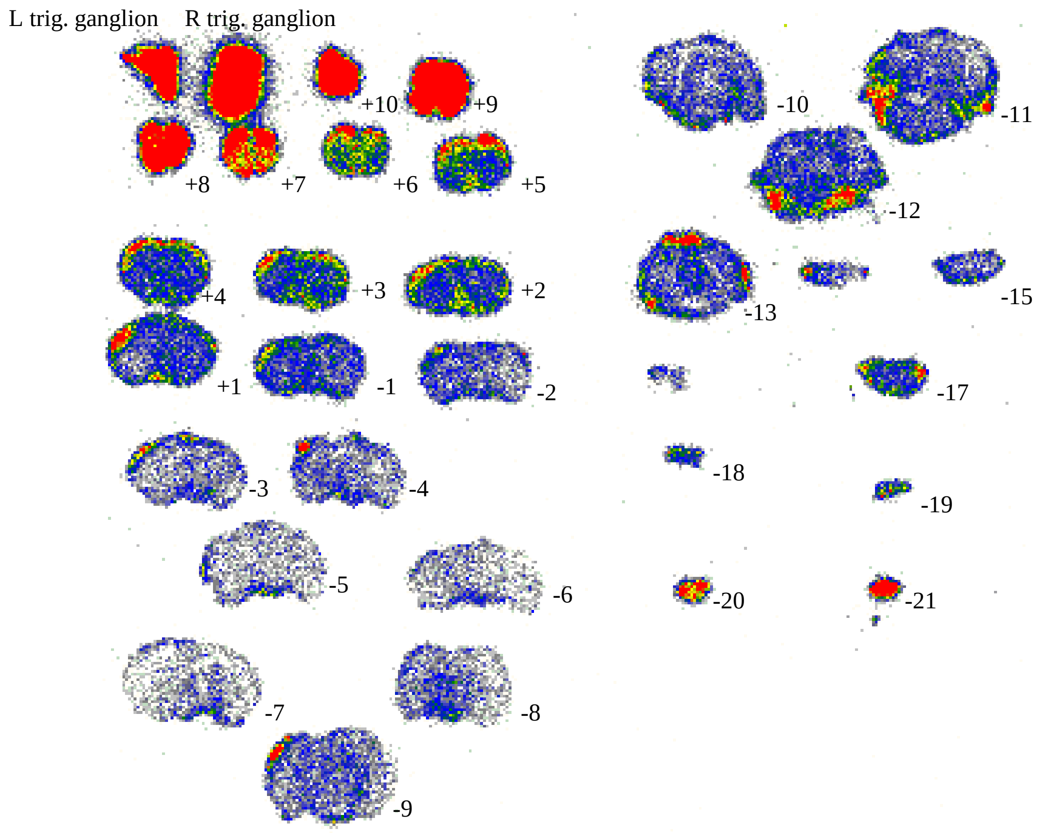

Image intensity range: 265-766

Figure D02: Rat 11 Intranasal Delivery of Oxytocin (labeled and unlabeled)

Delivered 51 ul over 14 min; 45 uCi, 0.4 mg, 397 nmol

Down on 10/22/07, developed on 11/13/07.

Slices measured from Bregma.

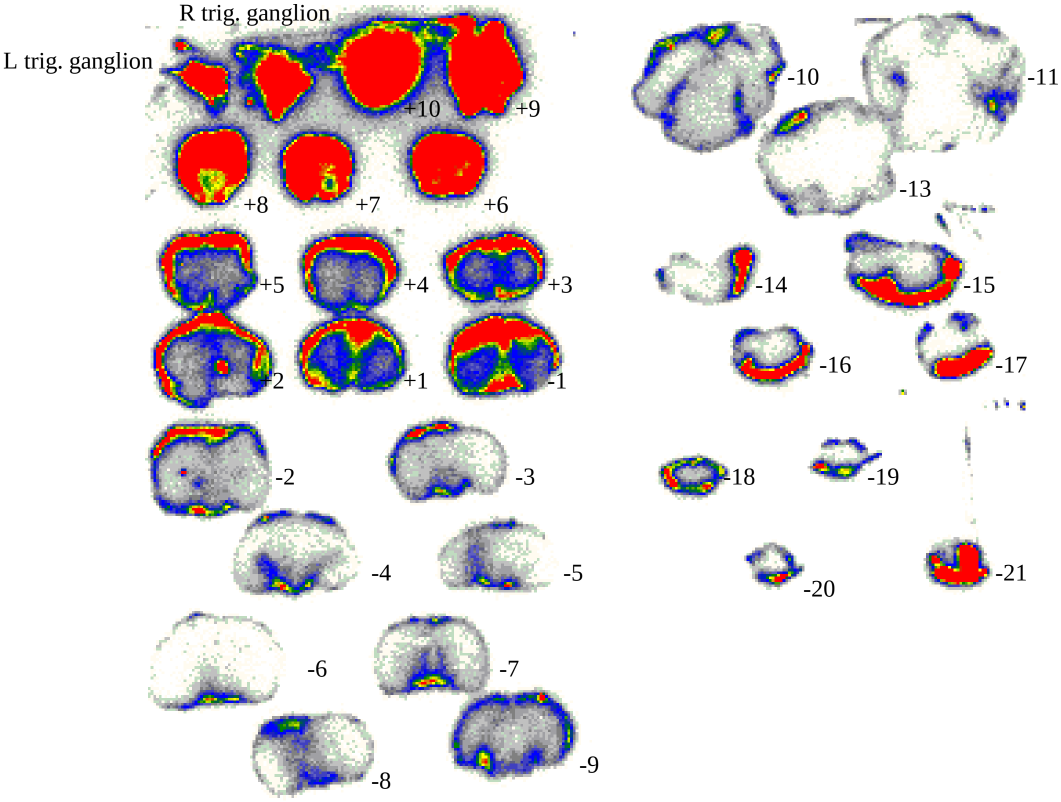

Image intensity range: 324-3223

Figure D03: Rat 12

Intranasal Delivery of Oxytocin (labeled and unlabeled)

Delivered 52 ul over 14 min; 45 uCi, 0.4 mg, 397 nmol

Down on 10/25/07, developed on 11/20/07.

Slices measured from Bregma.

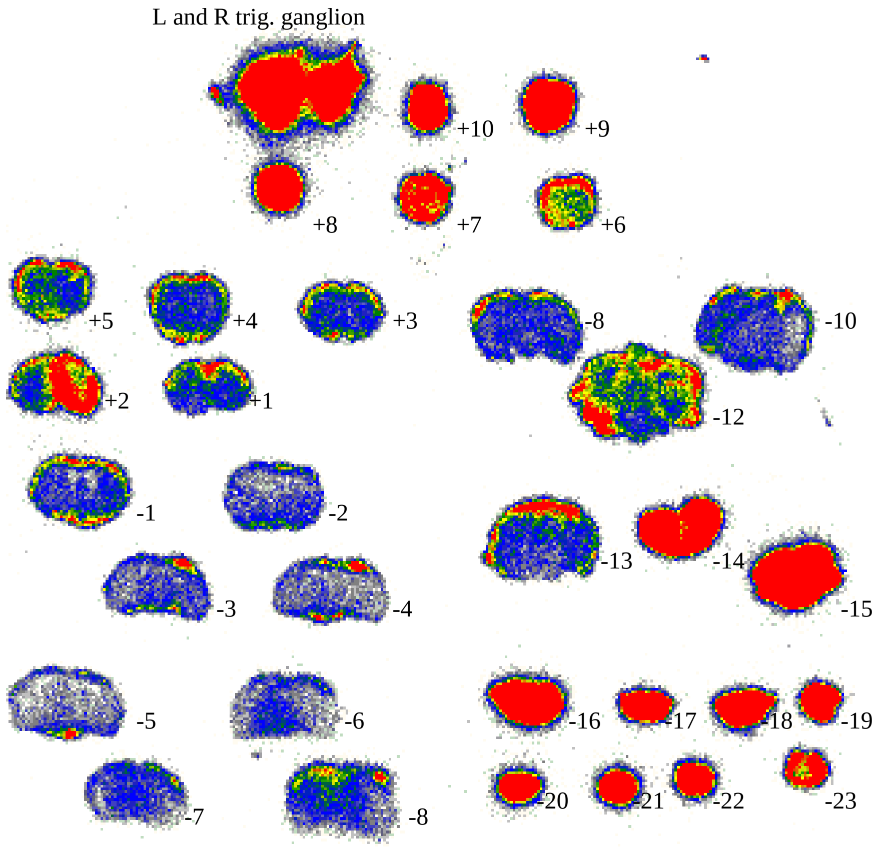

Image intensity range: 312-1141

Figure D04: Rat 13

Intranasal Delivery of Oxytocin (labeled and unlabeled)

Delivered 52 ul over 14 min; 45 uCi, 0.4 mg, 397 nmol

Down on 10/25/07, developed on 11/20/07.

Slices measured from Bregma.

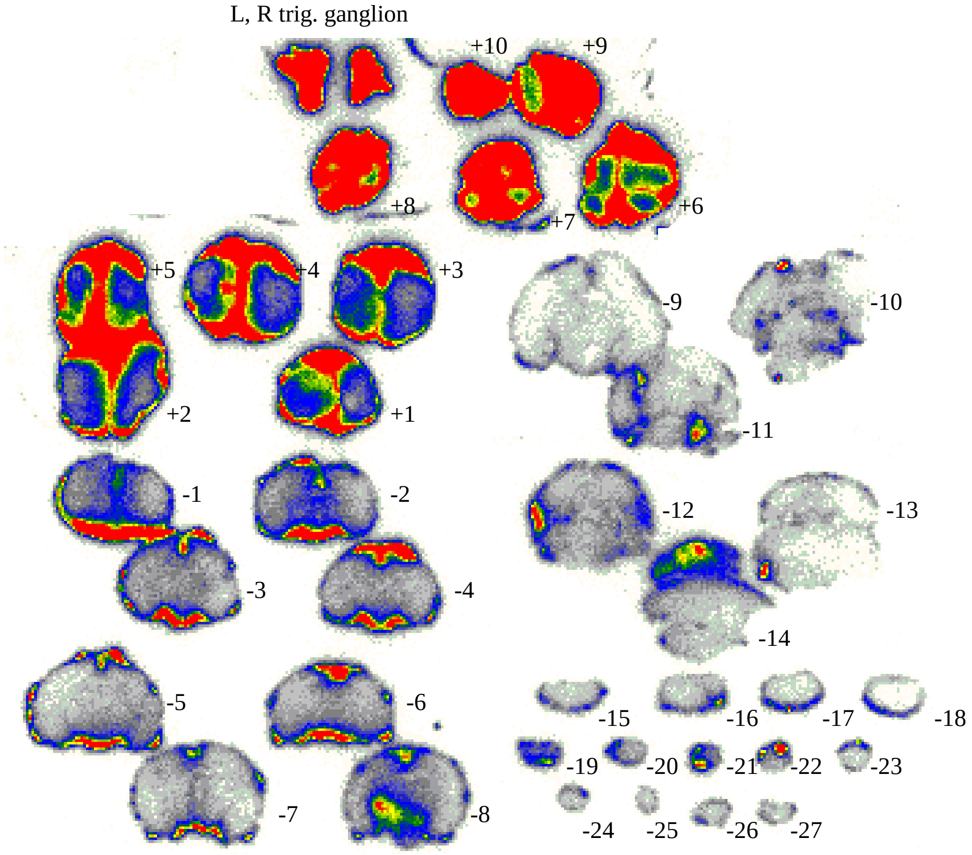

Image intensity range: 307-3627

Figure D05: Rat 14

Intranasal Delivery of Oxytocin (labeled and unlabeled)

Delivered 53 ul over 14 min; 45 uCi, 0.4 mg, 397 nmol

Down on 10/29/07, developed on 11/26/07.

Slices measured from Bregma.

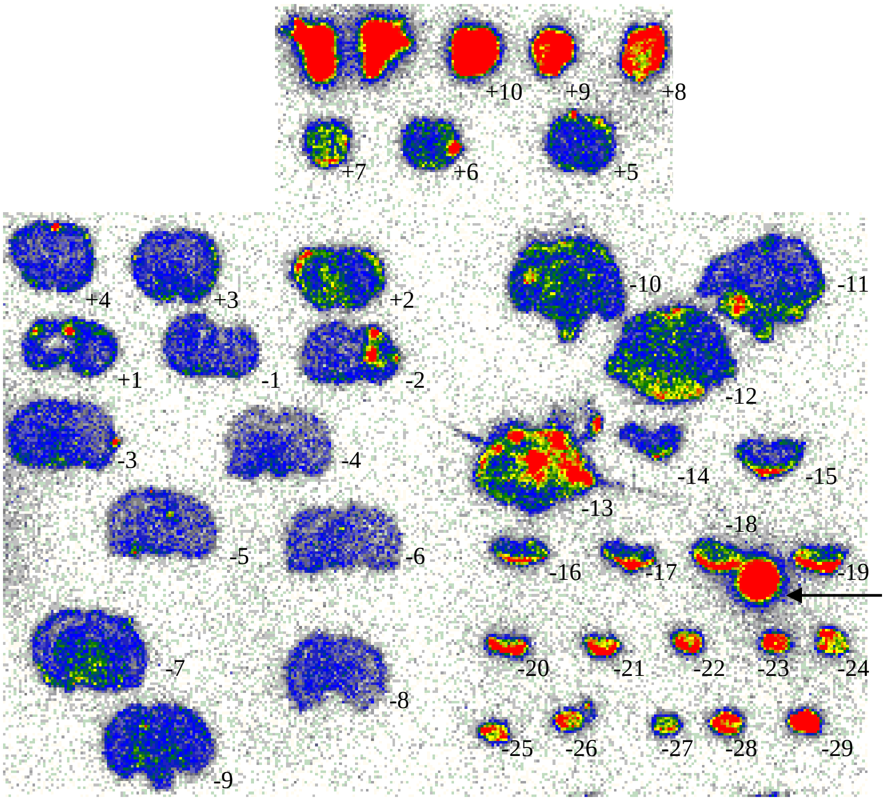

Image intensity range: 200-928

Figure D06: Rat 15

Intranasal Delivery of Oxytocin (labeled and unlabeled)

Delivered 53 ul over 14 min; 45 uCi, 0.4 mg, 397 nmol

Down on 10/29/07, developed on 11/26/07.

Slices measured from Bregma.

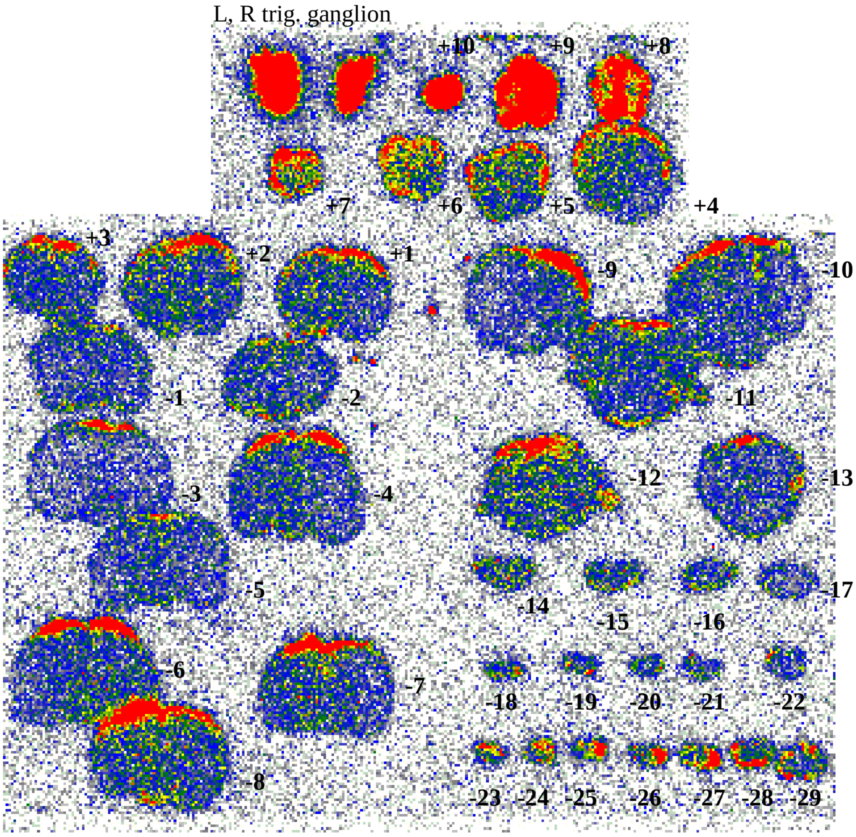

Image intensity range: 173-460

Figure D07: Rat 16

Intranasal Delivery of Oxytocin (labeled and unlabeled)
Delivered 48 ul over 14 min; 45 uCi, 0.4 mg, 397 nmol
Down on 10/30/07, developed on 12/11/07.
Slices measured from Bregma.

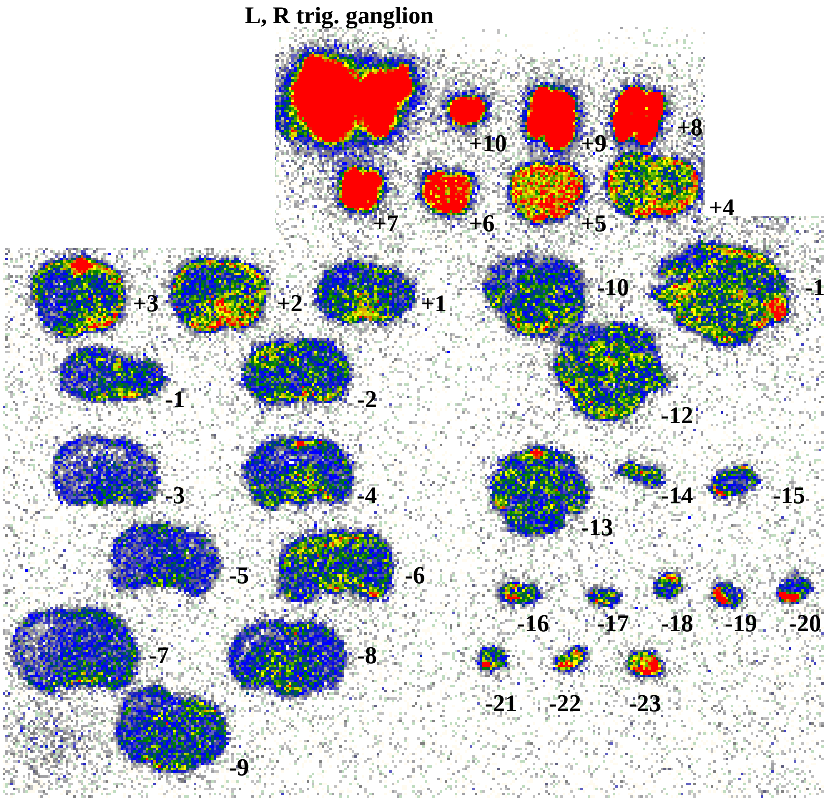

Image intensity range: 288-763

Figure D08: Rat 17

Intranasal Delivery of Oxytocin (labeled and unlabeled)
Delivered 48 ul over 14 min; 45 uCi, 0.4 mg, 397 nmol
Down on 10/30/07, developed on 12/11/07.
Slices measured from Bregma.

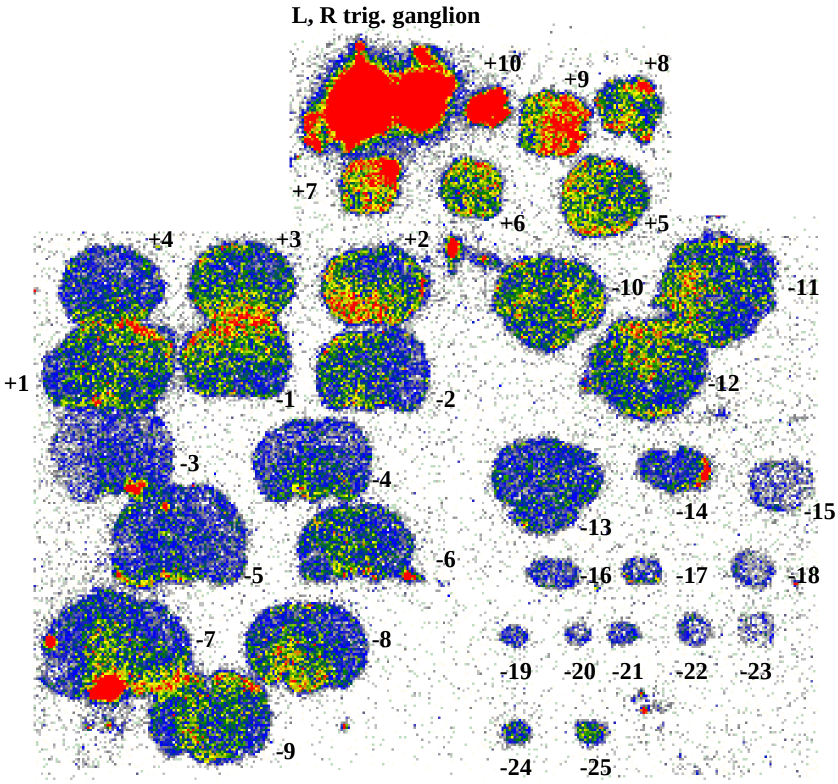

Image intensity range: 328-783

Figure D09: Rat 22

Intranasal Delivery of Oxytocin (labeled and unlabeled) with 1% Avicel Mucoadhesive

Delivered 48 ul over 14 min; 45 uCi, 0.4 mg, 397 nmol

Down on 10/30/07, developed on 12/11/07.

Slices measured from Bregma.

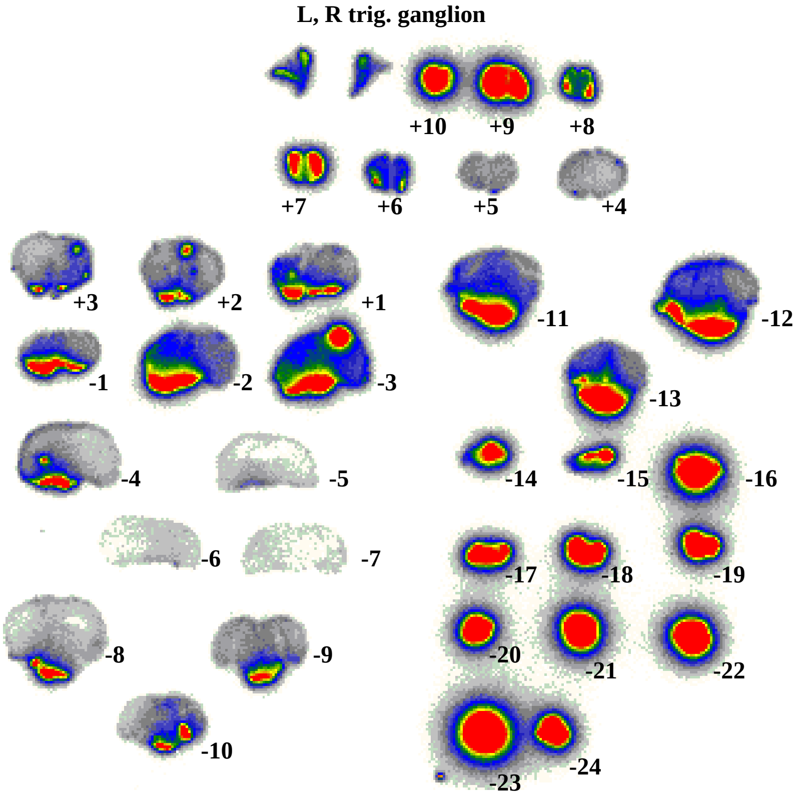

Image intensity range: 772-17,887

Figure D10: Rat 23

Intranasal Delivery of Oxytocin (labeled and unlabeled) with 1% Avicel Mucoadhesive

Delivered 48 ul over 14 min; 45 uCi, 0.4 mg, 397 nmol

Down on 10/30/07, developed on 12/11/07.

Slices measured from Bregma.

L, R trig. ganglion

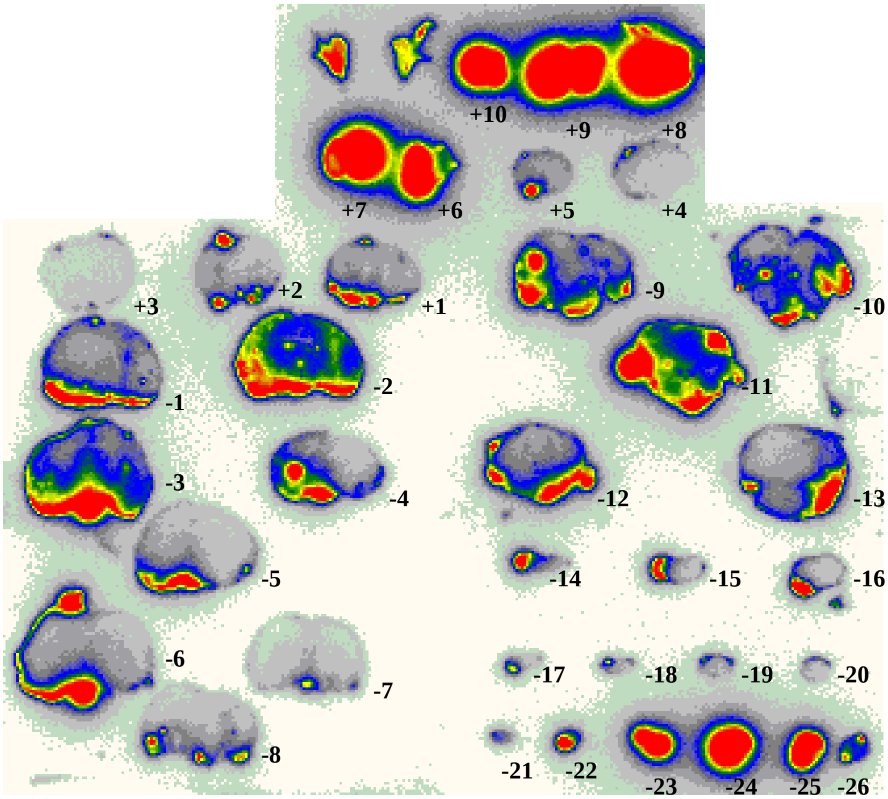

Image intensity range: 542-30,374

Figure D11: Rat 28

Intranasal Delivery of Oxytocin (labeled and unlabeled) with 1% Avicel Mucoadhesive

Delivered 51 ul over 14 min; 45 uCi, 0.4 mg, 397 nmol

Down on 11/13/07, developed on 12/17/07.

Slices measured from Bregma.

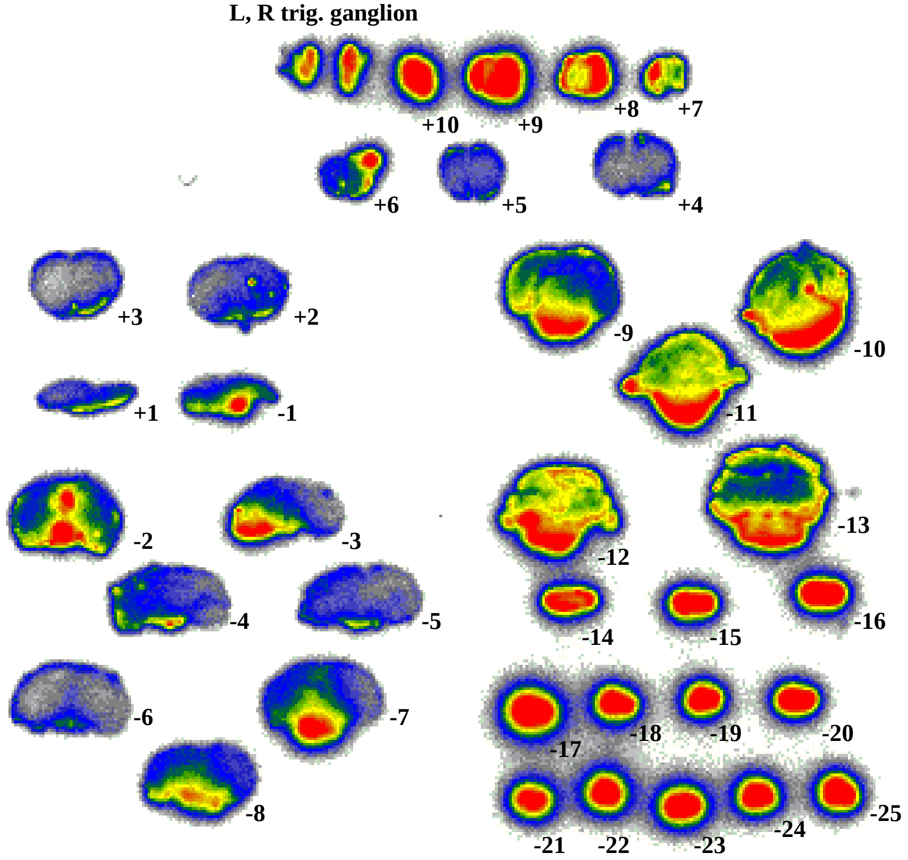

Image intensity range: 871-23,000

Figure D12: Rat 29

Intranasal Delivery of Oxytocin (labeled and unlabeled) with 1% Avicel Mucoadhesive

Delivered 51 ul over 14 min; 45 uCi, 0.4 mg, 397 nmol

Down on 11/13/07, developed on 12/17/07.

Slices measured from Bregma.

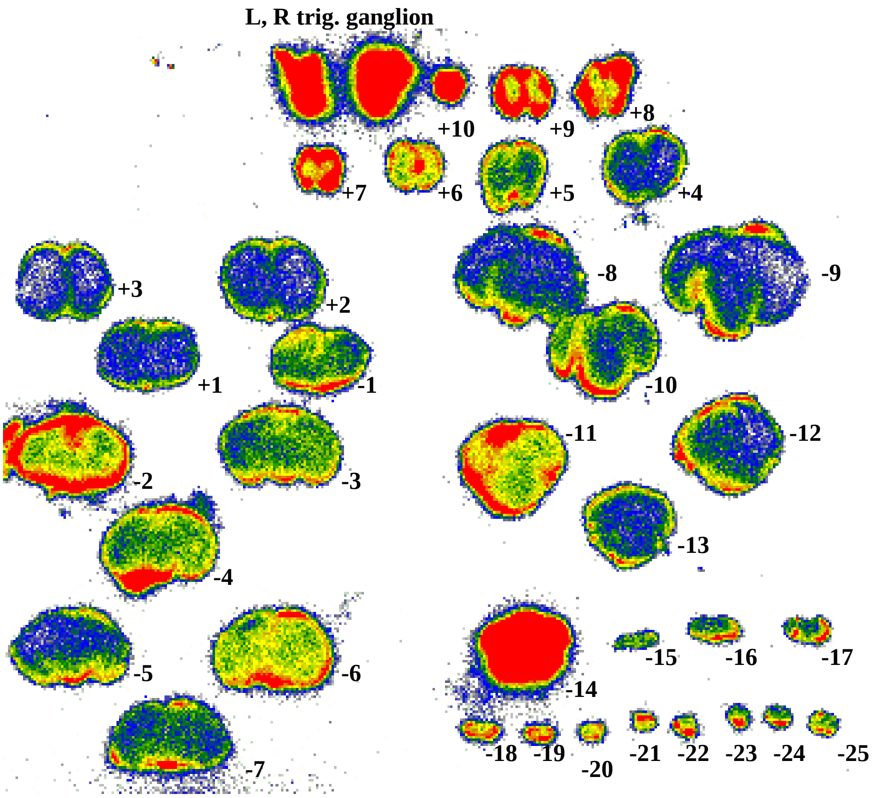

Image intensity range: 425-1,471

Figure D13: Rat 34

Intranasal Delivery of Oxytocin (labeled and unlabeled) with 1% Avicel Mucoadhesive

Delivered 51 ul over 14 min; 45 uCi, 0.4 mg, 397 nmol

Down on 11/20/07, developed on 12/20/07.

Slices measured from Bregma.

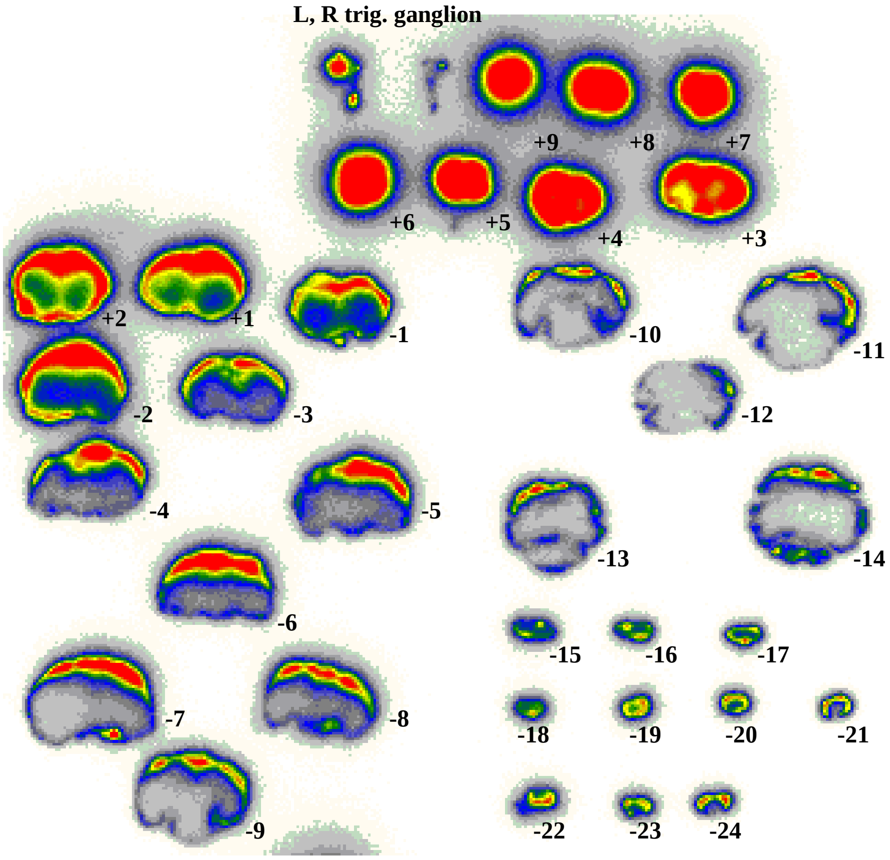

Image intensity range: 425-1,471

Figure D14: Rat 35

Intranasal Delivery of Oxytocin (labeled and unlabeled) with 1% Avicel Mucoadhesive
Delivered 51 ul over 14 min; 45 uCi, 0.4 mg, 397 nmol
Down on 11/20/07, developed on 12/20/07.
Slices measured from Bregma.

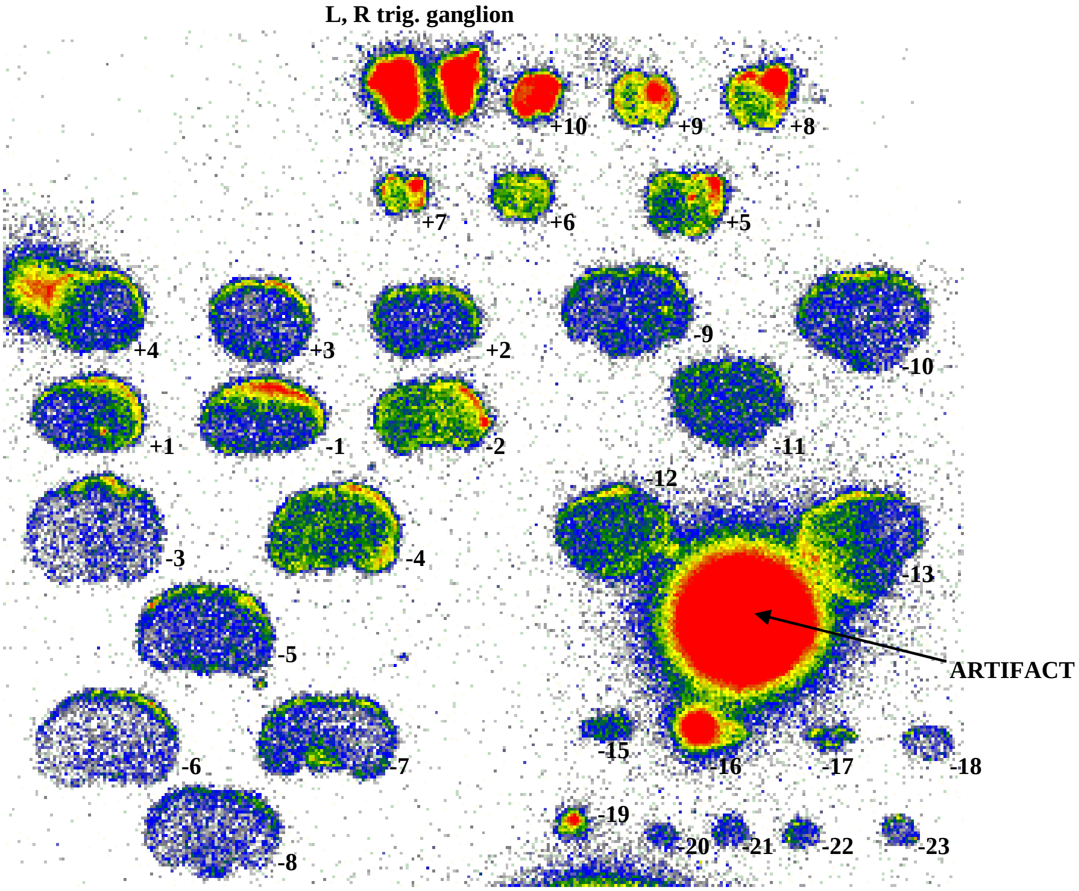

Image intensity range: 425-1,471

Figure D15: Rat 36

Intranasal Delivery of Oxytocin (labeled and unlabeled) with 1% Avicel Mucoadhesive

Delivered 49 ul over 14 min; 45 uCi, 0.4 mg, 397 nmol

Down on 11/26/07, developed on 01/04/08.

Slices measured from Bregma.

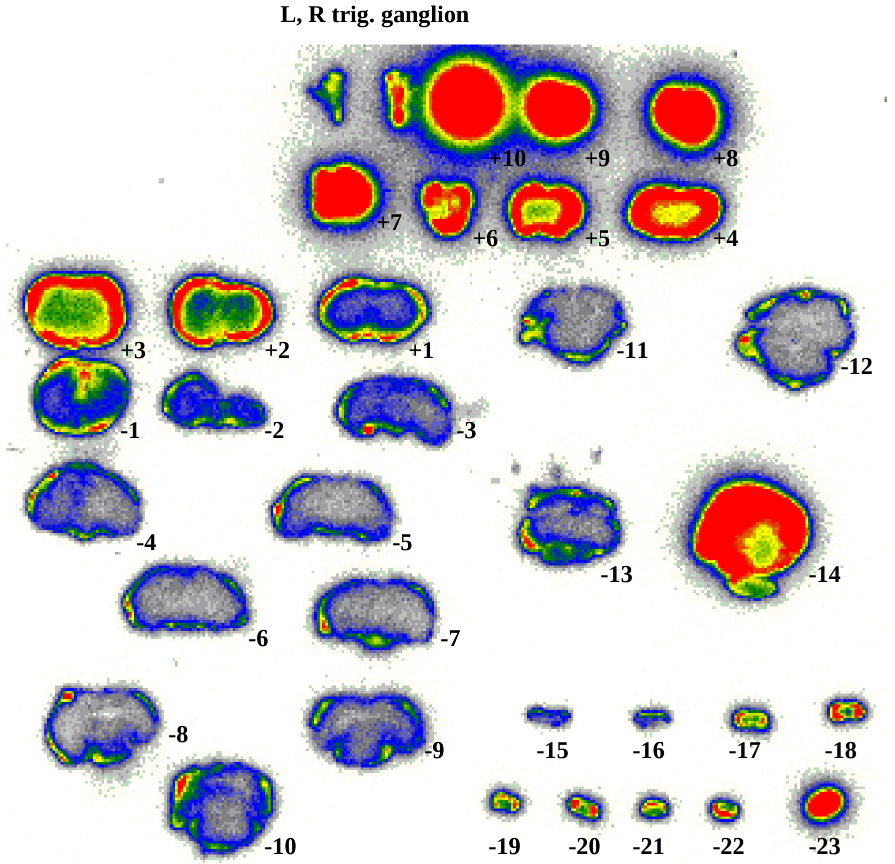

Image intensity range: 419-7,503

Figure D16: Rat 37

Intranasal Delivery of Oxytocin (labeled and unlabeled) with 1% Avicel Mucoadhesive

Delivered 49 ul over 14 min; 45 uCi, 0.4 mg, 397 nmol

Down on 11/26/07, developed on 01/04/08.

Slices measured from Bregma.

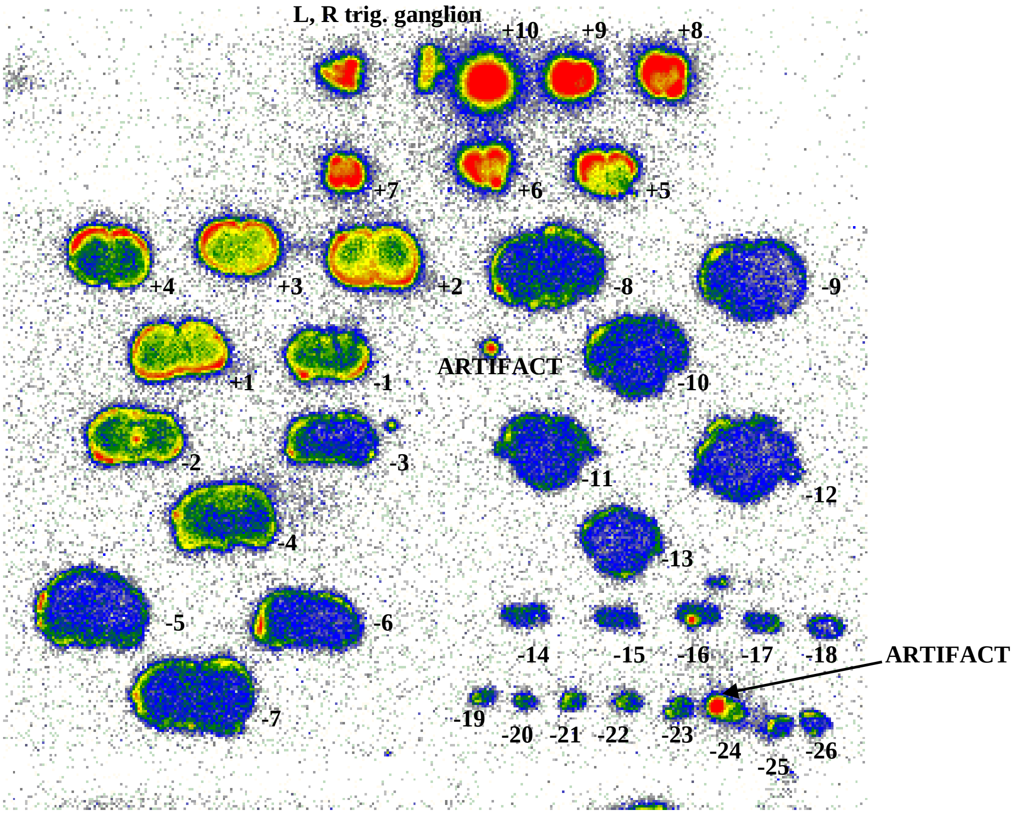

Image intensity range: 335-3,054

Appendix E – Coronal Rat Brain Slices

Figures

Figure E01: Brain Slice 01
Figure E02: Brain Slice 02
Figure E03: Brain Slice 03
Figure E04: Brain Slice 04
Figure E05: Brain Slice 05
Figure E06: Brain Slice 06
Figure E07: Lateral Brain Bisection

Figure E01: 1st Slice

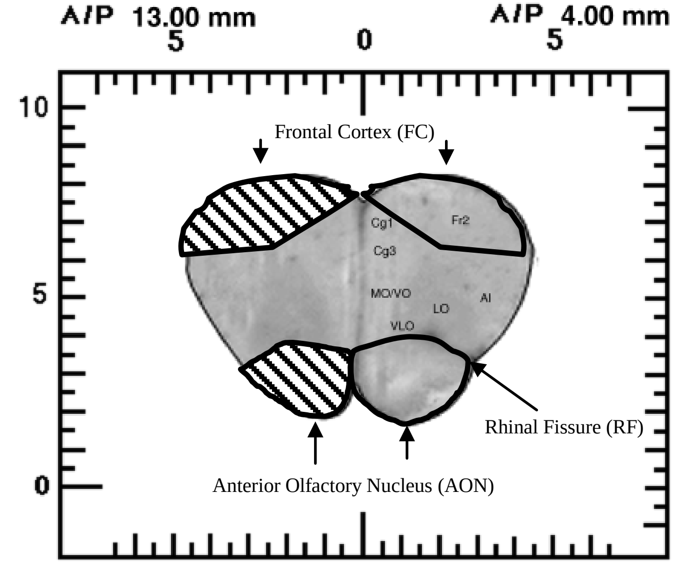

- **Frontal Cortex**
- **Ant. Olfactory Nucleus**

Figure E02: 2nd Slice

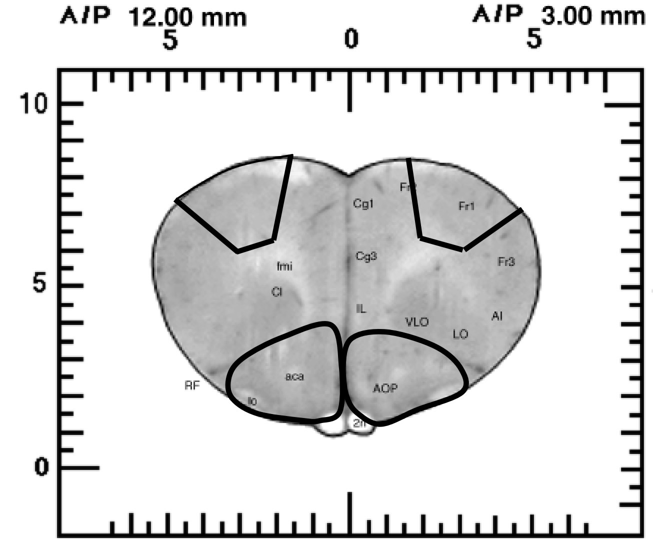

- **Frontal Cortex**
- **Ant. Olfactory Nucleus**

Figure E03: 3rd Slice

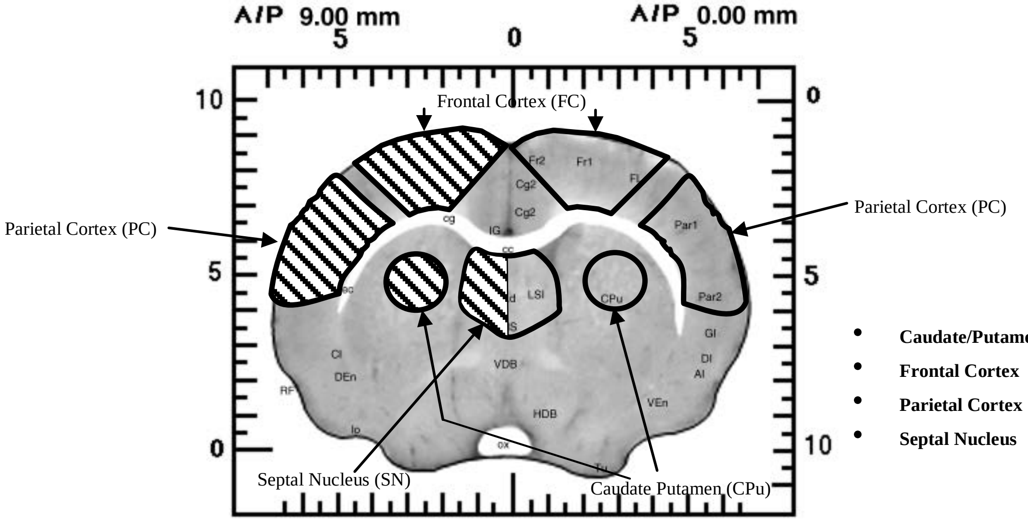

- **Caudate/Putamen**
- **Frontal Cortex**
- **Parietal Cortex**
- **Septal Nucleus**

Figure E04: 4th Slice

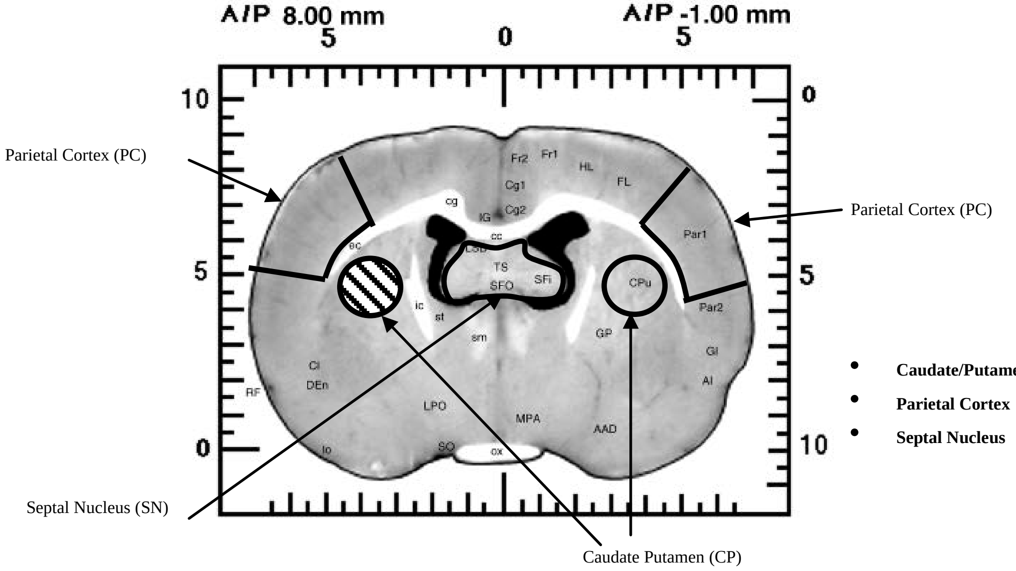

- **Caudate/Putamen**
- **Parietal Cortex**
- **Septal Nucleus**

Figure E05: 5th Slice

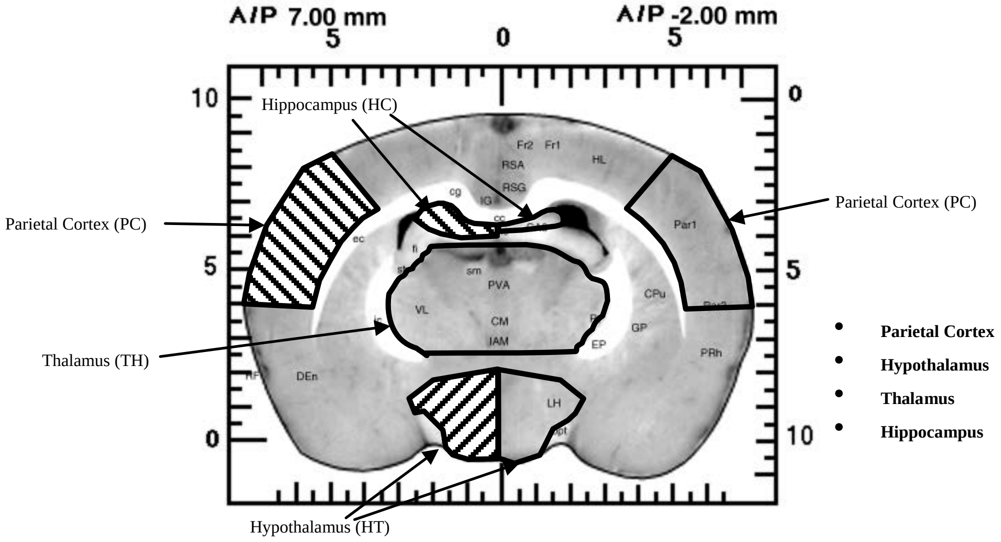

Figure E06: 6th Slice

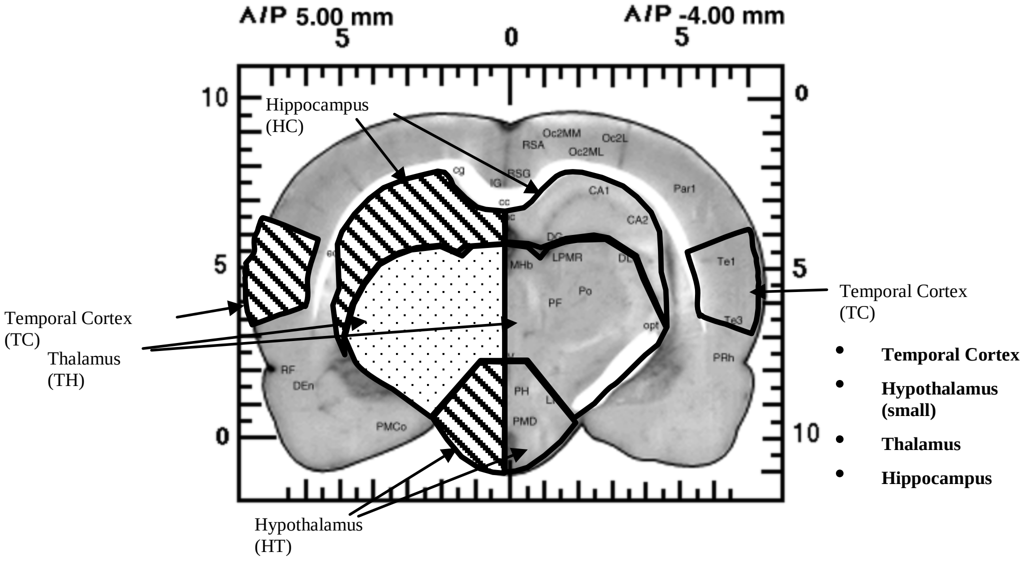

Figure E07: Rat Brain Bisection

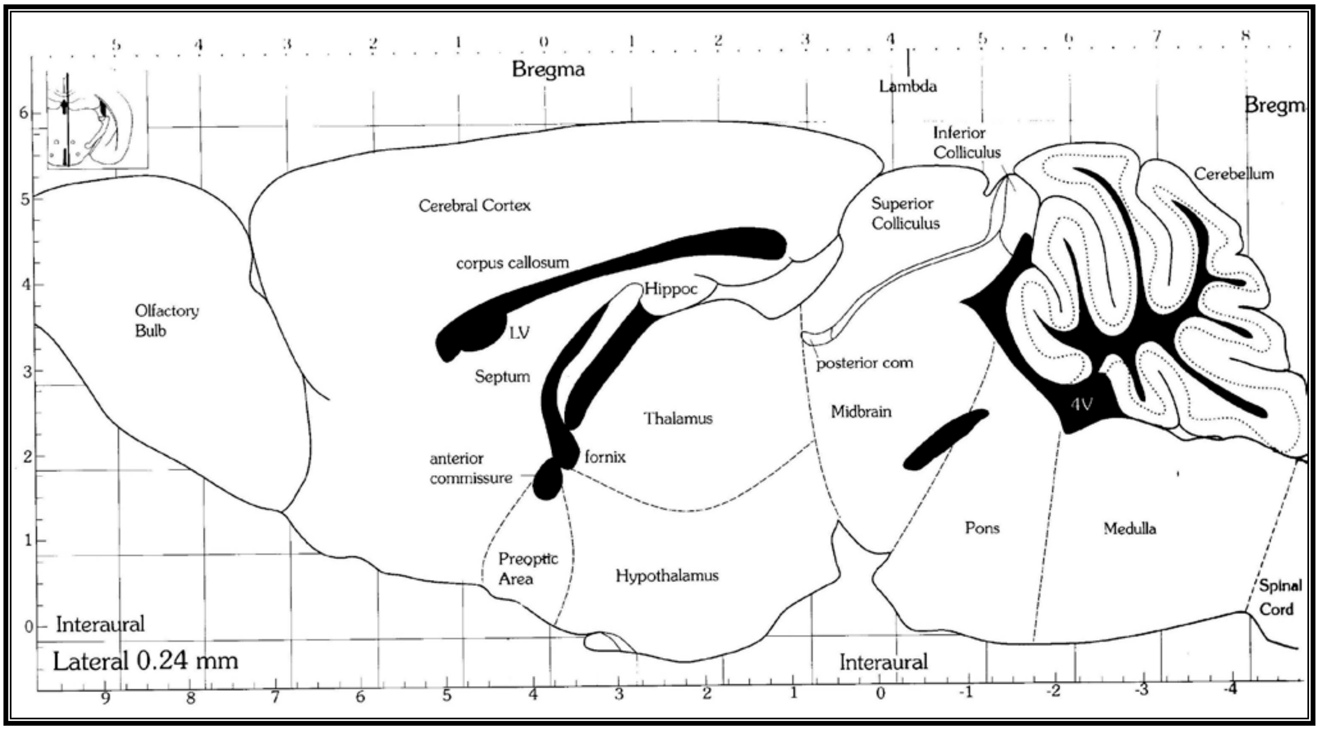

Pre-Clinical Pharmacokinetic Distribution Study in Rats for Intranasal Administration of Oxytocin

PART 2

Executive Summary

- Intranasal oxytocin rapidly reached the central nervous system with significant concentrations in the trigeminal nerve branches and trigeminal ganglion.

- Higher concentrations in olfactory and trigeminal structures confirmed delivery along these pathways.

- In animals receiving 0.04 mg oxytocin, the trigeminal ganglion was found to have an average concentration of 5.3 nM while the three major branches, maxillary, mandibular, and ophthalmic, were found to have concentrations of 7.5 nM, 8.9 nM, and 9.3 nM respectively. The average concentration of olfactory bulb samples was 3.6 nM.

- Concentrations achieved in the CNS after a single intranasal treatment with 0.04 mg (0.03 umol) oxytocin were high enough to be expected to activate high affinity oxytocin receptors in all brain and trigeminal tissues. Low affinity oxytocin receptors are not expected to be activated in any of the collected tissues as the nM tissue concentrations are not believed to be high enough.

- In animals receiving 0.004 mg oxytocin, the trigeminal ganglion was found to have an average concentration of 1.3 nM while the three major branches, maxillary, mandibular, and ophthalmic, were found to have oxytocin concentrations of 1.8 nM, 2.6 nM, and 2.1 nM respectively. The average concentration of olfactory bulb samples was 0.6 nM.

- Concentrations achieved in the CNS after a single intranasal treatment with 0.004 mg (0.003 umol) oxytocin were high enough to expect activation of high affinity oxytocin receptors in the trigeminal ganglion and all three trigeminal branches. However, concentrations were not high enough to expect activation of high affinity receptors in the brain or spinal cord.

- When comparing 0.4 mg, 0.04 mg, and 0.004 mg doses, concentrations achieved in collected tissues followed the expected dose-response trend when graphed logarithmically. Some tissues, including brain and trigeminal system samples from the 0.04 mg dose were not as high as expected while others were higher than expected. This may be in part due to the decreased viscosity of the dosing solution which may have decreased the efficiency of delivery. Similar trends were seen in the trigeminal system of 0.004 mg animals, but not as markedly in the brain concentrations of these animals.

♦ Table of Contents

Executive Summary 2

Table of Contents 3-4

Purpose of Study 5

Compounds and Dosing Calculations 5-6

General Procedures 7-13

Formulation of Dose Solution 7
Animals 7
Anesthesia 7-8
Intranasal Delivery of ^{125}I-Oxytocin 8-9
Transcardial Perfusion 9
Brain Dissection 9-11
Body Dissection 11-12
Tissue Counting 12
Data Analysis and Calculations 12-13

Results 13-20

Table 01: Intranasal Delivery of 0.04 mg Oxytocin Data Summary 16
Table 02: Intranasal Delivery of 0.004 mg Oxytocin Data Summary 17
Table 03: Comparison of 0.4 mg, 0.04 mg and 0.004 mg Intranasal Oxytocin 18
Table 04: Comparison of 0.4 mg, 0.04 mg and 0.004 mg Intranasal Oxytocin 19
Figure 01: Dose Response Curve 20

Appendices

Appendix A – 0.04 mg Intranasal Oxytocin Data 21-31

Table A01: Intranasal Oxytocin Rat 42 Data 22
Table A02: Intranasal Oxytocin Rat 43 Data 23
Table A03: Intranasal Oxytocin Rat 46 Data 24
Table A04: Intranasal Oxytocin Rat 47 Data 25
Table A05: Intranasal Oxytocin Rat 50 Data 26
Table A06: Intranasal Oxytocin Rat 51 Data 27
Table A07: Intranasal Oxytocin Rat 54 Data 28
Table A08: Intranasal Oxytocin Rat 55 Data 29
Table A09: Intranasal Oxytocin Rat 58 Data 30
Table A10: Intranasal Oxytocin Rat 59 Data 31

Appendix B – 0.004 mg Intranasal Oxytocin Data 32-42

Table B01: Intranasal Oxytocin with Mucoadhesive, Rat 44 Data 33
Table B02: Intranasal Oxytocin with Mucoadhesive, Rat 45 Data 34
Table B03: Intranasal Oxytocin with Mucoadhesive, Rat 48 Data 35
Table B04: Intranasal Oxytocin with Mucoadhesive, Rat 49 Data 36
Table B05: Intranasal Oxytocin with Mucoadhesive, Rat 52 Data 37
Table B06: Intranasal Oxytocin with Mucoadhesive, Rat 53 Data 38
Table B07: Intranasal Oxytocin with Mucoadhesive, Rat 56 Data 39
Table B08: Intranasal Oxytocin with Mucoadhesive, Rat 57 Data 40
Table B09: Intranasal Oxytocin with Mucoadhesive, Rat 60 Data 41
Table B10: Intranasal Oxytocin with Mucoadhesive, Rat 61 Data 42

Appendix C – Coronal Rat Brain Slices 43-50

Figure C01: Brain Slice 01 44
Figure C02: Brain Slice 02 45
Figure C03: Brain Slice 03 46
Figure C04: Brain Slice 04 47
Figure C05: Brain Slice 05 48
Figure C06: Brain Slice 06 49
Figure C07: Lateral Brain Bisection 50

Purpose of Study

The purpose of this study was to quantify the concentration and distribution of oxytocin in the central nervous system (CNS) and peripheral tissues after intranasal delivery to anesthetized rats. We examined the distribution of two different doses of oxytocin (0.04 mg and 0.004 mg) at 30 min after the onset of intranasal delivery. In both cases, a combination of ^{125}I-labeled oxytocin and unlabeled oxytocin was delivered. At 30 min after the onset of delivery, each animal was perfused with saline while under anesthesia followed by 4% paraformaldehyde and tissues were dissected. The concentration of oxytocin in the tissues was quantified using gamma counting.

Compounds and Dosing Calculations

Oxytocin was custom ^{125}I-radiolabeled by Perkin Elmer Life Sciences (3/10/08, Lot CIS31083) with an initial concentration of 1.0 mCi/ml and a specific activity of 2200 Ci/mmol. Radiolabeled oxytocin was prepared in a buffer containing 10 mM sodium phosphate (pH 7.4), 2.7 mM potassium phosphate, 137 mM sodium chloride, and 8.3% acetonitrile, 0.03% TFA. Using the molecular weight of oxytocin (1007.19 g/mol) the hot solution was calculated to contain 2184.3 uCi/ug at synthesis.

Cold oxytocin was purchased from Phoenix Pharmaceuticals (Acetate salt, ≥ 97%), product number 051-01, lot 425984. The product was stored at 0-5°C until ready for use.

The desired volume for intranasal delivery to anesthetized rats was 48 ul. Dosing calculations were used to determine the concentration necessary to achieve the desired volume to dose two rats (48 ul each), aliquot 3 standards (3 by 3 ul each), and provide 5 ul extra to for evaporation. The total prepared solution contained ~110 ul for each experimental day (enough for two rats). Using information provided by Perkin Elmer, the volume and concentration of hot and cold oxytocin in each dosing solution was calculated with a desired dose of 41 uCi and either 0.04 mg or 0.004 mg oxytocin.

Sample Calculations: ^{125}I-Oxytocin: Rat 42

To reach the desired 0.04 mg oxytocin while maintaining a 48 ul dose solution, the cold oxytocin solution was prepared at 20 mg/ml.

Volume hot to deliver=desired uCi/ (uCi/ul at synthesis)/ (decay rate of ^{125}I*(date of delivery - date of synthesis))

Delivery date 03/13/08:
Volume hot to deliver=41 uCi/1.0 uCi/ul/ (e^ (-0.01155*3 days) =42.45 ul

Volume cold to deliver=desired mg-(ul hot*(uCi/ul at synthesis)/ (uCi/ug at synthesis)/1000) / (cold ug/ul)*1000

Delivery date 3/13/08:
Volume cold to deliver= (0.04 mg-(42.45 uL*1.0 uCi/ul /2,184.3 uCi/ug /1000))/20 ug/ul *1000 =2.0 ul

Total volume to deliver= 42.45 + 2.0 = 44.44 ul

Volume hot to make dose solution=total ul desired/total ul to deliver*ul hot to deliver

Delivery date 3/13/08:
Volume hot to make dose solution=(44.44 + 9 + 5) ul/44.44 ul *42.45 ul=55.82 ul

Volume cold to make dose solution=total ul desired/total ul to deliver*ul cold in to deliver.

Delivery date 3/13/08:
Volume cold to make dose solution: (44.44 + 9 + 5) ul/44.44 ul*2.0 ul=2.63 ul

Total volume to make dose solution (dose, standards, evaporation) = 55.82 + 2.63 =58.44 ul

Sample Calculations: ^{125}I-Oxytocin, Rat 44

To reach the desired 0.004 mg oxytocin while maintaining a 48 ul dose solution, the cold oxytocin solution was prepared at 5 mg/ml.

Volume hot to deliver=desired uCi/ (uCi/ul at synthesis)/ (decay rate of ^{125}I*(date of delivery - date of synthesis))

Delivery date 03/17/08:
Volume hot to deliver=41 uCi/1.0 uCi/ul/ (e^ (-0.01155*7 days) =44.45 ul

Volume cold to deliver=desired mg-(ul hot*(uCi/ul at synthesis)/ (uCi/ug at synthesis)/1000) / (cold ug/ul)*1000

Delivery date 03/17/08:
Volume cold to deliver= (0.004 mg-(44.45 uL*1.0 uCi/ul /2,184.3 uCi/ug /1000))/5 ug/ul *1000 =0.8 ul

Total volume to deliver= 44.45 + 0.8 = 45.25 ul

Volume hot to make dose solution=total ul desired/total ul in dose solution*ul hot in dose solution

Delivery date 03/17/08:
Volume hot to make dose solution=(45.25 + 9 + 5) ul/45.25 ul *44.45 ul=58.21 ul

Volume cold to make dose solution=total ul desired/total ul in dose solution*ul cold in dose solution.

Delivery date 03/17/08:
Volume cold to make dose solution: (45.25 + 9 + 5) ul/45.25 ul *0.8 ul=1.04 ul

Total volume to make dose solution (dose, standards, evaporation) = 58.21 + 1.04 =59.25 ul

General Procedures

Throughout experimental procedures, strict precautions were followed to prevent radiation exposure and contamination of animal tissues, surgical tools, and equipment. Geiger counters were placed at each work station to continuously screen tools, workspace, and staff. Personal protective equipment including double layered gloves, lab coats, eye protection, masks, and bouffant caps was worn at all times. Lead impregnated shields were used to minimize exposure to radiation. Radioactive monitoring badges were also worn by staff throughout experimental procedures to quantify exposure.

Formulation of Dose Solution

To make the cold solution, a volume of 1X phosphate buffered saline (PBS) was prepared by diluting 10X PBS (Sigma Aldrich) into sterile water. Oxytocin was then weighed and added to the 1X PBS, making a solution of either 20.0 mg/ml or 5.0 mg/ml oxytocin in 1X PBS, pH 7. The solution was vortexed to ensure proper mixing.

According to dosing calculations, the corresponding volumes of hot and cold oxytocin were then added to pre-labeled 1.5 ml microcentrifuge tubes. The tubes were then capped, covered in parafilm for extra precaution, and placed in lead-lined storage containers, and stored at -20°C until ready for use.

On the day of delivery, the dosing solution was brought to room temperature on the bench top. All dosing solutions were vortexed prior to delivery.

Animals

Adult male Sprague Dawley rats (N=20, 275-300 g) were used throughout the study. Animals were group-housed in the Regions Hospital Animal Care Facility with free access to food and water. Animals were kept on a 12 h light cycle. All experimental procedures were approved by the Animal Care and Use Committee at HealthPartners Research Foundation.

There were two experimental groups of animals: group I received 0.04 mg oxytocin and was analyzed with gamma counting, group II received 0.004 mg oxytocin and was analyzed with gamma counting.

Anesthesia

Prior to anesthesia, each rat was weighed.

a. An anesthesia cocktail was made containing 3 parts ketamine, 3 parts xylazine, and 1 part acepromazine. A separate syringe containing pentobarbital was also drawn.
b. A 1-cc syringe fitted with a 22G, 1 in needle was assembled.
c. Full, half, and quarter doses of the ketamine cocktail were calculated according to the animal's weight at 0.5 ml/kg. A half dose of pentobarbital was also calculated at 50 mg/kg for use just prior to exsanguination.
d. A full dose of the ketamine cocktail was drawn into the syringe for injection.
e. The rat was placed ventral side down in the middle of a small hand towel.
f. The towel was wrapped around the animal's head and shoulders to restrain it.

g. With the animal restrained, a large flap of skin was gently pinched above the animal's right rear limb.
h. The needle was inserted subcutaneously into the skin fold at a 45° angle.
i. The plunger was pulled back to check for air or a blood flash.
j. If no blood was present, the anesthesia was pushed into the skin fold.
k. The rat was removed from the hand towel and placed in a holding cage.
l. The time and volume of anesthesia was noted on the animal's data sheet.
m. The animal's anesthesia level was assessed by pinching of the hind paw or tail. If the animal solicited a reflex, a ½ or ¼ dose booster was administered as necessary.
n. If the animal required an additional booster during drug delivery, the booster was given subcutaneously above the animal's left hind limb as follows:
    i. A skin flap above the hip joint was gently pinched and the needle inserted into the fold.
    ii. The plunger was pulled back to check for a blood flash.
    iii. If none was present, the booster was injected.
    iv. The time and dose were noted on the data sheet.

Intranasal Delivery of ^{125}I-Oxytocin

Anesthesia was monitored throughout the procedure. The animal's reflex was assessed by pinching the hind paw or tail while stabilizing the head to prevent movement. Quarter and half dose boosters of the ketamine cocktail were delivered subcutaneously as needed. Intranasal delivery was performed as follows:

a. The fully anesthetized rat was placed on its back on a heating pad in a metal surgical tray inside a fume hood. A properly lubricated rectal probe was inserted to monitor and maintain the rat's core temperature at 37°C.
b. Two 2"x2" gauze pads were rolled into a pillow and taped to secure. The pillow was then placed under the rat's head to maintain the position of the head so that the underside of the neck and mouth were horizontal.
c. A cotton swab was covered with parafilm.
d. A lead impregnated shield was placed in front of the surgical tray to protect the experimenter from radiation.
e. The dose solution, pipette, 30 ul with filter pipette tips, and waste receptacle were arranged behind the shield for easy access.
f. A series of three 3 ul standards were aspirated from the microcentrifuge tube containing the dosing solution and expelled into pre-weighed and labeled gamma counting tubes.
g. A 6 ul drop was loaded into the pipette behind the shield.
h. The cotton swab covered in parafilm was used to occlude one naris completely. The flat part of the swab was pushed gently against the naris at approximately a 45° angle to prevent airflow.
i. Holding the pipette at a 45° angle, the 6 ul drop was slowly expelled, forming a drop at the end of the tip.
j. The drop was lowered onto the open naris to be inhaled and the cotton swab was removed from the opposite naris.
k. The drop was inhaled.
l. The time and volume of the drop were recorded on the animal's data sheet.

m. After two minutes, the alternate naris was occluded and a 6 ul drop was administered in the same fashion to the open naris. The cotton swab was removed and the time was noted.
n. A drop was administered as described above every two minutes to alternating nares until a total of 8 drops were delivered (4 to each naris) for a total of ~48 ul.
o. Each drop was noted on the data sheet as well as any details regarding the animal or success of delivery.

Transcardial Perfusion

a. Eight minutes prior to exsanguination, a half dose of pentobarbital was administered as an intraperitoneal injection to the anesthetized rat to ensure surgical level of sedation.
b. Two min before the desired sacrifice time, the rectal probe and heating pad were removed from the metal surgical tray. While still on its back, labeling tape was used to secure the animal's front limbs to the pan. The back of the pan was elevated slightly to allow blood to flow away from the animal.
c. A toothed forceps was used to grasp the skin/fur covering the sternum. A cut was made through the animal's skin, exposing the sternum.
d. The sternum was clamped with a hemostat. The rib cage was cut open laterally, exposing the diaphragm.
e. The diaphragm was cut to expose the pleural cavity.
f. Surgical scissors were used to cut up the sides of the ribcage toward the armpits of the animal, creating a 'V' shaped incision exposing the heart.
g. The hemostat holding the sternum was taped above the animal's head to hold the cavity open.
h. The heart was stabilized using the blunt forceps while a 1 cc-syringe with 20 G, 1" sharp needle was inserted into the left ventricle. Approximately 0.2 cc of blood was removed and placed into a pre-weighed tube for gamma counting.
i. A small cut was made in the left ventricle using a spring scissors and an 18 G blunt needle attached to an extension set filled with 60 cc of saline was inserted through the left ventricle and into the aorta.
j. A large bulldog clamp was placed just above the heart on the aorta, securing the blunt needle in place.
k. The animal was perfused with 60 ml of saline followed by 360 ml of 4% paraformaldehyde at a rate of 15 ml/min.

Brain Dissection

Immediately after collection, each tissue sample was placed into a pre-labeled and pre-weighed gamma tube for quantification.

a. The skin and muscle around the neck were cut with a scalpel just above the shoulder blades. A large curved scissors was used to decapitate the animal, cutting dorsal to ventral to avoid contamination from the trachea and esophagus.
b. A midline incision was made on the dorsal side of the skull. The skin was peeled back up to the eyes.
c. A straight hemostat was used to peel the bone away from the top half of the brain. The top of the brain was exposed all the way to the olfactory bulbs.

d. Using a spring scissors and curved forceps, dorsal dura was collected off the surface of the brain.
e. The head was inverted and the brain was removed using a small spatula to free it from the cavity. The posterior optic nerve and trigeminal nerves were trimmed close to the brain using a spring scissors. The brain was then inverted and placed into a clean Petri dish.
f. The basilar artery and circle of Willis was carefully collected from the ventral side of the brain with sharp forceps. The brain was then covered with a kim-wipe moistened with 1X PBS, and placed aside for later dissection.
g. The ventral dura was collected from the skull cavity by scraping a forceps on the walls. The collected dura was placed into a pre-weighed gamma tube.
h. The ophthalmic branch of the trigeminal nerve was visualized on the inside of the skull. The scalpel was then used to carefully slice through the skull on the caudal side of the nerve, creating a vertical slice in the wall of the skull running parallel to the nerve.
i. A small hemostat was used to chip away the wall of the skull just up to the ophthalmic nerve.
j. A small spatula was run under the nerve and the skull, freeing the nerve from the skull wall. The nerve was then trimmed from the cavity and placed in the appropriate tube.
k. Once both sides of the ophthalmic nerve had been collected, the small hemostat was again used to chip away the bottom portion of the skull walls on both sides.
l. When the muscle layers were visible, the small spatula was used to tease out the mandibular nerve on both sides. Care was taken to retrieve a large portion by following the nerve into the lower jaw and chipping bone away with a hemostat if necessary.
m. Finally, the maxillary nerve and trigeminal ganglion were collected. The ganglion were removed with a curved forceps, trimming them from the maxillary segment just in front of the branching using a spring scissors.
n. The maxillary segment was collected by first removing any residual dura from the area using a forceps. Then, the curved forceps was used to gently pull the maxillary nerve while a spring scissors was inserted into the cavity as far as possible and used to snip the nerve from the remaining portion.
o. Once all of the nerve segments had been collected, the head was set aside and covered with a moist kim-wipe for later dissection.
p. The brain was retrieved and uncovered for dissection.
q. The brain was placed dorsal side down in a coronal brain matrix.
r. A razor blade was inserted at the optic chiasm. Additional blades were placed every 2 mm outward from the optic chiasm. Slices were generated from the midbrain to the tip of the forebrain, resulting in 6 slices. The blades were removed and inverted for dissection.
s. The olfactory bulbs were retrieved from the brain matrix and trimmed of any excess tissue. The bulbs were rostral to the first slice.
t. Tissues were dissected from corresponding slices (1-6) as diagrammed in Appendix E.

u. The remaining brain tissue in the matrix was retrieved and the upper cervical spinal cord was dissected and placed in the appropriate tube.
v. The brain segment was then bisected along the midline. Following figure 7 in Appendix E, the midbrain, pons, and medulla were collected by scoring each half straight down from the inferior colliculus. This marked the midbrain. Another score was made under the point in the fourth ventricle, marking the medulla. The cerebellum was peeled off and placed in the corresponding tube. If necessary, remaining cortex was also removed. Following the previously made scores, the midbrain, pons, and medulla were dissected and placed into pre-weighed tubes.
w. Next, the head was retrieved and the ventral side of the neck was cut anteriorly. The skin was peeled back exposing the neck muscles. The superficial nodes were dissected, cleared of connective tissues, and placed in pre-weighed gamma tubes.
x. The deep cervical nodes were located, dissected, cleared of connective tissue, and placed into pre-weighed tubes.
y. The thyroid was collected by gently peeling it off of the trachea with a forceps.
z. A single edge razor blade was then used to bisect the skull along the midline. The olfactory and respiratory epitheliums were collected from the cavity by scoring the edges and removing with a forceps. Care was taken to avoid the nasal septum.

Body Dissection

a. The rat was placed on its stomach and a superficial incision was made down the length of the animal from shoulders to hips, following the spine.
b. The skin was peeled away from the underlying tissue on both sides to expose the shoulder blades.
c. The axillary nodes in the connective tissue surrounding the shoulders were dissected, cleared of connective tissue, and placed in pre-weighed tubes.
d. A piece of right deltoid muscle was dissected, approximately 3 mm square, and placed into a pre-weighed gamma tube.
e. The muscles overlying the spine were scored with a scalpel. A small hemostat was inserted into the spinal column, above the cord, at the point of decapitation. The hemostat was clamped on the overlying vertebrae and tissues. The vertebrae and tissues were chipped away using an upward motion. The hemostat was inserted into the column repetitively, chipping away small pieces of the column each time, until the entire length of the cord was exposed.
f. When the spinal cord was fully exposed, a small spatula was used to loosen the cord from the spinal cavity. The spinal cord was than removed using a forceps and placed into a petri dish.
g. The intact spinal cord was cut into lower cervical, thoracic, and lumbar portions. The dura was peeled off of each section using a forceps and small spatula. All sections and the dura were then placed in respective pre-weighed tubes.
h. The rat was flipped onto its back and the sternum was cut vertically to further expose the abdominal and pleural cavities.
i. 3 mm square samples of the following tissues were dissected and placed into pre-weighed gamma counting tubes.
    1. liver, right superficial lobe
    2. left kidney, tip

3. right lung, top lobe
4. spleen, tip
5. heart, apex

j. Urine was removed from the bladder using a 1cc syringe and transferred to a pre-weighed tube.
k. A section of the renal artery feeding the animal's left kidney was collected.
l. A section of both the trachea and the esophagus were collected at the point of decapitation and placed in respective tubes.

Tissue Counting

The pre-weighed gamma tubes containing samples were reweighed to determine tissue weight. Tissue samples were then counted using a COBRA II Auto-Gamma Counter. The counter was normalized weekly to ensure a counting efficiency at or above 80%. The efficiency was then noted on each animal's data sheet. The samples were counted under a standard ^{125}I protocol with 5 min count time and an elevator position of 1. Immediately prior to sample counting, a background protocol was run to measure the current background noise. The average background at time of counting was then automatically subtracted by the gamma counter from the measured counts, a step previously calculated in the animal's EXCEL data sheet. In analysis of gamma counting results, any value outside two standard deviations of the mean for each tissue was considered an outlier and removed from the data set. Outliers are denoted on an animal's data sheet by an 'X'.

Data Analysis and Calculations

Sample calculations for rat 42 were completed by hand and imported to Microsoft EXCEL®. All values were then auto-calculated using identical spreadsheets and double checked for accuracy.

Sample Calculations

Specific activity from Perkin Elmer Specs = (uCi delivered*decay on day counted/decay on day delivered)/estimated nmol delivered * ($2.22*10^6$ DPM/uCi) * counting efficiency (CPM/DPM) * (10^{-6}) = specific activity (cpm/fmol)

Example: Rat 42 = (41.00 uCi * 0.9659 / 0.9659) / 39.71 nmol * (2.22 * 10^6 DPM/uCi) * 0.83 * (10^{-6}) = 1.90 CPM/fmol

Specific Activity from Standards = 1/ (average volume of standards (ul) * concentration of dose solution (ug/ul)/oxytocin molecular weight (ug/umol)/ average of standards (cpm-background)*10^9 fmol/umol) = specific activity (CPM/fmol)

Example: Rat 42 =1/ (3 ul *0.9 ug/ul /1007ug/umol /3,520,338 cpm * 10^9 fmol/umol)
= 1.31 CPM/fmol

nM tissue concentration (from Amersham Specs) = (CPM)/ (specific activity from specs in cpm/fmol)/ (tissue weight) *10^3 ml/L*10^{-6} fmol/nmol = nM tissue concentration

Example: Rat 42, Olfactory bulbs= (380 CPM)/ (1.90 CPM/fmol)/ (0.09985 g)/10^3
= 2.00 nM

nM tissue concentration (from standards) = (CPM)/tissue weight/ (specific activity from standards in CPM /fmol) $*10^3$ ml/L$*10^{-6}$ fmol/nmol = nM tissue concentration

Example: Rat 42, Olfactory bulbs= (380 CPM)/ (1.31 CPM/fmol)/ (0.09985 g)/10^3= 2.90 nM

Results

Intranasal Delivery of 0.04 mg ^{125}I-Oxytocin

Intranasal oxytocin successfully reaches the central nervous system with significant concentrations in the trigeminal nerve branches and trigeminal ganglion. The trigeminal ganglion was found to contain an average concentration of 5.3 nM while the three major branches, maxillary, mandibular, and ophthalmic, were found to contain average concentrations of 7.5 nM, 8.9 nM, and 9.3 nM respectively (Table 01). The average concentration of olfactory bulb samples was found to be 3.6 nM (Table 01). The high concentrations in these tissues confirm delivery to the brain along the olfactory and trigeminal nerve pathways. The highest delivery of oxytocin in the brain was observed in the frontal cortex (3.0 nM), septal nucleus (1.6 nM), anterior olfactory nucleus (1.5 nM), and hypothalamus (1.4 nM, Table 01). The lowest concentration of oxytocin in the brain was found in the thalamus at 1.0 nM (Table 01). The dorsal and ventral dura/meninges were found to contain concentrations of 4.0 and 2.9 nM oxytocin, respectively (Table 01). As the Kd for high affinity oxytocin receptors is ~1 nM and the Kd for low affinity oxytocin receptors is ~ 50 nM, this data suggests that at the dose administered, high affinity oxytocin receptors would be activated in all CNS tissues and low affinity receptors would not be activated.

The average concentration of oxytocin in the respiratory epithelium was found to be 58,080 nM (Table 01) leading to effective delivery along the trigeminal nerve pathway. The average concentration of oxytocin found in the upper cervical spinal cord was 1.8 nM (Table 01). The remaining spinal cord segments, lower cervical, thoracic, and lumbar, were found to contain concentrations of 0.7 nM, 0.6 nM, and 0.7 nM respectively (Table 01). With the exception of the upper cervical segment, these concentrations are not high enough to activate high affinity oxytocin receptors. Although our measurements were made 30 minutes after initiating intranasal treatment and 16 minutes after ending intranasal treatment, intranasal oxytocin likely reaches the trigeminal nerves within a minute or two following administration to the nasal cavity and reaches the brain within 10 minutes. Thirty minutes after initiation of intranasal delivery, the concentration of radiolabel found in the blood was 8.46 nM (Table 01). As the half-life of oxytocin in the blood is about 10 minutes, a significant portion of this radiolabel may represent degraded oxytocin.

Intranasal Delivery of 0.004 mg ^{125}I-Oxytocin

Oxytocin was again successfully delivered to the brain using intranasal delivery at the 0.004 mg dose but not in good yield. The average concentration found in olfactory bulb samples was 0.6 nM (Table 02). The trigeminal ganglion was found to contain an average concentration of 1.3 nM while the three major branches, maxillary, mandibular, and ophthalmic, were found to contain average concentrations of 1.8 nM, 2.6 nM, and 2.1 nM respectively (Table 02). All brain concentrations achieved at the 0.004 mg dose were found to be essentially equal, at 0.2 to 0.3 nM (Table 02). Concentration of oxytocin found in the respiratory epithelium averaged 2,728 nM

(Table 02). At this dose, high affinity oxytocin receptors would be activated in the trigeminal system only. The concentrations achieved in all brain structures are too low to activate even high affinity oxytocin receptors. The average concentration of oxytocin found in the upper cervical spinal cord was 0.4 nM (Table 02). The remaining spinal cord segments, lower cervical, thoracic, and lumbar, were found to contain average concentrations of 0.2 nM, 0.1 nM, and 0.1 nM respectively (Table 02). Again, these concentrations are too low to be expected to activate receptors. At 30 min after the onset of delivery, blood samples were found to average 2.42 nM oxytocin (Table 02).

Comparison of 0.4 mg (previous study), 0.04 mg, and 0.004 mg Intranasal Oxytocin
Table 3 was created to aid in side-by-side comparisons of the three doses explored to date: 0.4 mg, 0.04 mg, and 0.004 mg. As evident from Table 3, some tissues exhibit the expected ten fold decrease from dose to dose, while others did not. Of notable variation is the olfactory epithelium. At the 0.4 mg dose, oxytocin concentrations reached 4,112 nM (Table 3). However, the concentrations achieved at the 0.04 mg and 0.004 mg dose were both 92 nM (Table 3). Due to the decreased mg delivered, the dosing solution of the 0.04 mg dose was markedly less viscous than the 0.4 mg dose. This may attribute to the lower than expected nM concentrations of the 0.04 mg and 0.004 mg doses as a less viscous solution may not adhere to the walls of the nasal cavity as well, thereby decreasing the efficiency of delivery to the nasal mucosa. In addition, the company that previously ^{125}I-radiolabeled oxytocin is no longer in operation and a new company was used for this phase of the study. This may affect the legitimacy of comparisons to the 0.4 mg group.

To determine whether there is a dose dependant relationship of oxytocin when delivered intranasally, the ratios of the three different doses were compared to each other. In previous studies, rats were given 0.4 mg oxytocin intranasally using the same methods described above. Based on the amounts given which vary by three orders of magnitude (0.4 mg, 0.04 mg, and 0.004 mg) we expect to see tissue concentrations that vary by factors of ten as well. However, this was not always the case. These values were expressed as percentages for easy comparison and can be seen in Table 04.

When comparing the results of the 0.04 mg dose to previous results for the 0.4 mg dose, the blood sample for the 0.04 mg dose, found to be 8.46 nM (Table 03), is higher than expected at 13.8% of the 0.4 mg dose (Table 04). Despite a higher than expected blood concentration, almost all of the trigeminal and brain tissues were found to contain lower than expected oxytocin concentrations. The 0.04 mg trigeminal ganglion tissues were found to contain only 2% of the 0.4 mg tissues while all three branches contained only 3% (Table 04). With the exception of the olfactory bulbs, frontal cortex, and cerebellum tissues, all brain structures exhibited nM concentrations less than 10% of the nM concentrations achieved in the 0.4 mg dose (Table 04). The respiratory and olfactory epitheliums were also found to contain less than 10% of the 0.4 mg dose (Table 04). Again, this may be a result of the decreased viscosity of the dosing solution, varied preparation of the radiolabel by a different vendor, or a combination of the two.

When comparing the results of the 0.004 mg dose to previous results for the 0.4 mg dose, the blood sample for the 0.004 mg dose, found to be 2.42 nM (Table 03), is higher than expected at 3.94% of the 0.4 mg dose (Table 04). The concentration of oxytocin in the trigeminal ganglion

and three branches was again found to be lower than the anticipated 1% of the 0.4 mg dose (Table 04). The respiratory epithelium is lower than expected at 0.4% while the olfactory epithelium is higher than expected at 2.25% (Table 04). These results, again, may be attributed to the decreased viscosity of the dosing solution when compared to the 0.4 mg dose or the difference in vendor for ^{125}I-radiolabeling. With the exception of three brain tissues found to be slightly higher in concentration than expected (olfactory bulbs, midbrain, and cerebellum), many of the 0.004 mg brain tissues were found to contain nM concentrations at or near expected values when compared to the 0.4 mg dose (Table 04). Systemic tissues, however, were found to be higher than anticipated expected (Table 04).

Lastly, the nM tissue concentrations of animals dosed with 0.04 mg and 0.004 mg intranasal oxytocin were compared. The oxytocin concentration in the blood of animals receiving 0.004 mg was found to be nearly 3 times higher than anticipated (Table 04). Additionally, every brain, trigeminal, and systemic tissue was also found to be higher in concentration than expected when comparing the 0.004 mg animals to the 0.04 mg animals (Table 04). This may indicate less efficient delivery in the 0.04 mg animals or may suggest a non-linear response of mg dosed to nM tissue concentrations achieved.

For a visual comparison, 10 tissues of interest were selected and the data was compiled into a chart to evaluate the dose-response relationship of intranasal oxytocin. As seen in Figure 01, the concentration of oxytocin found in the ten tissues of interest increases as the mg dose of intranasal oxytocin increases. As the doses varied markedly and only included three values (0.4 mg, 0.04 mg, and 0.004 mg), a logarithmic scale was chosen.

Table 01: Intranasal Delivery of 0.04 mg Oxytocin Data Summary

	Mean	SE	Rat 42	Rat 43	Rat 46	Rat 47	Rat 50	Rat 51	Rat 54	Rat 55	Rat 58	Rat 59
**Anesthesia**			Ketamine	Ketamine	Ketamine	Ketamine	Ketamine	Ketamine	Ketamine	Ketamine	Ketamine	Ketamine
**mg oxytocin delivered**			0.040	0.040	0.040	0.040	0.040	0.040	0.040	0.040	0.040	0.040
**nmol oxytocin delivered**			39.71	39.71	39.72	39.72	39.71	39.71	39.72	39.71	39.72	39.72
**uCi delivered**			41.00	41.00	41.00	41.00	41.00	41.00	41.00	41.00	41.00	41.00
**uL delivered**			44.44	44.44	46.97	46.97	41.00	50.19	54.25	54.25	55.47	55.47
**Delivery time**			14:01	14:04	14:00	14:00	14:02	14:00	14:00	14:03	14:00	14:00
**Sac time**			31:01	30:57	30:36	30:46	31:47	30:07	30:58	31:10	30:45	30:40
**Notes**			Steady Breathing	Steady Breathing	Steady Breathing	Steady Breathing	Steady Breathing	Steady Breathing	Steady Breathing	Steady Breathing	Steady Breathing	Steady Breathing
**Tissue Description**	**nM**		nM	nM	nM	nM	nM	nM	nM	nM	nM	nM
**Blood Sample (30:00)**	**8.46**	**±0.4**	6.69	6.96	9.78	9.17	6.96	10.69	9.34	7.72	8.73	8.58
**Respiratory Epithelium**	**58,080**	**±10,300**	92,619.55	84,524.54	120,227.43	28,334.01	37,096.52	56,141.25	16,183.08	49,278.01	62,972.48	33,422.30
**Olfactory Epithelium**	**92**	**±27**	23.03	X	74.57	X	210.17	47.35	207.11	46.37	110.47	17.93
**Trigeminal Ganglion**	**5.3**	**±0.9**	7.49	9.19	X	2.65	7.12	8.18	2.37	3.52	3.24	4.12
**Maxillary Nerve**	**7.5**	**±0.9**	8.75	6.20	11.00	X	8.85	11.91	4.47	4.51	6.50	5.64
**Mandibular Nerve**	**8.9**	**±1.2**	6.62	14.62	X	X	12.55	9.40	8.93	5.78	4.90	8.18
**Opthalmic Nerve**	**9.3**	**±1.2**	5.86	10.58	10.82	X	7.61	9.95	9.54	4.79	6.98	17.25
**Olfactory Bulbs**	**3.6**	**±0.4**	2.90	4.33	3.30	2.90	5.74	2.48	1.36	2.90	4.96	5.43
**Anterior Olfactory Nucleus**	**1.5**	**±0.1**	1.24	X	1.57	X	1.81	1.60	0.93	1.45	1.89	1.23
**Frontal Cortex**	**3.0**	**±0.7**	1.36	5.45	1.44	6.38	2.17	2.12	1.12	1.85	6.17	1.58
**Caudate/Putamen**	**1.1**	**±0.1**	X	1.35	1.49	X	1.48	0.83	0.59	0.65	1.22	0.97
**Septal Nucleus**	**1.6**	**±0.3**	3.58	1.12	1.41	3.33	1.37	1.38	0.76	1.12	0.79	0.96
**Parietal Cortex**	**1.2**	**±0.1**	0.99	X	1.31	X	1.32	1.47	0.87	1.09	X	1.10
**Hippocampus**	**1.1**	**±0.1**	1.27	0.99	1.16	1.41	1.21	1.21	1.04	0.86	X	0.88
**Thalamus**	**1.0**	**±0.1**	0.82	1.12	1.04	X	1.25	1.15	0.72	1.00	1.20	0.72
**Hypothalamus**	**1.4**	**±0.2**	0.83	2.29	2.15	2.00	1.26	1.77	0.79	1.14	0.90	1.21
**Midbrain**	**1.1**	**±0.1**	0.97	1.52	1.36	X	1.03	1.67	0.80	0.96	1.00	0.89
**Pons**	**1.1**	**±0.1**	1.07	X	1.55	X	1.15	1.35	0.74	0.98	0.95	0.97
**Medulla**	**1.1**	**±0.1**	1.19	X	1.30	X	1.02	1.49	0.69	1.10	1.12	1.14
**Cerebellum**	**1.1**	**±0.0**	1.03	X	1.29	X	1.07	X	0.90	1.08	1.10	1.00
**Dorsal Dura**	**25**	**±4.8**	13.34	X	X	X	25.46	34.34	7.10	16.25	39.87	36.46
**Ventral Dura**	**14**	**±1.3**	12.52	X	15.49	12.55	14.42	X	8.23	16.10	21.24	15.26
**Spinal Dura**	**2.8**	**±0.6**	1.22	1.50	5.86	3.93	2.69	3.43	5.88	1.68	0.00	1.88
**Upper Cervical Spinal Cord**	**1.8**	**±0.1**	1.87	X	2.33	2.02	1.17	1.98	2.50	1.55	1.61	1.45
**Lower Cervical Spinal Cord**	**0.7**	**±0.1**	0.65	0.61	1.23	0.97	0.76	0.97	0.58	0.59	0.44	0.54
**Thoracic Spinal Cord**	**0.6**	**±0.0**	0.58	0.60	0.79	0.76	0.59	0.77	0.59	0.48	0.38	0.38
**Lumbar Spinal Cord**	**0.7**	**±0.0**	0.60	0.58	0.84	0.76	0.58	0.88	0.55	0.50	0.83	0.54
**Circle of Willis/Basilar Artery**	**7.0**	**±1.7**	-	-	-	-	-	-	2.84	10.64	5.83	8.62
**Renal Artery**	**2.0**	**±0.2**	-	-	-	-	-	-	2.37	1.43	2.41	1.81
**Superficial Nodes (2)**	**32**	**±7**	72.96	18.25	40.24	70.25	31.16	24.30	18.60	10.64	22.29	14.88
**Cervical Nodes (2)**	**13**	**±2.2**	X	8.66	25.59	6.79	11.18	18.68	18.59	6.72	6.87	11.98
**Axillary Nodes (2)**	**1.6**	**±0.1**	1.36	1.29	2.03	2.03	1.32	1.89	X	1.45	1.35	1.81
**Thyroid**	**80**	**±10**	X	44.78	64.39	70.76	54.13	116.86	69.92	137.25	75.81	86.96
**Muscle (R, deltoid)**	**1.4**	**±0.1**	1.15	1.46	1.79	1.72	1.32	X	1.47	1.06	1.23	1.32
**Liver (R, superficial lobe)**	**1.1**	**±0.1**	0.82	0.80	1.39	X	0.84	1.63	0.75	1.26	1.11	1.28
**Kidney (L, tip)**	**2.2**	**±0.3**	X	1.28	3.26	X	3.33	2.45	1.88	1.83	1.23	1.93
**Lung (R, top lobe)**	**2.1**	**±0.2**	X	2.99	2.93	1.86	1.54	2.07	1.26	2.59	1.41	2.28
**Trachea (near cut-off)**	**3.1**	**±0.5**	1.45	1.97	3.41	4.06	2.08	4.47	1.97	2.28	5.84	X
**Esophagus (near cut-off)**	**2.3**	**±0.2**	1.93	1.85	2.71	X	2.49	X	2.50	1.63	2.74	2.62
**Spleen (tip)**	**1.9**	**±0.2**	2.61	0.86	1.64	1.82	1.27	X	2.10	3.34	1.96	1.87
**Heart**	**1.1**	**±0.1**	1.11	1.46	1.10	X	0.99	1.39	1.14	1.17	0.77	0.66
**Urine (fill to line of tube)**	**4.2**	**±0.6**	4.23	0.91	3.60	2.65	5.43	7.72	6.54	2.98	5.31	2.92
**CPM/fmol**	**1.4**	**±0.0**	1.31	1.28	1.38	1.38	1.41	1.41	1.36	1.35	1.39	1.39

LEGEND
**X :** Outlier Removed from Data
**- :** Tissue Not Collected

Table 02: Intranasal Delivery of 0.004 mg Oxytocin Data Summary

**Anesthesia**			Ketamine	Ketamine	Ketamine	Ketamine	Ketamine	Ketamine	Ketamine	Ketamine	Ketamine	Ketamine
**mg oxytocin delivered**			0.004	0.004	0.004	0.004	0.004	0.004	0.004	0.004	0.004	0.004
**nmol oxytocin delivered**			3.97	3.97	3.97	3.97	3.97	3.97	3.97	3.97	3.97	3.97
**uCi delivered**			41.00	41.00	41.00	41.00	41.00	41.00	41.00	41.00	41.00	41.00
**uL delivered**			45.25	45.25	46.82	46.82	50.69	50.69	53.66	53.66	54.89	54.89
**Delivery time**			14:02	14:00	13:58	14:06	14:02	14:05	14:01	13:59	14:00	14:00
**Sac time**			31:05	31:00	31:02	30:35	31:04	30:44	30:46	31:01	30:37	30:30
**Notes**			Steady Breathing	Steady Breathing	Steady Breathing	Steady Breathing	Steady Breathing	Steady Breathing	Steady Breathing	Steady Breathing	Steady Breathing	Steady Breathing
	**Mean**	**SE**	**Rat 44**	**Rat 45**	**Rat 48**	**Rat 49**	**Rat 52**	**Rat 53**	**Rat 56**	**Rat 57**	**Rat 60**	**Rat 61**
**Tissue Description**	**nM**		nM	nM	nM	nM	nM	nM	nM	nM	nM	nM
**Blood Sample (30:00)**	**2.4**	**±0.2**	1.89	1.76	1.45	1.47	2.48	2.36	2.65	3.31	3.57	3.29
**Respiratory Epithelium**	**2,728**	**±233**	2,687.13	X	3,467.79	2,158.62	3,499.31	1,750.26	3,229.23	X	2,137.42	2,897.85
**Olfactory Epithelium**	**92**	**±36**	80.81	232.64	15.31	11.77	276.84	182.52	11.95	10.76	X	8.21
**Trigeminal Ganglion**	**1.3**	**±0.2**	1.75	1.44	2.15	1.39	1.82	1.05	0.98	0.70	0.65	0.62
**Maxillary Nerve**	**1.8**	**±0.4**	1.07	3.23	0.70	4.19	3.07	1.15	1.47	1.42	1.12	0.99
**Mandibular Nerve**	**2.6**	**±0.3**	2.80	2.19	X	1.25	3.43	1.54	3.94	2.13	2.10	4.04
**Opthalmic Nerve**	**2.1**	**±0.3**	1.59	3.96	0.58	2.02	X	2.63	2.08	X	2.08	2.04
**Olfactory Bulbs**	**0.6**	**±0.0**	0.57	0.66	0.65	0.45	0.63	0.54	0.82	0.82	0.71	X
**Anterior Olfactory Nucleus**	**0.3**	**±0.0**	0.27	0.56	0.21	0.19	0.31	0.27	0.46	0.33	0.33	X
**Frontal Cortex**	**0.3**	**±0.0**	0.30	0.32	0.24	0.20	0.35	0.34	X	0.34	0.27	0.38
**Caudate/Putamen**	**0.3**	**±0.0**	0.35	0.18	0.11	0.15	0.27	0.22	0.39	0.26	0.26	0.38
**Septal Nucleus**	**0.2**	**±0.0**	0.29	0.21	0.09	0.14	0.23	0.17	0.22	0.20	0.25	0.17
**Parietal Cortex**	**0.3**	**±0.0**	0.29	0.24	0.21	0.19	0.30	0.23	0.37	X	0.25	0.31
**Hippocampus**	**0.2**	**±0.0**	0.19	0.16	0.15	0.12	0.23	0.18	0.21	0.23	0.20	0.19
**Thalamus**	**0.2**	**±0.0**	0.23	0.15	0.13	0.14	0.22	0.16	0.18	0.21	0.22	0.20
**Hypothalamus**	**0.2**	**±0.0**	X	0.22	0.17	0.20	0.22	0.19	0.21	0.23	0.28	0.22
**Midbrain**	**0.2**	**±0.0**	0.38	0.19	0.18	0.15	0.28	0.21	0.25	0.20	0.25	0.22
**Pons**	**0.2**	**±0.0**	X	0.19	0.22	0.18	0.26	0.25	0.20	0.32	0.30	0.22
**Medulla**	**0.3**	**±0.0**	X	0.21	0.20	0.20	0.34	0.27	0.23	0.25	0.36	0.25
**Cerebellum**	**0.3**	**±0.0**	0.37	0.19	0.15	0.17	0.34	0.28	0.25	0.24	0.33	0.24
**Dorsal Dura**	**4.0**	**±0.7**	7.71	6.36	0.76	2.93	3.13	4.31	4.40	6.34	1.83	1.81
**Ventral Dura**	**2.9**	**±0.6**	1.63	1.69	1.14	1.51	4.78	5.05	6.44	2.19	2.22	2.44
**Spinal Dura**	**1.0**	**±0.3**	1.23	0.56	0.59	0.44	0.49	2.56	0.56	0.70	2.58	0.76
**Upper Cervical Spinal Cord**	**0.4**	**±0.1**	0.66	0.19	0.24	0.27	0.26	0.67	0.37	0.55	0.30	0.27
**Lower Cervical Spinal Cord**	**0.2**	**±0.0**	0.18	0.12	0.09	0.11	0.16	0.23	0.14	0.17	0.23	0.16
**Thoracic Spinal Cord**	**0.1**	**±0.0**	0.14	0.12	0.08	0.11	0.13	0.13	0.10	0.13	0.16	0.12
**Lumbar Spinal Cord**	**0.1**	**±0.0**	0.16	0.11	0.12	0.09	0.14	0.14	0.12	0.15	0.17	0.16
**Circle of Willis/Basilar Artery**	**1.2**	**±0.2**	-	-	-	-	1.09	1.30	2.29	0.65	1.26	0.86
**Renal Artery**	**0.6**	**±0.1**	-	-	-	-	0.36	0.72	0.62	0.79	X	0.51
**Superficial Nodes (2)**	**3.8**	**±0.7**	X	6.68	4.09	6.35	4.53	3.21	1.26	4.56	2.22	1.31
**Cervical Nodes (2)**	**3.0**	**±0.5**	5.48	2.97	2.08	3.21	1.16	3.17	5.60	0.91	1.78	3.23
**Axillary Nodes (2)**	**0.6**	**±0.1**	0.32	0.37	0.38	0.35	0.43	0.60	0.79	0.58	0.98	0.76
**Thyroid**	**29**	**±5**	16.23	12.68	14.95	11.87	24.01	26.84	35.63	40.36	51.47	53.85
**Muscle (R, deltoid)**	**0.3**	**±0.0**	0.27	0.27	0.25	0.32	0.35	X	0.44	0.29	X	0.39
**Liver (R, superficial lobe)**	**0.6**	**±0.1**	0.49	0.32	0.30	0.20	0.22	1.49	0.44	0.34	1.39	0.57
**Kidney (L, tip)**	**0.6**	**±0.1**	0.58	0.57	0.24	0.60	0.32	0.55	0.64	0.77	0.68	0.57
**Lung (R, top lobe)**	**0.6**	**±0.1**	X	0.40	0.40	0.30	0.56	0.43	0.64	1.23	0.79	0.96
**Trachea (near cut-off)**	**0.7**	**±0.1**	0.52	0.69	0.37	0.41	1.05	0.83	0.71	0.84	1.00	X
**Esophagus (near cut-off)**	**0.9**	**±0.1**	0.98	0.91	X	0.40	0.91	0.79	0.73	0.63	1.31	1.67
**Spleen (tip)**	**0.6**	**±0.1**	0.64	0.47	0.23	0.34	0.48	0.76	0.80	0.89	X	0.64
**Heart**	**0.3**	**±0.0**	X	0.26	0.23	0.19	0.29	0.40	0.25	0.27	X	0.29
**Urine (fill to line of tube)**	**1.1**	**±0.2**	1.47	0.70	0.49	0.97	0.66	1.09	X	1.93	1.41	1.00
**CPM/fmol**	**12.7**	**±0.1**	13.09	13.09	12.53	12.53	12.82	12.82	12.75	12.64	12.57	12.52

LEGEND
**X :** Outlier Removed from Data
**- :** Tissue Not Collected

Table 03: Intranasal Delivery of 0.4 mg, 0.04 mg and 0.004 mg Oxytocin

	0.4 mg Oxytocin (Previous Data)		0.04 mg Oxytocin		0.004 mg Oxytocin	
	Mean	SE	Mean	SE	Mean	SE
Tissue Description	nM		nM		nM	
Blood Sample (30:00)	61.36	±4.1	8.46	±0.4	2.42	±0.2
Respiratory Epithelium	680,567	±85,021	58,080	±10,300	2,728	±233
Olfactory Epithelium	4,112	±694	92	±27	92	±36
Trigeminal Ganglion	311	±79	5.3	±1	1.3	±0.2
Maxillary Nerve	264	±46	7.5	±0.9	1.8	±0.4
Mandibular Nerve	291	±38	8.9	±1.2	2.6	±0.3
Opthalmic Nerve	320	±58	9.3	±1.2	2.1	±0.3
Olfactory Bulbs	32.8	±13	3.6	±0.4	0.6	±0.0
Anterior Olfactory Nucleus	24.4	±4.6	1.5	±0.1	0.3	±0.0
Frontal Cortex	20.8	±3.8	3.0	±0.7	0.3	±0.0
Caudate/Putamen	41.1	±11	1.1	±0.1	0.3	±0.0
Septal Nucleus	28.0	±6	1.6	±0.3	0.2	±0.0
Parietal Cortex	27.9	±7	1.2	±0.1	0.3	±0.0
Hippocampus	15.5	±3.0	1.1	±0.1	0.2	±0.0
Thalamus	14.7	±6	1.0	±0.1	0.2	±0.0
Hypothalamus	21.5	±3.9	1.4	±0.2	0.2	±0.0
Midbrain	13.6	±2.7	1.1	±0.1	0.2	±0.0
Pons	25.6	±10	1.1	±0.1	0.2	±0.0
Medulla	25.4	±9	1.1	±0.1	0.3	±0.0
Cerebellum	10.3	±1.4	1.1	±0.0	0.3	±0.0
Dorsal Dura	152	±12	25	±4.8	4.0	±0.7
Ventral Dura	271	±43	14	±1.3	2.9	±0.6
Spinal Dura	31.3	±8	2.8	±0.6	1.0	±0.3
Upper Cervical Spinal Cord	32.7	±8	1.8	±0.1	0.4	±0.1
Lower Cervical Spinal Cord	4.2	±0.3	0.7	±0.1	0.2	±0.0
Thoracic Spinal Cord	4.7	±0.5	0.6	±0.0	0.1	±0.0
Lumbar Spinal Cord	4.6	±0.6	0.7	±0.0	0.1	±0.0
Circle of Willis/Basilar Artery	-	-	7	±1.7	1.2	±0.2
Renal Artery	-	-	2.0	±0.2	0.6	±0.1
Superficial Nodes (2)	232	±25	32	±7	3.8	±0.7
Cervical Nodes (2)	213	±36	13	±2.2	3.0	±0.5
Axillary Nodes (2)	21.8	±2.1	1.6	±0.1	0.6	±0.1
Thyroid	213	±36	80	±10	29	±5
Muscle (R, deltoid)	13.6	±1.2	1.4	±0.1	0.3	±0.0
Liver (R, superficial lobe)	13.3	±1.3	1.1	±0.1	0.6	±0.1
Kidney (L, tip)	43.6	±3.0	2.2	±0.3	0.6	±0.1
Lung (R, top lobe)	20.7	±2.4	2.1	±0.2	0.6	±0.1
Trachea (near cut-off)	25.7	±3.2	3.1	±0.5	0.7	±0.1
Esophagus (near cut-off)	24.6	±2.0	2.3	±0.2	0.9	±0.1
Spleen (tip)	22.7	±2.1	1.9	±0.2	0.6	±0.1
Heart	16.3	±1.2	1.1	±0.1	0.3	±0.0
Urine (fill to line of tube)	72.8	±11.2	4.2	±0.6	1.1	±0.2
CPM/fmol	0.2	±0.0	1.4	±0.0	12.7	±0.1

Table 04: Comparison of 0.04, 0.04, and 0.004 mg Intranasal Oxytocin Doses
(Expressed as Percentages)

Tissue Description	0.04 mg versus 0.4 mg (%)		0.004 mg versus 0.4 mg (%)		0.004 mg versus 0.04 mg (%)	
	Measured	Expected	Measured	Expected	Measured	Expected
Blood Sample (30:00)	13.8	10.0	3.9	1.00	28.6	10.0
Respiratory Epithelium	8.5	10.0	0.4	1.00	4.7	10.0
Olfactory Epithelium	2.2	10.0	2.2	1.00	100.2	10.0
Trigeminal Ganglion	1.7	10.0	0.4	1.00	23.6	10.0
Maxillary Nerve	2.9	10.0	0.7	1.00	24.4	10.0
Mandibular Nerve	3.0	10.0	0.9	1.00	29.3	10.0
Opthalmic Nerve	2.9	10.0	0.7	1.00	22.9	10.0
Olfactory Bulbs	11.1	10.0	2.0	1.00	17.9	10.0
Anterior Olfactory Nucleus	6.0	10.0	1.3	1.00	22.3	10.0
Frontal Cortex	14.2	10.0	1.5	1.00	10.3	10.0
Caudate/Putamen	2.6	10.0	0.6	1.00	23.9	10.0
Septal Nucleus	5.7	10.0	0.7	1.00	12.5	10.0
Parietal Cortex	4.2	10.0	0.9	1.00	22.7	10.0
Hippocampus	7.2	10.0	1.2	1.00	16.7	10.0
Thalamus	6.8	10.0	1.3	1.00	18.3	10.0
Hypothalamus	6.7	10.0	1.0	1.00	15.1	10.0
Midbrain	8.3	10.0	1.7	1.00	20.4	10.0
Pons	4.3	10.0	0.9	1.00	21.7	10.0
Medulla	4.5	10.0	1.0	1.00	22.7	10.0
Cerebellum	10.4	10.0	2.5	1.00	24.2	10.0
Dorsal Dura	16.2	10.0	2.6	1.00	16.0	10.0
Ventral Dura	5.3	10.0	1.1	1.00	20.1	10.0
Spinal Dura	9.0	10.0	3.3	1.00	37.3	10.0
Upper Cervical Spinal Cord	5.6	10.0	1.2	1.00	20.6	10.0
Lower Cervical Spinal Cord	17.4	10.0	3.7	1.00	21.4	10.0
Thoracic Spinal Cord	12.5	10.0	2.6	1.00	20.4	10.0
Lumbar Spinal Cord	14.5	10.0	3.0	1.00	20.6	10.0
Circle of Willis/Basilar Artery	-	-	-	-	17.8	10.0
Renal Artery	-	-	-	-	29.9	10.0
Superficial Nodes (2)	13.9	10.0	1.6	1.00	11.7	10.0
Cervical Nodes (2)	6.0	10.0	1.4	1.00	23.2	10.0
Axillary Nodes (2)	7.4	10.0	2.6	1.00	34.5	10.0
Thyroid	37.5	10.0	13.5	1.00	35.9	10.0
Muscle (R, deltoid)	10.2	10.0	2.4	1.00	23.2	10.0
Liver (R, superficial lobe)	8.3	10.0	4.3	1.00	52.5	10.0
Kidney (L, tip)	4.9	10.0	1.3	1.00	25.6	10.0
Lung (R, top lobe)	10.1	10.0	3.1	1.00	30.2	10.0
Trachea (near cut-off)	11.9	10.0	2.8	1.00	23.3	10.0
Esophagus (near cut-off)	9.4	10.0	3.8	1.00	40.1	10.0
Spleen (tip)	8.5	10.0	2.6	1.00	30.1	10.0
Heart	6.7	10.0	1.7	1.00	25.0	10.0
Urine (fill to line of tube)	5.8	10.0	1.5	1.00	25.5	10.0

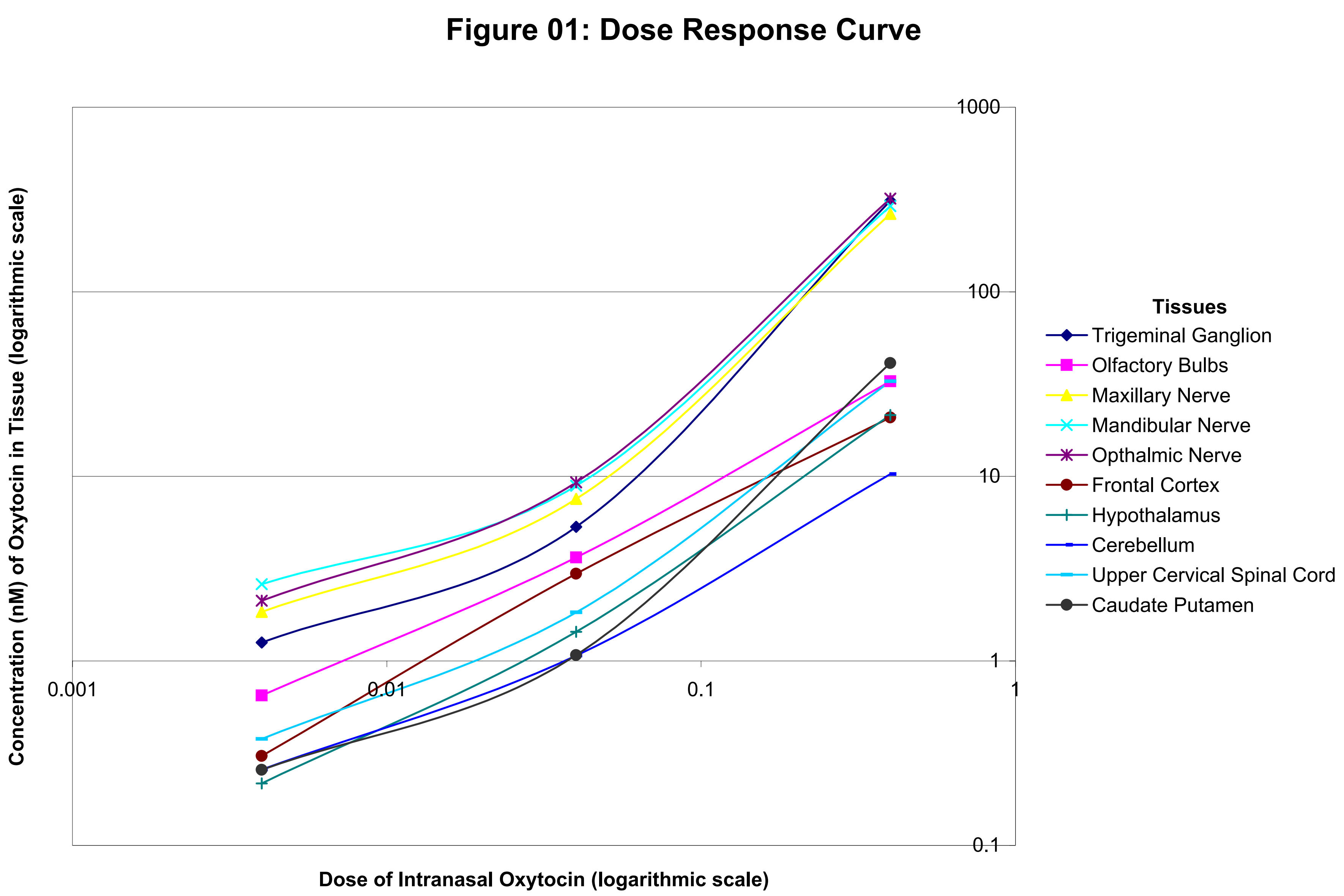
Figure 01: Dose Response Curve
Concentration (nM) of Oxytocin in Tissue (logarithmic scale)
1000
100
10
1
0.1
0.001
0.01
0.1
1
Dose of Intranasal Oxytocin (logarithmic scale)
Tissues
Trigeminal Ganglion
Olfactory Bulbs
Maxillary Nerve
Mandibular Nerve
Opthalmic Nerve
Frontal Cortex
Hypothalamus
Cerebellum
Upper Cervical Spinal Cord
Caudate Putamen

Appendix A – 0.04 mg Intranasal Oxytocin Data

Tables

Table A01: Intranasal Oxytocin Rat 42 Data
Table A02: Intranasal Oxytocin Rat 43 Data
Table A03: Intranasal Oxytocin Rat 46 Data
Table A04: Intranasal Oxytocin Rat 47 Data
Table A05: Intranasal Oxytocin Rat 50 Data
Table A06: Intranasal Oxytocin Rat 51 Data
Table A07: Intranasal Oxytocin Rat 54 Data
Table A08: Intranasal Oxytocin Rat 55 Data
Table A09: Intranasal Oxytocin Rat 58 Data
Table A10: Intranasal Oxytocin Rat 59 Data

Table A01: 0.04 mg Intranasal Oxytocin Rat 42 Data

Trigemina Rat 42	Route	Com-pound	Volume (uL)	uCi	mg	Time (min)	Perfused @ (min)
Amount Delivered:	IN	Oxytocin	44	41.00	0.040	14:01	31:01

Tissue Description	Tube #	Tube (g)	Tissue + Tube (g)	Tissue Weight (g)	CPM	nM (Spec. Act.)	nM (Stan.)
Dorsal Dura	1	2.99050	3.00015	0.00965	169	9.20	13.34
Ventral Dura	2	2.97726	2.98450	0.00724	119	8.63	12.52
Trigeminal Ganglion	3	2.98192	3.00475	0.02283	225	5.17	7.49
Maxillary Nerve	4	3.03144	3.04436	0.01292	149	6.04	8.75
Opthalmic Nerve	5	3.03160	3.03464	0.00304	23	4.04	5.86
Mandibular Nerve	6	2.97978	2.98647	0.00669	58	4.57	6.62
Upper Cervical Spinal Cord	7	2.98545	3.02174	0.03629	89	1.29	1.87
Olfactory Bulbs	8	2.98542	3.08527	0.09985	380	2.00	2.90
Anterior Olfactory Nucleus	9	2.97877	3.02220	0.04343	71	0.86	1.24
Frontal Cortex	10	2.97670	3.02615	0.04945	88	0.94	1.36
Caudate/Putamen	11	3.03418	3.04601	0.01183	37	1.63	2.36
Septal Nucleus	12	2.97729	2.98768	0.01039	49	2.47	3.58
Parietal Cortex	13	2.95332	3.00150	0.04818	63	0.68	0.99
Hippocampus	14	3.00003	3.03266	0.03263	55	0.88	1.27
Thalamus	15	2.98570	3.05692	0.07122	76	0.56	0.82
Hypothalamus	16	2.99831	3.03253	0.03422	37	0.57	0.83
Midbrain	17	2.95318	3.05411	0.10093	129	0.67	0.97
Pons	18	2.94860	3.01638	0.06778	95	0.73	1.07
Medulla	19	3.03292	3.14395	0.11103	173	0.82	1.19
Cerebellum	20	2.98115	3.24528	0.26413	358	0.71	1.03
Superficial Nodes (2)	21	2.99392	3.07652	0.08260	7914	50.32	72.96
Cervical Nodes (2)	22	2.97272	2.98781	0.01509	1083	37.68	54.63
Thyroid	23	2.97501	2.99773	0.02272	20353	470.46	682.16
Olfactory Epithelium	24	2.96018	3.01356	0.05338	1615	15.88	23.03
Respiratory Epithelium	25	2.98074	3.03969	0.05895	7170011	63,876.45	92,619.55
Axillary Nodes (2)	26	2.97257	3.01444	0.04187	75	0.94	1.36
Muscle (R, deltoid)	27	2.97866	3.00404	0.02538	38	0.79	1.15
Liver (R, superficial lobe)	28	2.94927	2.97412	0.02485	27	0.56	0.82
Kidney (L, tip)	29	2.98618	3.00920	0.02302	153	3.49	5.06
Lung (R, top lobe)	30	2.99626	3.00398	0.00772	43	2.94	4.26
Trachea (near cut-off)	31	2.96958	2.98401	0.01443	27	1.00	1.45
Esophagus (near cut-off)	32	2.99054	3.00383	0.01329	34	1.33	1.93
Spleen (tip)	33	2.97985	2.99870	0.01885	65	1.80	2.61
Heart	34	2.95214	2.98230	0.03016	44	0.76	1.11
Urine (fill to line of tube)	35	2.94895	3.06478	0.11583	643	2.91	4.23
Spinal Dura	36	2.97820	2.98371	0.00551	9	0.84	1.22
Lower Cervical Spinal Cord	37	2.94614	3.06525	0.11911	102	0.45	0.65
Thoracic Spinal Cord	38	2.96530	3.06651	0.10121	78	0.40	0.58
Lumbar Spinal Cord	39	2.97241	3.07740	0.10499	83	0.42	0.60
Blood Sample	40	2.98244	3.28836	0.30592	2688	4.61	6.69
Drug Standard	41	2.97704	2.97976	0.00272	3475052		
Drug Standard	42	2.95510	2.95777	0.00267	3539290		
Drug Standard	43	2.96602	2.96866	0.00264	3546673		
						1.904 cpm/fmol	1.313 cpm/fmol

Auto Calculations		Enter Values	
Total uL of Dose Sol =	58.4	Oxytocin Molecular Weight	1,007
Hot mg =	0.0000	Date of Synthesis =	03/10/08
Cold mg =	0.0526	Hot uCi/uL at Synthesis =	**1.000**
Total mg in Dose Sol =	0.0526	Hot uCi/ug at Synthesis =	**2184.30**
Conc Of Dose Sol (ug/uL) =	0.9000	Cold ug/uL =	20.00
Days after Synthesis Delivered =	3	Desired uCi =	41.0
Decay on Day Delivered =	0.9659	Desired mg =	0.040
Total uCi in Dose Sol on Day Delivered =	53.91	Estimated Desired nmol =	39.7
uCi/uL in Dose Sol on Day Delivered =	0.9225	**Date of Delivery =**	**03/13/08**
mg delivered =	0.040	**uL delivered (actual) =**	**44.4**
uCi delivered =	41.00	**Date of Counting =**	**03/13/08**
Estimated nmol delivered =	39.71	**Dose Calculator**	
Days after Synthesis Counted =	3	uL Hot to Deliver =	42.45
Decay on Day Counted =	0.9659	uL Cold to Deliver =	2.00
Specific Activity from Specs (cpm/fmol) =	1.904	Total uL to Deliver =	44.44
Avg. Volume of Standard (uL)* =	3.0	Hot to Cold Ratio =	21.23
Avg. CPM-bkgd of Standard =	3,520,338	Total uL to Make =	58.44
Specific Activity from Standards: (CPM/fmol) =	1.313	Dose Solution uL Hot =	55.82
Date of Disposal from Decay Storage =	10/31/09	Dose Solution uL Cold =	2.63
Average Background=	25.00	Efficiency of Counter=	83.08

*assume 3 uL

Table A02: 0.04 mg Intranasal Oxytocin Rat 43 Data

Trigemina Rat 43	Route	Com-pound	Volume (uL)	uCi	mg	Time (min)	Perfused @ (min)
Amount Delivered:	IN	Oxytocin	44	41.00	0.040	14:04	30:57

Tissue Description	Tube #	Tube (g)	Tissue + Tube (g)	Tissue Weight (g)	CPM	nM (Spec. Act.)	nM (Stan.)
Dorsal Dura	1	2.98521	2.99527	0.01006	2195	114.58	170.03
Ventral Dura	2	2.95690	2.96673	0.00983	880	47.04	69.80
Trigeminal Ganglion	3	2.97811	3.00671	0.02860	337	6.19	9.19
Maxillary Nerve	4	2.98860	3.00040	0.01180	94	4.18	6.20
Opthalmic Nerve	5	2.98178	2.98410	0.00232	32	7.13	10.58
Mandibular Nerve	6	2.95229	2.95462	0.00233	44	9.85	14.62
Upper Cervical Spinal Cord	7	2.96428	3.01973	0.05545	334	3.16	4.69
Olfactory Bulbs	8	2.99281	3.04388	0.05107	284	2.92	4.33
Anterior Olfactory Nucleus	9	2.98571	3.01269	0.02698	244	4.75	7.05
Frontal Cortex	10	2.97205	3.00083	0.02878	201	3.67	5.45
Caudate/Putamen	11	2.98470	2.99620	0.01150	20	0.91	1.35
Septal Nucleus	12	2.97616	2.99210	0.01594	23	0.75	1.12
Parietal Cortex	13	2.95444	2.99922	0.04478	318	3.73	5.53
Hippocampus	14	2.95529	2.97605	0.02076	26	0.67	0.99
Thalamus	15	3.00133	3.05580	0.05447	78	0.76	1.12
Hypothalamus	16	2.99070	3.00881	0.01811	53	1.54	2.29
Midbrain	17	2.98043	3.11021	0.12978	252	1.02	1.52
Pons	18	2.95288	3.03197	0.07909	331	2.19	3.26
Medulla	19	2.97975	3.08769	0.10794	403	1.96	2.91
Cerebellum	20	2.97704	3.22735	0.25031	2183	4.58	6.80
Superficial Nodes (2)	21	3.02877	3.11346	0.08469	1984	12.30	18.25
Cervical Nodes (2)	22	2.97540	2.98694	0.01154	128	5.83	8.66
Thyroid	23	2.98811	3.01505	0.02694	1548	30.18	44.78
Olfactory Epithelium	24	2.95713	3.03916	0.08203	420809	2,694.12	3,997.84
Respiratory Epithelium	25	2.98360	3.05101	0.06741	7311278	56,960.50	84,524.54
Axillary Nodes (2)	26	2.94250	2.96219	0.01969	33	0.87	1.29
Muscle (R, deltoid)	27	2.95922	2.98877	0.02955	55	0.98	1.46
Liver (R, superficial lobe)	28	2.95092	2.98225	0.03133	32	0.54	0.80
Kidney (L, tip)	29	2.97123	3.00429	0.03306	54	0.86	1.28
Lung (R, top lobe)	30	2.96880	2.98342	0.01462	56	2.01	2.99
Trachea (near cut-off)	31	2.97444	2.98824	0.01380	35	1.33	1.97
Esophagus (near cut-off)	32	2.99234	3.00316	0.01082	26	1.25	1.85
Spleen (tip)	33	2.98436	3.01511	0.03075	34	0.58	0.86
Heart	34	2.94326	2.96055	0.01729	32	0.98	1.46
Urine (fill to line of tube)	35	2.97844	3.10345	0.12501	145	0.61	0.91
Spinal Dura	36	2.94643	2.95623	0.00980	19	1.01	1.50
Lower Cervical Spinal Cord	37	3.06798	3.19276	0.12478	98	0.41	0.61
Thoracic Spinal Cord	38	2.97171	3.07038	0.09867	76	0.40	0.60
Lumbar Spinal Cord	39	2.96128	3.06910	0.10782	80	0.39	0.58
Blood Sample	40	3.01546	3.22838	0.21292	1901	4.69	6.96
Drug Standard	41	2.96995	2.97302	0.00307	3083515		
Drug Standard	42	2.95824	2.96087	0.00263	3601923		
Drug Standard	43	2.97378	2.97651	0.00273	3634058		
						1.904 cpm/fmol	1.283 cpm/fmol

Auto Calculations		Enter Values	
Total uL of Dose Sol =	58.4	Oxytocin Molecular Weight	1,007
Hot mg =	0.0000	Date of Synthesis =	03/10/08
Cold mg =	0.0526	Hot uCi/uL at Synthesis =	**1.000**
Total mg in Dose Sol =	0.0526	Hot uCi/ug at Synthesis =	**2184.30**
Conc Of Dose Sol (ug/uL) =	0.9000	Cold ug/uL =	20.00
Days after Synthesis Delivered =	3	Desired uCi =	41.0
Decay on Day Delivered =	0.9659	Desired mg =	0.040
Total uCi in Dose Sol on Day Delivered =	53.91	Estimated Desired nmol =	397.1
uCi/uL in Dose Sol on Day Delivered =	0.9225	**Date of Delivery =**	**03/13/08**
mg delivered =	0.040	**uL delivered (actual) =**	**44.4**
uCi delivered =	41.00	**Date of Counting =**	**03/13/08**
Estimated nmol delivered =	39.71	**Dose Calculator**	
Days after Synthesis Counted =	3	uL Hot to Deliver =	42.45
Decay on Day Counted =	0.9659	uL Cold to Deliver =	2.00
Specific Activity from Specs (cpm/fmol) =	1.904	Total uL to Deliver =	44.44
Avg. Volume of Standard (uL)* =	3.0	Hot to Cold Ratio =	21.23
Avg. CPM-bkgd of Standard =	3,439,832	Total uL to Make =	58.44
Specific Activity from Standards: (CPM/fmol) =	1.283	Dose Solution uL Hot =	55.82
Date of Disposal from Decay Storage =	10/31/09	Dose Solution uL Cold =	2.63
Average Background=	25.00	Efficiency of Counter=	83.08

*assume 3 uL

Table A03: 0.04 mg Intranasal Oxytocin Rat 46 Data

**Trigemina Rat 46**	Route	Com-pound	Volume (uL)	uCi	mg	Time (min)	Perfused @ (min)
Amount Delivered:	IN	Oxytocin	47	41.00	0.040	14:00	30:36

Tissue Description	Tube #	Tube (g)	Tissue + Tube (g)	Tissue Weight (g)	CPM	nM (Spec. Act.)	nM (Stan.)
Dorsal Dura	1	3.02796	3.04773	0.01977	2186	58.07	80.41
Ventral Dura	2	2.98364	3.00215	0.01851	394	11.19	15.49
Trigeminal Ganglion	3	2.95337	2.97663	0.02326	686	15.49	21.44
Maxillary Nerve	4	2.97884	2.99090	0.01206	183	7.95	11.00
Opthalmic Nerve	5	2.98899	2.99219	0.00320	48	7.81	10.82
Mandibular Nerve	6	2.97425	2.97722	0.00297	144	25.37	35.13
Upper Cervical Spinal Cord	7	2.97150	3.03344	0.06194	199	1.68	2.33
Olfactory Bulbs	8	2.99804	3.08156	0.08352	379	2.38	3.30
Anterior Olfactory Nucleus	9	2.99815	3.04099	0.04284	92	1.13	1.57
Frontal Cortex	10	2.98687	3.03045	0.04358	86	1.04	1.44
Caudate/Putamen	11	2.95049	2.96170	0.01121	23	1.07	1.49
Septal Nucleus	12	2.97796	2.98998	0.01202	23	1.02	1.41
Parietal Cortex	13	2.95776	3.00443	0.04667	84	0.95	1.31
Hippocampus	14	2.98269	3.01721	0.03452	55	0.84	1.16
Thalamus	15	2.99370	3.06652	0.07282	104	0.75	1.04
Hypothalamus	16	2.98642	3.01659	0.03017	89	1.55	2.15
Midbrain	17	2.98521	3.09260	0.10739	200	0.98	1.36
Pons	18	2.98613	3.04867	0.06254	133	1.12	1.55
Medulla	19	2.97710	3.08558	0.10848	194	0.94	1.30
Cerebellum	20	2.98320	3.25780	0.27460	488	0.93	1.29
Superficial Nodes (2)	21	2.99041	3.05877	0.06836	3783	29.06	40.24
Cervical Nodes (2)	22	2.95443	2.96596	0.01153	406	18.48	25.59
Thyroid	23	2.99327	3.01133	0.01806	1599	46.51	64.39
Olfactory Epithelium	24	2.98796	3.06220	0.07424	7614	53.86	74.57
Respiratory Epithelium	25	3.07243	3.12490	0.05247	8675732	86,836.01	120,227.43
Axillary Nodes (2)	26	2.99003	3.01438	0.02435	68	1.46	2.03
Muscle (R, deltoid)	27	2.96317	2.98566	0.02249	55	1.29	1.79
Liver (R, superficial lobe)	28	2.95492	2.98941	0.03449	66	1.00	1.39
Kidney (L, tip)	29	2.97860	3.00551	0.02691	121	2.36	3.26
Lung (R, top lobe)	30	2.97606	2.99186	0.01580	64	2.12	2.93
Trachea (near cut-off)	31	2.96368	2.97556	0.01188	56	2.46	3.41
Esophagus (near cut-off)	32	2.96596	2.97692	0.01096	41	1.96	2.71
Spleen (tip)	33	3.00121	3.04092	0.03971	90	1.18	1.64
Heart	34	3.02303	3.05359	0.03056	46	0.79	1.10
Urine (fill to line of tube)	35	2.97231	3.15169	0.17938	887	2.60	3.60
Spinal Dura	36	2.97063	2.97625	0.00562	45	4.23	5.86
Lower Cervical Spinal Cord	37	2.97902	3.09844	0.11942	201	0.88	1.23
Thoracic Spinal Cord	38	2.96411	3.05220	0.08809	96	0.57	0.79
Lumbar Spinal Cord	39	2.96741	3.09653	0.12912	149	0.61	0.84
Blood Sample	40	2.98429	3.32351	0.33922	4561	7.06	9.78
Drug Standard	41	2.99291	2.99540	0.00249	3462876		
Drug Standard	42	2.97024	2.97264	0.00240	3483020		
Drug Standard	43	3.03936	3.04188	0.00252	3520120		
						1.904 cpm/fmol	1.375 cpm/fmol

Auto Calculations		Enter Values	
Total uL of Dose Sol =	61.0	Oxytocin Molecular Weight	1,007
Hot mg =	0.0000	Date of Synthesis =	03/10/08
Cold mg =	0.0519	Hot uCi/uL at Synthesis =	**1.000**
Total mg in Dose Sol =	0.0519	Hot uCi/ug at Synthesis =	**2184.30**
Conc Of Dose Sol (ug/uL) =	0.8516	Cold ug/uL =	20.00
Days after Synthesis Delivered =	8	Desired uCi =	41.0
Decay on Day Delivered =	0.9117	Desired mg =	0.040
Total uCi in Dose Sol on Day Delivered =	53.22	Estimated Desired nmol =	397.1
uCi/uL in Dose Sol on Day Delivered =	0.8729	**Date of Delivery =**	**03/18/08**
mg delivered =	0.040	**uL delivered (actual) =**	**47.0**
uCi delivered =	41.00	**Date of Counting =**	**03/18/08**
Estimated nmol delivered =	39.72	**Dose Calculator**	
Days after Synthesis Counted =	8	uL Hot to Deliver =	44.97
Decay on Day Counted =	0.9117	uL Cold to Deliver =	2.00
Specific Activity from Specs (cpm/fmol) =	1.904	Total uL to Deliver =	46.97
Avg. Volume of Standard (uL)* =	3.0	Hot to Cold Ratio =	22.50
Avg. CPM-bkgd of Standard =	3,488,672	Total uL to Make =	60.97
Specific Activity from Standards: (CPM/fmol) =	1.375	Dose Solution uL Hot =	58.37
Date of Disposal from Decay Storage =	10/31/09	Dose Solution uL Cold =	2.59
Average Background=	25.00	Efficiency of Counter=	83.08

*assume 3 uL

Table A04: 0.04 mg Intranasal Oxytocin Rat 47 Data

Trigemina Rat 47	Route	Com-pound	Volume (uL)	uCi	mg	Time (min)	Perfused @ (min)
Amount Delivered:	IN	Oxytocin	47	41.00	0.040	14:00	30:46

Tissue Description	Tube #	Tube (g)	Tissue + Tube (g)	Tissue Weight (g)	CPM	nM (Spec. Act.)	nM (Stan.)
Dorsal Dura	1	2.98779	3.00120	0.01341	2138	83.71	115.91
Ventral Dura	2	2.98150	2.99090	0.00940	162	9.07	12.55
Trigeminal Ganglion	3	2.99245	3.01556	0.02311	84	1.91	2.65
Maxillary Nerve	4	3.03729	3.04822	0.01093	607	29.14	40.35
Opthalmic Nerve	5	2.99107	2.99398	0.00291	148	26.62	36.86
Mandibular Nerve	6	2.98316	2.99049	0.00733	485	34.77	48.14
Upper Cervical Spinal Cord	7	2.98588	3.00766	0.02178	61	1.46	2.02
Olfactory Bulbs	8	2.98815	3.05458	0.06643	265	2.09	2.90
Anterior Olfactory Nucleus	9	2.99280	3.03735	0.04455	7062	83.25	115.26
Frontal Cortex	10	3.03393	3.08126	0.04733	415	4.61	6.38
Caudate/Putamen	11	2.98377	2.99617	0.01240	72	3.04	4.20
Septal Nucleus	12	2.95657	2.96734	0.01077	49	2.40	3.33
Parietal Cortex	13	2.98383	3.03418	0.05035	215	2.24	3.10
Hippocampus	14	3.06544	3.09193	0.02649	51	1.02	1.41
Thalamus	15	2.98972	3.05209	0.06237	170	1.43	1.98
Hypothalamus	16	3.03132	3.06059	0.02927	81	1.45	2.00
Midbrain	17	2.98206	3.08742	0.10536	724	3.61	5.00
Pons	18	2.99870	3.06178	0.06308	568	4.73	6.54
Medulla	19	2.99163	3.09125	0.09962	571	3.01	4.17
Cerebellum	20	2.96819	3.22591	0.25772	8670	17.67	24.46
Superficial Nodes (2)	21	2.97129	3.03092	0.05963	5761	50.74	70.25
Cervical Nodes (2)	22	2.96645	2.98035	0.01390	130	4.90	6.79
Thyroid	23	3.00288	3.03315	0.03027	2946	51.11	70.76
Olfactory Epithelium	24	2.97767	3.03903	0.06136	459575	3,933.47	5,446.02
Respiratory Epithelium	25	2.98597	3.09304	0.10707	4172222	20,464.66	28,334.01
Axillary Nodes (2)	26	2.97429	3.00109	0.02680	75	1.46	2.03
Muscle (R, deltoid)	27	3.02020	3.05127	0.03107	74	1.25	1.72
Liver (R, superficial lobe)	28	3.05783	3.07973	0.02190	247	5.93	8.21
Kidney (L, tip)	29	2.98836	3.02732	0.03896	435	5.87	8.12
Lung (R, top lobe)	30	2.97500	2.99813	0.02313	59	1.34	1.86
Trachea (near cut-off)	31	2.98955	3.00013	0.01058	59	2.93	4.06
Esophagus (near cut-off)	32	2.95088	2.96398	0.01310	101	4.05	5.61
Spleen (tip)	33	2.98284	3.01933	0.03649	91	1.31	1.82
Heart	34	2.96076	2.98493	0.02417	65	1.41	1.95
Urine (fill to line of tube)	35	2.97748	3.17222	0.19474	709	1.91	2.65
Spinal Dura	36	2.98082	2.98801	0.00719	39	2.84	3.93
Lower Cervical Spinal Cord	37	2.98260	3.12707	0.14447	193	0.70	0.97
Thoracic Spinal Cord	38	2.95584	3.05119	0.09535	99	0.55	0.76
Lumbar Spinal Cord	39	3.02638	3.14129	0.11491	120	0.55	0.76
Blood Sample	40	2.98915	3.27922	0.29007	3657	6.62	9.17
Drug Standard	41	2.99291	2.99540	0.00249	3462876		
Drug Standard	42	2.97024	2.97264	0.00240	3483020		
Drug Standard	43	3.03936	3.04188	0.00252	3520120		
						1.904 cpm/fmol	1.375 cpm/fmol

Auto Calculations		Enter Values	
Total uL of Dose Sol =	61.0	Oxytocin Molecular Weight	1,007
Hot mg =	0.0000	Date of Synthesis =	03/10/08
Cold mg =	0.0519	Hot uCi/uL at Synthesis =	**1.000**
Total mg in Dose Sol =	0.0519	Hot uCi/ug at Synthesis =	**2184.30**
Conc Of Dose Sol (ug/uL) =	0.8516	Cold ug/uL =	20.00
Days after Synthesis Delivered =	8	Desired uCi =	41.0
Decay on Day Delivered =	0.9117	Desired mg =	0.040
Total uCi in Dose Sol on Day Delivered =	53.22	Estimated Desired nmol =	397.1
uCi/uL in Dose Sol on Day Delivered =	0.8729	**Date of Delivery =**	**03/18/08**
mg delivered =	0.040	**uL delivered (actual) =**	**47.0**
uCi delivered =	41.00	**Date of Counting =**	**03/18/08**
Estimated nmol delivered =	39.72	**Dose Calculator**	
Days after Synthesis Counted =	8	uL Hot to Deliver =	44.97
Decay on Day Counted =	0.9117	uL Cold to Deliver =	2.00
Specific Activity from Specs (cpm/fmol) =	1.904	Total uL to Deliver =	46.97
Avg. Volume of Standard (uL)* =	3.0	Hot to Cold Ratio =	22.50
Avg. CPM-bkgd of Standard =	3,488,672	Total uL to Make =	60.97
Specific Activity from Standards: (CPM/fmol) =	1.375	Dose Solution uL Hot =	58.37
Date of Disposal from Decay Storage =	10/31/09	Dose Solution uL Cold =	2.59
Average Background=	25.00	Efficiency of Counter=	83.08

*assume 3 uL

Table A05: 0.04 mg Intranasal Oxytocin Rat 50 Data

**Trigemina Rat 50**	Route	Com-pound	Volume (uL)	uCi	mg	Time (min)	Perfused @ (min)
Amount Delivered:	IN	Oxytocin	50	41.00	0.040	14:02	31:47

Tissue Description	Tube #	Tube (g)	Tissue + Tube (g)	Tissue Weight (g)	CPM	nM (Spec. Act.)	nM (Stan.)
Dorsal Dura	1	2.99010	3.00528	0.01518	546	18.93	25.46
Ventral Dura	2	2.97155	2.98825	0.01670	340	10.72	14.42
Trigeminal Ganglion	3	2.96501	2.99117	0.02616	263	5.29	7.12
Maxillary Nerve	4	2.98577	2.99921	0.01344	168	6.58	8.85
Opthalmic Nerve	5	2.96910	2.97345	0.00435	47	5.66	7.61
Mandibular Nerve	6	2.95921	2.96377	0.00456	81	9.33	12.55
Upper Cervical Spinal Cord	7	3.03072	3.09158	0.06086	100	0.87	1.17
Olfactory Bulbs	8	3.00425	3.07661	0.07236	587	4.27	5.74
Anterior Olfactory Nucleus	9	2.98778	3.02881	0.04103	105	1.35	1.81
Frontal Cortex	10	2.96492	3.01293	0.04801	147	1.61	2.17
Caudate/Putamen	11	2.96992	2.98176	0.01184	25	1.10	1.48
Septal Nucleus	12	2.98748	3.00338	0.01590	31	1.02	1.37
Parietal Cortex	13	2.92422	2.96719	0.04297	80	0.98	1.32
Hippocampus	14	2.98436	3.03699	0.05263	90	0.90	1.21
Thalamus	15	3.00835	3.08202	0.07367	131	0.93	1.25
Hypothalamus	16	3.06975	3.09697	0.02722	49	0.94	1.26
Midbrain	17	2.98461	3.05695	0.07234	105	0.76	1.03
Pons	18	3.01067	3.05972	0.04905	80	0.86	1.15
Medulla	19	2.97664	3.08383	0.10719	154	0.76	1.02
Cerebellum	20	2.98525	3.25281	0.26756	404	0.79	1.07
Superficial Nodes (2)	21	2.98303	3.03524	0.05221	2300	23.17	31.16
Cervical Nodes (2)	22	2.98644	2.99864	0.01220	193	8.31	11.18
Thyroid	23	2.97026	2.99118	0.02092	1601	40.25	54.13
Olfactory Epithelium	24	2.98471	3.05284	0.06813	20246	156.27	210.17
Respiratory Epithelium	25	2.98148	3.06784	0.08636	4529766	27,583.78	37,096.52
Axillary Nodes (2)	26	2.97833	2.99143	0.01310	25	0.98	1.32
Muscle (R, deltoid)	27	2.96100	2.98748	0.02648	49	0.98	1.32
Liver (R, superficial lobe)	28	2.97226	3.02939	0.05713	68	0.63	0.84
Kidney (L, tip)	29	2.98696	3.03470	0.04774	225	2.48	3.33
Lung (R, top lobe)	30	2.97860	2.98977	0.01117	24	1.14	1.54
Trachea (near cut-off)	31	3.03840	3.05491	0.01651	49	1.54	2.08
Esophagus (near cut-off)	32	3.02042	3.03468	0.01426	50	1.85	2.49
Spleen (tip)	33	2.97479	3.00954	0.03475	62	0.94	1.27
Heart	34	2.97024	3.01440	0.04416	62	0.73	0.99
Urine (fill to line of tube)	35	2.98836	3.25193	0.26357	2025	4.04	5.43
Spinal Dura	36	2.98537	2.99514	0.00977	37	2.00	2.69
Lower Cervical Spinal Cord	37	2.98347	3.11603	0.13256	142	0.56	0.76
Thoracic Spinal Cord	38	2.98152	3.09606	0.11454	95	0.44	0.59
Lumbar Spinal Cord	39	2.95414	3.09879	0.14465	118	0.43	0.58
Blood Sample	40	3.06940	3.41364	0.34424	3390	5.18	6.96
Drug Standard	41	2.97560	2.97816	0.00256	3355732	0.00	0.00
Drug Standard	42	2.96919	2.97144	0.00225	3383130	0.00	0.00
Drug Standard	43	2.97702	2.97927	0.00225	3329599	0.00	0.00
Circle of Willis/Basilar Artery	44	X	X	X	X	X	X
Renal Artery	45	X	X	X	X	X	X
Blank	46	0.00000	0.00000	0.00000	2	0.00	0.00
Blank	47	0.00000	0.00000	0.00000	10	0.00	0.00
						1.902 cpm/fmol	1.414 cpm/fmol

Auto Calculations		Enter Values	
Total uL of Dose Sol =	64.1947	Oxytocin Molecular Weight	**1007.190**
Hot mg =	0.0000	Date of Synthesis =	**39517.000**
Cold mg =	0.0511	Hot uCi/uL at Synthesis =	**1.000**
Total mg in Dose Sol =	0.0512	Hot uCi/ug at Synthesis =	**2184.30**
Conc Of Dose Sol (ug/uL) =	0.7969	Cold ug/uL =	20.00
Days after Synthesis Delivered =	14	Desired uCi =	41.0
Decay on Day Delivered =	0.8507	Desired mg =	0.040
Total uCi in Dose Sol on Day Delivered =	52.44	Estimated Desired nmol =	397.1
uCi/uL in Dose Sol on Day Delivered =	0.8168	**Date of Delivery =**	**03/24/08**
mg delivered =	0.040	**uL delivered (actual) =**	**50.2**
uCi delivered =	41.00	**Date of Counting =**	**03/24/08**
Estimated nmol delivered =	39.71		
Days after Synthesis Counted =	14	uL Hot to Deliver =	48.20
Decay on Day Counted =	0.8507	uL Cold to Deliver =	2.00
Specific Activity from Specs (cpm/fmol) =	1.902	Total uL to Deliver =	50.19
Avg. Volume of Standard (uL)* =	3.0	Hot to Cold Ratio =	24.11
Avg. CPM-bkgd of Standard =	3,356,154	Total uL to Make =	64.19
Specific Activity from Standards: (CPM/fmol) =	1.414	Dose Solution uL Hot =	61.64
Date of Disposal from Decay Storage =	10/31/09	Dose Solution uL Cold =	2.56
Average Background=	28.24	Efficiency of Counter=	82.97

Table A06: 0.04 mg Intranasal Oxytocin Rat 51 Data

Trigemina Rat 51	Route	Com-pound	Volume (uL)	uCi	mg	Time (min)	Perfused @ (min)
Amount Delivered:	IN	Oxytocin	50	41.00	0.040	14:00	30:07

Tissue Description	Tube #	Tube (g)	Tissue + Tube (g)	Tissue Weight (g)	CPM	nM (Spec. Act.)	nM (Stan.)
Dorsal Dura	1	2.99317	3.00429	0.01112	540	25.53	34.34
Ventral Dura	2	2.98079	3.00158	0.02079	1352	34.20	46.00
Trigeminal Ganglion	3	2.97072	2.99642	0.02570	297	6.08	8.18
Maxillary Nerve	4	3.06195	3.07537	0.01342	226	8.85	11.91
Opthalmic Nerve	5	2.98869	2.99181	0.00312	44	7.40	9.95
Mandibular Nerve	6	2.99946	3.00293	0.00347	46	6.99	9.40
Upper Cervical Spinal Cord	7	3.00843	3.05620	0.04777	133	1.47	1.98
Olfactory Bulbs	8	2.96720	3.03490	0.06770	238	1.85	2.48
Anterior Olfactory Nucleus	9	2.98584	3.02375	0.03791	86	1.19	1.60
Frontal Cortex	10	2.96492	3.00399	0.03907	117	1.58	2.12
Caudate/Putamen	11	3.00824	3.01839	0.01015	12	0.62	0.83
Septal Nucleus	12	2.98782	3.00398	0.01616	32	1.03	1.38
Parietal Cortex	13	2.99537	3.03911	0.04374	91	1.10	1.47
Hippocampus	14	2.97528	3.00843	0.03315	57	0.90	1.21
Thalamus	15	3.06547	3.12592	0.06045	98	0.85	1.15
Hypothalamus	16	2.99303	3.03208	0.03905	98	1.32	1.77
Midbrain	17	2.97316	3.09069	0.11753	277	1.24	1.67
Pons	18	2.99799	3.05381	0.05582	106	1.00	1.35
Medulla	19	2.99057	3.11812	0.12755	268	1.11	1.49
Cerebellum	20	2.98967	3.24829	0.25862	621	1.26	1.70
Superficial Nodes (2)	21	2.99203	3.04719	0.05516	1896	18.07	24.30
Cervical Nodes (2)	22	2.99907	3.00954	0.01047	277	13.89	18.68
Thyroid	23	2.98788	3.00491	0.01703	2814	86.89	116.86
Olfactory Epithelium	24	2.99503	3.07199	0.07696	5153	35.21	47.35
Respiratory Epithelium	25	2.96705	3.03449	0.06744	5353399	41,744.83	56,141.25
Axillary Nodes (2)	26	2.99217	3.01255	0.02038	55	1.41	1.89
Muscle (R, deltoid)	27	2.98113	3.01243	0.03130	95	1.60	2.15
Liver (R, superficial lobe)	28	2.96056	3.00947	0.04891	113	1.21	1.63
Kidney (L, tip)	29	2.97475	3.00786	0.03311	115	1.82	2.45
Lung (R, top lobe)	30	2.97743	2.98937	0.01194	35	1.54	2.07
Trachea (near cut-off)	31	3.03253	3.05650	0.02397	152	3.32	4.47
Esophagus (near cut-off)	32	2.98401	3.00172	0.01771	95	2.83	3.81
Spleen (tip)	33	2.94900	2.97266	0.02366	151	3.36	4.51
Heart	34	2.98129	3.01206	0.03077	60	1.03	1.39
Urine (fill to line of tube)	35	2.94920	3.21505	0.26585	2903	5.74	7.72
Spinal Dura	36	2.97919	2.99349	0.01430	69	2.55	3.43
Lower Cervical Spinal Cord	37	2.98858	3.14659	0.15801	218	0.72	0.97
Thoracic Spinal Cord	38	2.98937	3.10937	0.12000	130	0.57	0.77
Lumbar Spinal Cord	39	2.98455	3.13897	0.15442	192	0.65	0.88
Blood Sample	40	2.99402	3.24048	0.24646	3727	7.95	10.69
Drug Standard	41	2.97560	2.97816	0.00256	3355732		
Drug Standard	42	2.96919	2.97144	0.00225	3383130		
Drug Standard	43	2.97702	2.97927	0.00225	3329599		
Circle of Willis/Basilar Artery	44	0.01165	0.01494	0.00329	635	101.55	136.57
Renal Artery	45	2.99626	3.00449	0.00823	28	1.81	2.43
Blank	46				2		
Blank	47				10		
						1.902 cpm/fmol	1.414 cpm/fmol

Auto Calculations		Enter Values	
Total uL of Dose Sol =	64.2	Oxytocin Molecular Weight	1,007
Hot mg =	0.0000	Date of Synthesis =	03/10/08
Cold mg =	0.0511	Hot uCi/uL at Synthesis =	**1.000**
Total mg in Dose Sol =	0.0512	Hot uCi/ug at Synthesis =	**2184.30**
Conc Of Dose Sol (ug/uL) =	0.7969	Cold ug/uL =	20.00
Days after Synthesis Delivered =	14	Desired uCi =	41.0
Decay on Day Delivered =	0.8507	Desired mg =	0.040
Total uCi in Dose Sol on Day Delivered =	52.44	Estimated Desired nmol =	397.1
uCi/uL in Dose Sol on Day Delivered =	0.8168	**Date of Delivery =**	**03/24/08**
mg delivered =	0.040	**uL delivered (actual) =**	**50.2**
uCi delivered =	41.00	**Date of Counting =**	**03/24/08**
Estimated nmol delivered =	39.71	**Dose Calculator**	
Days after Synthesis Counted =	14	uL Hot to Deliver =	48.20
Decay on Day Counted =	0.8507	uL Cold to Deliver =	2.00
Specific Activity from Specs (cpm/fmol) =	1.902	Total uL to Deliver =	50.19
Avg. Volume of Standard (uL)* =	3.0	Hot to Cold Ratio =	24.11
Avg. CPM-bkgd of Standard =	3,356,154	Total uL to Make =	64.19
Specific Activity from Standards: (CPM/fmol) =	1.414	Dose Solution uL Hot =	61.64
Date of Disposal from Decay Storage =	10/31/09	Dose Solution uL Cold =	2.56
Average Background=	25.80	Efficiency of Counter=	82.97

*assume 3 uL

Table A07: 0.04 mg Intranasal Oxytocin Rat 54 Data

Trigemina Rat 54	Route	Com-pound	Volume (uL)	uCi	mg	Time (min)	Perfused @ (min)
Amount Delivered:	IN	Oxytocin	54	41.00	0.040	14:00	30:58

Tissue Description	Tube #	Tube (g)	Tissue + Tube (g)	Tissue Weight (g)	CPM	nM (Spec. Act.)	nM (Stan.)
Dorsal Dura	1	2.93231	2.94289	0.01058	102	5.07	7.10
Ventral Dura	2	2.95895	2.97274	0.01379	154	5.88	8.23
Trigeminal Ganglion	3	2.97068	2.99751	0.02683	86	1.69	2.37
Maxillary Nerve	4	2.99796	3.01193	0.01397	85	3.19	4.47
Opthalmic Nerve	5	2.97888	2.98276	0.00388	50	6.81	9.54
Mandibular Nerve	6	2.97117	2.97473	0.00356	43	6.38	8.93
Upper Cervical Spinal Cord	7	2.97304	3.00415	0.03111	106	1.79	2.50
Olfactory Bulbs	8	2.96501	3.04788	0.08287	153	0.97	1.36
Anterior Olfactory Nucleus	9	2.98365	3.02660	0.04295	54	0.66	0.93
Frontal Cortex	10	2.96633	3.00434	0.03801	58	0.80	1.12
Caudate/Putamen	11	2.95251	2.96452	0.01201	10	0.42	0.59
Septal Nucleus	12	2.96540	2.97530	0.00990	10	0.54	0.76
Parietal Cortex	13	2.98931	3.03371	0.04440	52	0.62	0.87
Hippocampus	14	2.97709	3.01910	0.04201	59	0.74	1.04
Thalamus	15	2.97711	3.05943	0.08232	81	0.52	0.72
Hypothalamus	16	2.95586	2.98580	0.02994	32	0.57	0.79
Midbrain	17	2.97673	3.08383	0.10710	116	0.57	0.80
Pons	18	2.99941	3.06825	0.06884	69	0.53	0.74
Medulla	19	2.98160	3.10639	0.12479	117	0.49	0.69
Cerebellum	20	2.94879	3.22441	0.27562	337	0.64	0.90
Superficial Nodes (2)	21	2.99836	3.06034	0.06198	1566	13.28	18.60
Cervical Nodes (2)	22	2.96623	2.97880	0.01257	318	13.27	18.59
Thyroid	23	2.99741	3.01818	0.02077	1973	49.92	69.92
Olfactory Epithelium	24	2.95677	3.04304	0.08627	24275	147.88	207.11
Respiratory Epithelium	25	2.97319	3.03173	0.05854	1287107	11,554.68	16,183.08
Axillary Nodes (2)	26	2.96494	2.97916	0.01422	52	1.92	2.69
Muscle (R, deltoid)	27	2.98567	3.00459	0.01892	38	1.05	1.47
Liver (R, superficial lobe)	28	2.92716	2.95377	0.02661	27	0.54	0.75
Kidney (L, tip)	29	2.92259	2.93899	0.01640	42	1.35	1.88
Lung (R, top lobe)	30	2.97639	2.98700	0.01061	18	0.90	1.26
Trachea (near cut-off)	31	2.96325	2.98617	0.02292	61	1.40	1.97
Esophagus (near cut-off)	32	2.95827	2.96467	0.00640	22	1.78	2.50
Spleen (tip)	33	2.94680	2.96638	0.01958	56	1.50	2.10
Heart	34	2.95836	2.97451	0.01615	25	0.81	1.14
Urine (fill to line of tube)	35	2.96917	3.23326	0.26409	2345	4.67	6.54
Spinal Dura	36	2.94506	2.96430	0.01924	154	4.20	5.88
Lower Cervical Spinal Cord	37	2.97051	3.12791	0.15740	125	0.42	0.58
Thoracic Spinal Cord	38	2.96378	3.07670	0.11292	91	0.42	0.59
Lumbar Spinal Cord	39	2.96376	3.08715	0.12339	92	0.39	0.55
Blood Sample	40	2.94669	3.20655	0.25986	3298	6.67	9.34
Drug Standard	41	2.97858	2.98082	0.00224	3076740		
Drug Standard	42	2.95702	2.95926	0.00224	2982622		
Drug Standard	43	2.97892	2.98112	0.00220	2893240		
Circle of Willis/Basilar Artery	44	0.01123	0.01468	0.00345	13	2.03	2.84
Renal Artery	45	2.96072	2.96943	0.00871	28	1.69	2.37
Blank	46				6		
Blank	47				8		
						1.903 cpm/fmol	1.359 cpm/fmol

Auto Calculations		Enter Values	
Total uL of Dose Sol =	68.3	Oxytocin Molecular Weight	1,007
Hot mg =	0.0000	Date of Synthesis =	03/10/08
Cold mg =	0.0503	Hot uCi/uL at Synthesis =	**1.000**
Total mg in Dose Sol =	0.0503	Hot uCi/ug at Synthesis =	**2184.30**
Conc Of Dose Sol (ug/uL) =	0.7373	Cold ug/uL =	20.00
Days after Synthesis Delivered =	21	Desired uCi =	41.0
Decay on Day Delivered =	0.7846	Desired mg =	0.040
Total uCi in Dose Sol on Day Delivered =	51.58	Estimated Desired nmol =	397.1
uCi/uL in Dose Sol on Day Delivered =	0.7557	**Date of Delivery =**	**03/31/08**
mg delivered =	0.040	**uL delivered (actual) =**	**54.3**
uCi delivered =	41.00	**Date of Counting =**	**03/31/08**
Estimated nmol delivered =	39.72	**Dose Calculator**	
Days after Synthesis Counted =	21	uL Hot to Deliver =	52.25
Decay on Day Counted =	0.7846	uL Cold to Deliver =	2.00
Specific Activity from Specs (cpm/fmol) =	1.903	Total uL to Deliver =	54.25
Avg. Volume of Standard (uL)* =	3.0	Hot to Cold Ratio =	26.14
Avg. CPM-bkgd of Standard =	2,984,201	Total uL to Make =	68.25
Specific Activity from Standards: (CPM/fmol) =	1.359	Dose Solution uL Hot =	65.74
Date of Disposal from Decay Storage =	10/31/09	Dose Solution uL Cold =	2.51
Average Background=	25.96	Efficiency of Counter=	83.04

Table A08: 0.04 mg Intranasal Oxytocin Rat 55 Data

Trigemina Rat 55	Route	Com-pound	Volume (uL)	uCi	mg	Time (min)	Perfused @ (min)
Amount Delivered:	IN	Oxytocin	54	41.00	0.040	14:03	31:10

Tissue Description	Tube #	Tube (g)	Tissue + Tube (g)	Tissue Weight (g)	CPM	nM (Spec. Act.)	nM (Stan.)
Dorsal Dura	1	2.97903	2.99096	0.01193	262	11.53	16.25
Ventral Dura	2	2.98049	2.99097	0.01048	228	11.43	16.10
Trigeminal Ganglion	3	2.94264	2.96434	0.02170	103	2.50	3.52
Maxillary Nerve	4	2.97538	2.99098	0.01560	95	3.20	4.51
Opthalmic Nerve	5	2.95205	2.95758	0.00553	36	3.40	4.79
Mandibular Nerve	6	2.99823	3.00425	0.00602	47	4.10	5.78
Upper Cervical Spinal Cord	7	2.93858	2.96564	0.02706	57	1.10	1.55
Olfactory Bulbs	8	2.97063	3.05223	0.08160	320	2.06	2.90
Anterior Olfactory Nucleus	9	2.96547	3.01532	0.04985	97	1.03	1.45
Frontal Cortex	10	2.97895	3.02438	0.04543	114	1.32	1.85
Caudate/Putamen	11	2.96601	2.97790	0.01189	10	0.46	0.65
Septal Nucleus	12	2.95813	2.97881	0.02068	31	0.80	1.12
Parietal Cortex	13	2.93532	2.98973	0.05441	80	0.78	1.09
Hippocampus	14	2.95695	2.99549	0.03854	45	0.61	0.86
Thalamus	15	2.96929	3.03241	0.06312	85	0.71	1.00
Hypothalamus	16	2.97939	3.01497	0.03558	55	0.81	1.14
Midbrain	17	2.96119	3.08513	0.12394	161	0.68	0.96
Pons	18	2.96636	3.04219	0.07583	101	0.70	0.98
Medulla	19	2.96845	3.06476	0.09631	143	0.78	1.10
Cerebellum	20	2.98573	3.26840	0.28267	411	0.76	1.08
Superficial Nodes (2)	21	2.93224	3.02468	0.09244	1328	7.55	10.64
Cervical Nodes (2)	22	2.97694	2.99073	0.01379	125	4.77	6.72
Thyroid	23	2.93836	2.96952	0.03116	5775	97.38	137.25
Olfactory Epithelium	24	2.95703	3.02701	0.06998	4382	32.90	46.37
Respiratory Epithelium	25	2.96863	3.07872	0.11009	7326000	34,964.95	49,278.01
Axillary Nodes (2)	26	2.94532	2.95990	0.01458	29	1.03	1.45
Muscle (R, deltoid)	27	2.95283	2.99142	0.03859	55	0.75	1.06
Liver (R, superficial lobe)	28	2.97723	2.99620	0.01897	32	0.89	1.26
Kidney (L, tip)	29	2.96436	2.98160	0.01724	43	1.30	1.83
Lung (R, top lobe)	30	2.97026	2.97822	0.00796	28	1.84	2.59
Trachea (near cut-off)	31	2.94458	2.96980	0.02522	78	1.62	2.28
Esophagus (near cut-off)	32	2.97501	2.98716	0.01215	27	1.16	1.63
Spleen (tip)	33	2.94112	2.95612	0.01500	68	2.37	3.34
Heart	34	2.96802	2.98810	0.02008	32	0.83	1.17
Urine (fill to line of tube)	35	2.95946	3.20461	0.24515	986	2.11	2.98
Spinal Dura	36	2.94104	2.94201	0.00097	2	1.19	1.68
Lower Cervical Spinal Cord	37	2.93805	3.07286	0.13481	107	0.42	0.59
Thoracic Spinal Cord	38	2.96842	3.10656	0.13814	89	0.34	0.48
Lumbar Spinal Cord	39	2.94809	3.07143	0.12334	84	0.36	0.50
Blood Sample	40	2.94607	3.16897	0.22290	2324	5.48	7.72
Drug Standard	41	2.97858	2.98082	0.00224	3029961		
Drug Standard	42	2.95702	2.95926	0.00224	2978113		
Drug Standard	43	2.97892	2.98112	0.00220	2888698		
Circle of Willis/Basilar Artery	44	0.01428	0.01687	0.00259	37	7.55	10.64
Renal Artery	45	2.96021	2.96625	0.00604	12	1.02	1.43
Blank	46				1		
Blank	47				0		
						1.903 cpm/fmol	1.350 cpm/fmol

Auto Calculations		Enter Values	
Total uL of Dose Sol =	68.3	Oxytocin Molecular Weight	1,007
Hot mg =	0.0000	Date of Synthesis =	03/10/08
Cold mg =	0.0503	Hot uCi/uL at Synthesis =	**1.000**
Total mg in Dose Sol =	0.0503	Hot uCi/ug at Synthesis =	**2184.30**
Conc Of Dose Sol (ug/uL) =	0.7373	Cold ug/uL =	20.00
Days after Synthesis Delivered =	21	Desired uCi =	41.0
Decay on Day Delivered =	0.7846	Desired mg =	0.040
Total uCi in Dose Sol on Day Delivered =	51.58	Estimated Desired nmol =	397.1
uCi/uL in Dose Sol on Day Delivered =	0.7557	**Date of Delivery =**	**03/31/08**
mg delivered =	0.040	**uL delivered (actual) =**	**54.25**
uCi delivered =	41.00	**Date of Counting =**	**03/31/08**
Estimated nmol delivered =	39.71	**Dose Calculator**	
Days after Synthesis Counted =	21	uL Hot to Deliver =	52.25
Decay on Day Counted =	0.7846	uL Cold to Deliver =	2.00
Specific Activity from Specs (cpm/fmol) =	1.903	Total uL to Deliver =	54.25
Avg. Volume of Standard (uL)* =	3.0	Hot to Cold Ratio =	26.14
Avg. CPM-bkgd of Standard =	2,965,590	Total uL to Make =	68.25
Specific Activity from Standards: (CPM/fmol) =	1.350	Dose Solution uL Hot =	65.74
Date of Disposal from Decay Storage =	10/31/09	Dose Solution uL Cold =	2.51
Average Background=	27.14	Efficiency of Counter=	83.04

*assume 3 uL

Table A09: 0.04 mg Intranasal Oxytocin Rat 58 Data

**Trigemina Rat 58**	Route	Com-pound	Volume (uL)	uCi	mg	Time (min)	Perfused @ (min)
Amount Delivered:	IN	Oxytocin	55	41.00	0.040	14:00	30:45

Tissue Description	Tube #	Tube (g)	Tissue + Tube (g)	Tissue Weight (g)	CPM	nM (Spec. Act.)	nM (Stan.)
Dorsal Dura	1	2.95078	2.96507	0.01429	790	29.05	39.87
Ventral Dura	2	2.96680	2.97881	0.01201	354	15.47	21.24
Trigeminal Ganglion	3	2.98498	3.01602	0.03104	139	2.36	3.24
Maxillary Nerve	4	3.01207	3.02825	0.01618	146	4.73	6.50
Opthalmic Nerve	5	2.98124	2.98531	0.00407	39	5.09	6.98
Mandibular Nerve	6	3.00280	3.00657	0.00377	26	3.57	4.90
Upper Cervical Spinal Cord	7	2.96182	3.00284	0.04102	91	1.17	1.61
Olfactory Bulbs	8	3.00703	3.09786	0.09083	625	3.61	4.96
Anterior Olfactory Nucleus	9	2.97392	3.01389	0.03997	105	1.38	1.89
Frontal Cortex	10	2.97263	3.01711	0.04448	381	4.50	6.17
Caudate/Putamen	11	2.96598	2.97695	0.01097	19	0.89	1.22
Septal Nucleus	12	2.96926	2.98795	0.01869	20	0.57	0.79
Parietal Cortex	13	2.96702	3.02175	0.05473	149	1.43	1.96
Hippocampus	14	2.98877	3.02641	0.03764	102	1.43	1.96
Thalamus	15	2.97678	3.07981	0.10303	171	0.87	1.20
Hypothalamus	16	2.95973	2.98274	0.02301	29	0.66	0.90
Midbrain	17	2.95726	3.07364	0.11638	162	0.73	1.00
Pons	18	3.01024	3.08641	0.07617	100	0.69	0.95
Medulla	19	2.96166	3.07441	0.11275	175	0.82	1.12
Cerebellum	20	2.97711	3.26821	0.29110	443	0.80	1.10
Superficial Nodes (2)	21	2.97961	3.02806	0.04845	1497	16.24	22.29
Cervical Nodes (2)	22	2.98823	2.99454	0.00631	60	5.01	6.87
Thyroid	23	3.01010	3.03697	0.02687	2824	55.22	75.81
Olfactory Epithelium	24	2.97400	3.06822	0.09422	14427	80.47	110.47
Respiratory Epithelium	25	2.98633	3.06509	0.07876	6874502	45,870.28	62,972.48
Axillary Nodes (2)	26	3.01071	3.02583	0.01512	28	0.98	1.35
Muscle (R, deltoid)	27	2.96422	2.98303	0.01881	32	0.89	1.23
Liver (R, superficial lobe)	28	2.95836	2.97454	0.01618	25	0.81	1.11
Kidney (L, tip)	29	2.96319	2.99990	0.03671	62	0.89	1.23
Lung (R, top lobe)	30	2.97247	2.99218	0.01971	38	1.02	1.41
Trachea (near cut-off)	31	2.96731	2.98403	0.01672	135	4.25	5.84
Esophagus (near cut-off)	32	2.95452	2.98411	0.02959	112	1.99	2.74
Spleen (tip)	33	2.95789	2.97731	0.01942	53	1.43	1.96
Heart	34	2.95685	2.97891	0.02206	24	0.56	0.77
Urine (fill to line of tube)	35	2.95188	3.21890	0.26702	1964	3.86	5.31
Spinal Dura	36	2.95802	2.96307	0.00505	0	0.00	0.00
Lower Cervical Spinal Cord	37	2.94874	3.09489	0.14615	89	0.32	0.44
Thoracic Spinal Cord	38	2.96661	3.08469	0.11808	61	0.27	0.38
Lumbar Spinal Cord	39	2.96963	3.11406	0.14443	165	0.60	0.83
Blood Sample	40	2.97104	3.23549	0.26445	3202	6.36	8.73
Drug Standard	41	2.96857	2.97157	0.00300	2995236		
Drug Standard	42	2.95233	2.95520	0.00287	2962188		
Drug Standard	43	2.98869	2.99139	0.00270	2974948		
Circle of Willis/Basilar Artery	44	2.94850	2.95224	0.00374	30	4.24	5.83
Renal Artery	45	2.97312	2.98510	0.01198	40	1.76	2.41
Blank	46				5		
Blank	47				0		
						1.903 cpm/fmol	1.386 cpm/fmol

**Auto Calculations**		**Enter Values**	
Total uL of Dose Sol =	69.5	Oxytocin Molecular Weight	1,007
Hot mg =	0.0000	Date of Synthesis =	03/10/08
Cold mg =	0.0501	Hot uCi/uL at Synthesis =	**1.000**
Total mg in Dose Sol =	0.0501	Hot uCi/ug at Synthesis =	**2184.30**
Conc Of Dose Sol (ug/uL) =	0.7211	Cold ug/uL =	20.00
Days after Synthesis Delivered =	23	Desired uCi =	41.0
Decay on Day Delivered =	0.7667	Desired mg =	0.040
Total uCi in Dose Sol on Day Delivered =	51.35	Estimated Desired nmol =	397.1
uCi/uL in Dose Sol on Day Delivered =	0.7391	**Date of Delivery =**	**04/02/08**
mg delivered =	0.040	**uL delivered (actual) =**	**55.5**
uCi delivered =	41.00	**Date of Counting =**	**04/02/08**
Estimated nmol delivered =	39.72	**Dose Calculator**	
Days after Synthesis Counted =	23	uL Hot to Deliver =	53.48
Decay on Day Counted =	0.7667	uL Cold to Deliver =	2.00
Specific Activity from Specs (cpm/fmol) =	1.903	Total uL to Deliver =	55.47
Avg. Volume of Standard (uL)* =	3.0	Hot to Cold Ratio =	26.75
Avg. CPM-bkgd of Standard =	2,977,457	Total uL to Make =	69.47
Specific Activity from Standards: (CPM/fmol) =	1.386	Dose Solution uL Hot =	66.97
Date of Disposal from Decay Storage =	10/31/09	Dose Solution uL Cold =	2.50
Average Background=	29.72	Efficiency of Counter=	83.04

*assume 3 uL

Table A10: 0.04 mg Intranasal Oxytocin Rat 59 Data

Trigemina Rat 59	Route	Com-pound	Volume (uL)	uCi	mg	Time (min)	Perfused @ (min)
Amount Delivered:	IN	Oxytocin	55	41.00	0.040	14:00	30:40

Tissue Description	Tube #	Tube (g)	Tissue + Tube (g)	Tissue Weight (g)	CPM	nM (Spec. Act.)	nM (Stan.)
Dorsal Dura	1	3.01854	3.02787	0.00933	472	26.58	36.46
Ventral Dura	2	2.98492	2.98993	0.00501	106	11.13	15.26
Trigeminal Ganglion	3	2.96298	2.98561	0.02263	129	3.00	4.12
Maxillary Nerve	4	2.98988	3.00223	0.01235	97	4.11	5.64
Opthalmic Nerve	5	2.95892	2.96302	0.00410	98	12.57	17.25
Mandibular Nerve	6	2.96259	2.96688	0.00429	49	5.97	8.18
Upper Cervical Spinal Cord	7	2.97467	3.02678	0.05211	105	1.06	1.45
Olfactory Bulbs	8	2.96749	3.03607	0.06858	517	3.96	5.43
Anterior Olfactory Nucleus	9	2.96789	3.00625	0.03836	66	0.90	1.23
Frontal Cortex	10	2.98814	3.03284	0.04470	98	1.15	1.58
Caudate/Putamen	11	3.00685	3.01747	0.01062	14	0.71	0.97
Septal Nucleus	12	2.97957	2.99611	0.01654	22	0.70	0.96
Parietal Cortex	13	2.96927	3.02106	0.05179	79	0.80	1.10
Hippocampus	14	3.01504	3.05627	0.04123	50	0.64	0.88
Thalamus	15	2.98556	3.05324	0.06768	68	0.52	0.72
Hypothalamus	16	2.97265	2.99902	0.02637	44	0.88	1.21
Midbrain	17	3.01995	3.12653	0.10658	131	0.65	0.89
Pons	18	3.00370	3.06390	0.06020	81	0.71	0.97
Medulla	19	2.95575	3.07375	0.11800	187	0.83	1.14
Cerebellum	20	2.98175	3.22789	0.24614	340	0.73	1.00
Superficial Nodes (2)	21	3.00301	3.06171	0.05870	1212	10.85	14.88
Cervical Nodes (2)	22	3.02309	3.03384	0.01075	179	8.74	11.98
Thyroid	23	2.99363	3.02070	0.02707	3266	63.41	86.96
Olfactory Epithelium	24	2.96627	3.01620	0.04993	1242	13.08	17.93
Respiratory Epithelium	25	3.01019	3.09783	0.08764	4063873	24,368.77	33,422.30
Axillary Nodes (2)	26	2.97014	2.98126	0.01112	28	1.32	1.81
Muscle (R, deltoid)	27	2.95914	2.98070	0.02156	39	0.96	1.32
Liver (R, superficial lobe)	28	2.98839	3.00759	0.01920	34	0.94	1.28
Kidney (L, tip)	29	2.94192	2.98214	0.04022	108	1.41	1.93
Lung (R, top lobe)	30	2.98913	3.00536	0.01623	51	1.66	2.28
Trachea (near cut-off)	31	2.98973	3.00956	0.01983	745	19.74	27.08
Esophagus (near cut-off)	32	2.96249	2.98068	0.01819	66	1.91	2.62
Spleen (tip)	33	2.96907	2.99270	0.02363	61	1.37	1.87
Heart	34	2.94376	2.98261	0.03885	36	0.48	0.66
Urine (fill to line of tube)	35	2.95774	3.21163	0.25389	1028	2.13	2.92
Spinal Dura	36	2.95141	2.96981	0.01840	48	1.37	1.88
Lower Cervical Spinal Cord	37	2.96841	3.09051	0.12210	91	0.39	0.54
Thoracic Spinal Cord	38	2.97356	3.06140	0.08784	47	0.28	0.38
Lumbar Spinal Cord	39	2.95727	3.05940	0.10213	76	0.39	0.54
Blood Sample	40	2.95970	3.24014	0.28044	3339	6.26	8.58
Drug Standard	41	2.96857	2.97157	0.00300	3010291		
Drug Standard	42	2.95233	2.95520	0.00287	2959285		
Drug Standard	43	2.98869	2.99139	0.00270	2971371		
Circle of Willis/Basilar Artery	44	2.97330	2.97611	0.00281	34	6.28	8.62
Renal Artery	45	2.97154	2.97806	0.00652	16	1.32	1.81
Blank	46				7		
Blank	47				4		
						1.903 cpm/fmol	1.387 cpm/fmol

Auto Calculations		Enter Values	
Total uL of Dose Sol =	69.5	Oxytocin Molecular Weight	1,007
Hot mg =	0.0000	Date of Synthesis =	03/10/08
Cold mg =	0.0501	Hot uCi/uL at Synthesis =	**1.000**
Total mg in Dose Sol =	0.0501	Hot uCi/ug at Synthesis =	**2184.30**
Conc Of Dose Sol (ug/uL) =	0.7211	Cold ug/uL =	20.00
Days after Synthesis Delivered =	23	Desired uCi =	41.0
Decay on Day Delivered =	0.7667	Desired mg =	0.040
Total uCi in Dose Sol on Day Delivered =	51.35	Estimated Desired nmol =	397.1
uCi/uL in Dose Sol on Day Delivered =	0.7391	**Date of Delivery =**	**04/02/08**
mg delivered =	0.040	**uL delivered (actual) =**	**55.5**
uCi delivered =	41.00	**Date of Counting =**	**04/02/08**
Estimated nmol delivered =	39.72	**Dose Calculator**	
Days after Synthesis Counted =	23	uL Hot to Deliver =	53.48
Decay on Day Counted =	0.7667	uL Cold to Deliver =	2.00
Specific Activity from Specs (cpm/fmol) =	1.903	Total uL to Deliver =	55.47
Avg. Volume of Standard (uL)* =	3.0	Hot to Cold Ratio =	26.75
Avg. CPM-bkgd of Standard =	2,980,316	Total uL to Make =	69.47
Specific Activity from Standards: (CPM/fmol) =	1.387	Dose Solution uL Hot =	66.97
Date of Disposal from Decay Storage =	10/31/09	Dose Solution uL Cold =	2.50
Average Background=	29.20	Efficiency of Counter=	83.04

*assume 3 uL

Appendix B – 0.004 mg Intranasal Oxytocin Data

Tables

Table B01: Intranasal Oxytocin Rat 44 Data
Table B02: Intranasal Oxytocin Rat 45 Data
Table B03: Intranasal Oxytocin Rat 48 Data
Table B04: Intranasal Oxytocin Rat 49 Data
Table B05: Intranasal Oxytocin Rat 52 Data
Table B06: Intranasal Oxytocin Rat 53 Data
Table B07: Intranasal Oxytocin Rat 56 Data
Table B08: Intranasal Oxytocin Rat 57 Data
Table B09: Intranasal Oxytocin Rat 60 Data
Table B10: Intranasal Oxytocin Rat 61 Data

Table B01: 0.004 mg Intranasal Oxytocin Rat 44 Data

Trigemina Rat 44	Route	Com-pound	Volume (uL)	uCi	mg	Time (min)	Perfused @ (min)
Amount Delivered:	IN	Oxytocin	45	41.00	0.004	14:02	31:05

Tissue Description	Tube #	Tube (g)	Tissue + Tube (g)	Tissue Weight (g)	CPM	nM (Spec. Act.)	nM (Stan.)
Dorsal Dura	1	2.98414	3.00256	0.01842	1859	5.30	7.71
Ventral Dura	2	2.98925	2.99973	0.01048	224	1.12	1.63
Trigeminal Ganglion	3	2.98891	3.01176	0.02285	523	1.20	1.75
Maxillary Nerve	4	2.98494	2.99580	0.01086	152	0.74	1.07
Opthalmic Nerve	5	2.97425	2.97723	0.00298	62	1.09	1.59
Mandibular Nerve	6	3.02673	3.03093	0.00420	154	1.93	2.80
Upper Cervical Spinal Cord	7	2.95224	2.99925	0.04701	404	0.45	0.66
Olfactory Bulbs	8	2.97822	3.04949	0.07127	529	0.39	0.57
Anterior Olfactory Nucleus	9	2.98274	3.02493	0.04219	151	0.19	0.27
Frontal Cortex	10	2.95940	3.00130	0.04190	163	0.20	0.30
Caudate/Putamen	11	3.00394	3.01621	0.01227	56	0.24	0.35
Septal Nucleus	12	2.97741	2.98842	0.01101	42	0.20	0.29
Parietal Cortex	13	2.97583	3.02578	0.04995	190	0.20	0.29
Hippocampus	14	2.98541	3.01572	0.03031	75	0.13	0.19
Thalamus	15	3.04342	3.11320	0.06978	206	0.16	0.23
Hypothalamus	16	2.98770	3.01631	0.02861	166	0.30	0.44
Midbrain	17	2.97922	3.08694	0.10772	541	0.26	0.38
Pons	18	3.03599	3.10368	0.06769	660	0.51	0.75
Medulla	19	2.95832	3.07013	0.11181	1152	0.54	0.79
Cerebellum	20	3.00124	3.25911	0.25787	1237	0.25	0.37
Superficial Nodes (2)	21	2.97866	3.04182	0.06316	8653	7.20	10.47
Cervical Nodes (2)	22	2.96180	2.97281	0.01101	790	3.77	5.48
Thyroid	23	2.97468	2.99595	0.02127	4520	11.16	16.23
Olfactory Epithelium	24	2.99324	3.10790	0.11466	121299	55.56	80.81
Respiratory Epithelium	25	2.98134	3.03066	0.04932	1734902	1,847.38	2,687.13
Axillary Nodes (2)	26	2.97049	3.00006	0.02957	125	0.22	0.32
Muscle (R, deltoid)	27	2.96775	2.99898	0.03123	110	0.18	0.27
Liver (R, superficial lobe)	28	2.96691	2.99433	0.02742	176	0.34	0.49
Kidney (L, tip)	29	2.96703	2.99087	0.02384	180	0.40	0.58
Lung (R, top lobe)	30	2.96479	2.98672	0.02193	452	1.08	1.58
Trachea (near cut-off)	31	2.94715	2.96432	0.01717	117	0.36	0.52
Esophagus (near cut-off)	32	2.96802	2.98031	0.01229	157	0.67	0.98
Spleen (tip)	33	2.95165	2.98265	0.03100	262	0.44	0.64
Heart	34	2.97633	2.99712	0.02079	800	2.02	2.94
Urine (fill to line of tube)	35	3.00183	3.15122	0.14939	2870	1.01	1.47
Spinal Dura	36	2.94560	2.95130	0.00570	92	0.84	1.23
Lower Cervical Spinal Cord	37	2.94408	3.07239	0.12831	310	0.13	0.18
Thoracic Spinal Cord	38	2.97814	3.06944	0.09130	162	0.09	0.14
Lumbar Spinal Cord	39	2.96796	3.08834	0.12038	245	0.11	0.16
Blood Sample	40	2.98465	3.31318	0.32853	8129	1.30	1.89
Drug Standard	41	2.96490	2.96748	0.00258	3430124		
Drug Standard	42	2.98813	2.99060	0.00247	3540001		
Drug Standard	43	3.00200	3.00457	0.00257	3370583		
						19.041 cpm/fmol	13.091 cpm/fmol

Auto Calculations		Enter Values	
Total uL of Dose Sol =	59.2	Oxytocin Molecular Weight	1,007
Hot mg =	0.0000	Date of Synthesis =	03/10/08
Cold mg =	0.0052	Hot uCi/uL at Synthesis =	**1.000**
Total mg in Dose Sol =	0.0052	Hot uCi/ug at Synthesis =	**2184.30**
Conc Of Dose Sol (ug/uL) =	0.0884	Cold ug/uL =	5.00
Days after Synthesis Delivered =	7	Desired uCi =	41.0
Decay on Day Delivered =	0.9223	Desired mg =	0.004
Total uCi in Dose Sol on Day Delivered =	53.69	Estimated Desired nmol =	4.0
uCi/uL in Dose Sol on Day Delivered =	0.9061	**Date of Delivery =**	**03/17/08**
mg delivered =	0.004	**uL delivered (actual) =**	**45.25**
uCi delivered =	41.00	**Date of Counting =**	**03/17/08**
Estimated nmol delivered =	3.97	**Dose Calculator**	
Days after Synthesis Counted =	7	uL Hot to Deliver =	44.45
Decay on Day Counted =	0.9223	uL Cold to Deliver =	0.80
Specific Activity from Specs (cpm/fmol) =	19.041	Total uL to Deliver =	45.25
Avg. Volume of Standard (uL)* =	3.0	Hot to Cold Ratio =	55.85
Avg. CPM-bkgd of Standard =	3,446,903	Total uL to Make =	59.25
Specific Activity from Standards: (CPM/fmol) =	13.091	Dose Solution uL Hot =	58.21
Date of Disposal from Decay Storage =	10/31/09	Dose Solution uL Cold =	1.04
Average Background=	25.00	Efficiency of Counter=	83.08

*assume 3 uL

Table B02: 0.004 mg Intranasal Oxytocin Rat 45 Data

**Trigemina Rat 45**	Route	Com-pound	Volume (uL)	uCi	mg	Time (min)	Perfused @ (min)
Amount Delivered:	IN	Oxytocin	45	41.00	0.004	14:00	31:00

Tissue Description	Tube #	Tube (g)	Tissue + Tube (g)	Tissue Weight (g)	CPM	nM (Spec. Act.)	nM (Stan.)
Dorsal Dura	1	3.03604	3.04928	0.01324	1102	4.37	6.36
Ventral Dura	2	2.99912	3.00621	0.00709	157	1.16	1.69
Trigeminal Ganglion	3	2.95925	2.98360	0.02435	460	0.99	1.44
Maxillary Nerve	4	2.95927	2.97646	0.01719	727	2.22	3.23
Opthalmic Nerve	5	2.97412	2.97716	0.00304	157	2.72	3.96
Mandibular Nerve	6	2.98688	2.99045	0.00357	102	1.50	2.19
Upper Cervical Spinal Cord	7	2.98645	3.02411	0.03766	94	0.13	0.19
Olfactory Bulbs	8	2.97426	3.04741	0.07315	636	0.46	0.66
Anterior Olfactory Nucleus	9	2.98121	3.02102	0.03981	293	0.39	0.56
Frontal Cortex	10	2.96916	3.01340	0.04424	184	0.22	0.32
Caudate/Putamen	11	2.98701	2.99897	0.01196	28	0.12	0.18
Septal Nucleus	12	2.95779	2.96837	0.01058	29	0.14	0.21
Parietal Cortex	13	2.99155	3.03637	0.04482	142	0.17	0.24
Hippocampus	14	2.96058	3.00087	0.04029	86	0.11	0.16
Thalamus	15	2.98499	3.06697	0.08198	164	0.11	0.15
Hypothalamus	16	3.00477	3.03185	0.02708	79	0.15	0.22
Midbrain	17	2.98305	3.08768	0.10463	260	0.13	0.19
Pons	18	2.99736	3.06590	0.06854	166	0.13	0.19
Medulla	19	2.98082	3.07752	0.09670	266	0.14	0.21
Cerebellum	20	2.97122	3.23126	0.26004	663	0.13	0.19
Superficial Nodes (2)	21	2.97586	3.04111	0.06525	5708	4.59	6.68
Cervical Nodes (2)	22	2.97333	2.98796	0.01463	570	2.04	2.97
Thyroid	23	2.97849	3.00334	0.02485	4125	8.72	12.68
Olfactory Epithelium	24	2.97791	3.07827	0.10036	305640	159.94	232.64
Respiratory Epithelium	25	2.97686	3.02862	0.05176	8241971	8,362.61	12,163.93
Axillary Nodes (2)	26	2.98301	3.00965	0.02664	130	0.26	0.37
Muscle (R, deltoid)	27	2.97291	3.00320	0.03029	107	0.19	0.27
Liver (R, superficial lobe)	28	2.96272	2.99233	0.02961	125	0.22	0.32
Kidney (L, tip)	29	2.96579	3.00845	0.04266	316	0.39	0.57
Lung (R, top lobe)	30	2.98229	3.00153	0.01924	101	0.28	0.40
Trachea (near cut-off)	31	2.98340	3.00265	0.01925	175	0.48	0.69
Esophagus (near cut-off)	32	2.97259	2.98762	0.01503	180	0.63	0.91
Spleen (tip)	33	2.94896	2.98555	0.03659	226	0.32	0.47
Heart	34	2.96921	2.99880	0.02959	101	0.18	0.26
Urine (fill to line of tube)	35	2.96316	3.08997	0.12681	1162	0.48	0.70
Spinal Dura	36	2.96848	2.97428	0.00580	43	0.39	0.56
Lower Cervical Spinal Cord	37	2.97932	3.11443	0.13511	213	0.08	0.12
Thoracic Spinal Cord	38	2.95062	3.04611	0.09549	148	0.08	0.12
Lumbar Spinal Cord	39	2.98554	3.11382	0.12828	188	0.08	0.11
Blood Sample	40	3.00265	3.22093	0.21828	5023	1.21	1.76
Drug Standard	41	2.96490	2.96748	0.00258	3430124		
Drug Standard	42	2.98813	2.99060	0.00247	3540001		
Drug Standard	43	3.00200	3.00457	0.00257	3370583		
						19.041 cpm/fmol	13.091 cpm/fmol

**Auto Calculations**		**Enter Values**	
Total uL of Dose Sol =	59.2	Oxytocin Molecular Weight	1,007
Hot mg =	0.0000	Date of Synthesis =	03/10/08
Cold mg =	0.0052	Hot uCi/uL at Synthesis =	**1.000**
Total mg in Dose Sol =	0.0052	Hot uCi/ug at Synthesis =	**2184.30**
Conc Of Dose Sol (ug/uL) =	0.0884	Cold ug/uL =	5.00
Days after Synthesis Delivered =	7	Desired uCi =	41.0
Decay on Day Delivered =	0.9223	Desired mg =	0.004
Total uCi in Dose Sol on Day Delivered =	53.69	Estimated Desired nmol =	4.0
uCi/uL in Dose Sol on Day Delivered =	0.9061	**Date of Delivery =**	**03/17/08**
mg delivered =	0.004	**uL delivered (actual) =**	**45.25**
uCi delivered =	41.00	**Date of Counting =**	**03/17/08**
Estimated nmol delivered =	3.97	**Dose Calculator**	
Days after Synthesis Counted =	7	uL Hot to Deliver =	44.45
Decay on Day Counted =	0.9223	uL Cold to Deliver =	0.80
Specific Activity from Specs (cpm/fmol) =	19.041	Total uL to Deliver =	45.25
Avg. Volume of Standard (uL)* =	3.0	Hot to Cold Ratio =	55.85
Avg. CPM-bkgd of Standard =	3,446,903	Total uL to Make =	59.25
Specific Activity from Standards: (CPM/fmol) =	13.091	Dose Solution uL Hot =	58.21
Date of Disposal from Decay Storage =	10/31/09	Dose Solution uL Cold =	1.04
Average Background=	25.00	Efficiency of Counter=	83.08

*assume 3 uL

Table B03: 0.004 mg Intranasal Oxytocin Rat 48 Data

Trigemina Rat 48	Route	Com-pound	Volume (uL)	uCi	mg	Time (min)	Perfused @ (min)
Amount Delivered:	IN	Oxytocin	47	41.00	0.004	13:58	31:02

Tissue Description	Tube #	Tube (g)	Tissue + Tube (g)	Tissue Weight (g)	CPM	nM (Spec. Act.)	nM (Stan.)
Dorsal Dura	1	2.97590	2.98682	0.01092	103	0.50	0.76
Ventral Dura	2	2.99145	3.00518	0.01373	197	0.75	1.14
Trigeminal Ganglion	3	2.98722	3.00970	0.02248	607	1.42	2.15
Maxillary Nerve	4	2.98747	3.00041	0.01294	114	0.46	0.70
Opthalmic Nerve	5	2.98993	2.99451	0.00458	33	0.38	0.58
Mandibular Nerve	6	2.95482	2.95763	0.00281	425	7.96	12.08
Upper Cervical Spinal Cord	7	2.96278	3.00134	0.03856	115	0.16	0.24
Olfactory Bulbs	8	3.00811	3.07331	0.06520	528	0.43	0.65
Anterior Olfactory Nucleus	9	2.98866	3.03674	0.04808	129	0.14	0.21
Frontal Cortex	10	3.00038	3.03738	0.03700	114	0.16	0.24
Caudate/Putamen	11	2.98630	2.99739	0.01109	16	0.07	0.11
Septal Nucleus	12	2.99600	2.99909	0.00309	4	0.06	0.09
Parietal Cortex	13	2.99685	3.05172	0.05487	147	0.14	0.21
Hippocampus	14	3.06065	3.09460	0.03395	64	0.10	0.15
Thalamus	15	2.98203	3.05620	0.07417	123	0.09	0.13
Hypothalamus	16	2.98413	3.01329	0.02916	62	0.11	0.17
Midbrain	17	3.06089	3.16404	0.10315	230	0.12	0.18
Pons	18	2.99393	3.05806	0.06413	177	0.15	0.22
Medulla	19	2.98372	3.09572	0.11200	277	0.13	0.20
Cerebellum	20	3.00533	3.25092	0.24559	474	0.10	0.15
Superficial Nodes (2)	21	2.98543	3.05920	0.07377	3777	2.69	4.09
Cervical Nodes (2)	22	2.98462	2.99178	0.00716	187	1.37	2.08
Thyroid	23	2.99132	3.01572	0.02440	4571	9.86	14.95
Olfactory Epithelium	24	2.99315	3.07361	0.08046	15433	10.09	15.31
Respiratory Epithelium	25	2.98535	3.04918	0.06383	2773508	2,286.37	3,467.79
Axillary Nodes (2)	26	2.98877	3.00394	0.01517	73	0.25	0.38
Muscle (R, deltoid)	27	2.97676	2.99224	0.01548	49	0.17	0.25
Liver (R, superficial lobe)	28	3.01972	3.05078	0.03106	118	0.20	0.30
Kidney (L, tip)	29	2.98417	3.01962	0.03545	105	0.16	0.24
Lung (R, top lobe)	30	2.97634	2.98796	0.01162	59	0.27	0.40
Trachea (near cut-off)	31	2.98581	3.00273	0.01692	78	0.24	0.37
Esophagus (near cut-off)	32	2.95300	2.97087	0.01787	1357	3.99	6.06
Spleen (tip)	33	2.94784	2.97488	0.02704	79	0.15	0.23
Heart	34	2.96851	2.99843	0.02992	85	0.15	0.23
Urine (fill to line of tube)	35	2.98666	3.22285	0.23619	1441	0.32	0.49
Spinal Dura	36	2.97546	2.98716	0.01170	87	0.39	0.59
Lower Cervical Spinal Cord	37	2.98804	3.13026	0.14222	155	0.06	0.09
Thoracic Spinal Cord	38	2.97859	3.08360	0.10501	111	0.06	0.08
Lumbar Spinal Cord	39	2.95079	3.08034	0.12955	198	0.08	0.12
Blood Sample	40	2.97054	3.26676	0.29622	5377	0.96	1.45
Drug Standard	41	2.98211	2.98530	0.00319	3241197		
Drug Standard	42	2.97255	2.97532	0.00277	3145247		
Drug Standard	43	2.97440	2.97701	0.00261	3180032		
						19.005 cpm/fmol	12.530 cpm/fmol

Auto Calculations		Enter Values	
Total uL of Dose Sol =	60.8	Oxytocin Molecular Weight	1,007
Hot mg =	0.0000	Date of Synthesis =	03/10/08
Cold mg =	0.0052	Hot uCi/uL at Synthesis =	**1.000**
Total mg in Dose Sol =	0.0052	Hot uCi/ug at Synthesis =	**2184.30**
Conc Of Dose Sol (ug/uL) =	0.0854	Cold ug/uL =	5.00
Days after Synthesis Delivered =	10	Desired uCi =	41.0
Decay on Day Delivered =	0.8909	Desired mg =	0.004
Total uCi in Dose Sol on Day Delivered =	53.26	Estimated Desired nmol =	4.0
uCi/uL in Dose Sol on Day Delivered =	0.8758	**Date of Delivery =**	**03/20/08**
mg delivered =	0.004	**uL delivered (actual) =**	**46.82**
uCi delivered =	41.00	**Date of Counting =**	**03/20/08**
Estimated nmol delivered =	3.97	**Dose Calculator**	
Days after Synthesis Counted =	10	uL Hot to Deliver =	46.02
Decay on Day Counted =	0.8909	uL Cold to Deliver =	0.80
Specific Activity from Specs (cpm/fmol) =	19.005	Total uL to Deliver =	46.82
Avg. Volume of Standard (uL)* =	3.0	Hot to Cold Ratio =	57.83
Avg. CPM-bkgd of Standard =	3,188,825	Total uL to Make =	60.82
Specific Activity from Standards: (CPM/fmol) =	12.530	Dose Solution uL Hot =	59.78
Date of Disposal from Decay Storage =	10/31/09	Dose Solution uL Cold =	1.03
Average Background=	25.00	Efficiency of Counter=	82.92

*assume 3 uL

Table B04: 0.004 mg Intranasal Oxytocin Rat 49 Data

Trigemina Rat 49	Route	Com-pound	Volume (uL)	uCi	mg	Time (min)	Perfused @ (min)
Amount Delivered:	IN	Oxytocin	47	41.00	0.004	14:06	30:35

Tissue Description	Tube #	Tube (g)	Tissue + Tube (g)	Tissue Weight (g)	CPM	nM (Spec. Act.)	nM (Stan.)
Dorsal Dura	1	2.98911	3.00515	0.01604	588	1.93	2.93
Ventral Dura	2	3.00608	3.01794	0.01186	224	0.99	1.51
Trigeminal Ganglion	3	2.97585	3.00089	0.02504	436	0.92	1.39
Maxillary Nerve	4	2.98694	3.00157	0.01463	769	2.76	4.19
Opthalmic Nerve	5	2.95829	2.96230	0.00401	102	1.33	2.02
Mandibular Nerve	6	3.02816	3.03075	0.00259	41	0.82	1.25
Upper Cervical Spinal Cord	7	2.95549	2.99095	0.03546	121	0.18	0.27
Olfactory Bulbs	8	2.98457	3.05718	0.07261	405	0.29	0.45
Anterior Olfactory Nucleus	9	2.98536	3.03009	0.04473	109	0.13	0.19
Frontal Cortex	10	2.98740	3.03023	0.04283	108	0.13	0.20
Caudate/Putamen	11	2.98610	2.99791	0.01181	22	0.10	0.15
Septal Nucleus	12	2.97598	2.99197	0.01599	29	0.10	0.14
Parietal Cortex	13	2.95987	3.01012	0.05025	117	0.12	0.19
Hippocampus	14	2.97800	3.01141	0.03341	50	0.08	0.12
Thalamus	15	2.99678	3.06457	0.06779	118	0.09	0.14
Hypothalamus	16	2.95924	2.99331	0.03407	85	0.13	0.20
Midbrain	17	2.98373	3.08353	0.09980	191	0.10	0.15
Pons	18	2.99060	3.06245	0.07185	164	0.12	0.18
Medulla	19	2.96564	3.06645	0.10081	247	0.13	0.20
Cerebellum	20	2.99302	3.24258	0.24956	530	0.11	0.17
Superficial Nodes (2)	21	2.99466	3.09319	0.09853	7839	4.19	6.35
Cervical Nodes (2)	22	2.98745	2.99949	0.01204	485	2.12	3.21
Thyroid	23	2.97372	2.99918	0.02546	3785	7.82	11.87
Olfactory Epithelium	24	2.98396	3.07670	0.09274	13676	7.76	11.77
Respiratory Epithelium	25	3.07142	3.14207	0.07065	1910911	1,423.21	2,158.62
Axillary Nodes (2)	26	2.96590	2.97844	0.01254	55	0.23	0.35
Muscle (R, deltoid)	27	2.97860	3.00426	0.02566	104	0.21	0.32
Liver (R, superficial lobe)	28	2.96459	2.99333	0.02874	72	0.13	0.20
Kidney (L, tip)	29	2.97033	3.02784	0.05751	436	0.40	0.60
Lung (R, top lobe)	30	2.96859	2.98783	0.01924	71	0.19	0.30
Trachea (near cut-off)	31	2.97382	2.99873	0.02491	127	0.27	0.41
Esophagus (near cut-off)	32	2.94802	2.96536	0.01734	88	0.27	0.40
Spleen (tip)	33	2.98469	3.00785	0.02316	97	0.22	0.34
Heart	34	2.96468	3.01621	0.05153	122	0.12	0.19
Urine (fill to line of tube)	35	2.95915	3.20350	0.24435	2966	0.64	0.97
Spinal Dura	36	2.97503	2.98040	0.00537	30	0.29	0.44
Lower Cervical Spinal Cord	37	2.98077	3.11365	0.13288	175	0.07	0.11
Thoracic Spinal Cord	38	3.03041	3.13968	0.10927	147	0.07	0.11
Lumbar Spinal Cord	39	2.98257	3.12216	0.13959	161	0.06	0.09
Blood Sample	40	2.98591	3.31631	0.33040	6085	0.97	1.47
Drug Standard	41	2.98211	2.98530	0.00319	3241197		
Drug Standard	42	2.97255	2.97532	0.00277	3145247		
Drug Standard	43	2.97440	2.97701	0.00261	3180032		
Circle of Willis/Basilar Artery	44	x	x	x	x	x	x
Renal Artery	45	x	x	x	x	x	x
Blank	46				0		
Blank	47				0		
						19.005 cpm/fmol	12.530 cpm/fmol

Auto Calculations		Enter Values	
Total uL of Dose Sol =	60.8	Oxytocin Molecular Weight	1,007
Hot mg =	0.0000	Date of Synthesis =	03/10/08
Cold mg =	0.0052	Hot uCi/uL at Synthesis =	**1.000**
Total mg in Dose Sol =	0.0052	Hot uCi/ug at Synthesis =	**2184.30**
Conc Of Dose Sol (ug/uL) =	0.0854	Cold ug/uL =	5.00
Days after Synthesis Delivered =	10	Desired uCi =	41.0
Decay on Day Delivered =	0.8909	Desired mg =	0.004
Total uCi in Dose Sol on Day Delivered =	53.26	Estimated Desired nmol =	4.0
uCi/uL in Dose Sol on Day Delivered =	0.8758	**Date of Delivery =**	**03/20/08**
mg delivered =	0.004	**uL delivered (actual) =**	**46.8**
uCi delivered =	41.00	**Date of Counting =**	**03/20/08**
Estimated nmol delivered =	3.97	**Dose Calculator**	
Days after Synthesis Counted =	10	uL Hot to Deliver =	46.02
Decay on Day Counted =	0.8909	uL Cold to Deliver =	0.80
Specific Activity from Specs (cpm/fmol) =	19.005	Total uL to Deliver =	46.82
Avg. Volume of Standard (uL)* =	3.0	Hot to Cold Ratio =	57.83
Avg. CPM-bkgd of Standard =	3,188,825	Total uL to Make =	60.82
Specific Activity from Standards: (CPM/fmol) =	12.530	Dose Solution uL Hot =	59.78
Date of Disposal from Decay Storage =	10/31/09	Dose Solution uL Cold =	1.03
Average Background=	25.00	Efficiency of Counter=	82.92

*assume 3 uL

Table B05: 0.004 mg Intranasal Oxytocin Rat 52 Data

Trigemina Rat 52	Route	Com-pound	Volume (uL)	uCi	mg	Time (min)	Perfused @ (min)
Amount Delivered:	IN	Oxytocin	51	41.00	0.004	14:02	31:04

Tissue Description	Tube #	Tube (g)	Tissue + Tube (g)	Tissue Weight (g)	CPM	nM (Spec. Act.)	nM (Stan.)
Dorsal Dura	1	2.97283	2.98861	0.01578	633	2.11	3.13
Ventral Dura	2	3.00272	3.01463	0.01191	730	3.22	4.78
Trigeminal Ganglion	3	3.03322	3.05758	0.02436	567	1.22	1.82
Maxillary Nerve	4	2.99020	3.00473	0.01453	571	2.07	3.07
Opthalmic Nerve	5	2.99781	3.00184	0.00403	402	5.24	7.77
Mandibular Nerve	6	2.95777	2.96174	0.00397	175	2.31	3.43
Upper Cervical Spinal Cord	7	2.97755	3.03112	0.05357	177	0.17	0.26
Olfactory Bulbs	8	2.98949	3.05963	0.07014	570	0.43	0.63
Anterior Olfactory Nucleus	9	2.99435	3.03649	0.04214	168	0.21	0.31
Frontal Cortex	10	2.96148	3.00446	0.04298	191	0.23	0.35
Caudate/Putamen	11	2.99717	3.00921	0.01204	41	0.18	0.27
Septal Nucleus	12	2.98456	2.99961	0.01505	44	0.15	0.23
Parietal Cortex	13	3.06426	3.10583	0.04157	158	0.20	0.30
Hippocampus	14	2.98073	3.02026	0.03953	115	0.15	0.23
Thalamus	15	2.99167	3.07197	0.08030	222	0.15	0.22
Hypothalamus	16	2.95636	2.98823	0.03187	91	0.15	0.22
Midbrain	17	2.96073	3.05450	0.09377	334	0.19	0.28
Pons	18	2.99576	3.07321	0.07745	257	0.17	0.26
Medulla	19	2.98761	3.09521	0.10760	468	0.23	0.34
Cerebellum	20	2.97384	3.23587	0.26203	1140	0.23	0.34
Superficial Nodes (2)	21	2.97851	3.04561	0.06710	3901	3.06	4.53
Cervical Nodes (2)	22	2.95705	2.96596	0.00891	132	0.78	1.16
Thyroid	23	3.00788	3.02905	0.02117	6519	16.19	24.01
Olfactory Epithelium	24	2.99637	3.08368	0.08731	309940	186.68	276.84
Respiratory Epithelium	25	2.98835	3.08741	0.09906	4444910	2,359.69	3,499.31
Axillary Nodes (2)	26	2.97381	2.99963	0.02582	142	0.29	0.43
Muscle (R, deltoid)	27	2.96837	2.98437	0.01600	72	0.24	0.35
Liver (R, superficial lobe)	28	2.99184	3.02729	0.03545	101	0.15	0.22
Kidney (L, tip)	29	2.98588	3.01878	0.03290	137	0.22	0.32
Lung (R, top lobe)	30	2.98006	3.00188	0.02182	156	0.38	0.56
Trachea (near cut-off)	31	2.97574	2.99804	0.02230	299	0.70	1.05
Esophagus (near cut-off)	32	2.97524	2.99135	0.01611	189	0.62	0.91
Spleen (tip)	33	2.96518	2.99342	0.02824	174	0.32	0.48
Heart	34	2.98308	3.01344	0.03036	112	0.19	0.29
Urine (fill to line of tube)	35	2.97853	3.24084	0.26231	2225	0.45	0.66
Spinal Dura	36	3.05732	3.07314	0.01582	99	0.33	0.49
Lower Cervical Spinal Cord	37	2.95901	3.08756	0.12855	260	0.11	0.16
Thoracic Spinal Cord	38	2.97911	3.07116	0.09205	151	0.09	0.13
Lumbar Spinal Cord	39	2.94528	3.07719	0.13191	242	0.10	0.14
Blood Sample	40	3.03530	3.35299	0.31769	10087	1.67	2.48
Drug Standard	41	2.97652	2.97834	0.00182	2921650		
Drug Standard	42	2.95744	2.95965	0.00221	3010902		
Drug Standard	43	2.99509	2.99803	0.00294	3109091		
Circle of Willis/Basilar Artery	44	0.01080	0.01585	0.00505	70	0.73	1.09
Renal Artery	45	2.97825	2.98632	0.00807	37	0.24	0.36
Blank	46				0		
Blank	47				0		
						19.016 cpm/fmol	12.823 cpm/fmol

Auto Calculations		Enter Values	
Total uL of Dose Sol =	64.7	Oxytocin Molecular Weight	1,007
Hot mg =	0.0000	Date of Synthesis =	03/10/08
Cold mg =	0.0051	Hot uCi/uL at Synthesis =	**1.000**
Total mg in Dose Sol =	0.0051	Hot uCi/ug at Synthesis =	**2184.30**
Conc Of Dose Sol (ug/uL) =	0.0789	Cold ug/uL =	5.00
Days after Synthesis Delivered =	17	Desired uCi =	41.0
Decay on Day Delivered =	0.8217	Desired mg =	0.004
Total uCi in Dose Sol on Day Delivered =	52.32	Estimated Desired nmol =	4.0
uCi/uL in Dose Sol on Day Delivered =	0.8088	**Date of Delivery =**	**03/27/08**
mg delivered =	0.004	**uL delivered (actual) =**	**50.69**
uCi delivered =	41.00	**Date of Counting =**	**03/27/08**
Estimated nmol delivered =	3.97	**Dose Calculator**	
Days after Synthesis Counted =	17	uL Hot to Deliver =	49.90
Decay on Day Counted =	0.8217	uL Cold to Deliver =	0.80
Specific Activity from Specs (cpm/fmol) =	19.016	Total uL to Deliver =	50.69
Avg. Volume of Standard (uL)* =	3.0	Hot to Cold Ratio =	62.73
Avg. CPM-bkgd of Standard =	3,013,881	Total uL to Make =	64.69
Specific Activity from Standards: (CPM/fmol) =	12.823	Dose Solution uL Hot =	63.68
Date of Disposal from Decay Storage =	10/31/09	Dose Solution uL Cold =	1.02
Average Background=	28.60	Efficiency of Counter=	82.97

*assume 3 uL

Table B06: 0.004 mg Intranasal Oxytocin Rat 53 Data

Trigemina Rat 53	Route	Com-pound	Volume (uL)	uCi	mg	Time (min)	Perfused @ (min)
Amount Delivered:	IN	Oxytocin	51	41.00	0.004	14:05	30:44

Tissue Description	Tube #	Tube (g)	Tissue + Tube (g)	Tissue Weight (g)	CPM	nM (Spec. Act.)	nM (Stan.)
Dorsal Dura	1	2.99520	3.00861	0.01341	740	2.90	4.31
Ventral Dura	2	2.98568	2.99685	0.01117	724	3.41	5.05
Trigeminal Ganglion	3	2.97120	2.99488	0.02368	319	0.71	1.05
Maxillary Nerve	4	2.98280	2.99744	0.01464	217	0.78	1.15
Opthalmic Nerve	5	2.98535	2.99022	0.00487	164	1.77	2.63
Mandibular Nerve	6	2.96441	2.96834	0.00393	78	1.04	1.54
Upper Cervical Spinal Cord	7	2.98901	3.01680	0.02779	238	0.45	0.67
Olfactory Bulbs	8	2.98638	3.08342	0.09704	668	0.36	0.54
Anterior Olfactory Nucleus	9	2.98233	3.01776	0.03543	123	0.18	0.27
Frontal Cortex	10	2.97563	3.02582	0.05019	222	0.23	0.34
Caudate/Putamen	11	2.96831	2.97900	0.01069	30	0.15	0.22
Septal Nucleus	12	3.06952	3.08020	0.01068	24	0.12	0.17
Parietal Cortex	13	3.01080	3.05457	0.04377	126	0.15	0.23
Hippocampus	14	2.98260	3.02051	0.03791	88	0.12	0.18
Thalamus	15	2.98199	3.05458	0.07259	152	0.11	0.16
Hypothalamus	16	3.00783	3.03267	0.02484	62	0.13	0.19
Midbrain	17	2.99078	3.11354	0.12276	325	0.14	0.21
Pons	18	3.00703	3.07980	0.07277	234	0.17	0.25
Medulla	19	2.96792	3.07098	0.10306	359	0.18	0.27
Cerebellum	20	2.99564	3.24284	0.24720	892	0.19	0.28
Superficial Nodes (2)	21	2.98763	3.08794	0.10031	4123	2.16	3.21
Cervical Nodes (2)	22	2.96899	2.98281	0.01382	563	2.14	3.17
Thyroid	23	2.97979	3.00430	0.02451	8435	18.10	26.84
Olfactory Epithelium	24	2.96876	3.03420	0.06544	153160	123.08	182.52
Respiratory Epithelium	25	2.95822	3.05323	0.09501	2132327	1,180.25	1,750.26
Axillary Nodes (2)	26	2.95439	2.98390	0.02951	228	0.41	0.60
Muscle (R, deltoid)	27	2.98275	3.00598	0.02323	189	0.43	0.64
Liver (R, superficial lobe)	28	2.98892	3.00125	0.01233	235	1.00	1.49
Kidney (L, tip)	29	2.98430	3.00272	0.01842	130	0.37	0.55
Lung (R, top lobe)	30	2.98135	2.98928	0.00793	44	0.29	0.43
Trachea (near cut-off)	31	3.00051	3.02224	0.02173	231	0.56	0.83
Esophagus (near cut-off)	32	2.97405	2.99157	0.01752	177	0.53	0.79
Spleen (tip)	33	2.97665	2.99360	0.01695	165	0.51	0.76
Heart	34	2.96030	2.99504	0.03474	180	0.27	0.40
Urine (fill to line of tube)	35	2.96168	3.21590	0.25422	3545	0.73	1.09
Spinal Dura	36	2.99808	3.00904	0.01096	360	1.73	2.56
Lower Cervical Spinal Cord	37	3.04121	3.19307	0.15186	444	0.15	0.23
Thoracic Spinal Cord	38	2.98327	3.09687	0.11360	187	0.09	0.13
Lumbar Spinal Cord	39	3.05464	3.14626	0.09162	169	0.10	0.14
Blood Sample	40	2.98932	3.19607	0.20675	6260	1.59	2.36
Drug Standard	41	2.97652	2.97834	0.00182	2921650		
Drug Standard	42	2.95744	2.95965	0.00221	3010902		
Drug Standard	43	2.99509	2.99803	0.00294	3109091		
Circle of Willis/Basilar Artery	44	0.01103	0.01561	0.00458	76	0.88	1.30
Renal Artery	45	2.96957	2.97294	0.00337	31	0.48	0.72
Blank	46				0		
Blank	47				0		
						19.016 cpm/fmol	12.823 cpm/fmol

Auto Calculations		Enter Values	
Total uL of Dose Sol =	64.7	Oxytocin Molecular Weight	1,007
Hot mg =	0.0000	Date of Synthesis =	03/10/08
Cold mg =	0.0051	Hot uCi/uL at Synthesis =	**1.000**
Total mg in Dose Sol =	0.0051	Hot uCi/ug at Synthesis =	**2184.30**
Conc Of Dose Sol (ug/uL) =	0.0789	Cold ug/uL =	5.00
Days after Synthesis Delivered =	17	Desired uCi =	41.0
Decay on Day Delivered =	0.8217	Desired mg =	0.004
Total uCi in Dose Sol on Day Delivered =	52.32	Estimated Desired nmol =	4.0
uCi/uL in Dose Sol on Day Delivered =	0.8088	**Date of Delivery =**	**03/27/08**
mg delivered =	0.004	**uL delivered (actual) =**	**50.69**
uCi delivered =	41.00	**Date of Counting =**	**03/27/08**
Estimated nmol delivered =	3.97	**Dose Calculator**	
Days after Synthesis Counted =	17	uL Hot to Deliver =	49.90
Decay on Day Counted =	0.8217	uL Cold to Deliver =	0.80
Specific Activity from Specs (cpm/fmol) =	19.016	Total uL to Deliver =	50.69
Avg. Volume of Standard (uL)* =	3.0	Hot to Cold Ratio =	62.73
Avg. CPM-bkgd of Standard =	3,013,881	Total uL to Make =	64.69
Specific Activity from Standards: (CPM/fmol) =	12.823	Dose Solution uL Hot =	63.68
Date of Disposal from Decay Storage =	10/31/09	Dose Solution uL Cold =	1.02
Average Background=	26.24	Efficiency of Counter=	82.97

*assume 3 uL

Table B07: 0.004 mg Intranasal Oxytocin Rat 56 Data

**Trigemina Rat 56**	Route	Com-pound	Volume (uL)	uCi	mg	Time (min)	Perfused @ (min)
Amount Delivered:	IN	Oxytocin	54	41.00	0.004	14:01	30:46

Tissue Description	Tube #	Tube (g)	Tissue + Tube (g)	Tissue Weight (g)	CPM	nM (Spec. Act.)	nM (Stan.)
Dorsal Dura	1	2.98175	2.99366	0.01191	668	2.95	4.40
Ventral Dura	2	2.96257	2.97124	0.00867	712	4.32	6.44
Trigeminal Ganglion	3	2.97989	3.00374	0.02385	299	0.66	0.98
Maxillary Nerve	4	3.00356	3.01500	0.01144	215	0.99	1.47
Opthalmic Nerve	5	2.97995	2.98262	0.00267	71	1.39	2.08
Mandibular Nerve	6	2.96805	2.97178	0.00373	187	2.64	3.94
Upper Cervical Spinal Cord	7	2.96551	3.03448	0.06897	327	0.25	0.37
Olfactory Bulbs	8	2.98216	3.05573	0.07357	767	0.55	0.82
Anterior Olfactory Nucleus	9	2.96982	3.00589	0.03607	210	0.31	0.46
Frontal Cortex	10	2.97500	3.03004	0.05504	377	0.36	0.54
Caudate/Putamen	11	3.00038	3.01146	0.01108	55	0.26	0.39
Septal Nucleus	12	2.97171	2.98548	0.01377	38	0.15	0.22
Parietal Cortex	13	3.00161	3.04272	0.04111	195	0.25	0.37
Hippocampus	14	3.00119	3.04032	0.03913	104	0.14	0.21
Thalamus	15	2.98581	3.05492	0.06911	162	0.12	0.18
Hypothalamus	16	3.01928	3.05436	0.03508	95	0.14	0.21
Midbrain	17	3.00320	3.11236	0.10916	353	0.17	0.25
Pons	18	2.96699	3.04605	0.07906	203	0.13	0.20
Medulla	19	3.00131	3.11695	0.11564	336	0.15	0.23
Cerebellum	20	2.96470	3.23093	0.26623	840	0.17	0.25
Superficial Nodes (2)	21	2.97586	3.04227	0.06641	1066	0.84	1.26
Cervical Nodes (2)	22	2.96574	2.98406	0.01832	1309	3.75	5.60
Thyroid	23	2.96602	2.98443	0.01841	8364	23.87	35.63
Olfactory Epithelium	24	2.95628	3.03319	0.07691	11713	8.00	11.95
Respiratory Epithelium	25	2.97604	3.05274	0.07670	3157624	2,163.52	3,229.23
Axillary Nodes (2)	26	2.95346	2.97177	0.01831	185	0.53	0.79
Muscle (R, deltoid)	27	2.96282	2.97316	0.01034	58	0.29	0.44
Liver (R, superficial lobe)	28	2.96537	2.99635	0.03098	174	0.30	0.44
Kidney (L, tip)	29	2.94323	2.97210	0.02887	235	0.43	0.64
Lung (R, top lobe)	30	2.95329	2.96918	0.01589	130	0.43	0.64
Trachea (near cut-off)	31	3.00549	3.02524	0.01975	179	0.47	0.71
Esophagus (near cut-off)	32	2.99560	3.01235	0.01675	156	0.49	0.73
Spleen (tip)	33	2.94670	2.98046	0.03376	343	0.53	0.80
Heart	34	2.96332	2.99214	0.02882	92	0.17	0.25
Urine (fill to line of tube)	35	2.97299	3.00261	0.02962	1003	1.78	2.66
Spinal Dura	36	2.95670	2.96821	0.01151	82	0.37	0.56
Lower Cervical Spinal Cord	37	2.93994	3.04969	0.10975	189	0.09	0.14
Thoracic Spinal Cord	38	2.96630	3.06451	0.09821	127	0.07	0.10
Lumbar Spinal Cord	39	3.01254	3.14495	0.13241	211	0.08	0.12
Blood Sample	40	3.02033	3.28992	0.26959	9113	1.78	2.65
Drug Standard	41	2.95428	2.95714	0.00286	2849408		
Drug Standard	42	2.95382	2.95678	0.00296	2834480		
Drug Standard	43	2.95761	2.96103	0.00342	2810189		
Circle of Willis/Basilar Artery	44	0.01171	0.01574	0.00403	118	1.54	2.29
Renal Artery	45	2.95370	2.95998	0.00628	50	0.42	0.62
Blank	46				3		
Blank	47				1		
						19.028 cpm/fmol	12.749 cpm/fmol

Auto Calculations		Enter Values	
Total uL of Dose Sol =	67.7	Oxytocin Molecular Weight	1,007
Hot mg =	0.0000	Date of Synthesis =	03/10/08
Cold mg =	0.0050	Hot uCi/uL at Synthesis =	**1.000**
Total mg in Dose Sol =	0.0050	Hot uCi/ug at Synthesis =	**2184.30**
Conc Of Dose Sol (ug/uL) =	0.0745	Cold ug/uL =	5.00
Days after Synthesis Delivered =	22	Desired uCi =	41.0
Decay on Day Delivered =	0.7756	Desired mg =	0.004
Total uCi in Dose Sol on Day Delivered =	51.70	Estimated Desired nmol =	4.0
uCi/uL in Dose Sol on Day Delivered =	0.7641	**Date of Delivery =**	**04/01/08**
mg delivered =	0.004	**uL delivered (actual) =**	**53.7**
uCi delivered =	41.00	**Date of Counting =**	**04/01/08**
Estimated nmol delivered =	3.97	**Dose Calculator**	
Days after Synthesis Counted =	22	uL Hot to Deliver =	52.86
Decay on Day Counted =	0.7756	uL Cold to Deliver =	0.80
Specific Activity from Specs (cpm/fmol) =	19.028	Total uL to Deliver =	53.66
Avg. Volume of Standard (uL)* =	3.0	Hot to Cold Ratio =	66.48
Avg. CPM-bkgd of Standard =	2,831,359	Total uL to Make =	67.66
Specific Activity from Standards: (CPM/fmol) =	12.749	Dose Solution uL Hot =	66.65
Date of Disposal from Decay Storage =	10/31/09	Dose Solution uL Cold =	1.00
Average Background=	29.28	Efficiency of Counter=	83.04

*assume 3 uL

Table B08: 0.004 mg Intranasal Oxytocin Rat 57 Data

Trigemina Rat 57	Route	Com-pound	Volume (uL)	uCi	mg	Time (min)	Perfused @ (min)
Amount Delivered:	IN	Oxytocin	54	41.00	0.004	13:59	31:01

Tissue Description	Tube #	Tube (g)	Tissue + Tube (g)	Tissue Weight (g)	CPM	nM (Spec. Act.)	nM (Stan.)
Dorsal Dura	1	3.00218	3.01633	0.01415	1134	4.21	6.34
Ventral Dura	2	2.98415	2.99490	0.01075	297	1.45	2.19
Trigeminal Ganglion	3	2.99506	3.02138	0.02632	233	0.47	0.70
Maxillary Nerve	4	2.98597	3.00091	0.01494	268	0.94	1.42
Opthalmic Nerve	5	2.96332	2.96751	0.00419	607	7.61	11.45
Mandibular Nerve	6	2.97128	2.97529	0.00401	108	1.41	2.13
Upper Cervical Spinal Cord	7	2.97611	3.01320	0.03709	257	0.36	0.55
Olfactory Bulbs	8	2.98553	3.04546	0.05993	623	0.55	0.82
Anterior Olfactory Nucleus	9	2.98936	3.02002	0.03066	130	0.22	0.33
Frontal Cortex	10	3.00227	3.03779	0.03552	152	0.23	0.34
Caudate/Putamen	11	2.98048	2.99279	0.01231	41	0.17	0.26
Septal Nucleus	12	2.98411	2.99857	0.01446	36	0.13	0.20
Parietal Cortex	13	2.96289	3.00236	0.03947	230	0.31	0.46
Hippocampus	14	3.02996	3.06074	0.03078	89	0.15	0.23
Thalamus	15	2.97257	3.02928	0.05671	147	0.14	0.21
Hypothalamus	16	2.97274	2.99768	0.02494	71	0.15	0.23
Midbrain	17	2.97839	3.11388	0.13549	347	0.13	0.20
Pons	18	3.01404	3.07943	0.06539	262	0.21	0.32
Medulla	19	2.97675	3.08118	0.10443	330	0.17	0.25
Cerebellum	20	2.96726	3.22870	0.26144	807	0.16	0.24
Superficial Nodes (2)	21	2.98894	3.06348	0.07454	4300	3.03	4.56
Cervical Nodes (2)	22	2.96702	2.97547	0.00845	97	0.61	0.91
Thyroid	23	2.96541	2.99074	0.02533	12921	26.81	40.36
Olfactory Epithelium	24	3.02241	3.11150	0.08909	12121	7.15	10.76
Respiratory Epithelium	25	2.96131	3.04921	0.08790	6925466	4,140.53	6,233.58
Axillary Nodes (2)	26	2.95589	2.97826	0.02237	163	0.38	0.58
Muscle (R, deltoid)	27	2.99322	3.01448	0.02126	77	0.19	0.29
Liver (R, superficial lobe)	28	2.95839	2.99098	0.03259	139	0.22	0.34
Kidney (L, tip)	29	2.99264	3.01664	0.02400	233	0.51	0.77
Lung (R, top lobe)	30	2.94835	2.96854	0.02019	314	0.82	1.23
Trachea (near cut-off)	31	2.95961	2.97613	0.01652	176	0.56	0.84
Esophagus (near cut-off)	32	2.99048	3.00186	0.01138	91	0.42	0.63
Spleen (tip)	33	2.97647	2.99890	0.02243	253	0.59	0.89
Heart	34	2.96029	2.99370	0.03341	113	0.18	0.27
Urine (fill to line of tube)	35	2.94686	3.18522	0.23836	5817	1.28	1.93
Spinal Dura	36	2.96649	2.98074	0.01425	125	0.46	0.70
Lower Cervical Spinal Cord	37	2.99054	3.13461	0.14407	308	0.11	0.17
Thoracic Spinal Cord	38	2.95716	3.05892	0.10176	162	0.08	0.13
Lumbar Spinal Cord	39	2.97439	3.10729	0.13290	249	0.10	0.15
Blood Sample	40	2.95056	3.23941	0.28885	12100	2.20	3.31
Drug Standard	41	2.95428	2.95714	0.00286	2806898		
Drug Standard	42	2.95382	2.95678	0.00296	2821020		
Drug Standard	43	2.95761	2.96103	0.00342	2793265		
Circle of Willis/Basilar Artery	44	0.01097	0.01614	0.00517	43	0.44	0.65
Renal Artery	45	2.96847	2.97417	0.00570	57	0.53	0.79
Blank	46				4		
Blank	47				0		
						19.028 cpm/fmol	12.639 cpm/fmol

Auto Calculations		Enter Values	
Total uL of Dose Sol =	67.7	Oxytocin Molecular Weight	1,007
Hot mg =	0.0000	Date of Synthesis =	03/10/08
Cold mg =	0.0050	Hot uCi/uL at Synthesis =	**1.000**
Total mg in Dose Sol =	0.0050	Hot uCi/ug at Synthesis =	**2184.30**
Conc Of Dose Sol (ug/uL) =	0.0745	Cold ug/uL =	5.00
Days after Synthesis Delivered =	22	Desired uCi =	41.0
Decay on Day Delivered =	0.7756	Desired mg =	0.004
Total uCi in Dose Sol on Day Delivered =	51.70	Estimated Desired nmol =	4.0
uCi/uL in Dose Sol on Day Delivered =	0.7641	**Date of Delivery =**	**04/01/08**
mg delivered =	0.004	**uL delivered (actual) =**	**53.7**
uCi delivered =	41.00	**Date of Counting =**	**04/01/08**
Estimated nmol delivered =	3.97	**Dose Calculator**	
Days after Synthesis Counted =	22	uL Hot to Deliver =	52.86
Decay on Day Counted =	0.7756	uL Cold to Deliver =	0.80
Specific Activity from Specs (cpm/fmol) =	19.028	Total uL to Deliver =	53.66
Avg. Volume of Standard (uL)* =	3.0	Hot to Cold Ratio =	66.48
Avg. CPM-bkgd of Standard =	2,807,061	Total uL to Make =	67.66
Specific Activity from Standards: (CPM/fmol) =	12.639	Dose Solution uL Hot =	66.65
Date of Disposal from Decay Storage =	10/31/09	Dose Solution uL Cold =	1.00
Average Background=	27.68	Efficiency of Counter=	83.04

*assume 3 uL

Table B09: 0.004 mg Intranasal Oxytocin Rat 60 Data

Trigemina Rat 60	Route	Com-pound	Volume (uL)	uCi	mg	Time (min)	Perfused @ (min)
Amount Delivered:	IN	Oxytocin	55	41.00	0.004	14:00	30:37

Tissue Description	Tube #	Tube (g)	Tissue + Tube (g)	Tissue Weight (g)	CPM	nM (Spec. Act.)	nM (Stan.)
Dorsal Dura	1	2.99228	3.00653	0.01425	328	1.21	1.83
Ventral Dura	2	2.98288	2.98817	0.00529	147	1.46	2.22
Trigeminal Ganglion	3	2.95639	2.98442	0.02803	231	0.43	0.65
Maxillary Nerve	4	2.97070	2.98327	0.01257	177	0.74	1.12
Opthalmic Nerve	5	2.98086	2.98417	0.00331	87	1.37	2.08
Mandibular Nerve	6	2.97240	2.97689	0.00449	119	1.39	2.10
Upper Cervical Spinal Cord	7	2.98554	3.01161	0.02607	100	0.20	0.30
Olfactory Bulbs	8	2.95058	3.02713	0.07655	688	0.47	0.71
Anterior Olfactory Nucleus	9	2.96318	2.99609	0.03291	136	0.22	0.33
Frontal Cortex	10	2.98206	3.02503	0.04297	147	0.18	0.27
Caudate/Putamen	11	3.00266	3.01481	0.01215	40	0.17	0.26
Septal Nucleus	12	2.96613	2.98740	0.02127	67	0.17	0.25
Parietal Cortex	13	2.98552	3.03780	0.05228	161	0.16	0.25
Hippocampus	14	3.00733	3.05059	0.04326	108	0.13	0.20
Thalamus	15	3.00739	3.09175	0.08436	235	0.15	0.22
Hypothalamus	16	2.96678	2.99114	0.02436	87	0.19	0.28
Midbrain	17	2.97622	3.06450	0.08828	272	0.16	0.25
Pons	18	3.01867	3.08787	0.06920	261	0.20	0.30
Medulla	19	2.98351	3.09023	0.10672	485	0.24	0.36
Cerebellum	20	2.96179	3.22100	0.25921	1089	0.22	0.33
Superficial Nodes (2)	21	2.99719	3.06397	0.06678	1867	1.47	2.22
Cervical Nodes (2)	22	2.97185	2.98755	0.01570	352	1.18	1.78
Thyroid	23	2.96182	2.98774	0.02592	16772	34.00	51.47
Olfactory Epithelium	24	2.98513	3.08862	0.10349	627437	318.62	482.29
Respiratory Epithelium	25	2.97211	3.07359	0.10148	2726655	1,412.04	2,137.42
Axillary Nodes (2)	26	2.96872	2.99273	0.02401	295	0.65	0.98
Muscle (R, deltoid)	27	3.01127	3.03483	0.02356	220	0.49	0.74
Liver (R, superficial lobe)	28	3.00891	3.03126	0.02235	389	0.92	1.39
Kidney (L, tip)	29	2.97651	3.02224	0.04573	390	0.45	0.68
Lung (R, top lobe)	30	2.96729	2.98593	0.01864	185	0.52	0.79
Trachea (near cut-off)	31	2.97421	3.00407	0.02986	376	0.66	1.00
Esophagus (near cut-off)	32	2.99169	3.02011	0.02842	467	0.86	1.31
Spleen (tip)	33	2.96353	2.98240	0.01887	387	1.08	1.63
Heart	34	3.00958	3.03817	0.02859	366	0.67	1.02
Urine (fill to line of tube)	35	2.96717	3.27614	0.30897	5461	0.93	1.41
Spinal Dura	36	2.95176	2.95887	0.00711	231	1.71	2.58
Lower Cervical Spinal Cord	37	2.94723	3.09459	0.14736	422	0.15	0.23
Thoracic Spinal Cord	38	2.95688	3.06722	0.11034	223	0.11	0.16
Lumbar Spinal Cord	39	2.95150	3.05799	0.10649	233	0.12	0.17
Blood Sample	40	2.96696	3.23308	0.26612	11935	2.36	3.57
Drug Standard	41	2.96241	2.96517	0.00276	2718457		
Drug Standard	42	2.94818	2.95065	0.00247	2726004		
Drug Standard	43	2.98948	2.99265	0.00317	2742571		
Circle of Willis/Basilar Artery	44	2.95241	2.95877	0.00636	101	0.83	1.26
Renal Artery	45	2.96999	2.97717	0.00718	248	1.82	2.75
Blank	46				0		
Blank	47				0		
						19.028 cpm/fmol	12.571 cpm/fmol

Auto Calculations		Enter Values	
Total uL of Dose Sol =	68.9	Oxytocin Molecular Weight	1,007
Hot mg =	0.0000	Date of Synthesis =	03/10/08
Cold mg =	0.0050	Hot uCi/uL at Synthesis =	**1.000**
Total mg in Dose Sol =	0.0050	Hot uCi/ug at Synthesis =	**2184.30**
Conc Of Dose Sol (ug/uL) =	0.0729	Cold ug/uL =	5.00
Days after Synthesis Delivered =	24	Desired uCi =	41.0
Decay on Day Delivered =	0.7579	Desired mg =	0.004
Total uCi in Dose Sol on Day Delivered =	51.46	Estimated Desired nmol =	4.0
uCi/uL in Dose Sol on Day Delivered =	0.7469	**Date of Delivery =**	**04/03/08**
mg delivered =	0.004	**uL delivered (actual) =**	**54.9**
uCi delivered =	41.00	**Date of Counting =**	**04/03/08**
Estimated nmol delivered =	3.97	**Dose Calculator**	
Days after Synthesis Counted =	24	uL Hot to Deliver =	54.10
Decay on Day Counted =	0.7579	uL Cold to Deliver =	0.80
Specific Activity from Specs (cpm/fmol) =	19.028	Total uL to Deliver =	54.89
Avg. Volume of Standard (uL)* =	3.0	Hot to Cold Ratio =	68.04
Avg. CPM-bkgd of Standard =	2,729,011	Total uL to Make =	68.89
Specific Activity from Standards: (CPM/fmol) =	12.571	Dose Solution uL Hot =	67.89
Date of Disposal from Decay Storage =	10/31/09	Dose Solution uL Cold =	1.00
Average Background=	27.50	Efficiency of Counter=	83.04

*assume 3 uL

Table B10: 0.004 mg Intranasal Oxytocin Rat 61 Data

Trigemina Rat 61	Route	Com-pound	Volume (uL)	uCi	mg	Time (min)	Perfused @ (min)
Amount Delivered:	IN	Oxytocin	55	41.00	0.004	14:00	30:30

Tissue Description	Tube #	Tube (g)	Tissue + Tube (g)	Tissue Weight (g)	CPM	nM (Spec. Act.)	nM (Stan.)
Dorsal Dura	1	2.96168	2.97305	0.01137	258	1.19	1.81
Ventral Dura	2	2.96514	2.97217	0.00703	215	1.61	2.44
Trigeminal Ganglion	3	2.97727	3.00629	0.02902	226	0.41	0.62
Maxillary Nerve	4	2.98685	2.99894	0.01209	150	0.65	0.99
Opthalmic Nerve	5	2.95942	2.96194	0.00252	64	1.34	2.04
Mandibular Nerve	6	2.96601	2.96935	0.00334	169	2.66	4.04
Upper Cervical Spinal Cord	7	2.98783	3.03119	0.04336	148	0.18	0.27
Olfactory Bulbs	8	2.96814	3.04200	0.07386	1282	0.91	1.39
Anterior Olfactory Nucleus	9	2.97162	3.01233	0.04071	14512	18.73	28.46
Frontal Cortex	10	2.98220	3.01419	0.03199	152	0.25	0.38
Caudate/Putamen	11	2.96207	2.97381	0.01174	56	0.25	0.38
Septal Nucleus	12	2.96519	2.97795	0.01276	26	0.11	0.17
Parietal Cortex	13	2.97176	3.01433	0.04257	163	0.20	0.31
Hippocampus	14	2.95749	2.99750	0.04001	96	0.13	0.19
Thalamus	15	2.96097	3.02665	0.06568	161	0.13	0.20
Hypothalamus	16	2.96429	2.99945	0.03516	97	0.14	0.22
Midbrain	17	2.96535	3.08404	0.11869	325	0.14	0.22
Pons	18	2.97668	3.05545	0.07877	221	0.15	0.22
Medulla	19	2.96570	3.07702	0.11132	351	0.17	0.25
Cerebellum	20	2.97350	3.22093	0.24743	757	0.16	0.24
Superficial Nodes (2)	21	3.01837	3.09608	0.07771	1275	0.86	1.31
Cervical Nodes (2)	22	3.01413	3.02420	0.01007	407	2.13	3.23
Thyroid	23	2.97066	2.98558	0.01492	10063	35.44	53.85
Olfactory Epithelium	24	2.97519	3.04879	0.07360	7568	5.40	8.21
Respiratory Epithelium	25	2.99212	3.08998	0.09786	3551628	1,907.30	2,897.85
Axillary Nodes (2)	26	2.95951	2.98527	0.02576	246	0.50	0.76
Muscle (R, deltoid)	27	2.96367	2.99353	0.02986	146	0.26	0.39
Liver (R, superficial lobe)	28	2.95718	3.00987	0.05269	378	0.38	0.57
Kidney (L, tip)	29	2.95989	2.98479	0.02490	178	0.37	0.57
Lung (R, top lobe)	30	2.96151	2.97661	0.01510	182	0.63	0.96
Trachea (near cut-off)	31	2.99886	3.02261	0.02375	592	1.31	1.99
Esophagus (near cut-off)	32	2.98411	3.00882	0.02471	515	1.10	1.67
Spleen (tip)	33	2.99574	3.02264	0.02690	215	0.42	0.64
Heart	34	2.94832	2.98259	0.03427	125	0.19	0.29
Urine (fill to line of tube)	35	2.97249	3.21791	0.24542	3080	0.66	1.00
Spinal Dura	36	2.95492	2.96529	0.01037	98	0.50	0.76
Lower Cervical Spinal Cord	37	2.99284	3.13240	0.13956	274	0.10	0.16
Thoracic Spinal Cord	38	2.94762	3.06624	0.11862	176	0.08	0.12
Lumbar Spinal Cord	39	2.96390	3.06462	0.10072	197	0.10	0.16
Blood Sample	40	2.96763	3.23207	0.26444	10908	2.17	3.29
Drug Standard	41	2.96241	2.96517	0.00276	2709448		
Drug Standard	42	2.94818	2.95065	0.00247	2715717		
Drug Standard	43	2.98948	2.99265	0.00317	2731494		
Circle of Willis/Basilar Artery	44	2.99924	3.00230	0.00306	33	0.57	0.86
Renal Artery	45	2.96734	2.97405	0.00671	43	0.34	0.51
Blank	46				6		
Blank	47				1		
						19.028 cpm/fmol	12.524 cpm/fmol

Auto Calculations		Enter Values	
Total uL of Dose Sol =	68.9	Oxytocin Molecular Weight	1,007
Hot mg =	0.0000	Date of Synthesis =	03/10/08
Cold mg =	0.0050	Hot uCi/uL at Synthesis =	**1.000**
Total mg in Dose Sol =	0.0050	Hot uCi/ug at Synthesis =	**2184.30**
Conc Of Dose Sol (ug/uL) =	0.0729	Cold ug/uL =	5.00
Days after Synthesis Delivered =	24	Desired uCi =	41.0
Decay on Day Delivered =	0.7579	Desired mg =	0.004
Total uCi in Dose Sol on Day Delivered =	51.46	Estimated Desired nmol =	4.0
uCi/uL in Dose Sol on Day Delivered =	0.7469	**Date of Delivery =**	**04/03/08**
mg delivered =	0.004	**uL delivered (actual) =**	**54.9**
uCi delivered =	41.00	**Date of Counting =**	**04/03/08**
Estimated nmol delivered =	3.97	**Dose Calculator**	
Days after Synthesis Counted =	24	uL Hot to Deliver =	54.10
Decay on Day Counted =	0.7579	uL Cold to Deliver =	0.80
Specific Activity from Specs (cpm/fmol) =	19.028	Total uL to Deliver =	54.89
Avg. Volume of Standard (uL)* =	3.0	Hot to Cold Ratio =	68.04
Avg. CPM-bkgd of Standard =	2,718,887	Total uL to Make =	68.89
Specific Activity from Standards: (CPM/fmol) =	12.524	Dose Solution uL Hot =	67.89
Date of Disposal from Decay Storage =	10/31/09	Dose Solution uL Cold =	1.00
Average Background=	29.30	Efficiency of Counter=	83.04

*assume 3 uL

Appendix C – Coronal Rat Brain Slices

Figures

Figure C01: Brain Slice 01
Figure C02: Brain Slice 02
Figure C03: Brain Slice 03
Figure C04: Brain Slice 04
Figure C05: Brain Slice 05
Figure C06: Brain Slice 06
Figure C07: Lateral Brain Bisection

Figure C01: 1st Slice

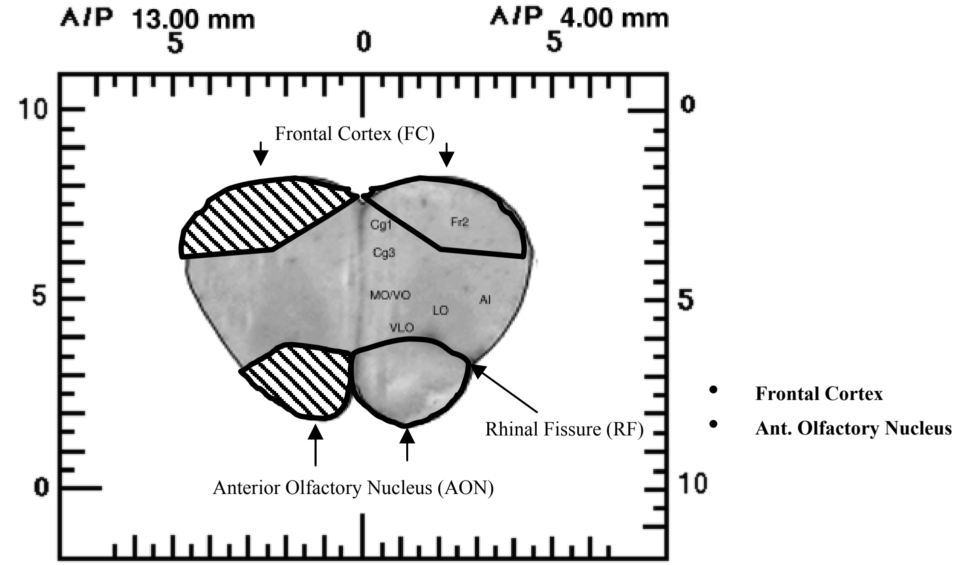

Figure C02: 2nd Slice

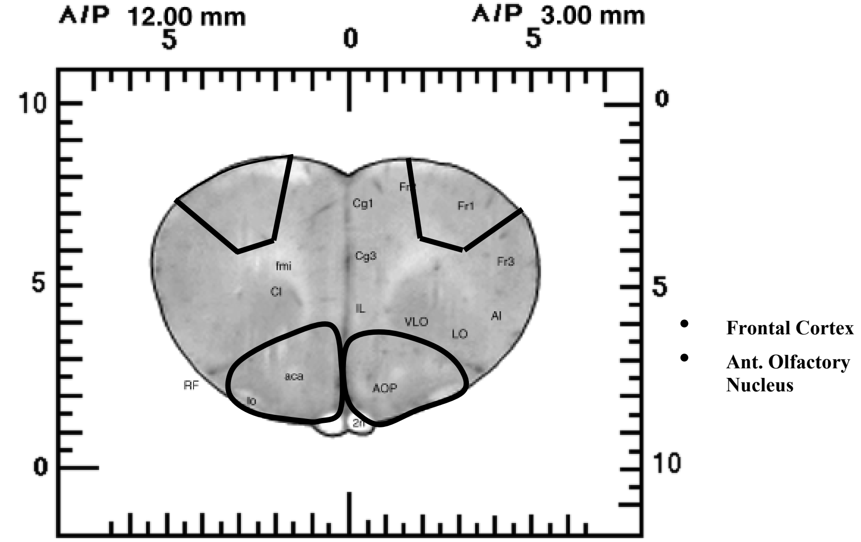

Figure C03: 3rd Slice

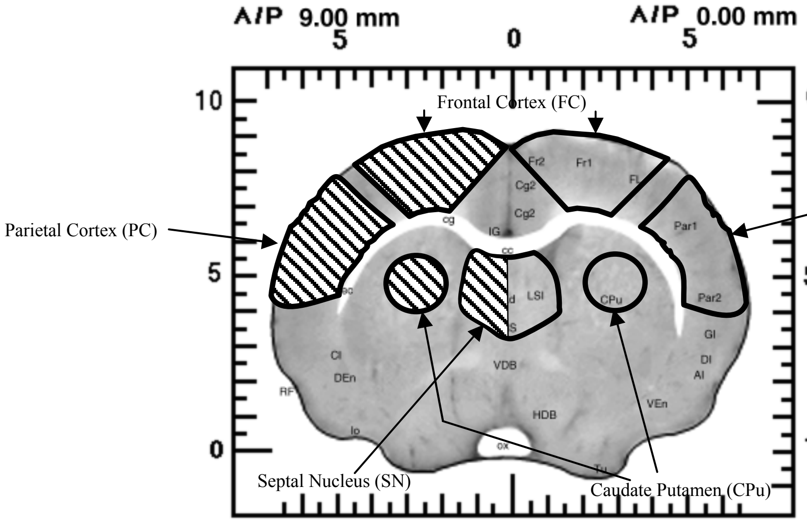

- **Caudate/Putamen**
- **Frontal Cortex**
- **Parietal Cortex**
- **Septal Nucleus**

Figure C04: 4th Slice

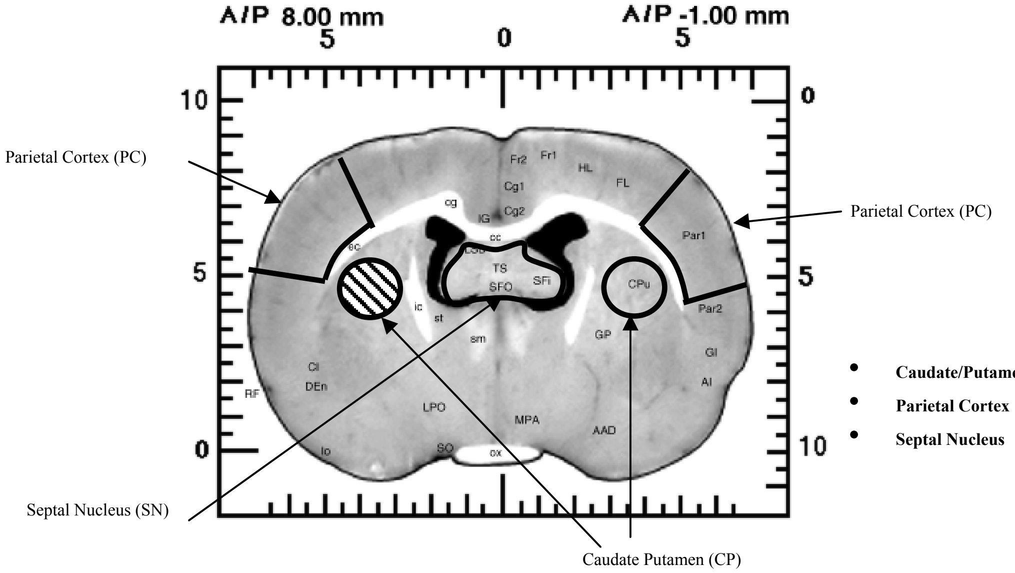

- **Caudate/Putamen**
- **Parietal Cortex**
- **Septal Nucleus**

Figure C05: 5th Slice

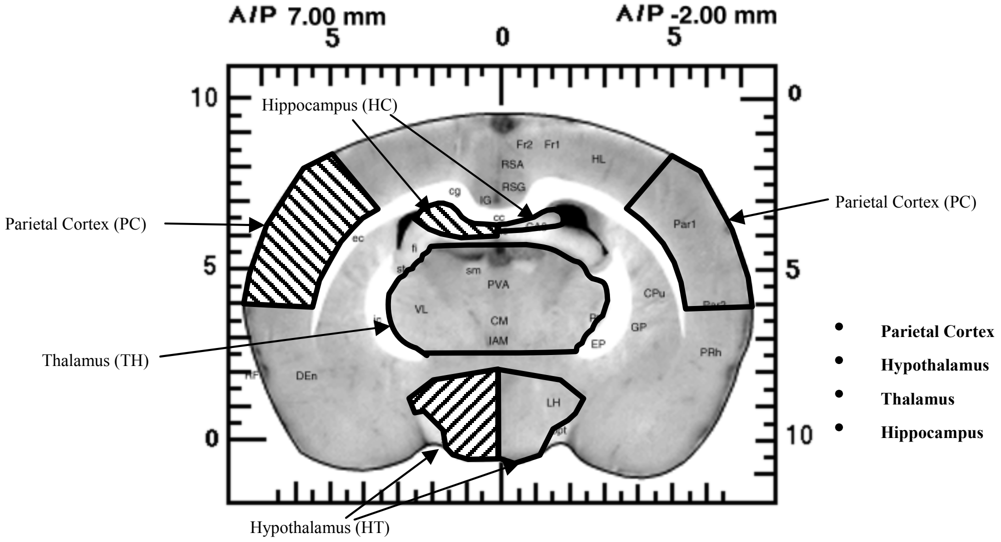

Figure C06: 6th Slice

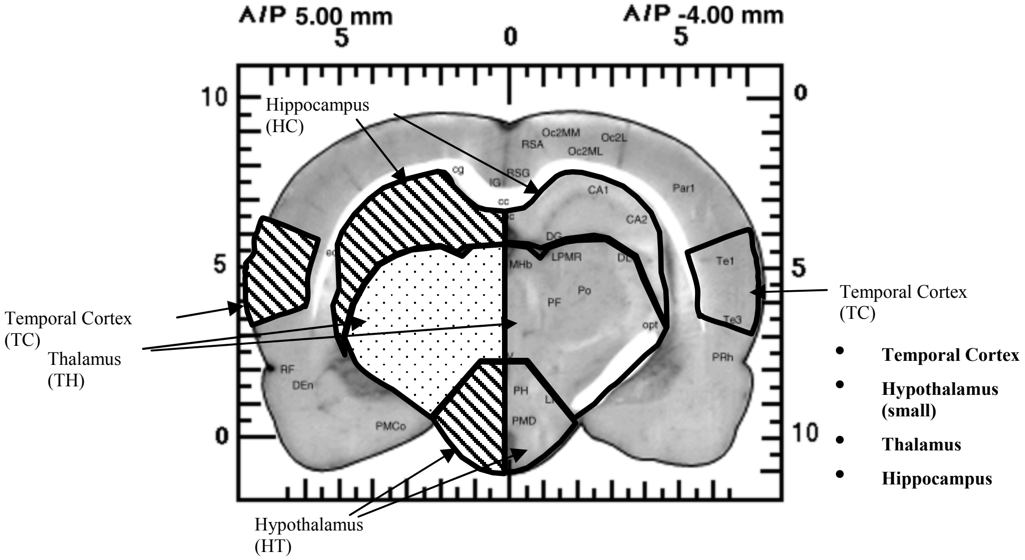

Figure C07: Rat Brain Bisection

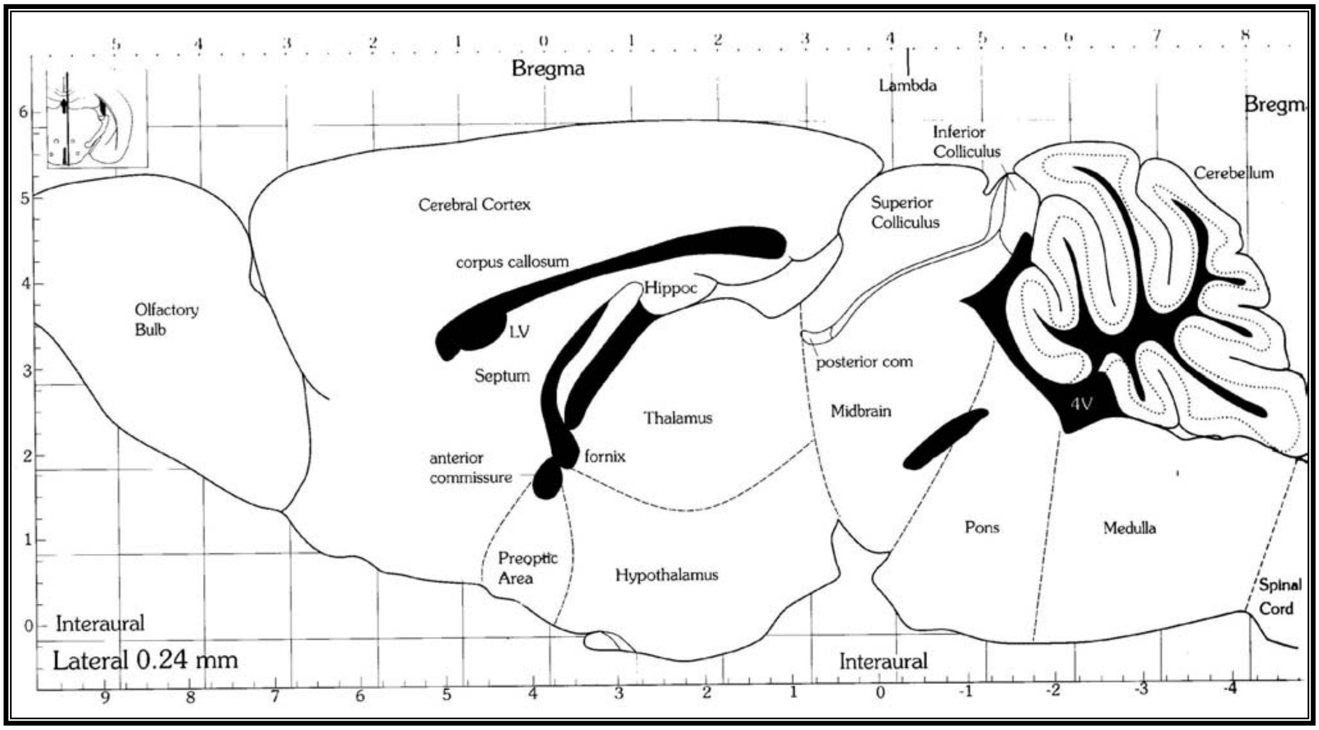

Supplement: Supplementary file 1 — SUPPLEMENT [file 41398_2021_1511_MOESM1_ESM.pdf]
